# Supplementary material for: Genome-wide meta-analysis of 241,258 adults accounting for smoking behaviour identifies novel loci for obesity traits
Source: Nat Commun. 2017 Apr 26;8:14977. doi: 10.1038/ncomms14977 (PMC5414044; doi:10.1038/ncomms14977)
Supplement: Supplementary Information — Supplementary Figures, Supplementary Tables, Supplementary Notes and Supplementary References [file ncomms14977-s1.pdf]

**Supplementary Figure 1**. **Summary of overall study design and workflow for meta-analyses**. All numbers provided represent the maximum number specific for that trait (BMI-red, WCadjBMI-blue, and WHRadjBMI-green) and strata (EUR-European descent participants, nonEUR-excluding European descent participants). Three studies provided GWAS data for EUR and nonEUR participants.

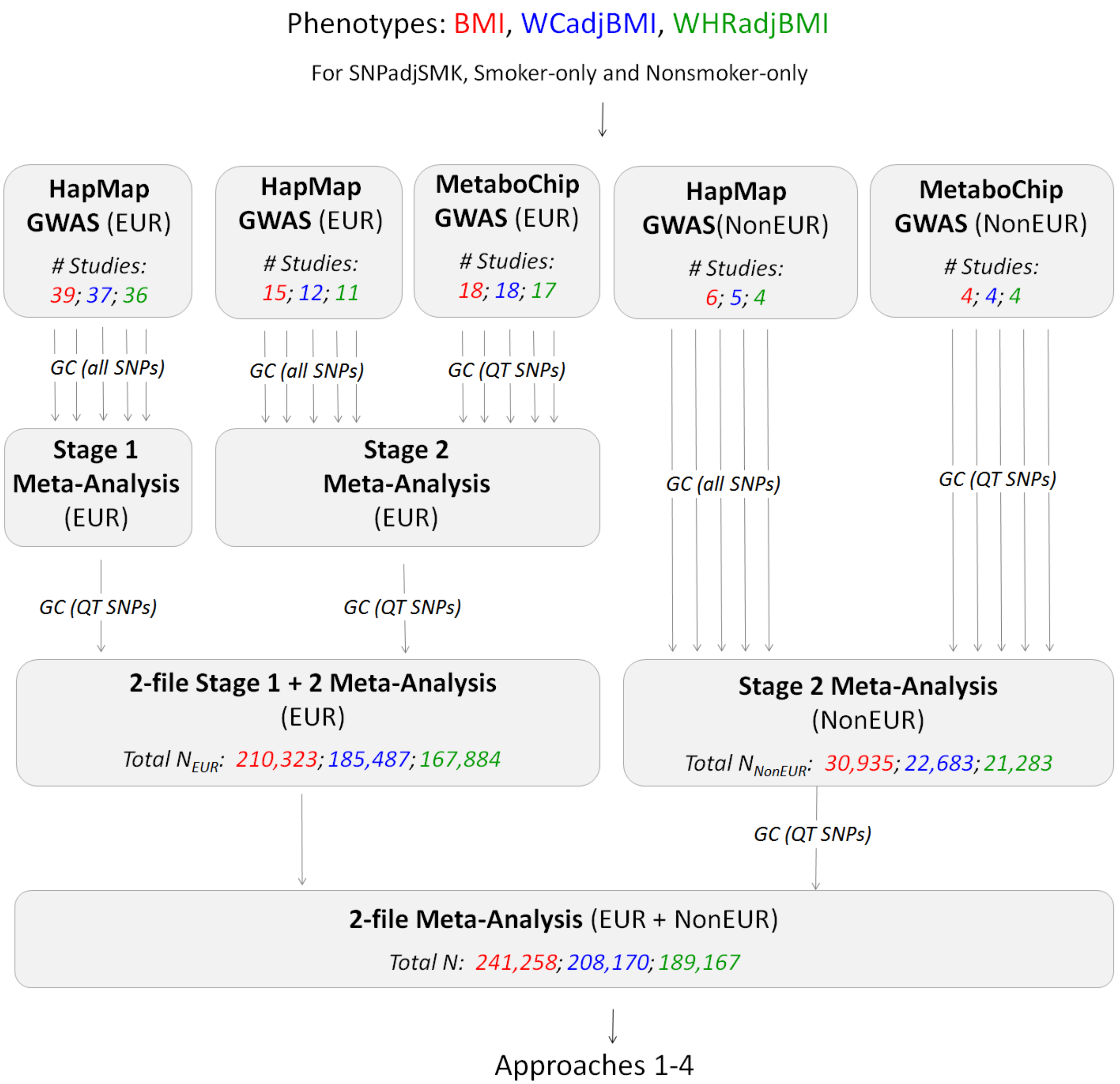

**Supplementary Figure 2**. **Summary plots of discovery meta-analysis for Approach 1 primary meta-analyses**. (**a**) Manhattan plot showing the loci identified in Approach 1 in primary meta-analyses, used to identify significant main effects loci (SNPadjSMK), in the primary meta-analyses association –log10P-values for BMI-red, WCadjBMI-blue, and WHRadjBMI-green; (**b**) Manhattan plot showing the loci identified in Approach 1 excluding known regions +/- 500 kb and labeled with the nearest gene to the index SNP; (**c**) QQ-plot showing the Approach 1 P-values as observed against those expected under the null for each phenotypes separately (colored); (**d**) QQ-plot for Approach 1 after excluding known association regions. **PSMB10* locus is >500 +/- kb from previously identified index SNPs, but is not independent of known GWAS signals.

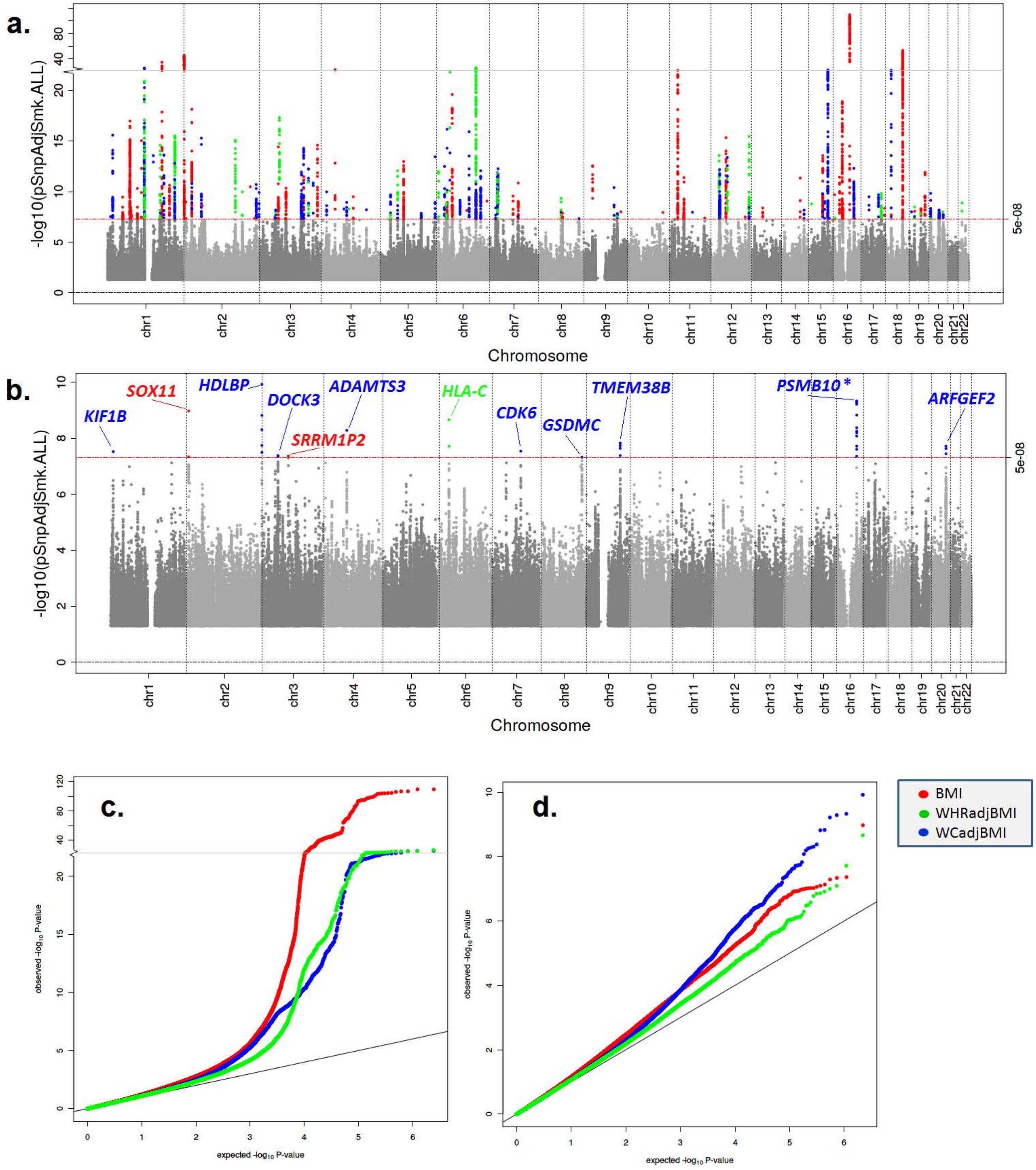

**Supplementary Figure 3**. **Regional association plots for Approach 1 primary meta-analyses**. Regional association plots for all novel loci identified in Approach 1 (SNPadjSMK) in primary meta-analyses for BMI: (**a**) rs10929925, (**b**) rs6794880; WCadjBMI: (**c**) rs17396340, (**d**) rs6743226, (**e**) rs4378999, (**f**) rs7697556, (**g**) rs10269774, (**h**) rs6470765, (**i**) rs9409082, (**j**) rs6012558; and WHRadjBMI: (**k**) rs1049281, and ordered as they appear in Table 1. LD has been calculated using the combined ancestries from the 1000 Genomes Phase 1 reference panel. For comparison, each plot highlights the p-value for the tag SNP in Approach 1 ($P_{adjSMK}$), Approach 2 ($P_{joint}$), Approach 3 ($P_{int}$), current smokers ($P_{SMK}$), and in nonsmokers ($P_{nonSMK}$).

## a. BMI: rs10929925 −Approach 1

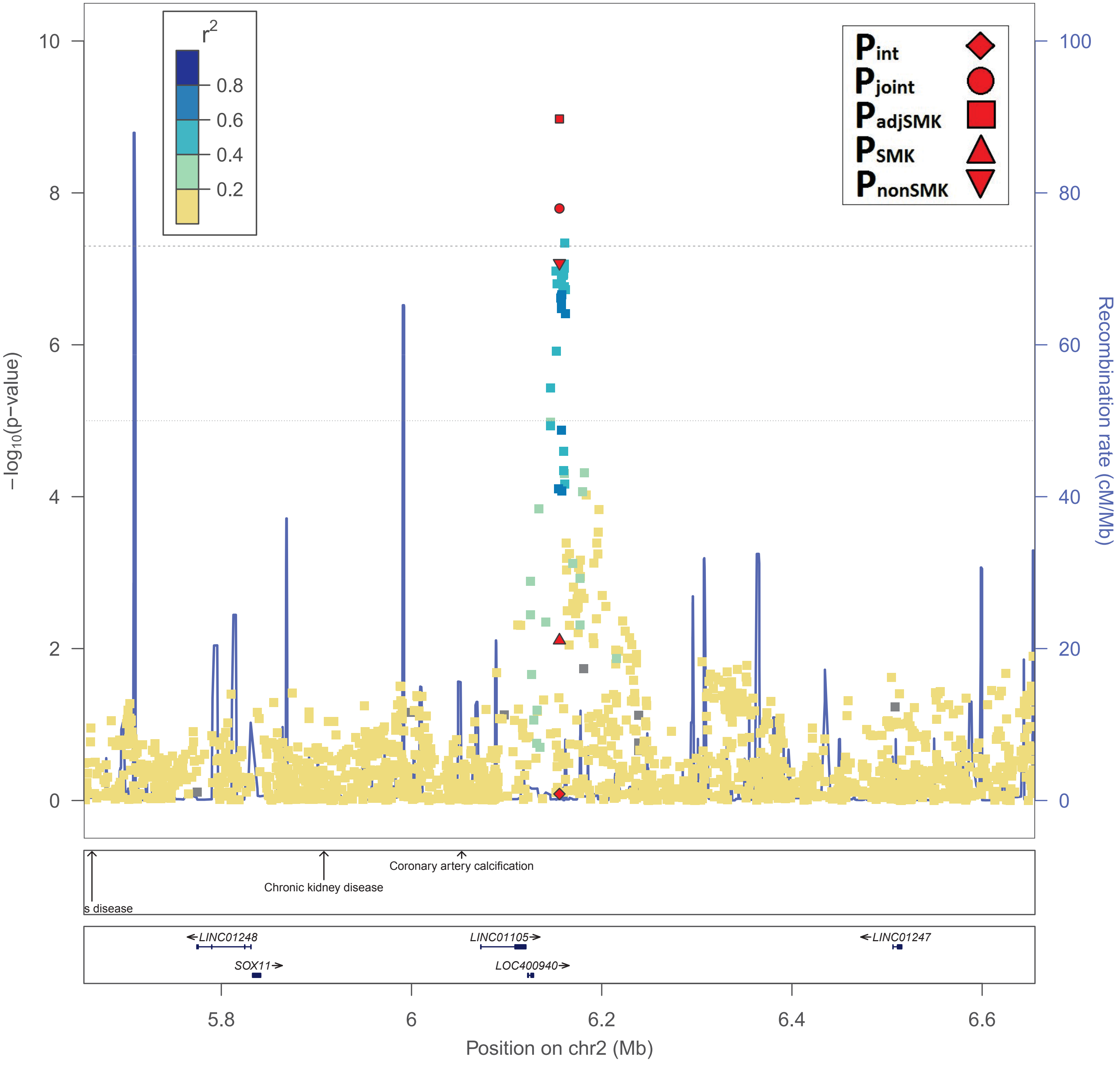

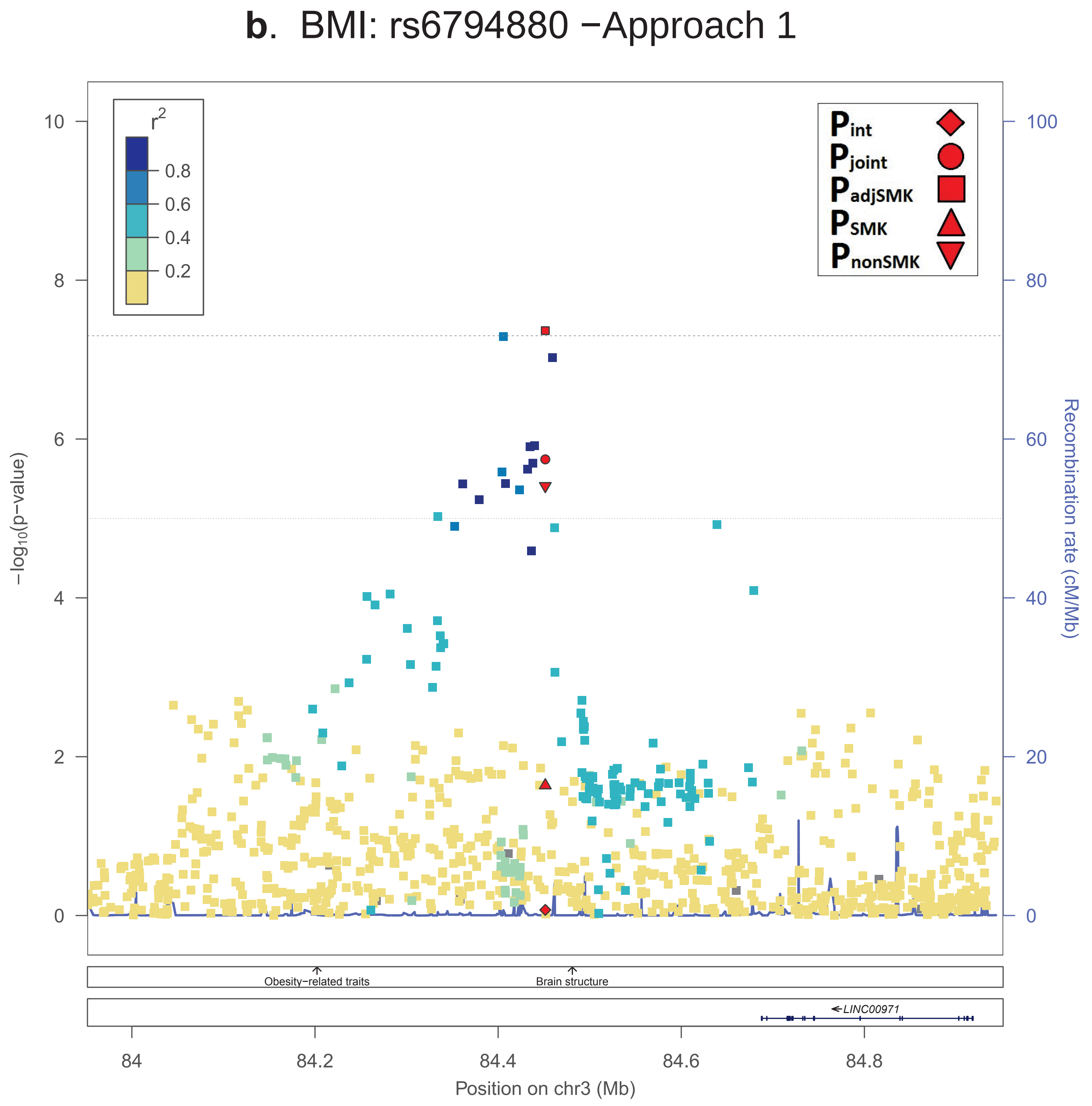

b. BMI: rs6794880 −Approach 1
r2
0.8
0.6
0.4
0.2
Pint
Pjoint
PadjSMK
PSMK
PnonSMK
−log10(p−value)
Recombination rate (cM/Mb)
Obesity−related traits
Brain structure
LINC00971
Position on chr3 (Mb)

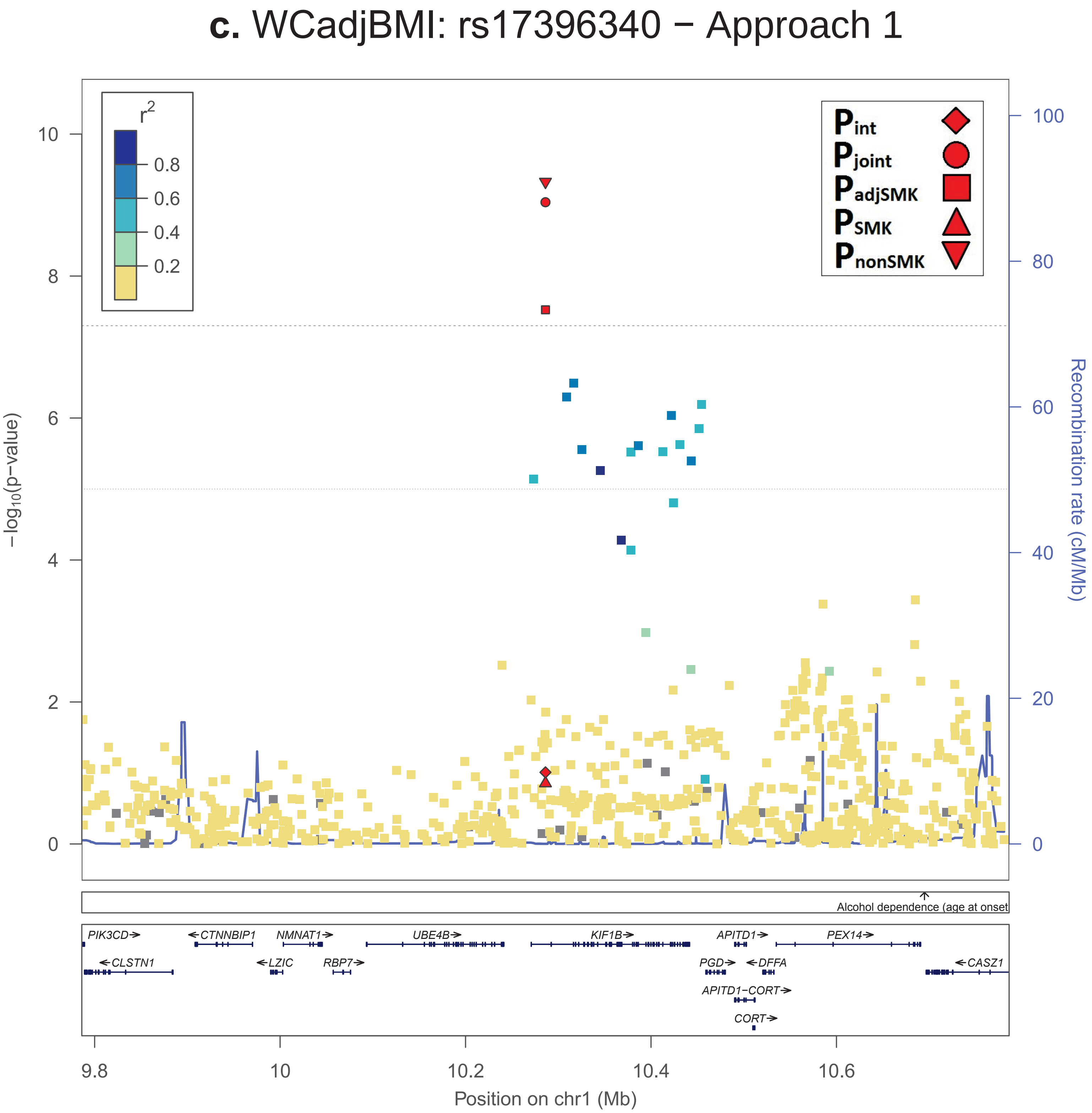
c. WCadjBMI: rs17396340 − Approach 1
r2
0.8
0.6
0.4
0.2
Pint
Pjoint
PadjSMK
PSMK
PnonSMK
−log10(p−value)
Recombination rate (cM/Mb)
Alcohol dependence (age at onset
PIK3CD
CTNNBIP1
NMNAT1
UBE4B
KIF1B
APITD1
PEX14
CLSTN1
LZIC
RBP7
PGD
DFFA
CASZ1
APITD1−CORT
CORT
Position on chr1 (Mb)

**d.** WCadjBMI: rs6743226 − Approach 1

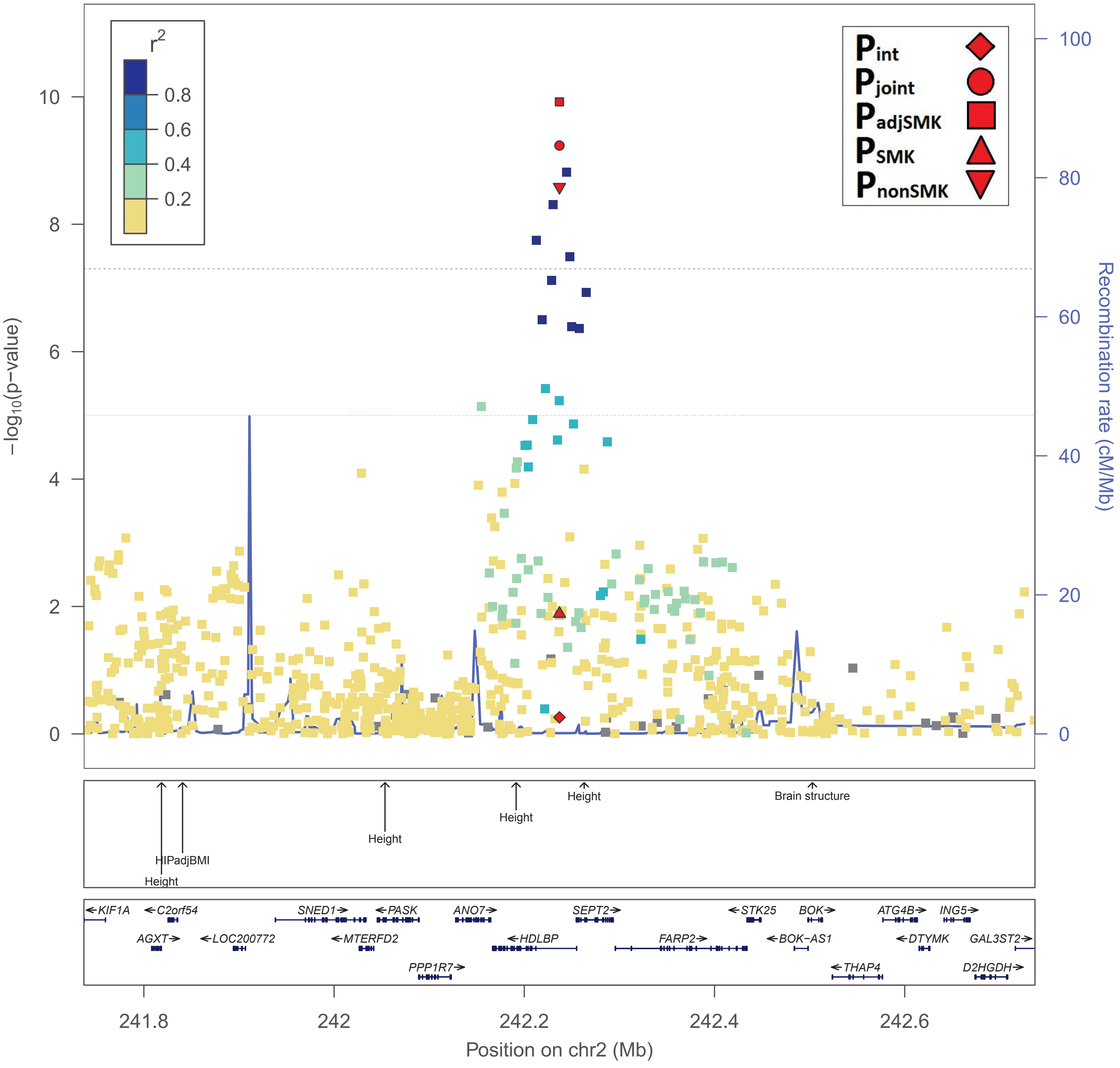

## e. WCadjBMI: rs4378999 − Approach 1

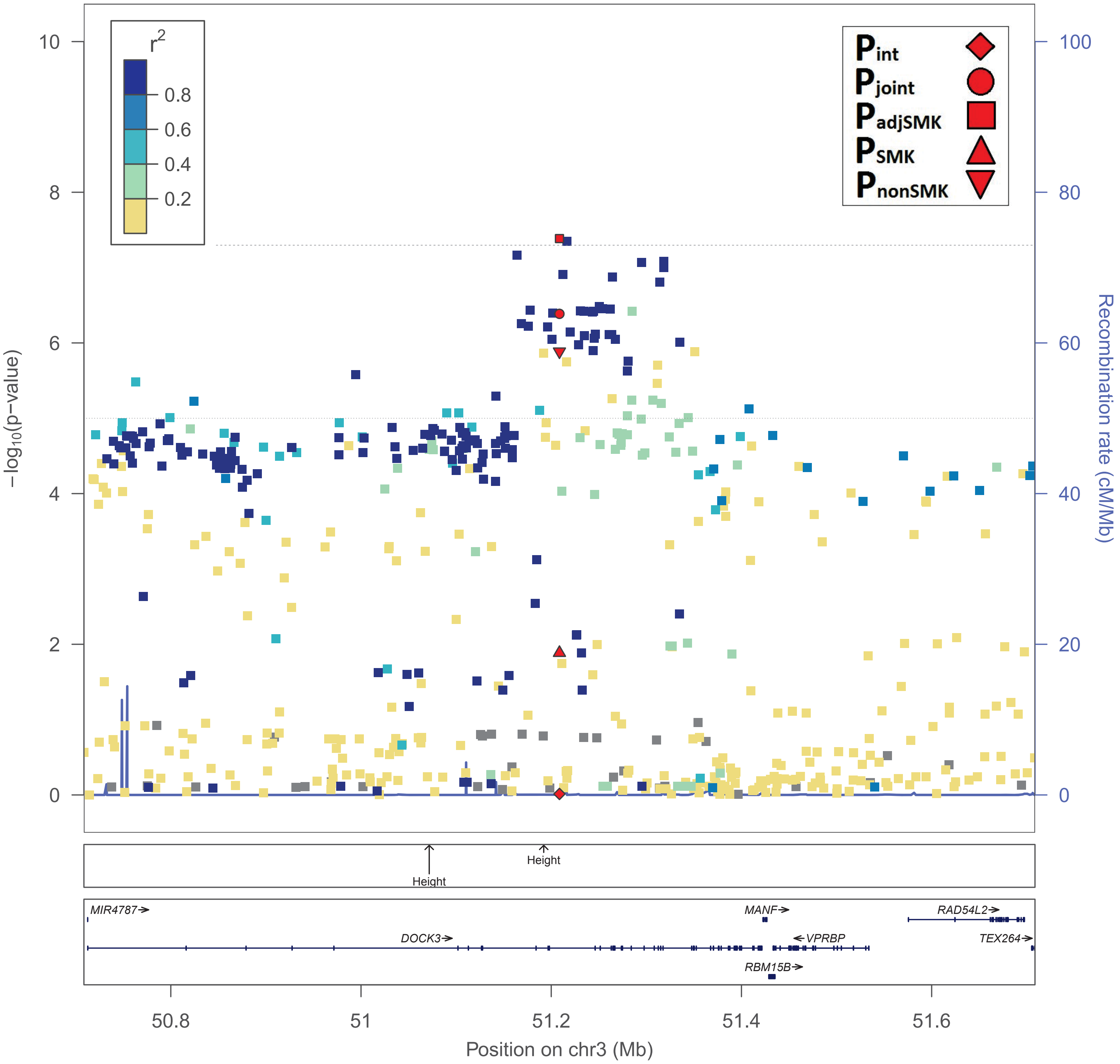

**f.** WCadjBMI: rs7697556 − Approach 1

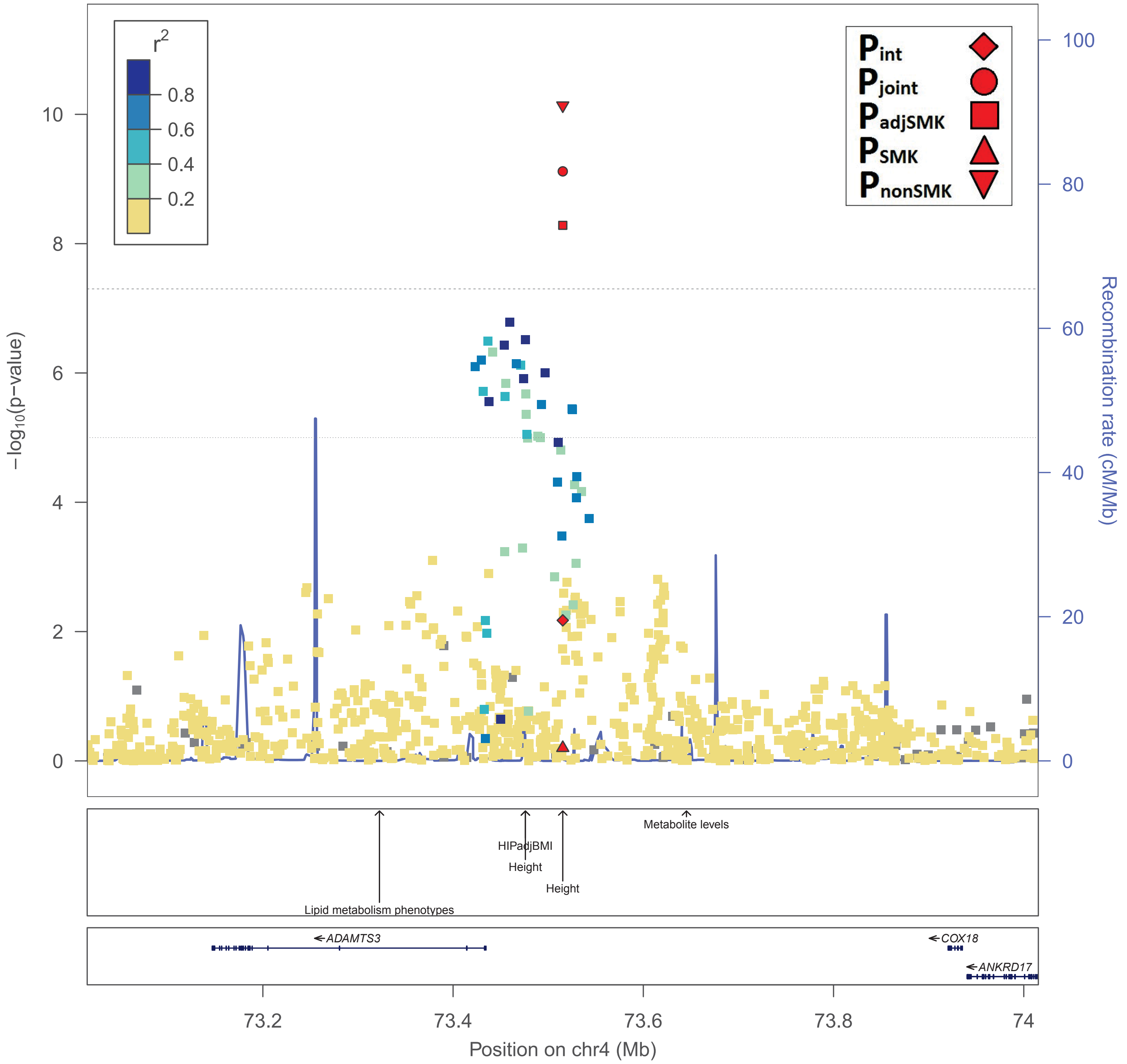

**g.** WCadjBMI: rs10269774 − Approach 1

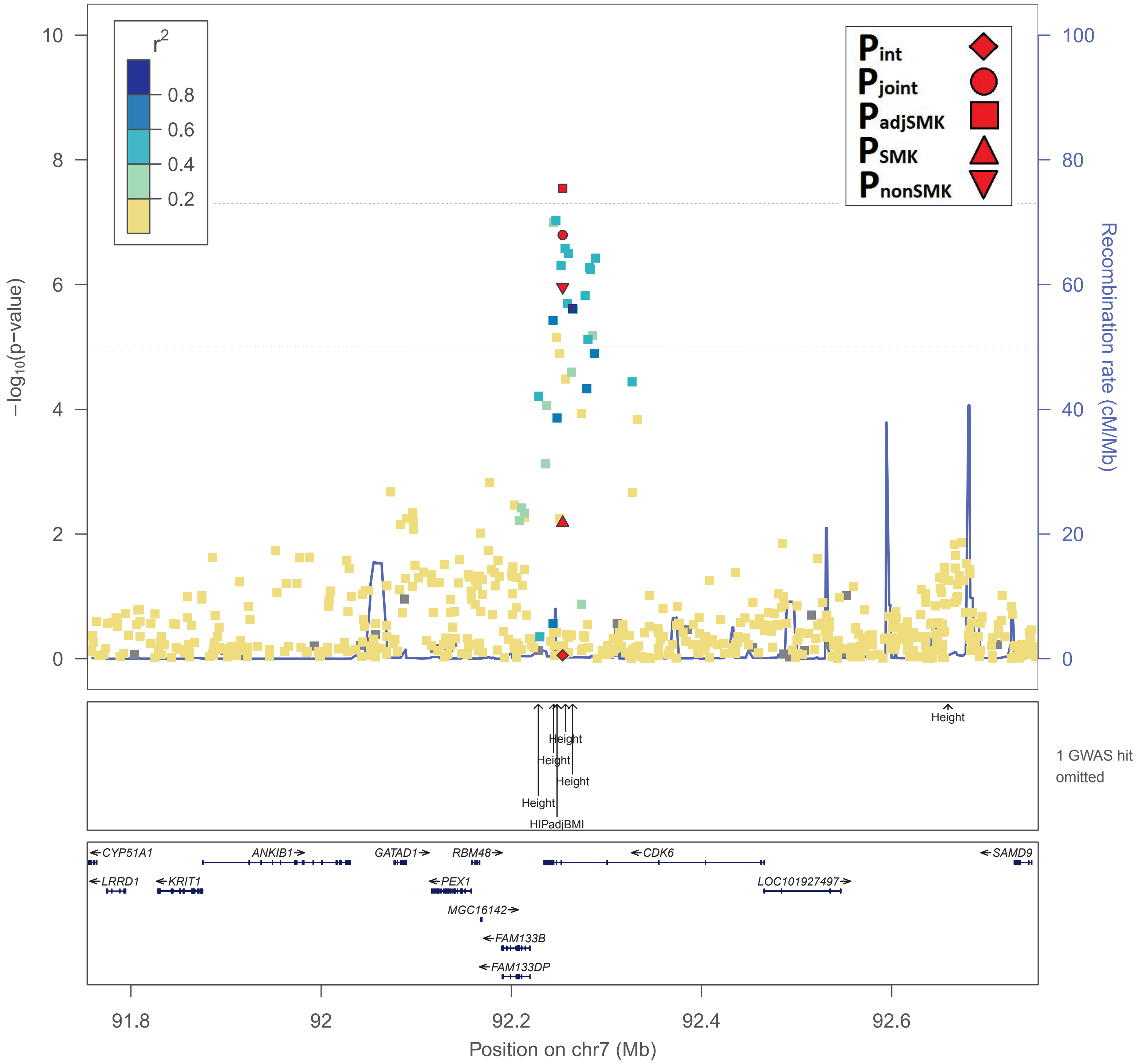

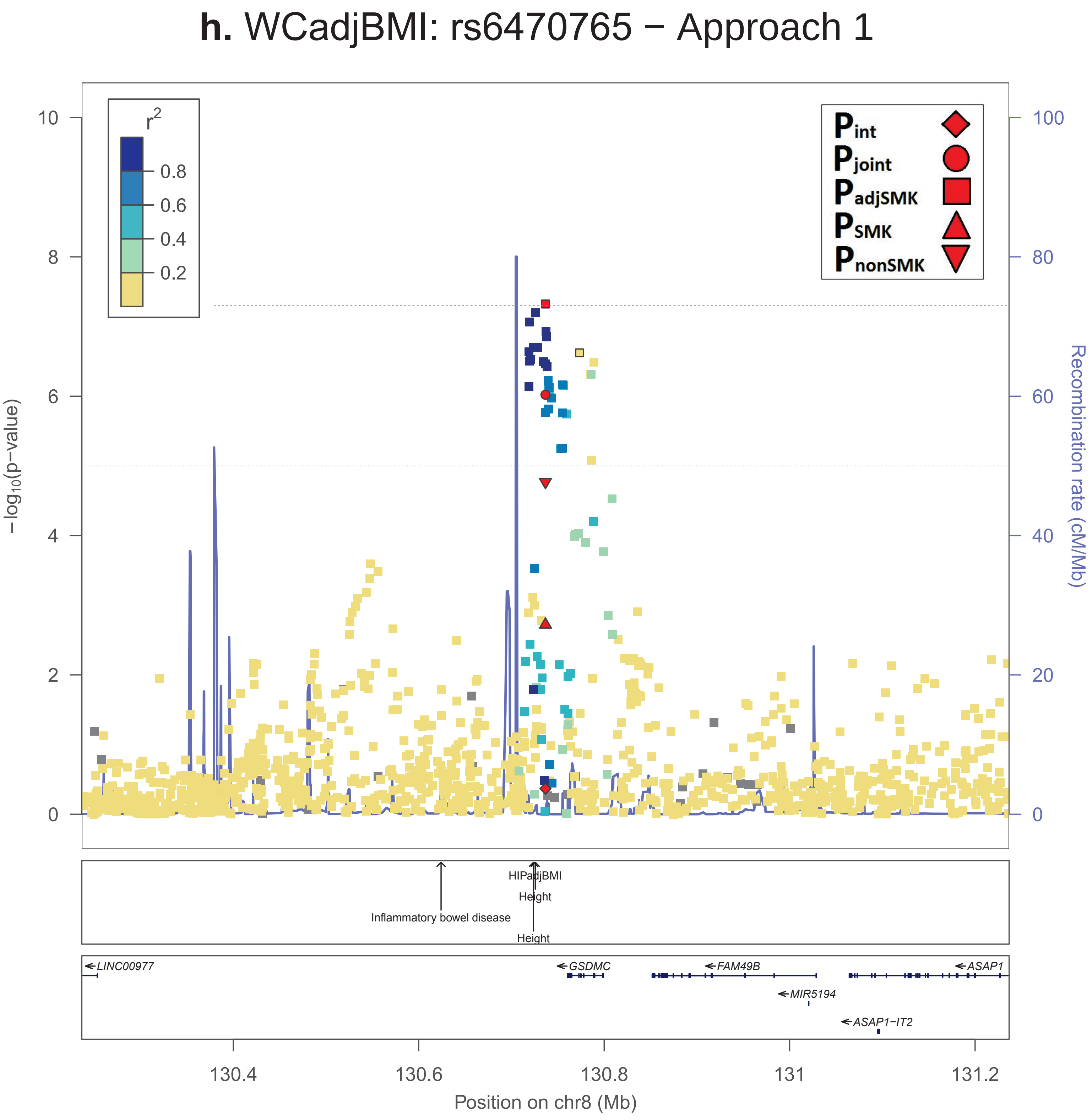

h. WCadjBMI: rs6470765 − Approach 1
r2
0.8
0.6
0.4
0.2
Pint
Pjoint
PadjSMK
PSMK
PnonSMK
−log10(p−value)
Recombination rate (cM/Mb)
HIPadjBMI
Height
Inflammatory bowel disease
Height
LINC00977
GSDMC
FAM49B
ASAP1
MIR5194
ASAP1−IT2
130.4
130.6
130.8
131
131.2
Position on chr8 (Mb)

i. WCadjBMI: rs9409082 − Approach 1

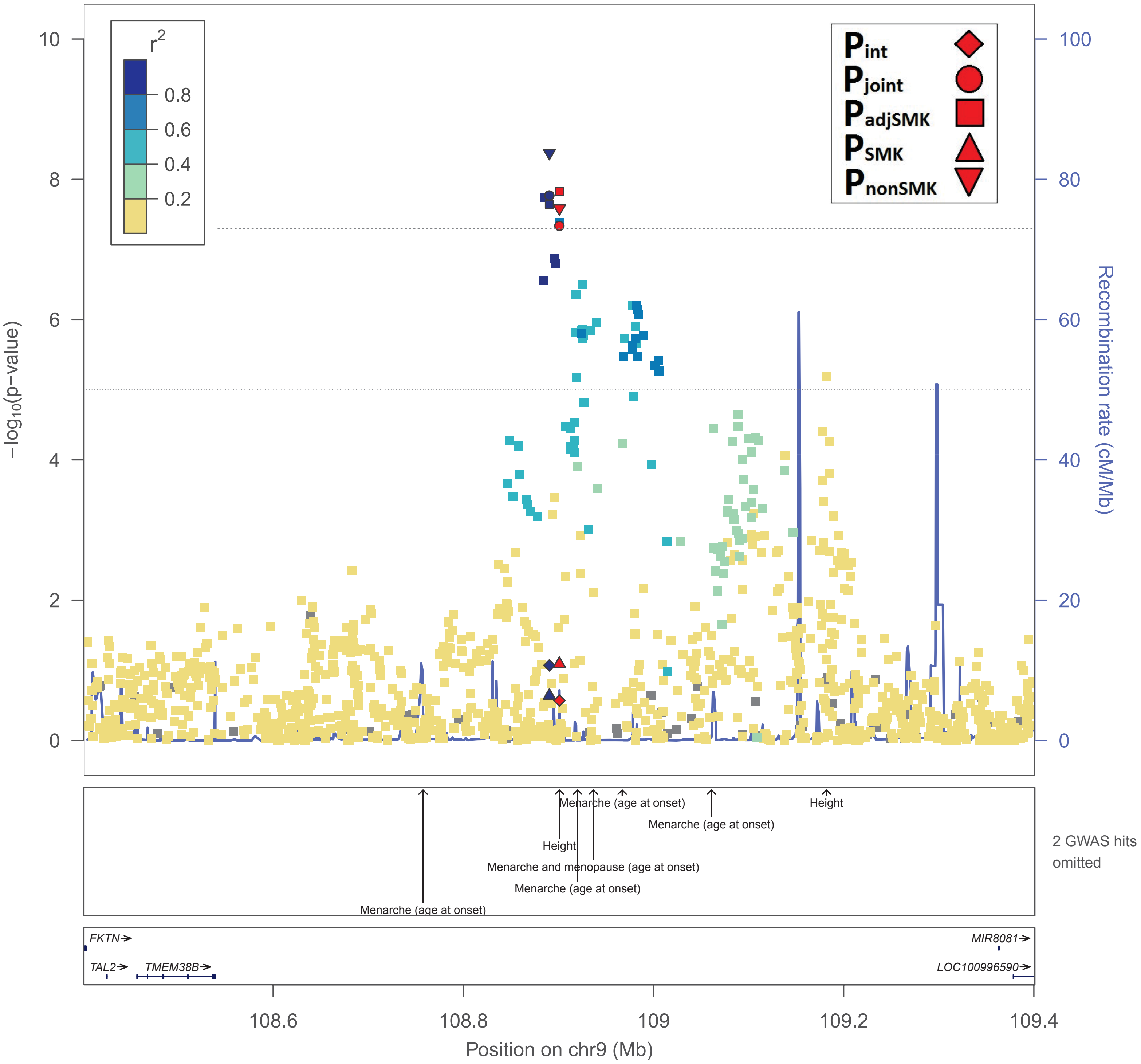

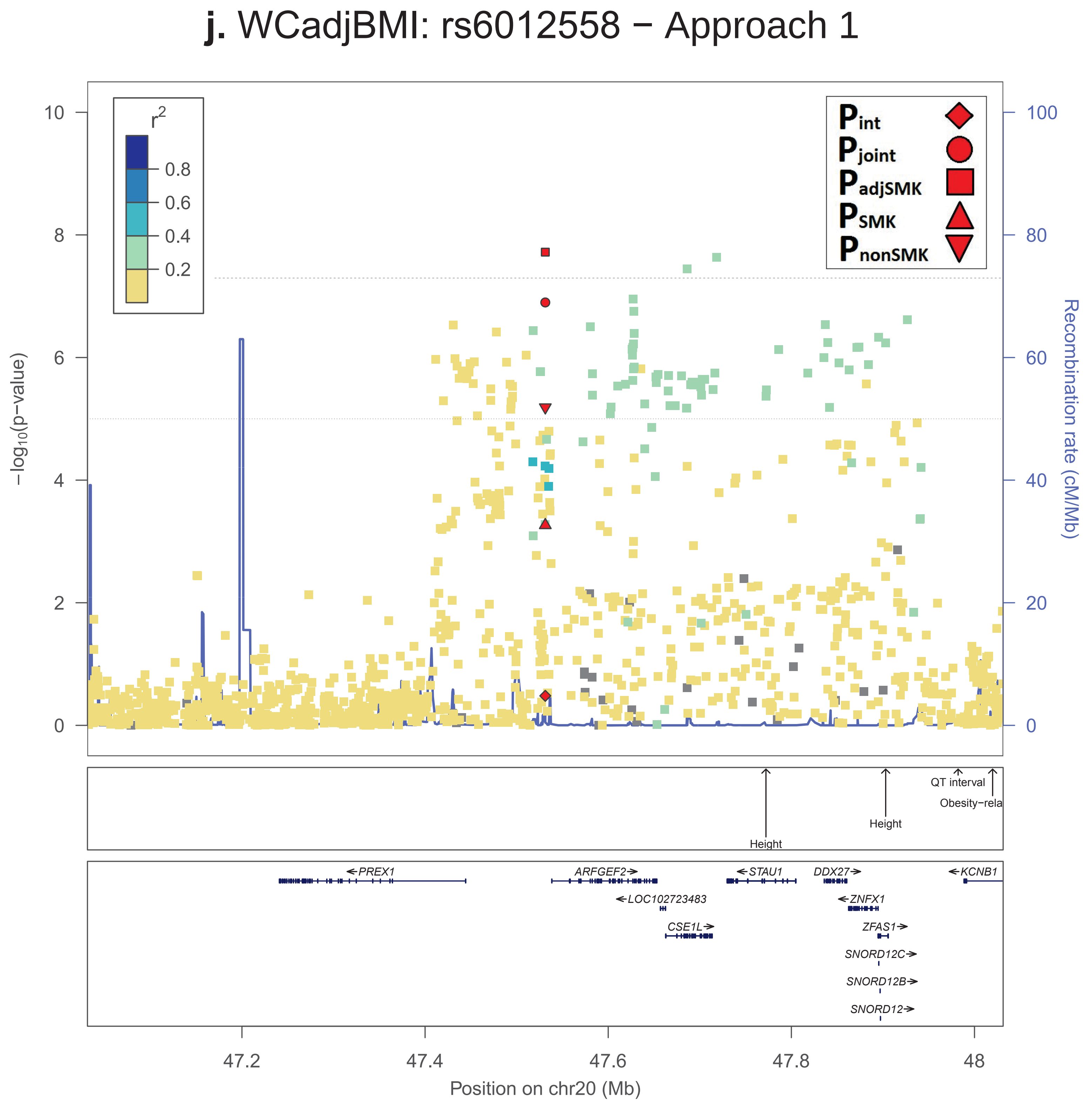
j. WCadjBMI: rs6012558 − Approach 1
r2
0.8
0.6
0.4
0.2
Pint
Pjoint
PadjSMK
PSMK
PnonSMK
−log10(p−value)
Recombination rate (cM/Mb)
Height
Height
QT interval
Obesity−rela
PREX1
ARFGEF2
LOC102723483
CSE1L
STAU1
DDX27
ZNFX1
ZFAS1
SNORD12C
SNORD12B
SNORD12
KCNB1
Position on chr20 (Mb)

## **k**. WHRadjBMI: rs1049281 − Approach 1

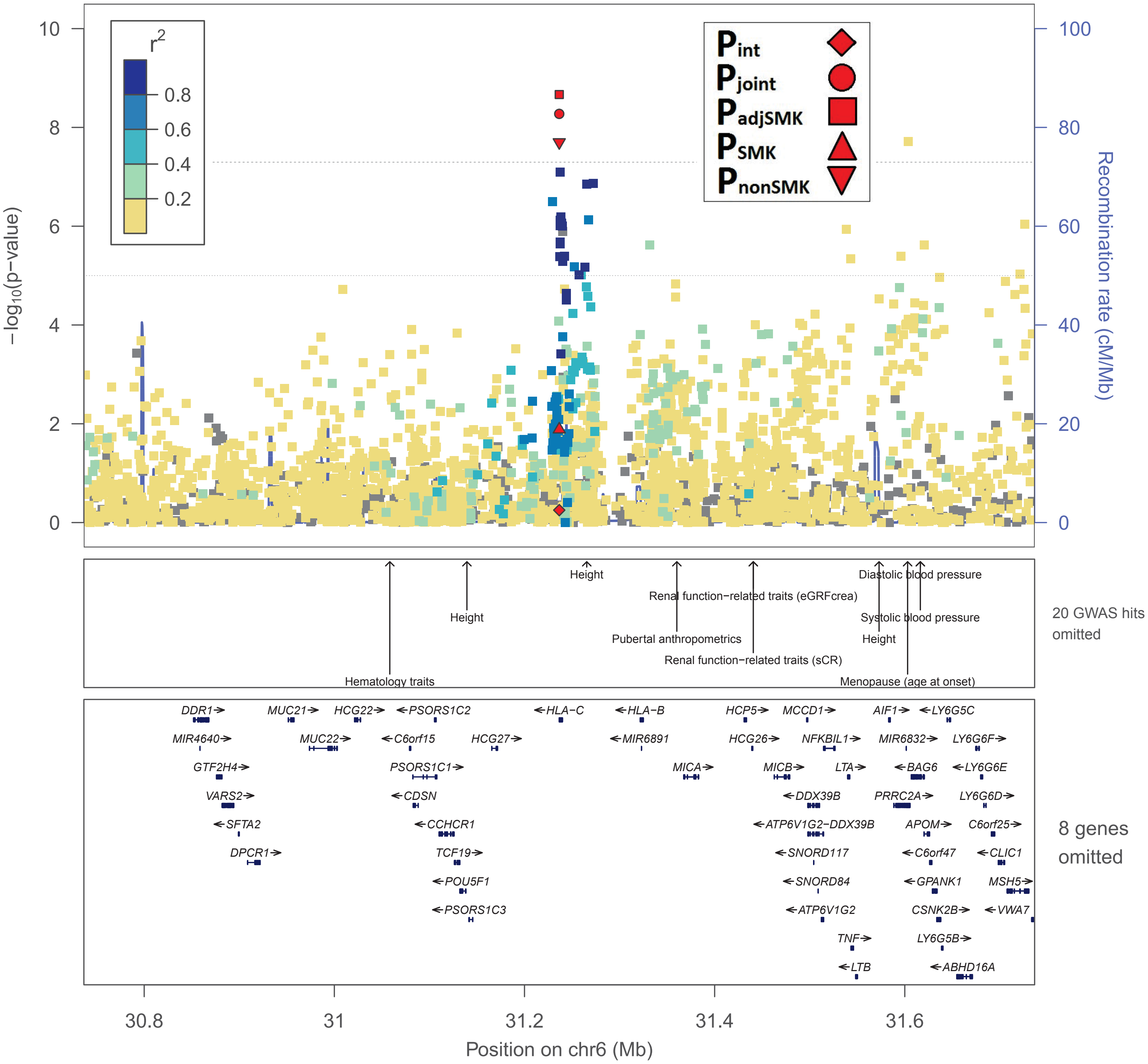

**Supplementary Figure 4**. **Summary plots of discovery meta-analysis for Approach 2 primary meta-analyses**. (**a**) Manhattan plot showing the loci identified in Approach 2 in primary meta-analyses, used to identify significant joint main+interaction effects loci (SNPjoint), in the primary meta-analyses association –log10P-values for BMI-red, WCadjBMI-blue, and WHRadjBMI-green; (**b**) Manhattan plot showing the loci identified in Approach 2 excluding known regions +/- 500 kb and labeled with the nearest gene to the index SNP; (**c**) QQ-plot showing the Approach 2 P-values as observed against those expected under the null for each phenotypes separately (colored); (**d**) QQ-plot for Approach 2 after excluding known association regions.

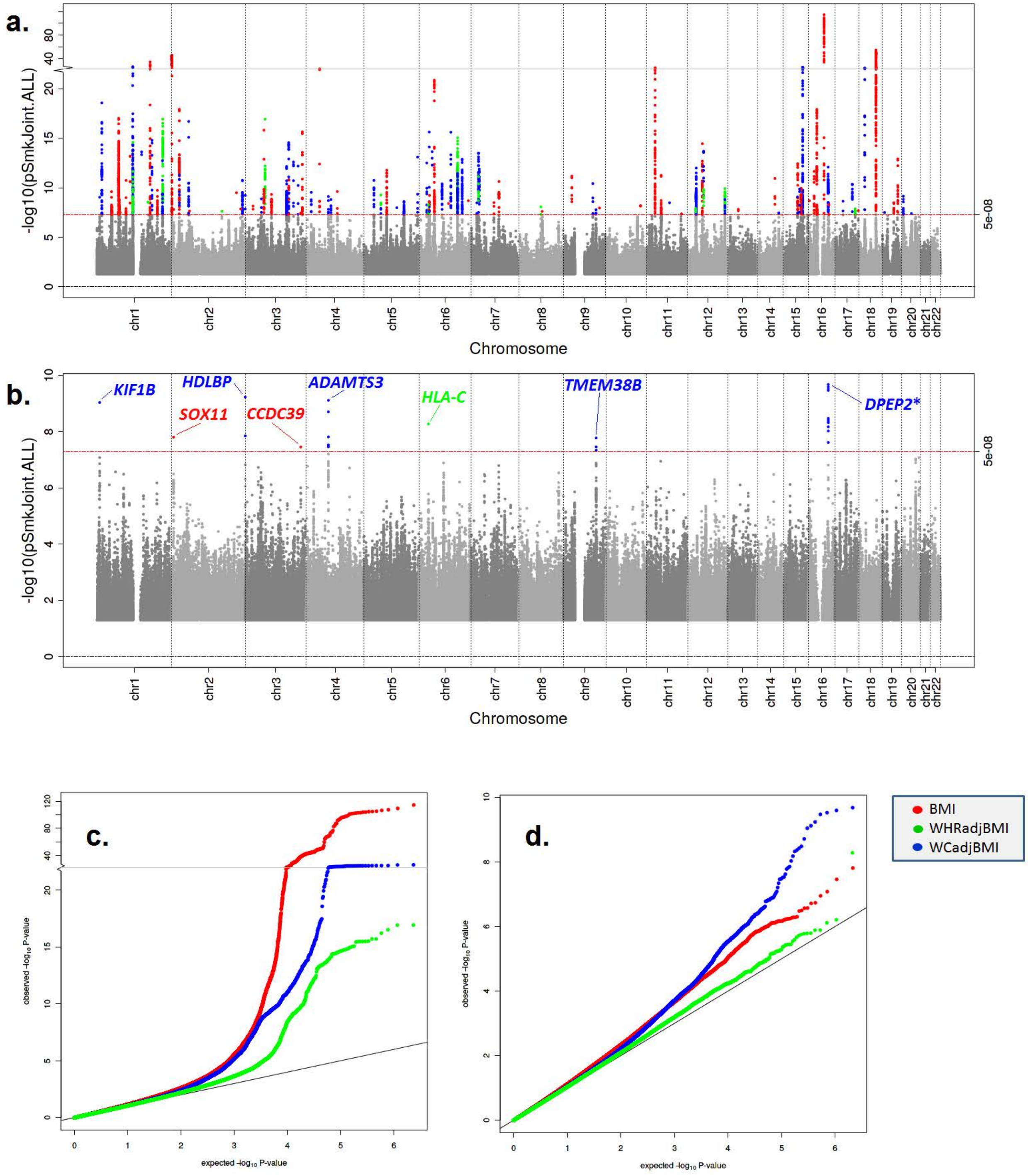

**Supplementary Figure 5**. **Regional association plots for Approach 2 primary meta-analyses**. Regional association plot for all novel loci identified in Approach 2 (SNPjoint) in the primary meta-analyses for BMI: (**a**) rs10929925, (**b**) rs13069244; WCadjBMI: (**c**) rs17396340, (**d**) rs6743226, (**e**) rs7697556, (**f**) rs9408815, and WHRadjBMI: (**g**) rs1049281, and ordered as they appear in Table 1. LD has been calculated using the combined ancestries from the 1000 Genomes Phase 1 reference panel. For comparison, each plot highlights the p-value for the tag SNP in Approach 1 ($P_{adjSMK}$), Approach 2 ($P_{joint}$), Approach 3 ($P_{int}$), current smokers ($P_{SMK}$), and in nonsmokers ($P_{nonSMK}$).

# a. BMI: rs10929925 − Approach 2

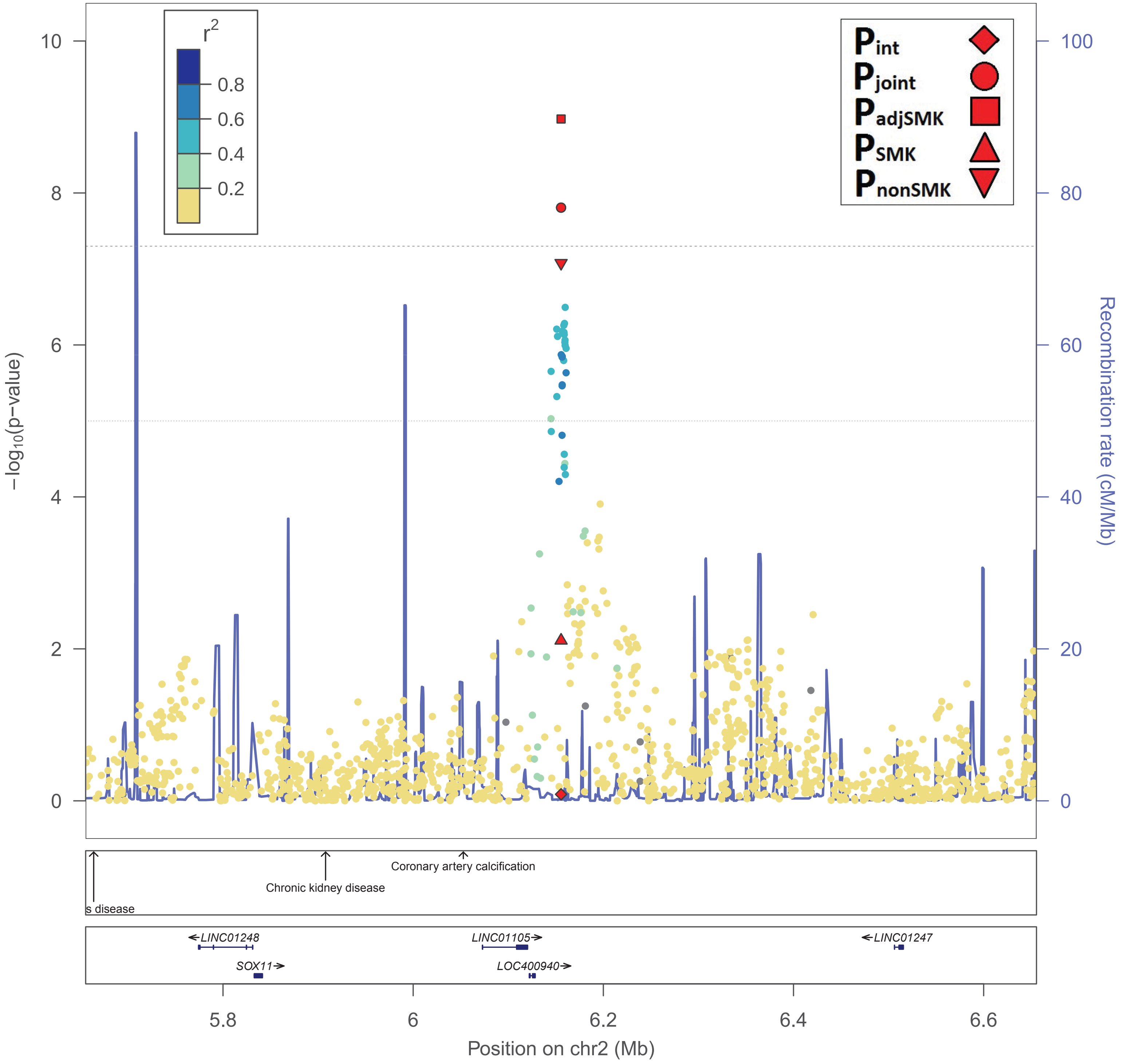

# **b.** BMI: rs13069244 − Approach 2

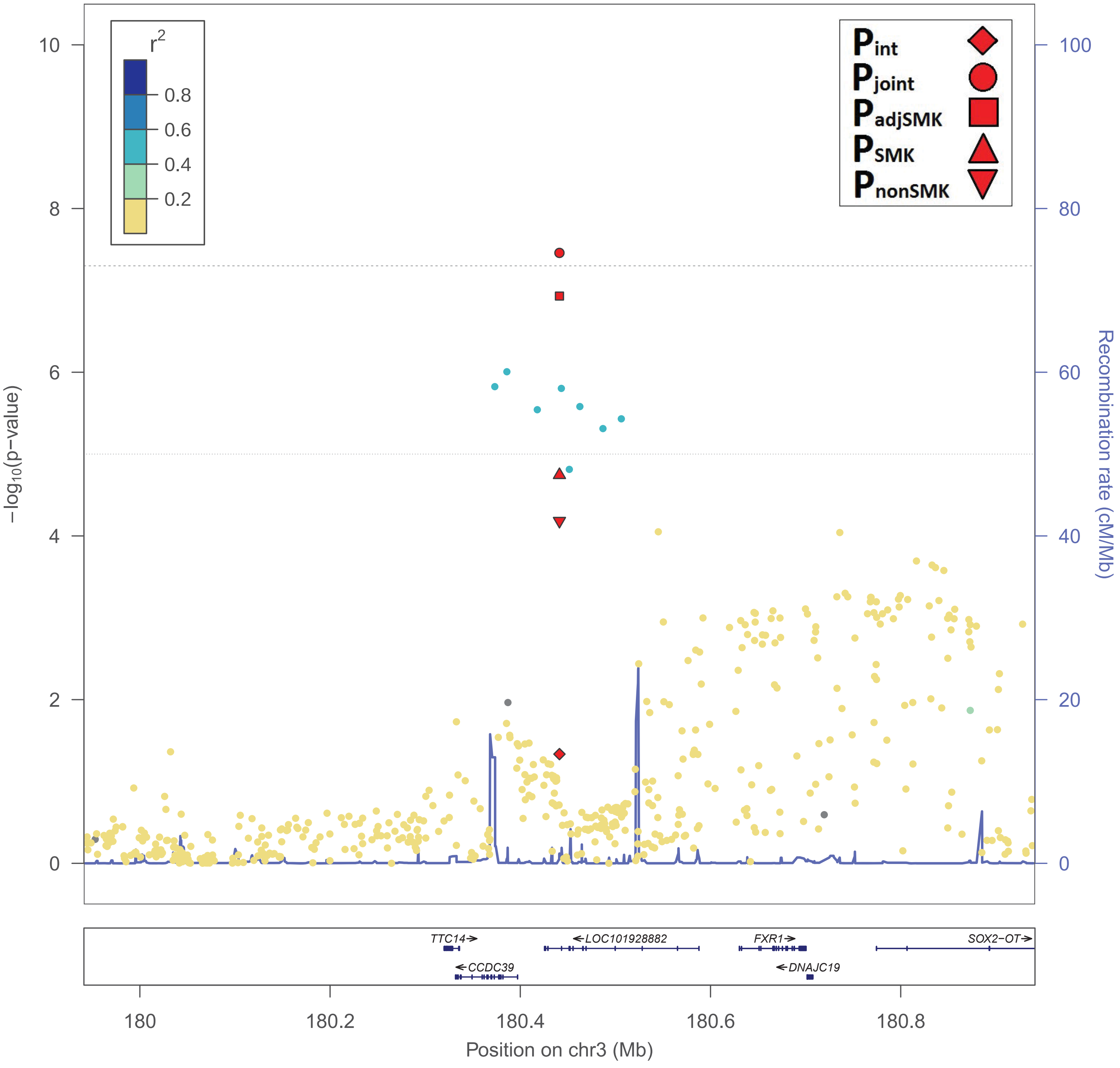

# **c.** WCadjBMI: rs17396340 − Approach 2

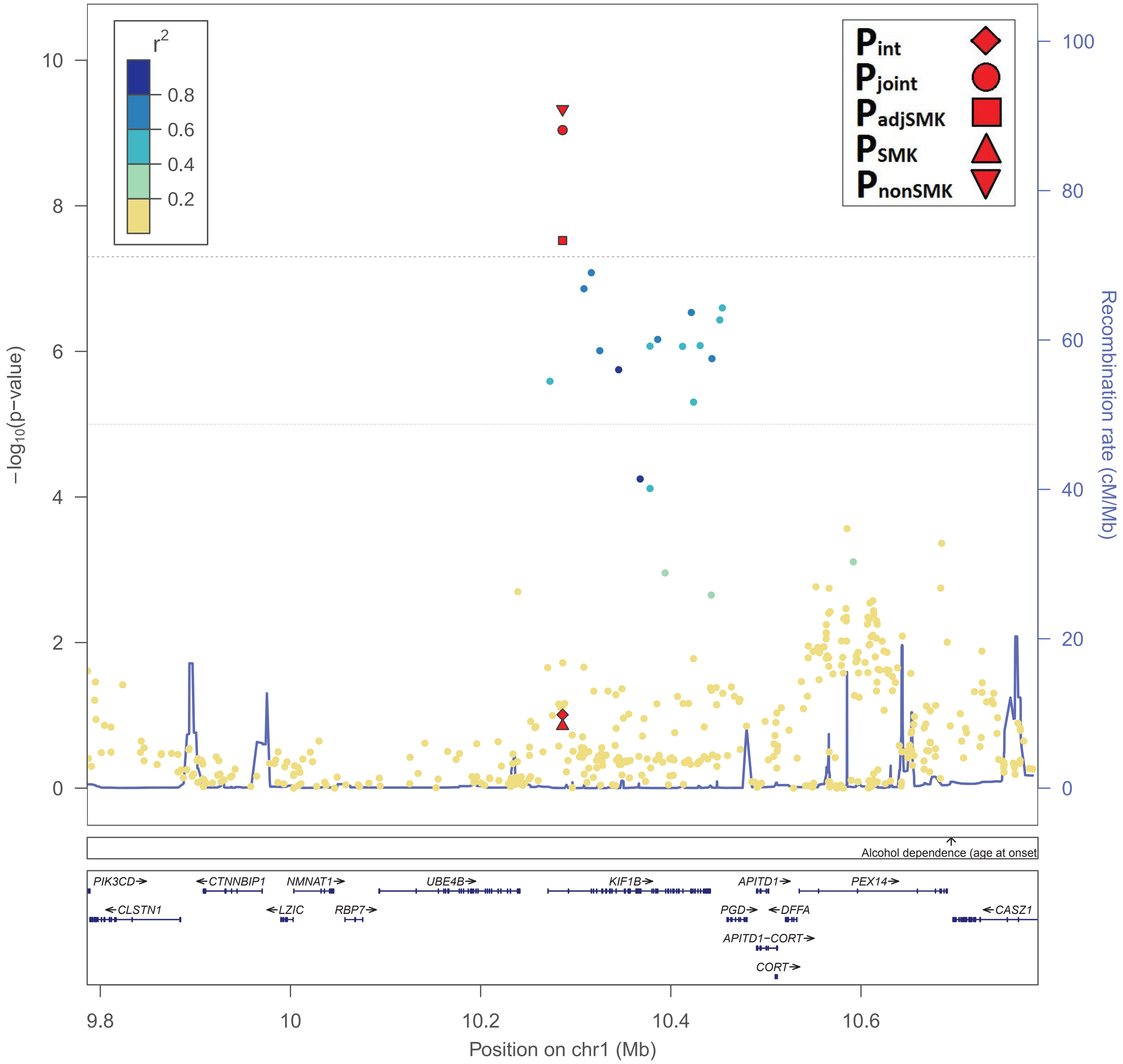

**d.** WCadjBMI: rs6743226 − Approach 2

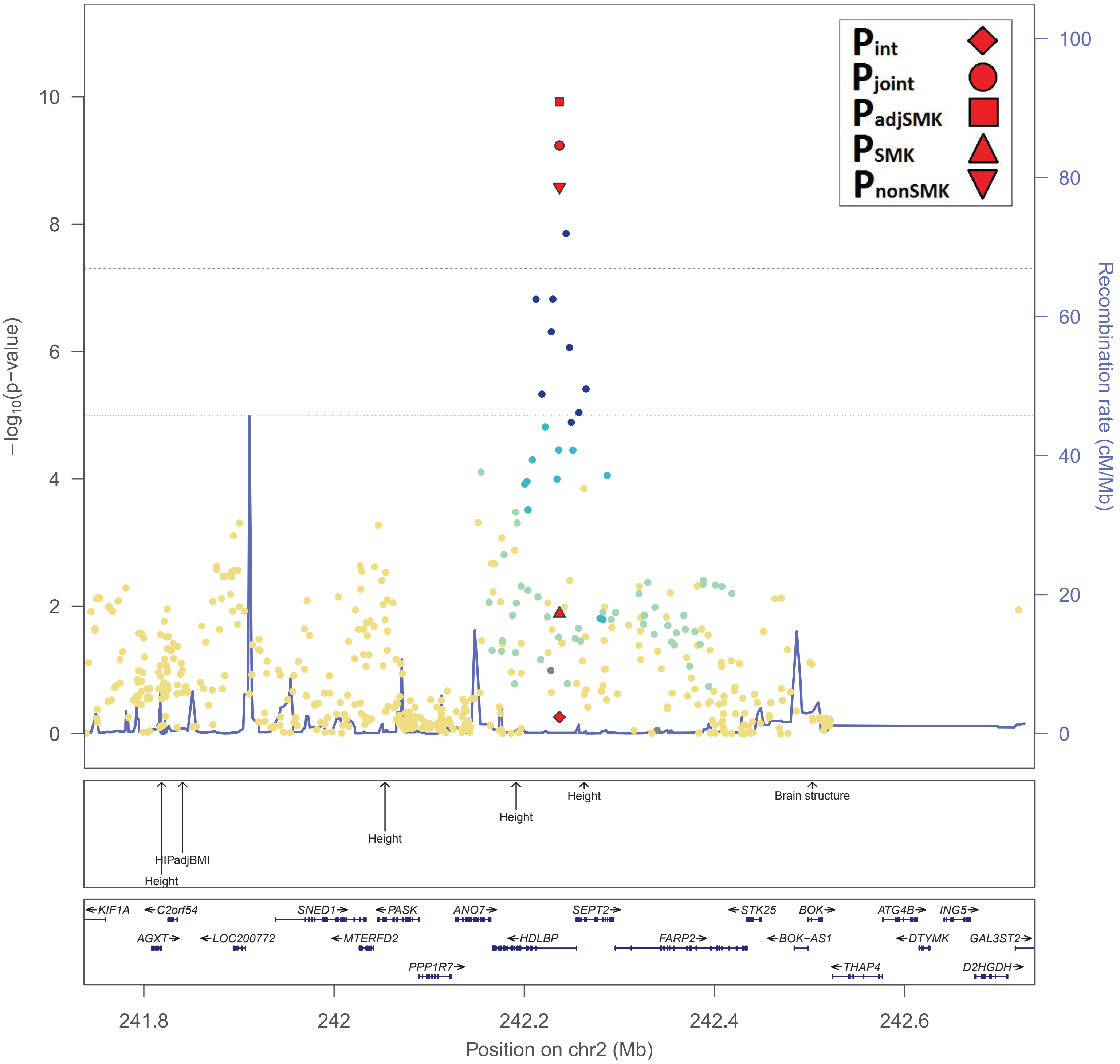

# e. WCadjBMI: rs7697556 − Approach 2

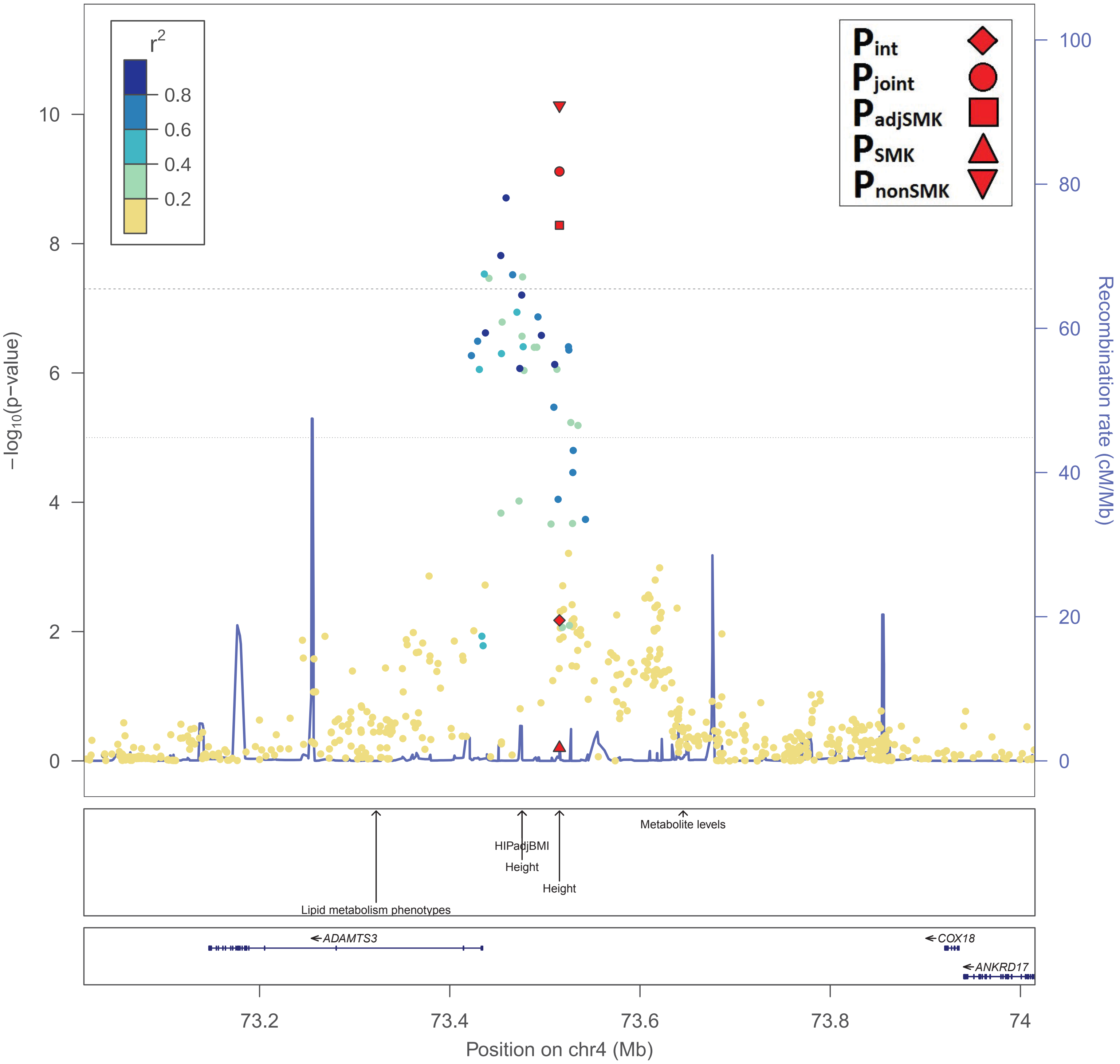

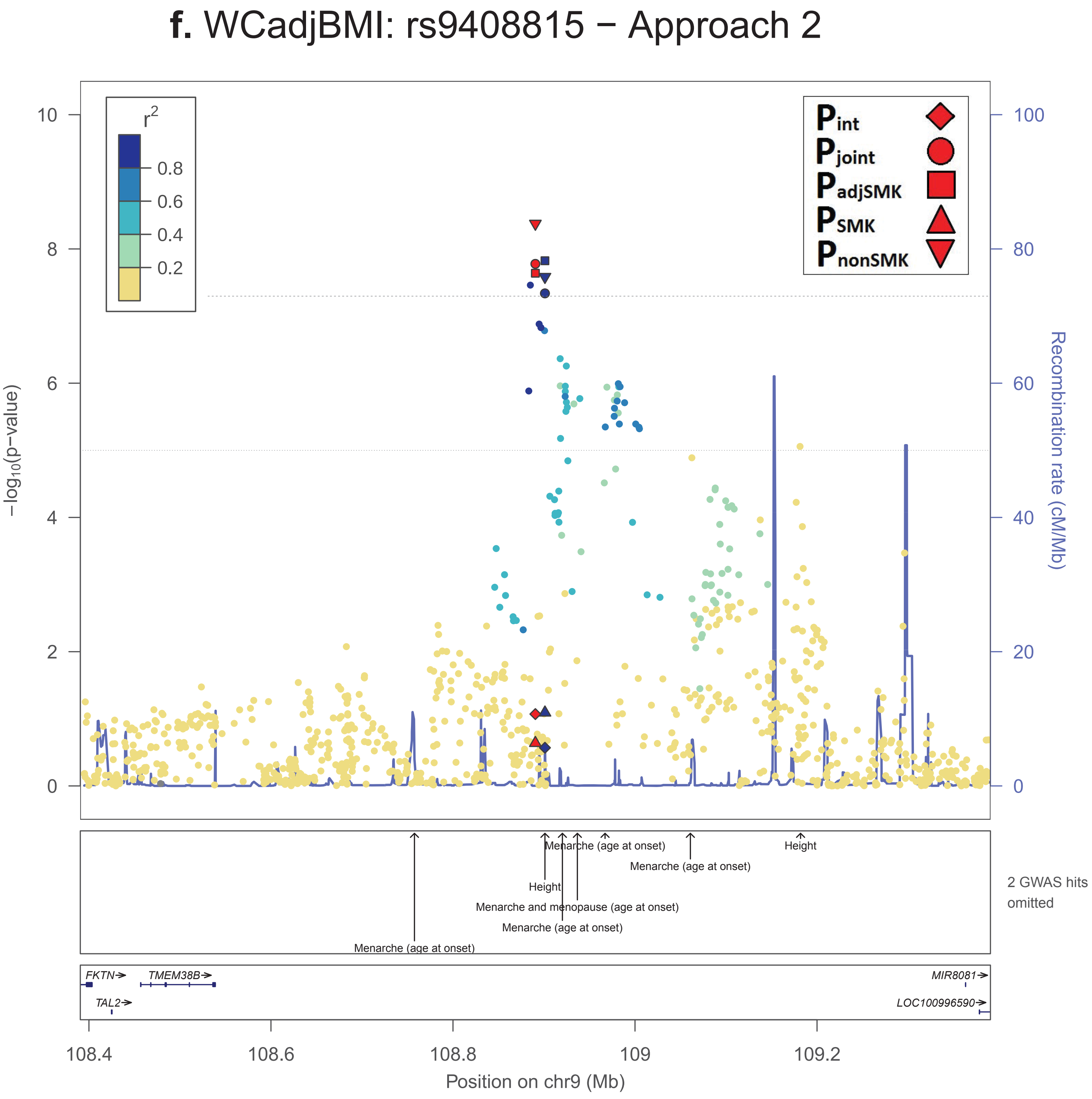
f. WCadjBMI: rs9408815 − Approach 2
r2
0.8
0.6
0.4
0.2
Pint
Pjoint
PadjSMK
PSMK
PnonSMK
−log10(p−value)
Recombination rate (cM/Mb)
Menarche (age at onset)
Height
Menarche (age at onset)
Height
Menarche and menopause (age at onset)
Menarche (age at onset)
Menarche (age at onset)
2 GWAS hits omitted
FKTN
TMEM38B
TAL2
MIR8081
LOC100996590
108.4
108.6
108.8
109
109.2
Position on chr9 (Mb)

# g. WHRadjBMI: rs1049281 − Approach 2

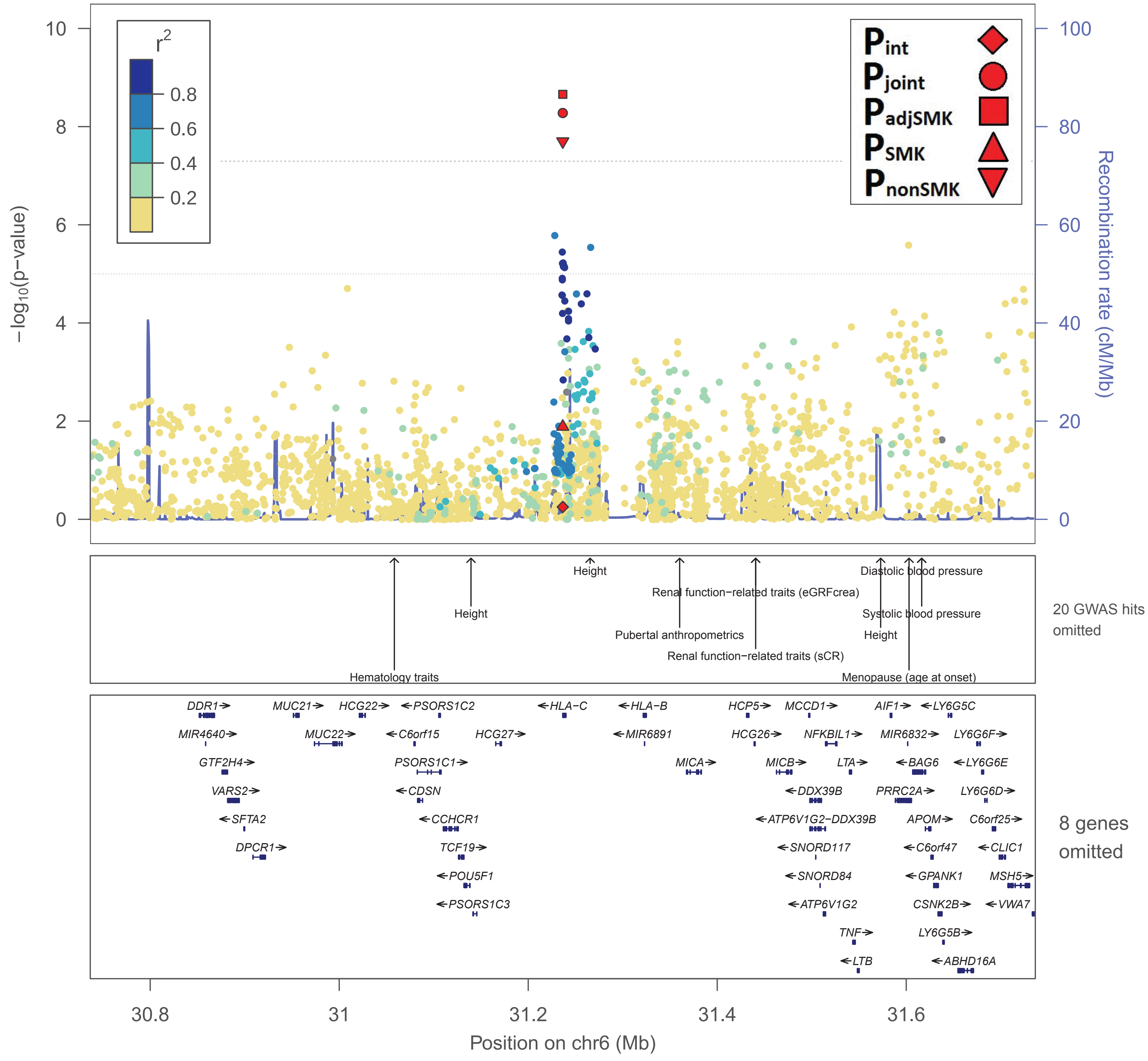

**Supplementary Figure 6**. **Regional association plots for Approaches 1-3 secondary meta-analyses**. Regional association plot for additional novel loci identified in Approaches 1 (SNPadjSMK), Approach 2 (SNPjoint), or Approach 3 (SNPint) secondary meta-analyses for BMI: (**a**) rs2481665, (**b**) rs2173039, (**c**) rs12629427; WCadjBMI: (**d**) rs1545348, (**e**) rs6076699 (Approach 2), (**f**) rs6076699 (Approach 3), (**g**)  rs670752; and WHRadjBMI: (**h**) rs589428, (**i**) rs1856293, (**j**) rs2001945, (**k**) rs17065323. LD has been calculated using the combined ancestries from the 1000 Genomes Phase 1 reference panel. For comparison, each plot highlights the p-value for the tag SNP in Approach 1 ($P_{adjSMK}$), Approach 2 ($P_{joint}$), Approach 3 ($P_{int}$), current smokers ($P_{SMK}$), and in nonsmokers ($P_{nonSMK}$). P-values are shown from the strata in which the signal was identified (e.g. European-only women). EUR- European-only meta-analyses; ALL- all ancestries combined meta-analyses.

# **a.** BMI: rs2481665 − Approach 1, EUR, Combined Sexes

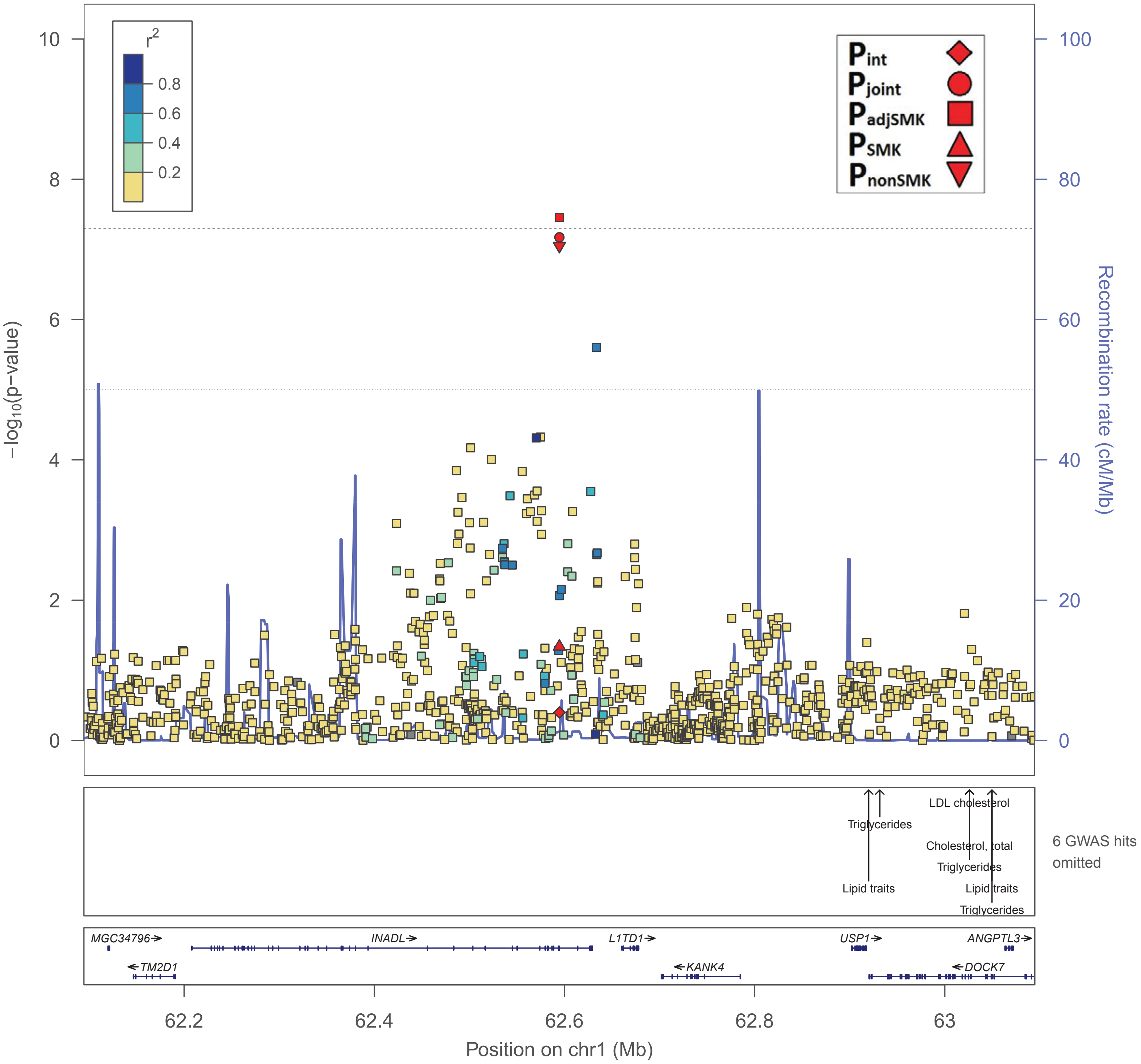

**b.** BMI: rs2173039 − Approach 1, ALL Women

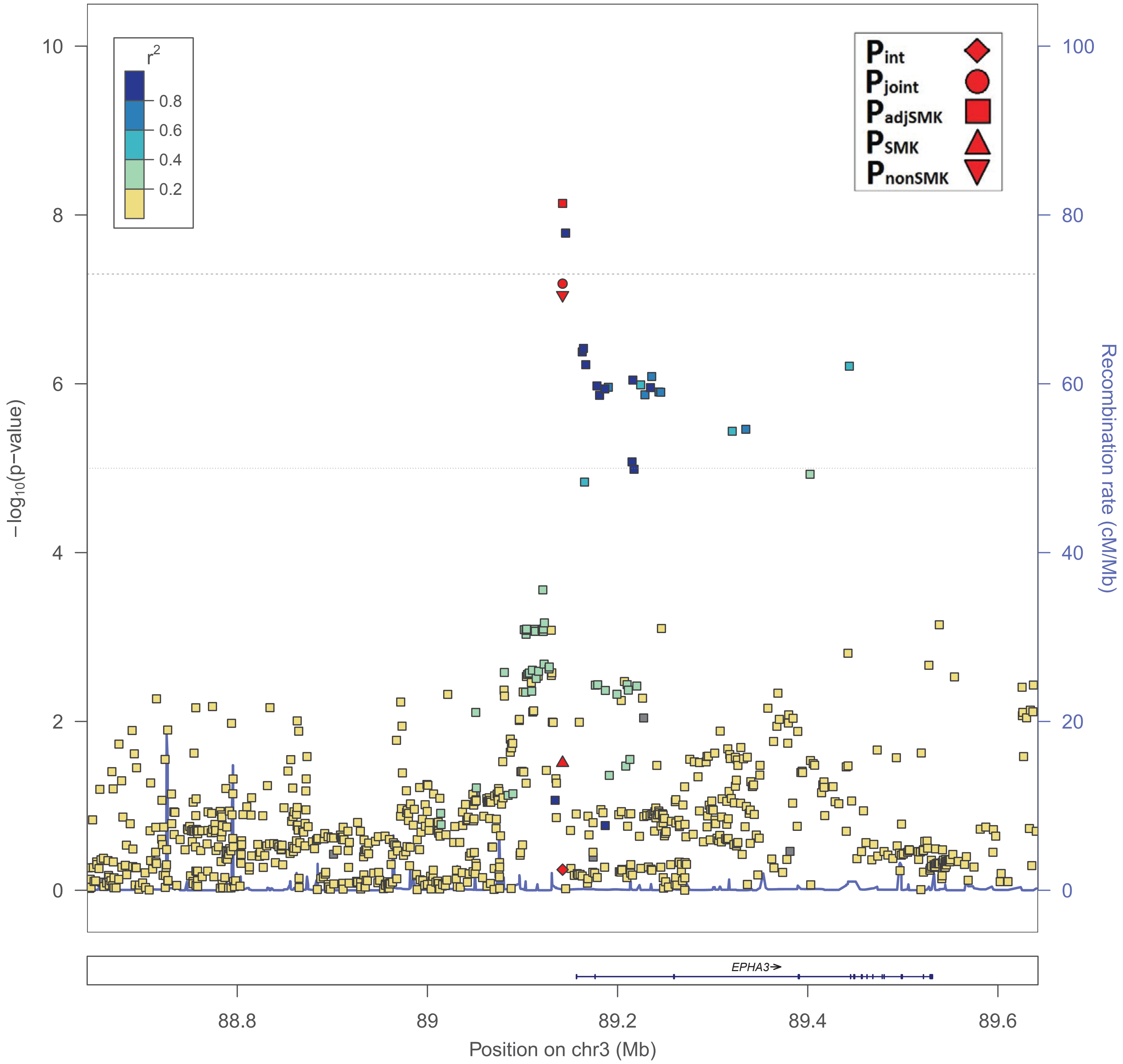

**c.** BMI: rs12629427 − Approach 1, EUR Women

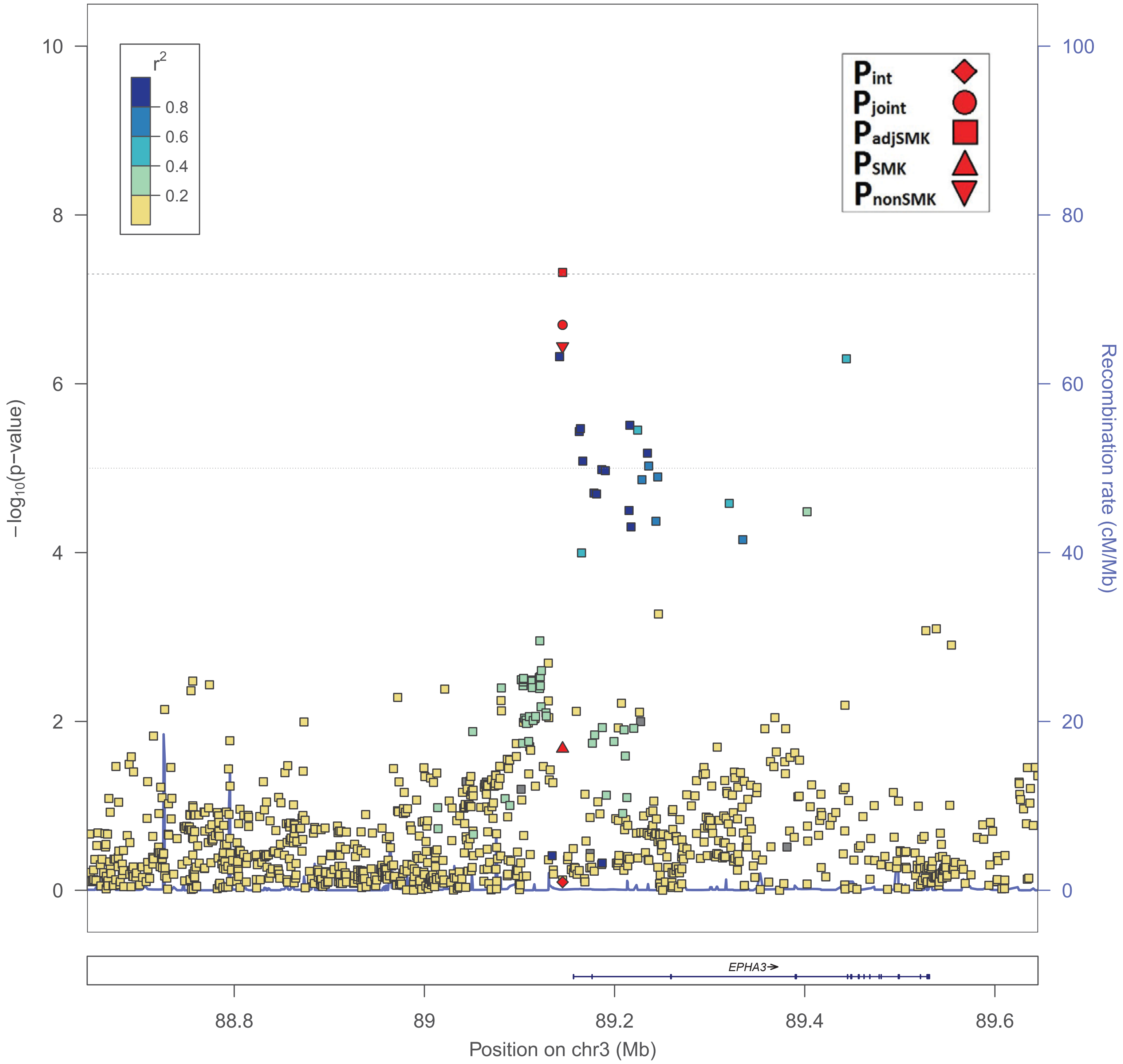

# **d.** WCadjBMI: rs1545348 − Approach 1, EUR Men

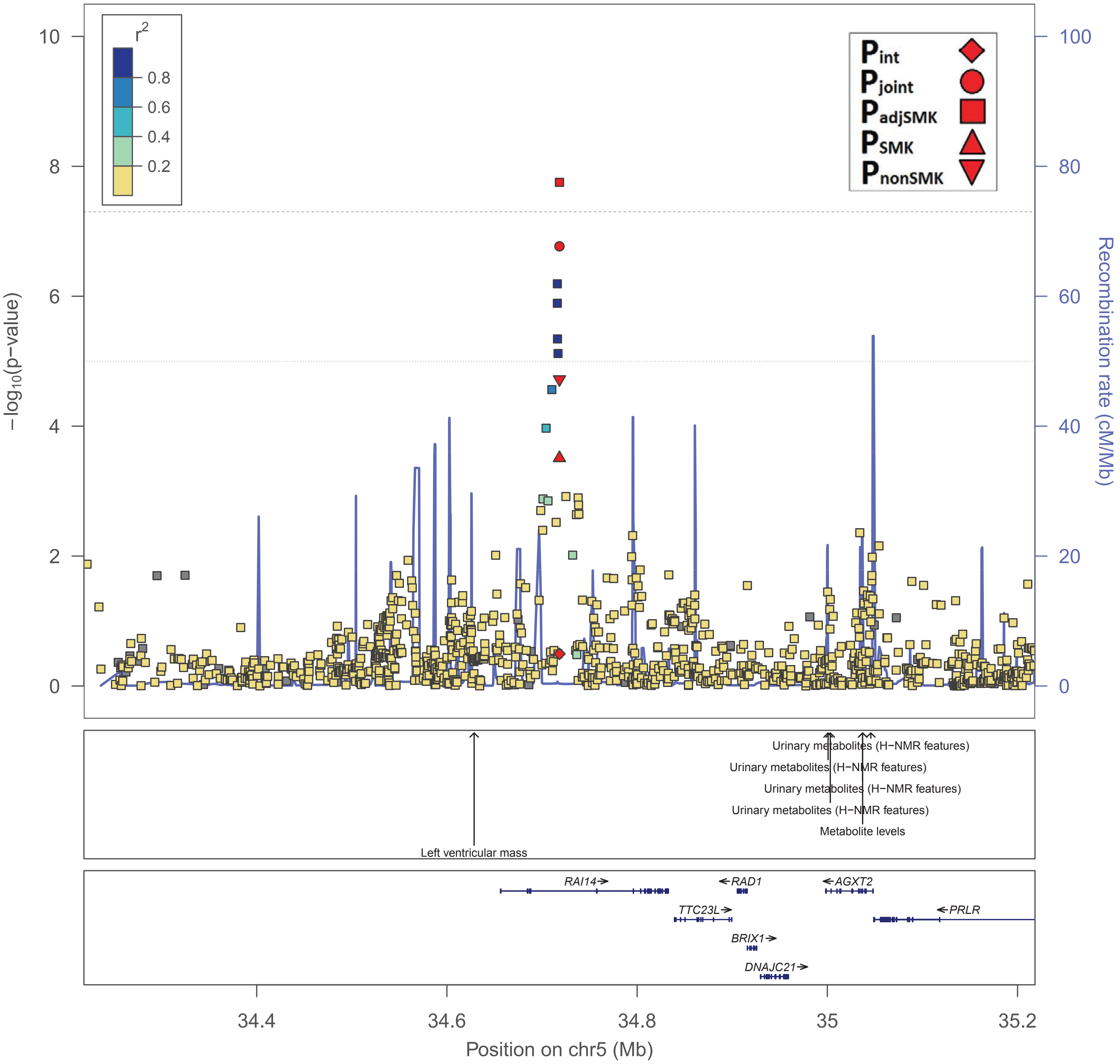

**e.** WCadjBMI: rs6076699 − Approach 2, EUR Women

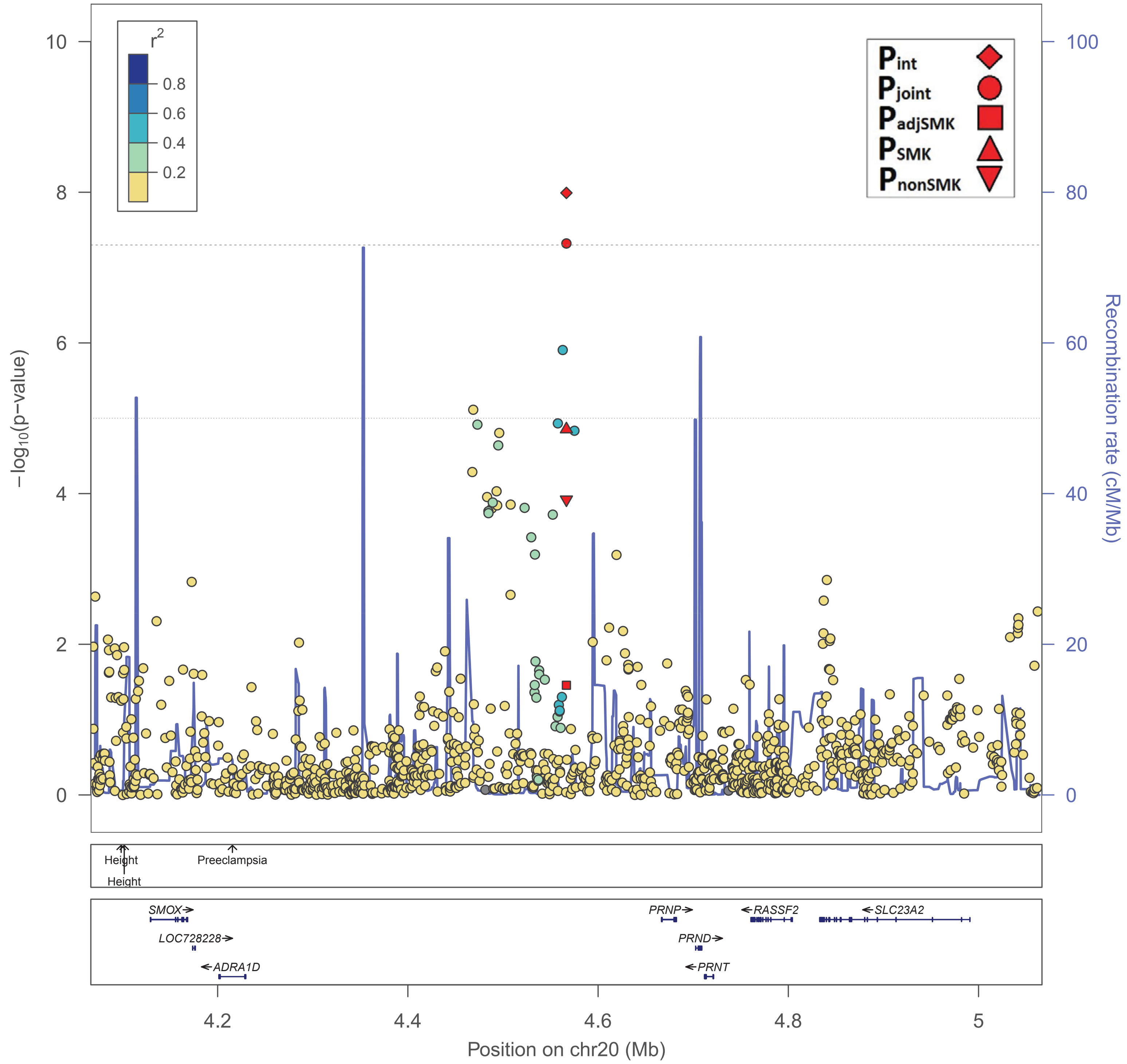

**f.** WCadjBMI: rs6076699 − Approach 3, EUR Women

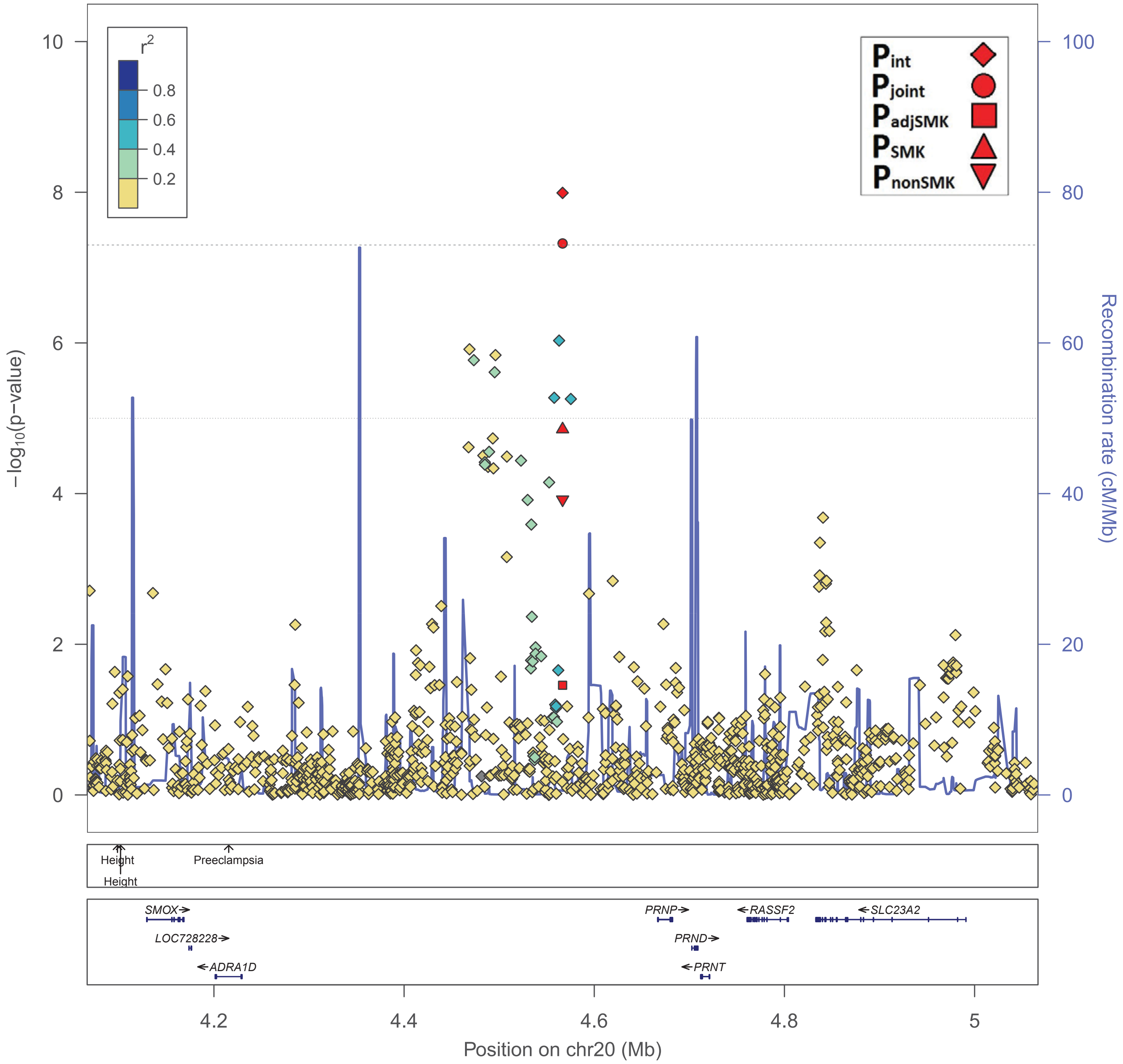

# **g.** WHRadjBMI: rs670752 − Approach 1, ALL Women

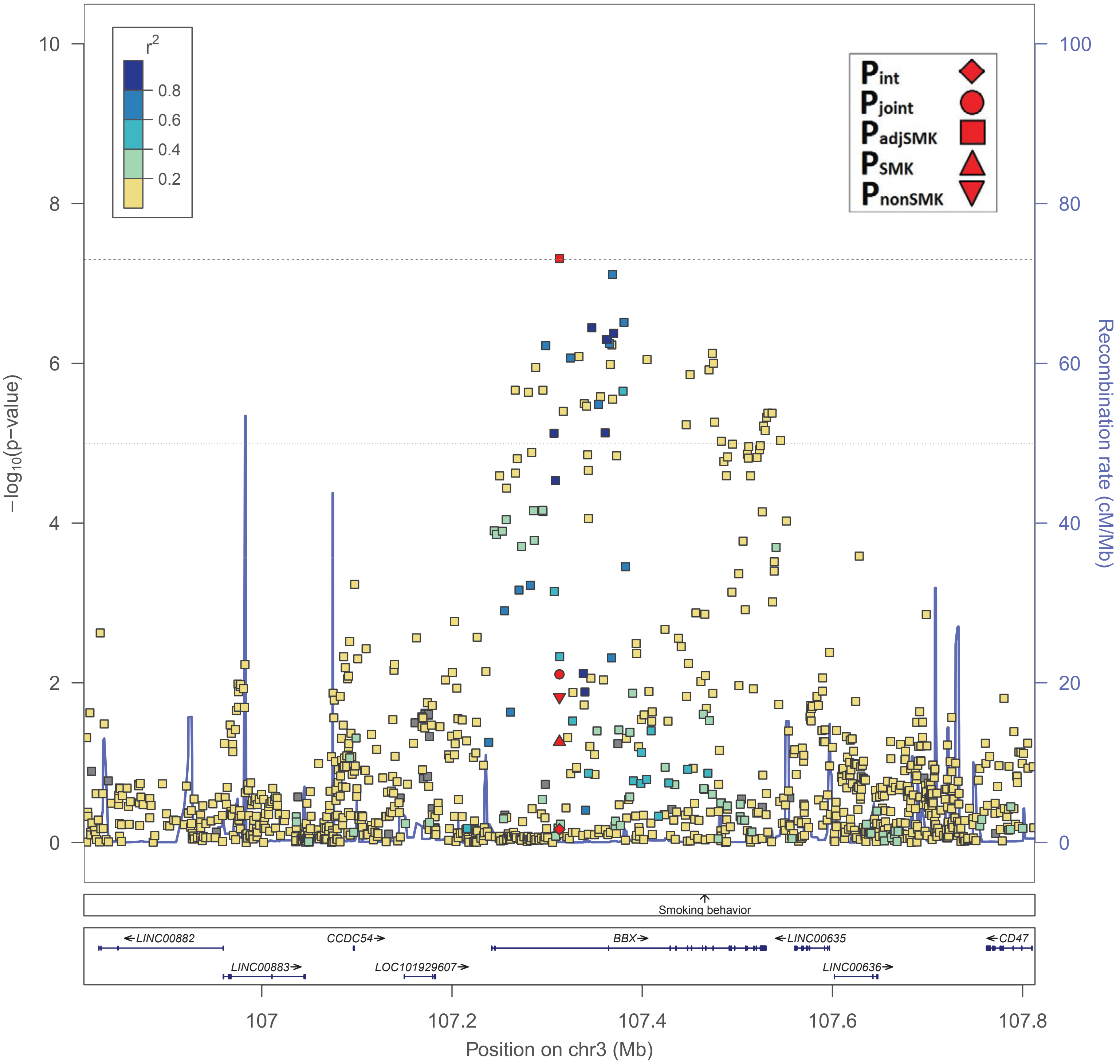

## **h.** WHRadjBMI: rs589428 − Approach 1, EUR Combined Sexes

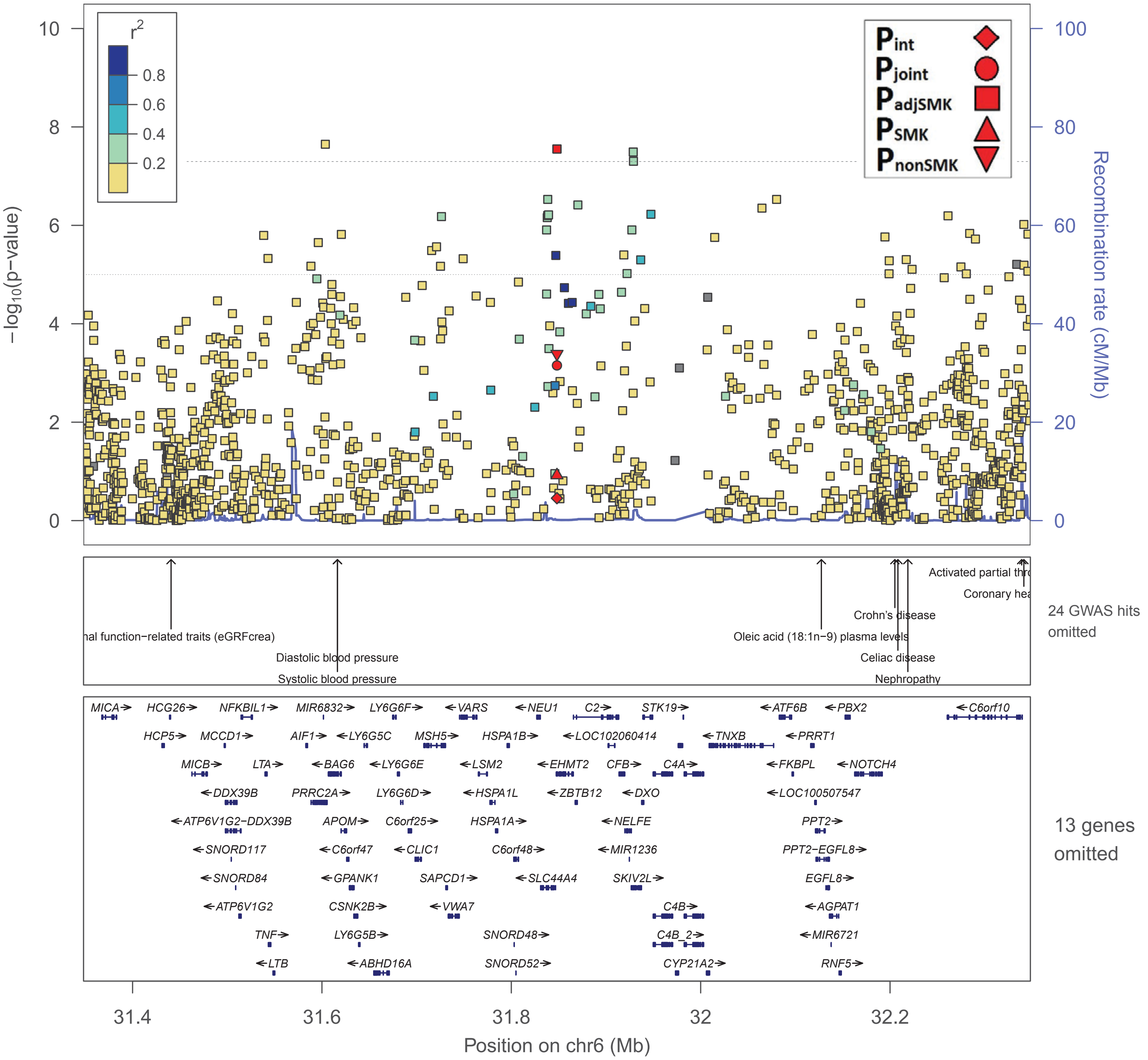

# i. WHRadjBMI: rs1856293 − Approach 2
EUR Combined Sexes

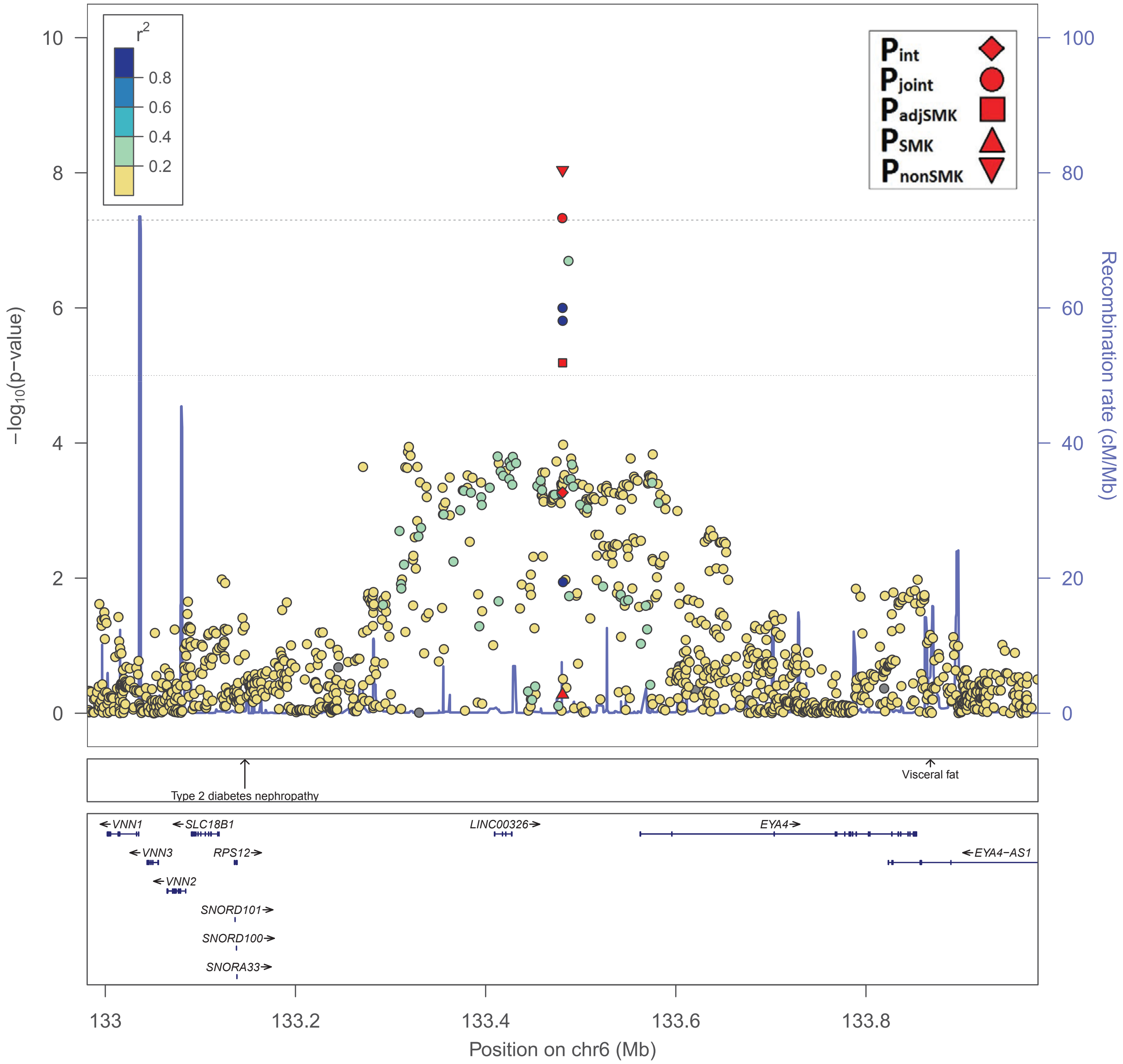

**j.** WHRadjBMI: rs2001945 − Approach 1, ALL Women

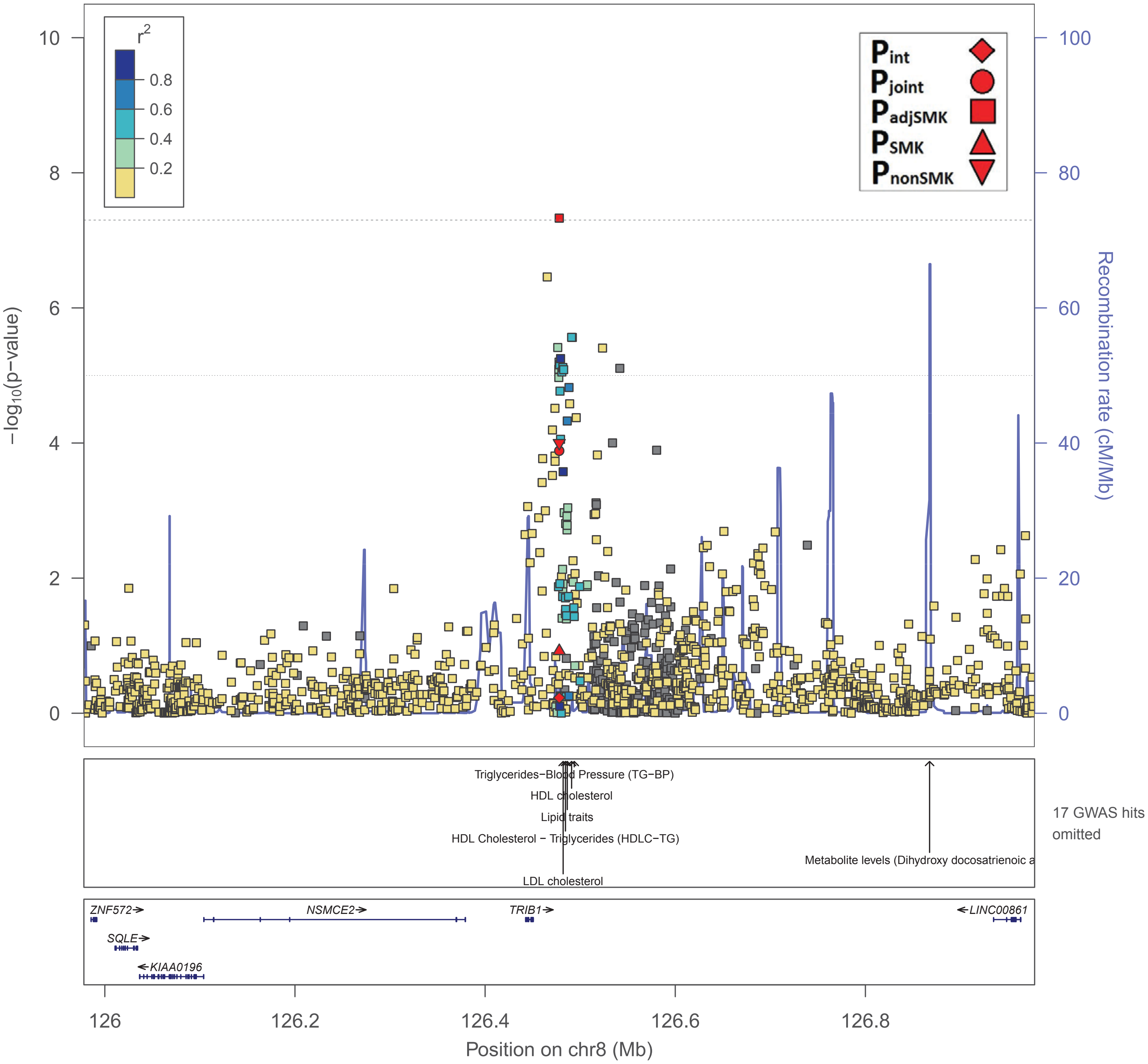

# **k.** WHRadjBMI: rs17065323 – Approach 1, EUR Combined Sexes

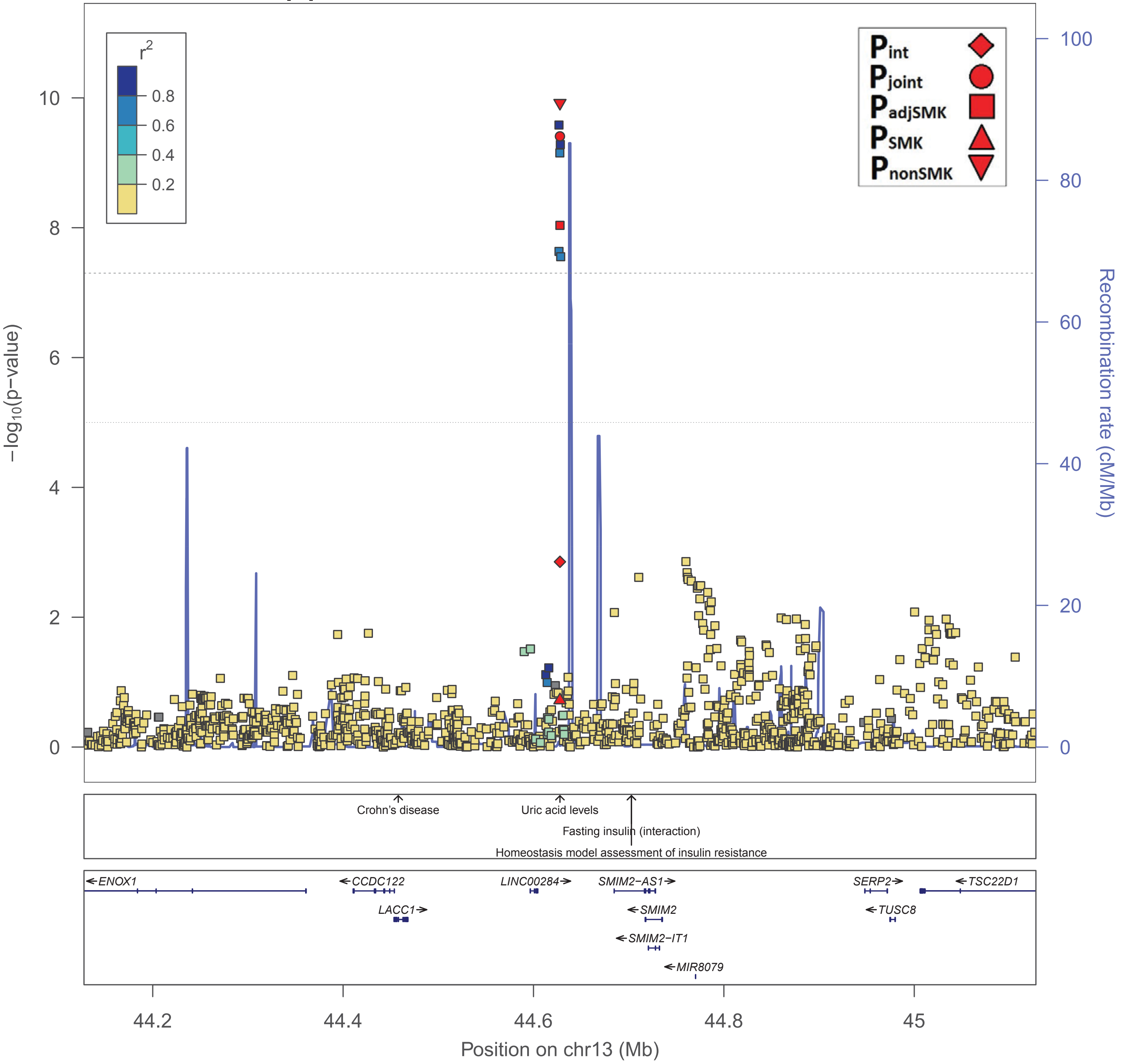

**Supplementary Figure 7. Simulation-based estimation of type 1 error using QQ plots**. Shown are the QQ plots of simulation results for Approach 1 (adjusted effect), Approach 2 (joint effect), Approach 3 and 4 (interaction effects). The simulation was based on MAF=0.05, 50,000 smokers and 180,000 nonsmokers.

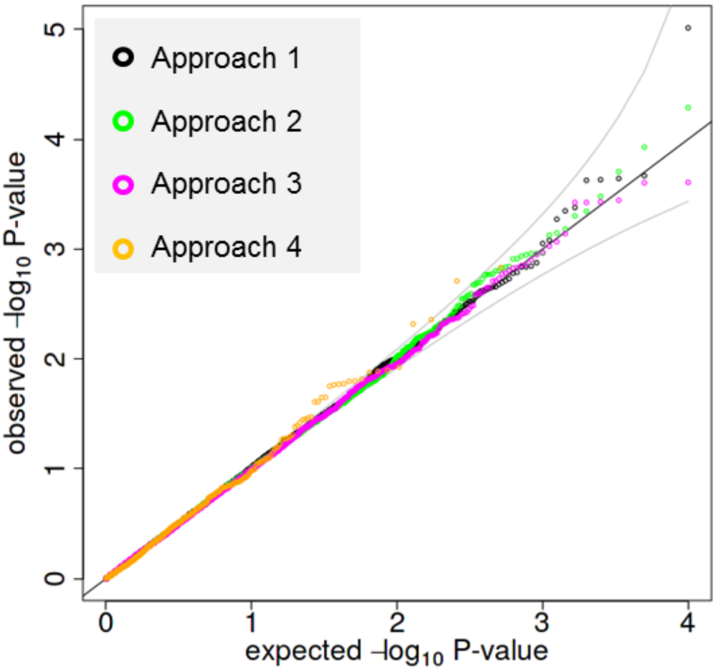

**Supplementary Fig. 8**. Heatmap of –log10P-values for SNPadjSMK, SNPjoint, and SNPint models. We have included each variant identified in the all ancestries analysis which was significant for Approaches 1-3. Strength of color represents the –log10 P-value from the all ancestries, combined sexes meta-analysis for **(a)** BMI in red, **(b)** WCadjBMI in blue, and **(c)** WHRadjBMI in green.

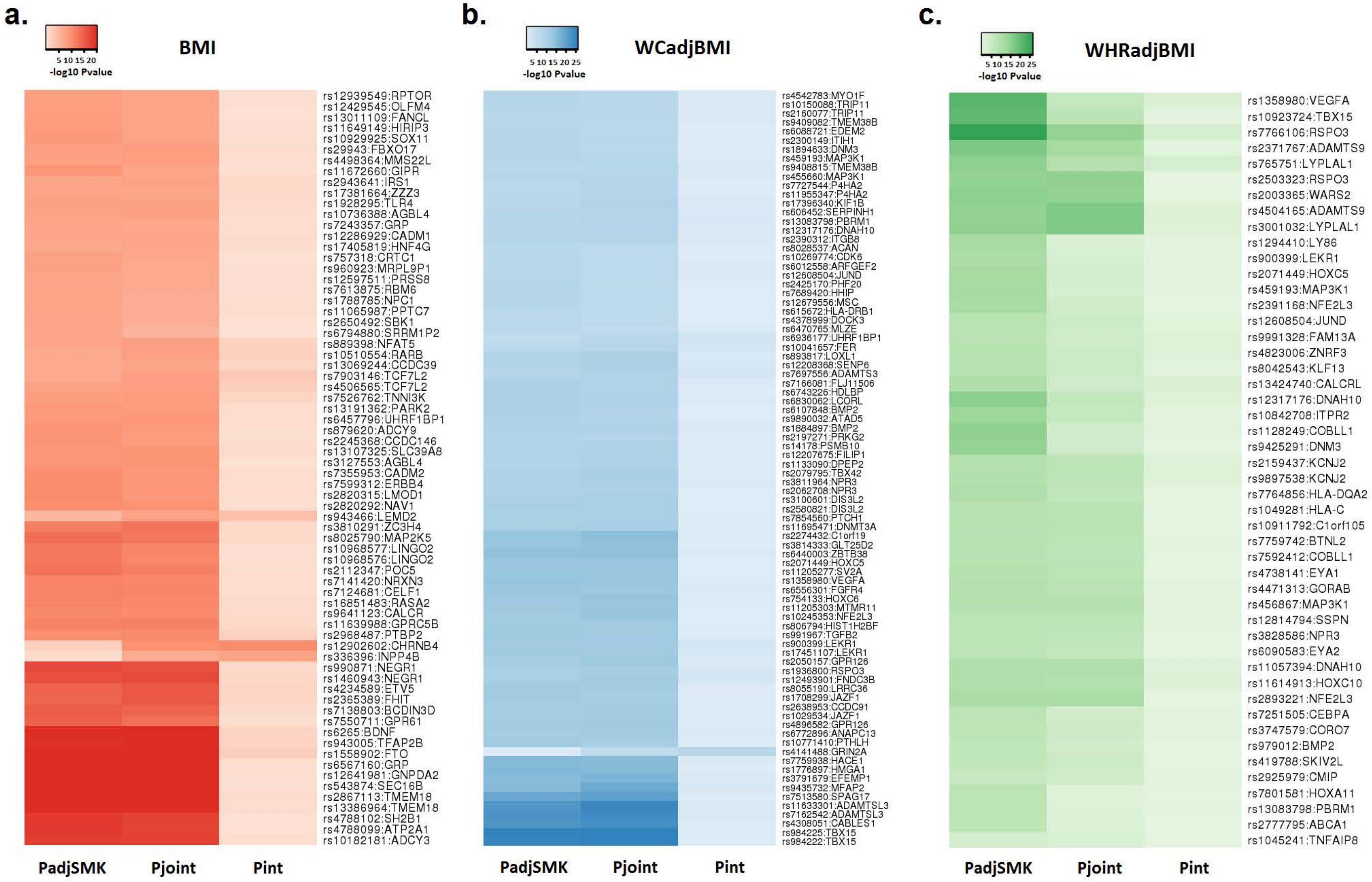

**Supplementary Figure ɸ. Summary plots of discovery meta-analysis for Approach 3 primary meta-analyses**. (**a**) Manhattan plot showing the loci identified in Approach 3 in primary meta-analyses, used to identify significant interaction effects loci (SNPint), in the primary meta-analyses association –log10P-values for BMI-red, WCadjBMI-blue, and WHRadjBMI-green; (**b**) Manhattan plot showing the loci identified in Approach 3 excluding known regions +/- 500 kb and labeled with the nearest gene to the index SNP; (**c**) QQ-plot showing the Approach 3 P-values as observed against those expected under the null for each phenotypes separately (colored); (**d**) QQ-plot for Approach 3 after excluding known association regions.

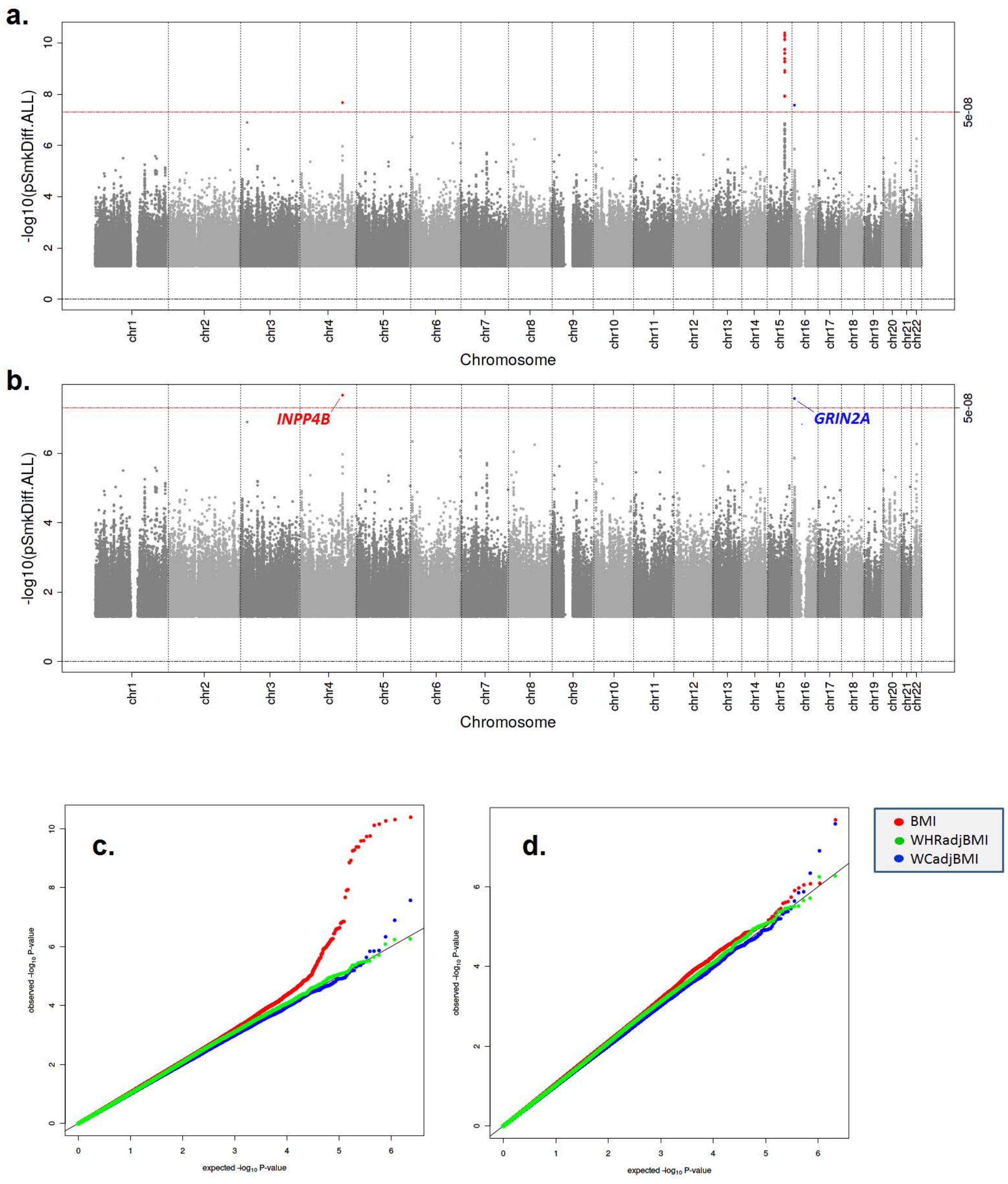

**Supplementary Figure МЛ. Regional association plots for Approach 3 primary meta-analyses.** Regional association plot for all loci identified in Approach 3 in primary meta-analyses, used to identify significant interaction (SNPint), in the primary meta-analyses for BMI: (**a**) rs336396, (**b**) rs12902602; and WCadjBMI: (**c**) rs4141488, and ordered as they appear in Table 3. LD has been calculated using the combined ancestries from the 1000 Genomes Phase 1 reference panel. For comparison, each plot highlights the p-value for the tag SNP in Approach 1 ($P_{adjSMK}$), Approach 2 ($P_{joint}$), Approach 3 ($P_{int}$), current smokers ($P_{SMK}$), and in nonsmokers ($P_{nonSMK}$).

# **a.** BMI: rs336396 − Approach 3

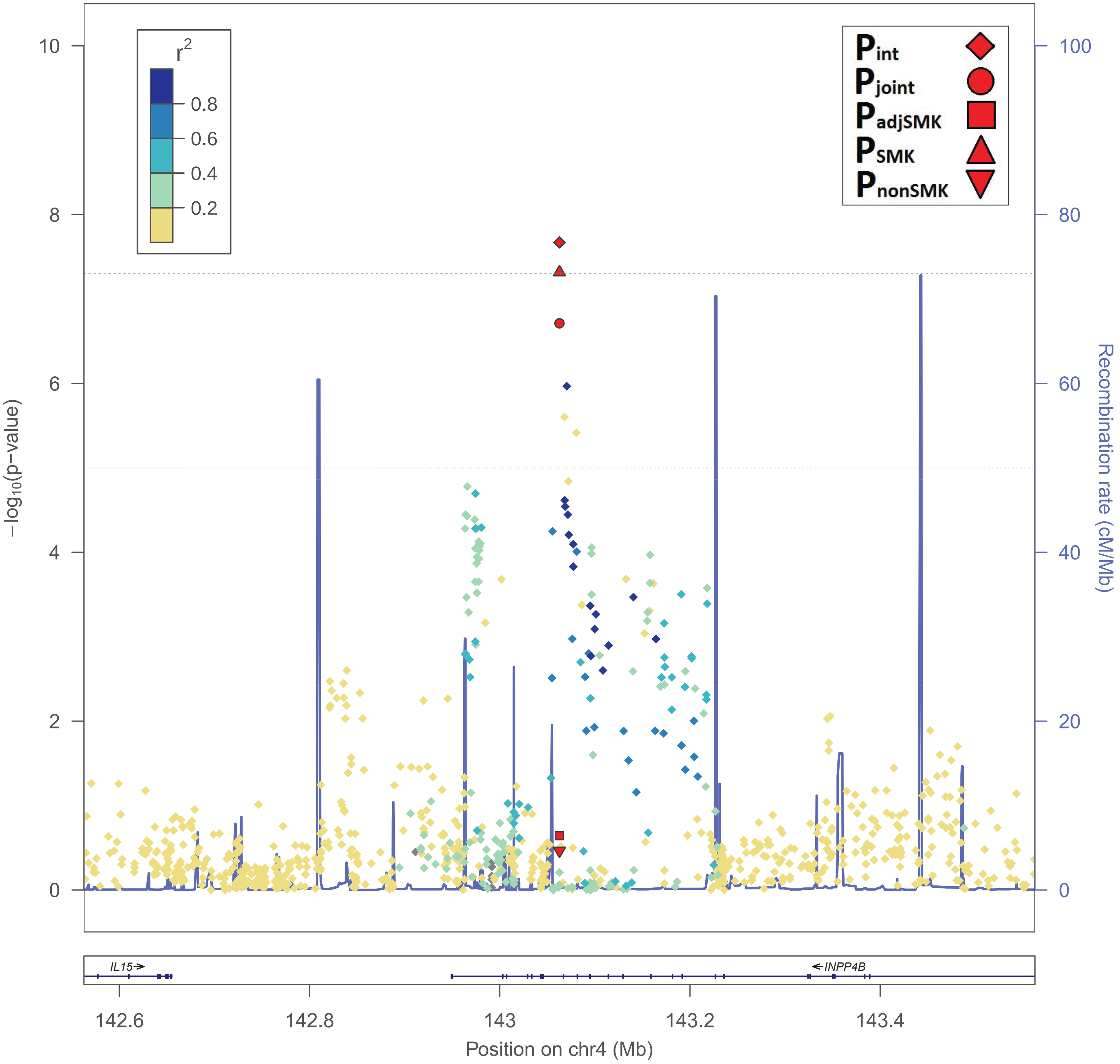

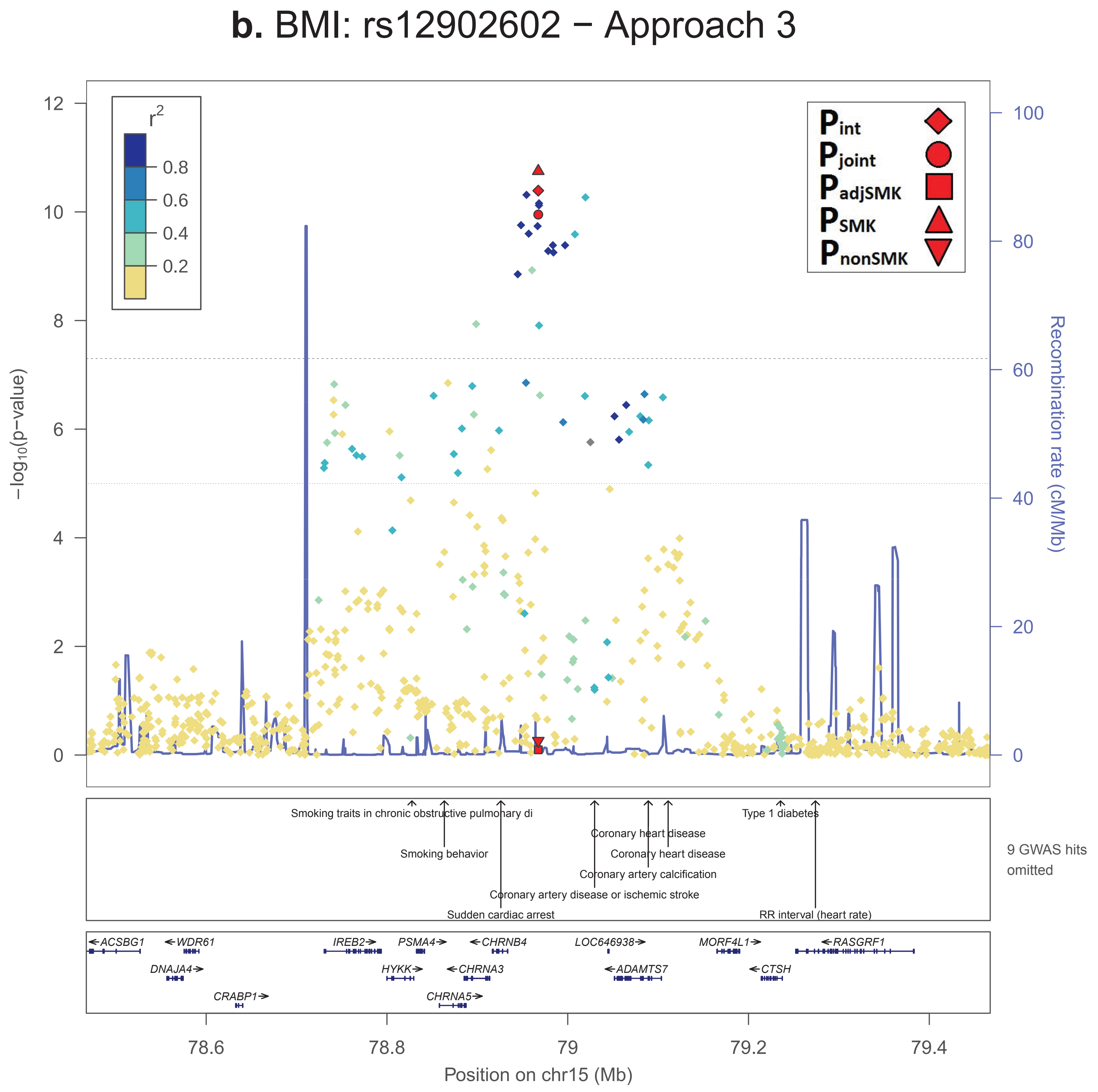
b. BMI: rs12902602 − Approach 3
−log10(p−value)
Recombination rate (cM/Mb)
r2
0.8
0.6
0.4
0.2
Pint
Pjoint
PadjSMK
PSMK
PnonSMK
Smoking traits in chronic obstructive pulmonary di
Smoking behavior
Sudden cardiac arrest
Coronary artery disease or ischemic stroke
Coronary artery calcification
Coronary heart disease
Coronary heart disease
Type 1 diabetes
RR interval (heart rate)
9 GWAS hits omitted
ACSBG1
WDR61
DNAJA4
CRABP1
IREB2
PSMA4
HYKK
CHRNB4
CHRNA3
CHRNA5
LOC646938
ADAMTS7
MORF4L1
CTSH
RASGRF1
78.6
78.8
79
79.2
79.4
Position on chr15 (Mb)

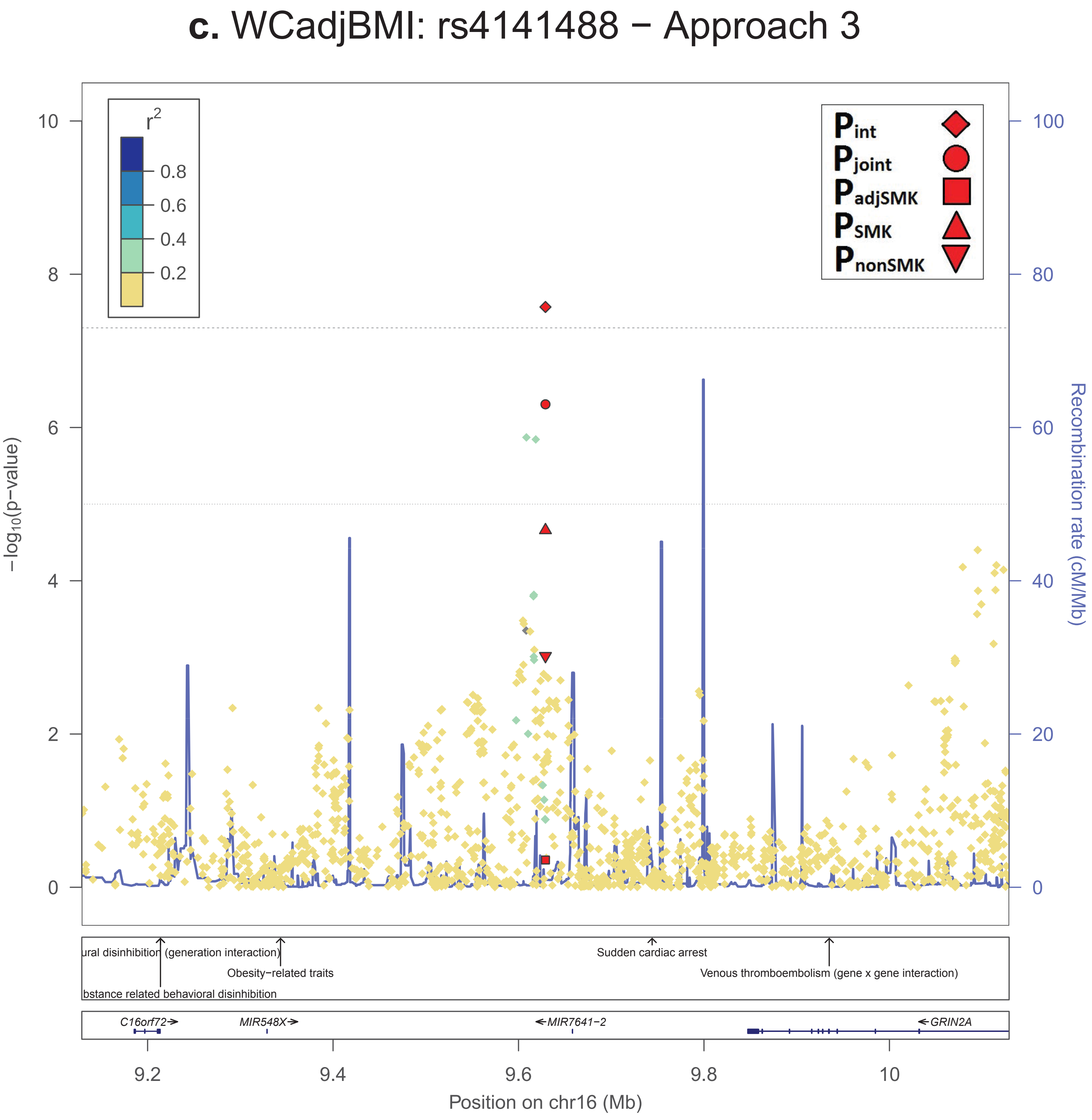
c. WCadjBMI: rs4141488 − Approach 3
r2
0.8
0.6
0.4
0.2
Pint
Pjoint
PadjSMK
PSMK
PnonSMK
−log10(p−value)
Recombination rate (cM/Mb)
ural disinhibition
(generation interaction)
Obesity−related traits
bstance related behavioral disinhibition
Sudden cardiac arrest
Venous thromboembolism (gene x gene interaction)
C16orf72
MIR548X
MIR7641−2
GRIN2A
Position on chr16 (Mb)

**Supplementary Figure MM. Forest plot for significant loci stratified by smoking status.** Estimated effects (β ± 95% CI) for smokers (N upto 51,080) and nonsmokers (N up to 190,178) per risk allele for (**a**) BMI, (**b**) WCadjBMI, and (**c**) WHRadjBMI for the most significant variant for each locus identified in the primary meta-analyses (combined ancestries and combined sexes) for Approaches 1 (SNPadjSMK), 2 (SNPjoint) and 3 (SNPint). Loci are grouped by those with greater effect in nonsmokers, then smokers and then ordered by magnitude of effect in smokers and labeled with the nearest gene.

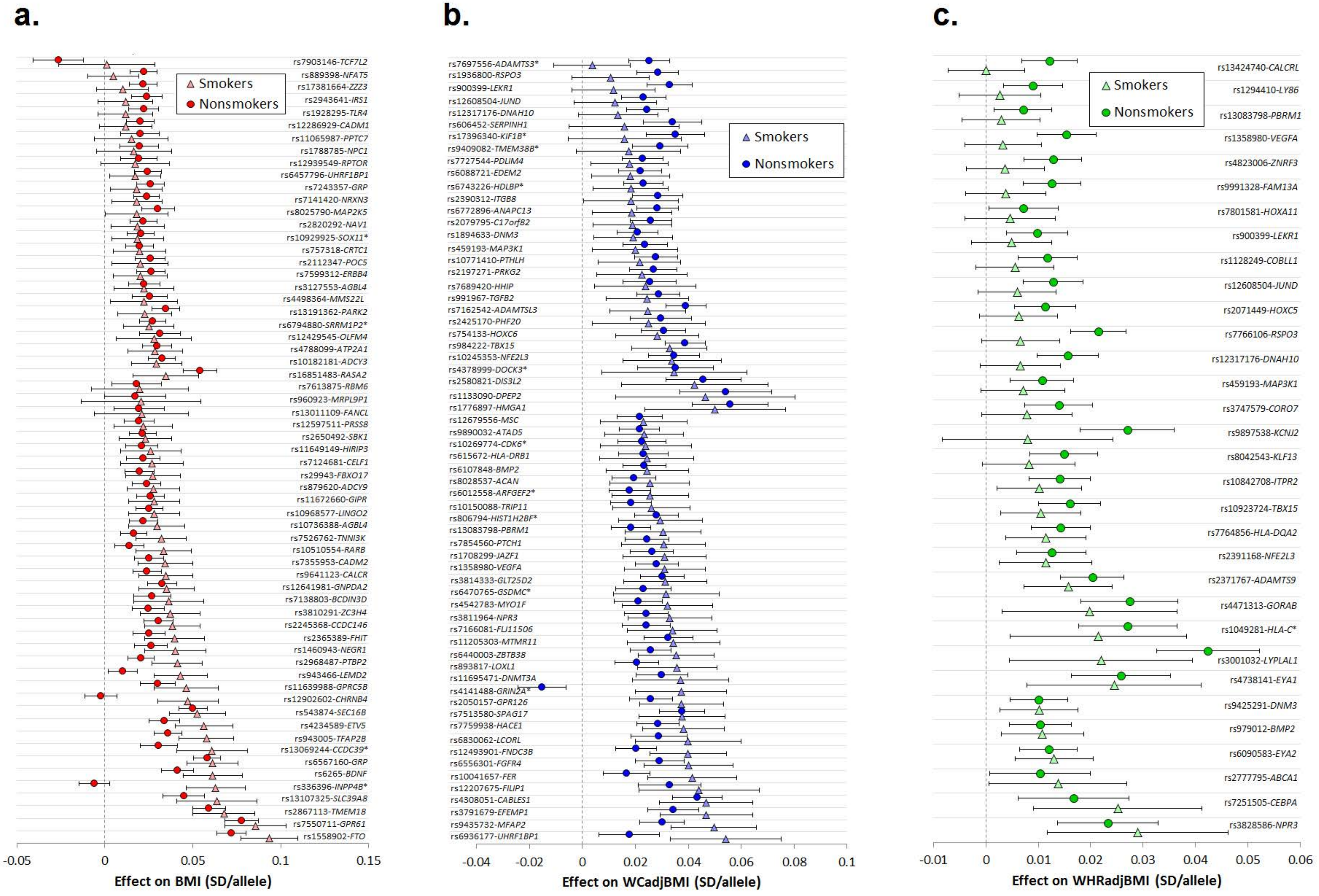

**Supplementary Figure 1H**. Estimated effects (β ± 95% CI) in smokers (N up to 51,080) and nonsmokers (N up to 190,178) per risk allele for (**a**) BMI, (**b**) WCadjBMI, and (**c**) WHRadjBMI for the most significant variant for each locus identified in the secondary meta-analyses (sex-stratified and European-only analyses) for Approaches 1 (SNPadjSMK), 2 (SNPjoint) and 3 (SNPint).

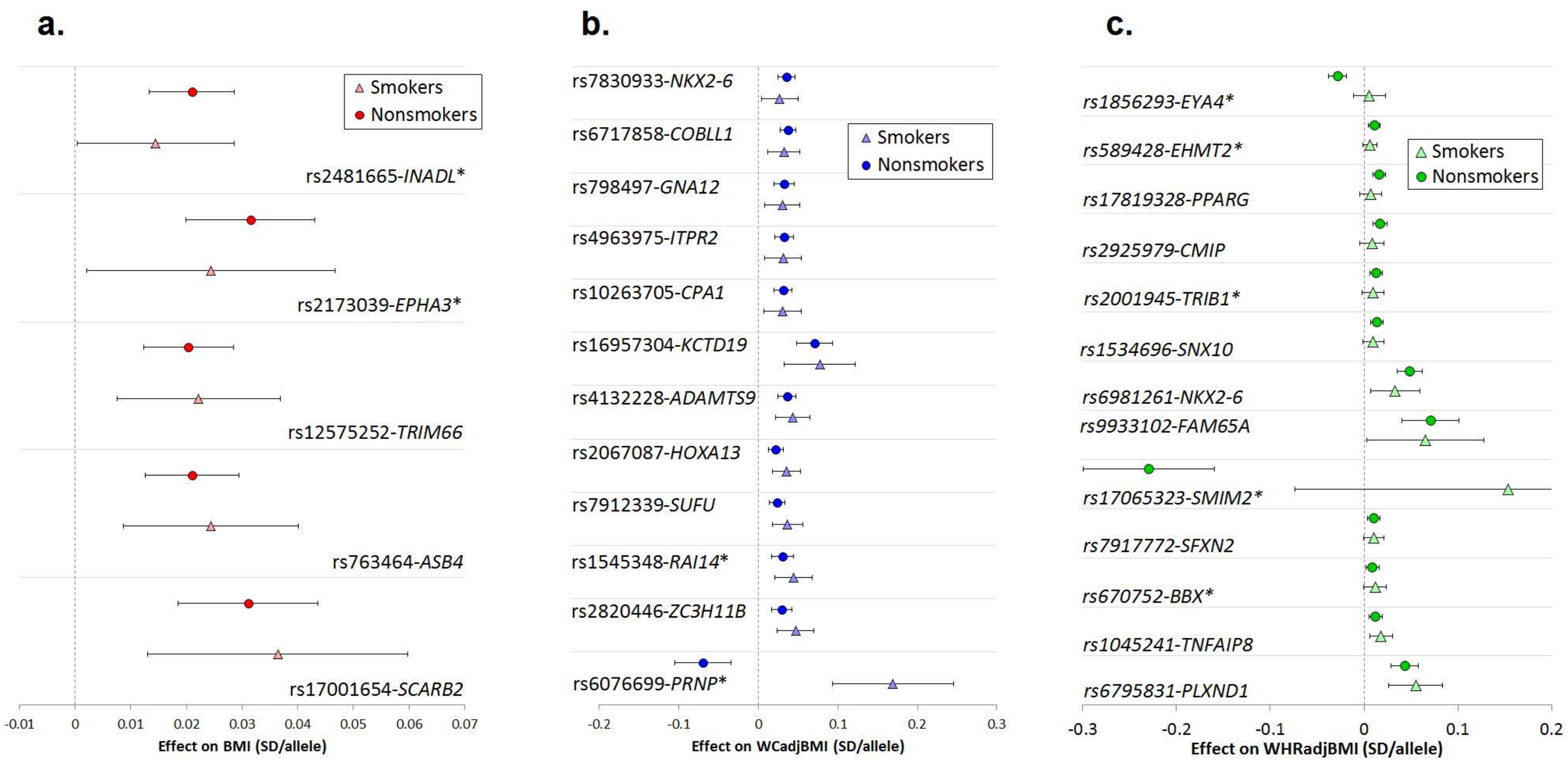

**Supplementary Figure 13**. Comparison of estimated effect estimates (+/-SE) for smokers (GIANT N up to 51,080; UKBB N up to 13,416) and nonsmokers (GIANT N up to 190,178; UKBB N up to 105,218) per risk allele in GIANT only and UKBiobank validation analysis for (**a**) BMI stratified by smoking status, (**b**) BMI adjusted for smoking status, (**c**) WCadjBMI stratified by smoking status, (**d**) WCadjBMI adjusted for smoking status,(**e**) WHRadjBMI stratified by smoking status, and (**f**) WHRadjBMI adjusted for smoking status for each novel and GxSMK SNP in Tables 1-4. All loci are ordered by chromosome and position.

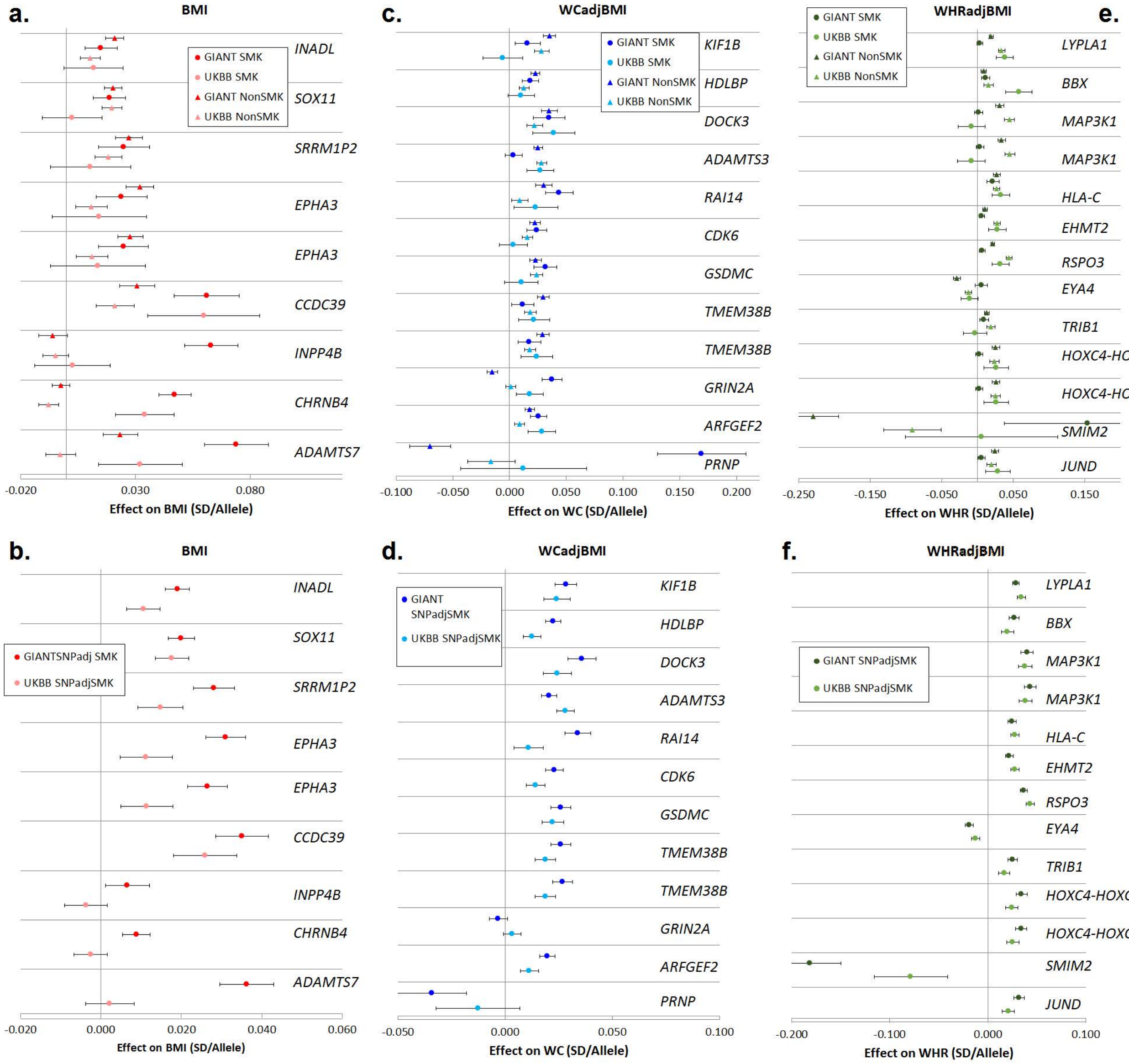

**Supplementary Table 1.** Study design, sample size, and data quality control for contributing genome-wide association study and Metabochip cohorts.

| GWAS DATA | | | | | | | | | | | |
|---|---|---|---|---|---|---|---|---|---|---|---|
| Study | | | | | | Sample QC | | Anthropometric assessment method | | | |
| Short name | Full name | Study design | Ancestry | Region | Total genotyped sample size (N) | Call rate* | sample exclusion criteria | BMI | WHR | WHR and BMI were assessed at the same time? | Study References (PMID) |
| AE | Athero-Express Biobank Study | Cohort-study | European | Utrecht, the Netherlands | 690 | ≥ 97% | 1) heterozygosity (Ĥ) ± 3 standard deviations of the mean<br>2) ethnic outliers through Principal Component Analysis compared to HapMap 2 (r22)<br>3) related individuals and duplicates, π>0.20<br>4) missing body weight and height<br>5) gender-discrepancies | Self-reported | NA | NA | 15678794 |
| AGES | Age, Gene/Environment Susceptibility-Reykjavik Study | Population based | European | Iceland | 3219 | >99% | 1) missing phenotypes<br>2) mismatch previous genotypes | Measured | NA | NA | 17351290 |
| ARIC | Atherosclerosis Risk in Communities | Population-based | European | USA-North America | 9713 | >90% | 1) first-degree relatives<br>2) ancestry outliers<br>3) gender mismatch<br>4) identity issues<br>5) excessive heterozygosity<br>6) missing height or weight | Measured | Measured | Yes | 2646917 |
| ARIC | Atherosclerosis Risk in Communities | Population-based | African American | USA-North America | 3207 | >90% | 1) first-degree relatives<br>2) ancestry outliers<br>3) gender mismatch<br>4) identity issues<br>5) excessive heterozygosity<br>6) missing height or weight | Measured | Measured | Yes | 2646917 |
| AUSTWIN | Australian Twin-Family Studies | Population-based | European | Australia | 2166 | 95% | 1) mendelian errors<br>2) HWE p < 10-6<br>3) sample call rate  96% | Self-reported | NA | NA | 21529783 |
| BHS | Busselton Health Study | Population-based | European | Australia | 1468 | 97% | 1) duplicates<br>2) sex check<br>3) ethnic outliers | Measured | Measured | Yes | 15486340, 19643935 |
| BioMe (MSSM) | The Charles Bronfman Institute for Personalized Medicine; BioMe Program | EHR-linked clinical care cohort | European American | USA (New York City) | 2026 | ≥98% | 1) sex mismatch<br>2) ancestry Outliers<br>3) related Individuals (inbreeding coefficient < -0.1 or > 0.3 for common variants (MAF>1%)<br>4) inbreeding coefficient < 0.4 or > 0.9 for rare variants (MAF<1%) | Self-reported | NA | NA | 25673413 |
| BioMe (MSSM) | The Charles Bronfman Institute for Personalized Medicine; BioMe Program | EHR-linked clinical care cohort | African American | USA (New York City) | 3495 | ≥98% | 1) sex mismatch<br>2) ancestry Outliers<br>3) related Individuals (inbreeding coefficient < -0.1 or > 0.3 for common variants (MAF>1%)<br>4) inbreeding coefficient < 0.4 or > 0.9 for rare variants (MAF<1%) | Self-reported | NA | NA | 25673413 |

| | | | | | | | | | | | |
|---|---|---|---|---|---|---|---|---|---|---|---|
| BioMe (MSSM) | The Charles Bronfman Institute for Personalized Medicine; BioMe Program | EHR-linked clinical care cohort | Hispanic / Latinos | USA (New York City) | 4711 | ≥98% | 1) sex mismatch<br>2) ancestry Outliers<br>3) related Individuals (inbreeding coefficient < -0.1 or > 0.3 for common variants (MAF>1%)<br>4) inbreeding coefficient < 0.4 or > 0.9 for rare variants (MAF<1%) | Self-reported | NA | NA | 25673413 |
| BLSA | Baltimore Longitudinal Study of Aging | Population-based | European | Italy | 1230 | 98% | 1) Missing Phenotype<br>2) sex mismatch<br>3) ethnic outliers | measured | measured | Yes | NA |
| British 1958 birth cohort (B58C) | British 1958 birth cohort | Population-based | European | UK | 6481 | ≥ 97% | 1) Contamination<br>2) non-European identity<br>3) missing height or weight | Measured | Measured | Yes | 17255346 |
| CHS | The Cardiovascular Health Study | Population-based cohort | European | USA-North America | 3271 | >95% | 1) baseline CHD, CHF, PAD, valvular heart disease, stroke, TIA<br>2) lack of available DNA<br>3) genotype discordant with known sex or prior genotyping | Measured | Measured | Yes | 1669507 |
| CLHNS | Cebu Longitudinal Health and Nutrition Survey (Offspring) | Population-based | East Asian (Filipino) | Philippines | 1895 | ≥97% | 1) 1st-degree relatvies<br>2) missing phenotypes (age, weight, height, smoking) | Measured | Measured | Yes | 20507864 |
| COLAUS | Cohorte Lausannoise | Population-based | European | Switzerland | 5435 | 90% | 1) PCA outliers | Measured | Measured | Yes | 22415877, 18366642 |
| CROATIA-Korcula | The CROATIA study, Korcula Island cohort | Population-based | European | Croatia | 898 | >97% | 1) duplicates<br>2) gender mismatch<br>3) identity issues<br>4) excess heterozygousity | Measured | Measured | Yes | 18952825<br>19798445 |
| CROATIA-Vis | The CROATIA study, Vis Island cohort | Population-based | European | Croatia | 924 | >97% | 1) duplicates<br>2) gender mismatch<br>3) identity issues<br>4) excess heterozygousity | Measured | Measured | Yes | 18952825, 19798445 |
| DESIR | Data from an Epidemiological Study on the Insulin Resistance syndrome | Case-control | European | France | 731 | ≥90% | 1) low call rate (none)<br>2) ethnic outliers (n=15 dropped using STRUCTURE) | Measured | Measured | Yes | 8927780 |
| EGCUT | Estonian Genome Center, University of Tartu | Population-based | European | Estonia | 2059 | ≥98% | 1) call-rate < 95%<br>2) related smaller than 2nd degree<br>3) duplicated samples<br>4) gender mismatch | Measured | Measured | Yes | 24518929 |
| EPIC-Norfolk | EPIC (European Prospective Investigation into Cancer) Norfolk | Population-based | European | England | 2417 | ≥94% | 1) relatedness<br>2) ethnic outlier<br>3) missing height | Measured | Measured | Yes | 10466767 |
| ERF | Erasmus Rucphen Family Study | Family-based | European | Netherlands | 3,485 | 95% | 1) gender mismatch<br>2) ethnic outliers<br>3) sex mismatch<br>4) excess heterozygosity<br>5) duplicates | Measured | Measured | Yes | 15845033 |

| | | | | | | | | | | | |
|---|---|---|---|---|---|---|---|---|---|---|---|
| FamHS | Family Heart Study | Family-based | European | USA - North America | 3869 | 98% | 1) To assess Mendelian errors, we ran LOKI on our family data and removed 5,035 SNPs with enough Mendelian errors to be considered outlier SNPs. We also removed 2 individuals who had an unacceptable number (greater than 600) of Mendelian errors, making them outliers as compared to the rest of | Measured | Measured | Yes | 8651220 |
| Fenland | Fenland Study | Population-based | European | UK | 1500 | 95% | 1) sex-check<br>2) relatedness check<br>3) ethnic ancestry outlying<br>4) hetezygosity check | Measured | Measured | Yes | 20519560 |
| FramHS | Framingham Heart Study | Population-based | European | USA | 8481 | >97% | 1) Heterozygosity ± 5 SD from the mean<br>2) Call rate < 97%<br>3) Excessive Mendelian errors | Measured | Measured | Yes | 14025561<br>1208363<br>17372189 |
| FUSION | Finland-United States Investigation of NIDDM Genetics | Case-control | European | Finland | 2333 | >99% | 1) Missing phenotypes or age<18<br>2) Gender discrepancy or anomaly<br>3) Unexplained duplicate sample<br>4) Relateds | Measured | Measured | Yes | 17463248 |
| Gendian | Genetics of Diabetic Nephropathy | Cohort study of type 2 diabetes complications | European | Germany | 1026 | 95% | 1) call-rate < 95%<br>2) related smaller than 2nd degree<br>3) duplicated samples<br>4) gender mismatch<br>5) non-european ethnicity | Measured | Measured | Yes | 16961167<br>21980298 |
| GS | Generation Scotland | Population-based | European | Scotland | 9860 | >97% | 1) duplicates<br>2) gender mismatch<br>3) identity issues<br>4) excess heterozygousity | Measured | Measured | Yes | 22786799 |
| GENOA | Genetic Epidemiology Network of Arteriopathy | Cohort of sibships enriched for hypertension | European-American | USA- Rochester, MN | 1509 | ≥ 95% | 1) Identical twins<br>2) sex mismatch<br>3) outliers (±6 SDs) on first 10 PCs from EIGENSTRAT | Measured | Measured | Yes | 11799070,<br>15121494 |
| GOOD | Gothenburg Osteoporosis and Obesity Determinants Study | Population-based | European | Sweden | 941 | >97% | 1) heterozygosity > 33%<br>2) ethnic outliers<br>3) related individuals<br>4) duplicates | Measured | Measured | Yes | 22247082 |
| GOYA cases | Genetics of Overweight Young Adults | Population-based (case-cohort) | European | Denmark | 671 cases, | 95% | 1) heterozygosity<br>2) duplicates<br>3) sex mismatch<br>4) non-caucasians | Measured | Measured | Yes | 18445669,<br>21935397 |
| GOYA controls | Genetics of Overweight Young Adults | Population-based (case-cohort) | European | Denmark | 790 controls | 95% | 1) heterozygosity<br>2) duplicates<br>3) sex mismatch<br>4) non-caucasians | Measured | Measured | Yes | 18445669,<br>21935397 |
| HERITAGE Family Study | Health, Risk Factors, Training and Genetics (HERITAGE) Family Study | Baseline measurements of an exercise intervention | European | USA -North America | 499 | >99% | None | Measured | Measured | Yes | 21183627 |

| | | | | | | | | | | | |
|---|---|---|---|---|---|---|---|---|---|---|---|
| HRS | Health and Retirement Study | Population-based | European-American | USA-North America | 8652 | >98% | 1) duplicates<br>2) gender discrepancy<br>3) relatedness<br>4) ethnic outliers | Measured | NA | NA | 24671021 |
| HRS | Health and Retirement Study | Population-based | African American | USA-North America | 1519 | >98% | 1) duplicates<br>2) gender discrepancy<br>3) relatedness<br>4) ethnic outliers | Measured | NA | NA | 24671021 |
| HYPERGENES | HYPERGENES | Case-Control | European | Europe | 3916 | >95% | 1) first-degree and second-degree relatives<br>2) ancestry outliers<br>3) gender mismatch<br>4) identity issues<br>5) excessive heterozygosity<br>6) missing height or weight | Measured | Measured | Yes | 22184326 |
| InCHIANTI | Invecchiare in Chianti | Population-based | European | Italy | 1231 | 97% | 1) missing Phenotype 2) sex mismatch 3) Heterozygosity > 0.3 | Measured | Measured | Yes | 11129752 |
| KORA3 | Cooperative Health Research in the Region of Augsburg | Population-based | European | Germany | 1,644 | 93% | 1) german passport<br>2) missing height | Measured | Measured | Yes | 16032513, 16032514 |
| KORA4 | Cooperative Health Research in the Region of Augsburg | Population-based | European | Germany | 1,814 | 93% | 1) german passport<br>2) missing height | Measured | Measured | Yes | 16032513, 16032514 |
| Lifelines | Lifelines Cohort study | Population-based | European | The Netherlands | 9,388 | 95% | 1) heterozygosity > 4SD from mean<br>2) duplicate and MZ samples<br>3) first-degree relatives<br>4) sex mismatch<br>5) non-caucasians | Measured | Measured | Yes | 18075776, 25502107, 26333164 |
| LOLIPOP_EW610 | London Life Sciences Prospective Population Study | Population-based | European | UK - Lodon | 945 | 95% | 1) duplicates<br>2) gender discrepancy<br>3) contaminated samples<br>4) relatedness | Measured | Measured | Yes | 21909110 |
| LOLIPOP_IA317 | London Life Sciences Prospective Population Study | Population-based with some enrichment | Indian Asian | UK - Lodon | 2694 | 95% | 1) duplicates<br>2) gender discrepancy<br>3) ethnic outliers<br>4) contaminated samples<br>5) relatedness<br>6) samples already in IA610 | Measured | Measured | Yes | 18454146 |
| LOLIPOP_IA610 | London Life Sciences Prospective Population Study | CHD case-control study | Indian Asian | UK - Lodon | 7032 | 95% | 1) duplicates<br>2) gender discrepancy<br>3) ethnic outliers<br>4) contaminated samples<br>5) relatedness | Measured | Measured | Yes | 19820698 |
| LURIC | Ludwigshafen Risk and Cardiovascular Health Study | Case-control | European | Ludwigshafen, Germany | 2984 | ≥95% | 1) related individuals<br>2) ambigous sex | Measured | Measured | Yes | 11258203 |
| MESA | Multi-Ethnic Study of Atherosclerosis | Population-based | European | USA-North America | 2399 | >95% | 1) first-degree relatives<br>2) ancestry outliers<br>3) gender mismatch,<br>4) excessive heterozygosity<br>5) missing height or weight | Measured | Measured | Yes | 19075250 |

| | | | | | | | | | | | |
|---|---|---|---|---|---|---|---|---|---|---|---|
| MrOS Sweden | Osteoporotic Fractures in Men (MrOS) Sweden | Population-based | European | Sweden | 962 | >97% | 1) exclusion based on IBD clustering<br>2) checked for duplicates<br>3) identical twins | Measured | NA | NA | 16598372 |
| NTR | Netherlands Twin Register | Population-based | European | Netherlands | 3331 | >90% | 1) duplicates<br>2) gender discrepancy<br>3) contaminated samples<br>4) relatedness | Measured | Measured | Yes | 18763692, 18197199 |
| NFBC66 | Northern Finland Birth Cohorts 1966 | Population-based | European | Finland | 5402 | ≥95% | (1) duplicates<br>(2) contaminated samples<br>(3) excess heterozygosity<br>(4) cryptic relatedness<br>(5) withdrew consent (6) gender mismatch<br>(7) MDS outliers | Measured | Measured | Yes | 4911003 |
| NHS | The Nurses' Health Study | Population-based | European | USA | 2368 | 95% | 1) duplicate<br>2) samples with misidentified sex,<br>3) related samples (siblings or possible first cousins)<br>4) samples with evidence of contamination<br>5) samples with highly variable intensity data, and | Measured | Measured | Yes | 7860180 |
| ORCADES | Orkney Complex Disease Study | Population-based | European | Scotland | 889 | >97% | 1) duplicates<br>2) gender mismatch<br>3) identity issues<br>4) excess heterozygousity | Measured | Measured | Yes | 18760389 |
| PIVUS | The Prospective Investigation of the Vasculature in Uppsala Seniors | Prospective cohort | European | Sweden | 949 | >95% | 1) gender mismatch<br>2) excess heterozygosity<br>3) duplicates | Measured | Measured | Yes | 16141402 |
| Prevend | Prevention of renal and vascular end-stage disease | Population-based | European | The Netherlands | 3,649 | ≥95% | 1) ethnic outliers<br>2) related individuals and duplicates<br>3) missing phenotype | Measured | Measured | Yes | 12356629 |
| PROSPER | The PROspective study of Pravastatin in the Elderly at Risk for vascular disease | Population-based | European | Europe | 5244 | ≥95% | 1) sex mismatch<br>2) ethnic outliers<br>3) heterozygosity (3 SD) | Measured | NA | NA | 10569329 |
| QFS | Quebec Family Study | Population-based | European | Quebec-Canada | 929 | 95% | None | Measured | Measured | Yes | 24533236 |
| Rotterdam Study I | Rotterdam Study - I | Population-based | European | Netherlands | 5974 | ≥ 98% | 1) sex mismatch<br>2)excess autosomal heterozygosity >0.336 3) outliers identified by IBS clustering analysis | Measured | Measured | Yes | 26386597 |
| Rotterdam Study II | Rotterdam Study - II | Population-based | European | Netherlands | 2157 | ≥98% | 1) sex mismatch<br>2)excess autosomal heterozygosity >0.336<br>3) outliers identified by IBS clustering analysis | Measured | Measured | Yes | 26386597 |
| Rotterdam Study III | Rotterdam Study - III | Population-based | European | Netherlands | 2078 | ≥ 98% | 1) sex mismatch<br>2)excess autosomal heterozygosity >0.336<br>3) outliers identified by IBS clustering analysis | Measured | Measured | Yes | 26386597 |

| | | | | | | | | | | | |
|---|---|---|---|---|---|---|---|---|---|---|---|
| SardiNIA | SardiNIA Study on Aging | Population-based | European | Ogliastra Region in Sardinia (Italy) | 4694 | 95% | 1) gender discrepancy with genetic data from X-linked marker; 2) discrepancy between genetically inferred relationship with other samples and reported pedigree | Measured | Measured | Yes | 16934002, 22291609 |
| SHIP | Study of Health in Pomerania | Population-based | European | Germany - West Pomerania | 4081 | >92% | 1) duplicates<br>2) gender discrepancy | Measured | Measured | Yes | 20167617 |
| THISEAS | The Hellenic study of Interactions between Snps and Eating in Atherosclerosis Susceptibility | CAD case-control | European | Greece | 1000 | ≥95% | 1) sex mismatch<br>2) ethnic outliers<br>3) heterozygosity (3 SD) | Measured | Measured | Yes | 20167083 |
| TRAILS | Tracking Adolescents' Individual Lives Survey | Population-based | European | The Netherlands | 1354 | 95% | 1) heterozygosity<br>2) duplicate and MZ samples<br>3) sex mismatch<br>4) non-caucasians | Measured | Measured | Yes | 18263649, 23021478, 25431468 |
| TwinsUK | TwinsUK | Population-based | European | UK | 5654 | >98% | 1) call-rate < 98%<br>2) heterozygosity across all SNPs ≥2 s.d. from the sample mean<br>3) duplicated samples<br>4) gender mismatch<br>5) evidence of non-European ancestry as assessed by PCA [illegible] | Measured | Measured | Yes | 23088889 |
| WGHS | Women's Genome Health Study | Population-based | European | USA | 23294 | 98% | None | Self-reported | Self-reported | No, waist and WHR assessed 6 years after BMI | 18070814 |
| YFS | The Cardiovascular Risk in Young Finns Study | Population-based | European | Finland | 2442 | 95% | 1) gender mismatch<br>2) excess heterozygosity<br>3) duplicates<br>4) cryptic relatedness<br>5) ethnic outliers | Measured | Measured | Yes | 18263651 |

**METABOCHIP DATA**

| Study | | Study design | Ancestry | Region | Total sample size (N) | Sample QC | | Anthropometric assessment method | | | Study References (PMID) |
|---|---|---|---|---|---|---|---|---|---|---|---|
| Short name | Full name | | | | | Call rate* | sample exclusion criteria | BMI | WHR | WHR and BMI were assessed at the same time? | |
| DESIR | Data from an Epidemiological Study on the Insulin Resistance syndrome | Population based | European | France | 4993 | ≥ 90% | 1) missing data<br>2) ethnic outliers (n=66 dropped using PCA) | Measured | Measured | Yes | 8927780 |
| DR's EXTRA | DR's EXTRA | Population-based | European | Finland | 1250 | >99% | 1) missing phenotypes or age<18<br>2) gender discrepancy or anomaly<br>3) unexplained duplicate sample<br>4) relateds across DR's EXTRA, FUSION, and METSIM | Measured | Measured | Yes | 8177243 |
| EGCUT Metabochip | Estonian Genome Center, University of Tartu | Population-based | European | Estonia | 2510 | ≥ 98% | 1) call-rate < 95%<br>2) related smaller than 2nd degree<br>3) duplicated samples<br>4) gender mismatch | Measured | Measured | Yes | 24518929 |
| Ely | MRC Ely Study | Population-based | European | UK | 1602 | 95% | 1) sex-check<br>2) relatedness check<br>3) ethnic ancestry outlying<br>4) hetezygosity check | Measured | Measured | Yes | 17257284 |

| | | | | | | | | | | | |
|---|---|---|---|---|---|---|---|---|---|---|---|
| EPIC | European Prospective Investigation into Cancer and Nutrition - Obesity Study | Case-cohort design | European | UK | 1700 | 95% | 1) sex-check<br>2) relatedness check<br>3) ethnic ancestry outlying<br>4) hetezygosity check | Measured | Measured | Yes | 10466767<br>12795830 |
| Fenland | Fenland Study | Population-based | European | UK | 3217 | 95% | 1) sex-check<br>2) relatedness check<br>3) ethnic ancestry outlying<br>4) hetezygosity check | Measured | Measured | Yes | 20519560 |
| FUSION2 | Finland-United States Investigation of NIDDM Genetics | Case-control | European | Finland | 2014 | >99% | 1) missing phenotypes or age<18<br>2) gender discrepancy or anomaly<br>3) unexplained duplicate sample<br>4) relateds across DR's EXTRA, FUSION, and METSIM | Measured | Measured | Yes | 17463248 |
| GLACIER | Gene x Lifestyle Interactions and Complex Traits Involved in Elevated Disease Risk | Population-based | European | Sweden | 6064 | ≥95% | 1) call-rate < 95%<br>2) sex mismatch | Measured | Waist, but not hip circumference | Yes | 25396097 |
| GXE | Gene By Environment | Population-based | Jamaican | Caribbean | 613 | ≥95% | 1) missing phenotype data<br>2) heterozygosity<br>3) PCA outliers 4) IBD cryptic relateds | Measured | Measured | Yes | 9103091,<br>9098179,<br>20400458 |
| Health2006 | Health2006 | Population-based | European | Capital region of Denmark | 3207 | >99% | 1) individuals with 1st or 2nd degree familial relationship<br>2) extreme inbreeding coefficient<br>3) low call rate<br>4) mislabelled sex and<br>5) high discordance to previous genotypings | Measured | Measured | Yes | 23615486 |
| HUNT | The Nord-Trøndelag Health Study | Population-based with enrichment for T2D cases | European | Norway | 1567 | >99% | 1) missing phenotypes or age<18<br>2) gender discrepancy or anomaly<br>3) unexplained duplicate sample<br>4) relateds | Measured | Measured | Yes | [No PMID] Holmen J, Midthjell K, et al. The Nord-Trøndelag Health Study 1995-97 (HUNT 2):Objectives, contents, methods and participation. |
| IMPROVE | Carotid Intima Media Thickness (IMT) and IMT-PRogression as Predictors of Vascular Events in a High-Risk European Population | Population-based (high CVD-risk) | European | Europe (Italy, France, Netherlands, Sweden, Finland) | 3426 | ≥ 95% | 1) ambiguous sex<br>2) cryptic relatedness<br>3) non-european descent | Measured | Measured | Yes | 22999719 |
| Inter99 (I99) | Inter99 | Population-based | European | Capital region of Denmark | 6127 | >99% | 1) individuals with 1st or 2nd degree familial relationship<br>2) extreme inbreeding coefficient<br>3) low call rate<br>4) mislabelled sex and<br>5) high discordance to previous genotypings | Measured | Measured | Yes | 14663300<br>12882858<br>19898830 |
| KORA S3 | Cooperative Health Research in the Region of Augsburg | Population-based | European | Germany | 3,113 | 93% | none | Measured | Measured | Yes | 16032513<br>16032514 |
| KORA S4 | Cooperative Health Research in the Region of Augsburg | Population-based | European | Germany | 3,028 | 93% | none | Measured | Measured | Yes | |

| | | | | | | | | | | | |
|---|---|---|---|---|---|---|---|---|---|---|---|
| MEC | The Multiethnic Cohort Study | Population-based | African | USA-North America | ≥ 95% | >95% | 1) first-degree relatives<br>2) ancestry outliers<br>3) gender mismatch<br>4) identity issues | Self-reported | Self-measured | Yes | 10695593 |
| METSIM | Metabolic Syndrome In Men | Population-based with enrichment for T2D cases | European | Finland | 2162 | >99% | 1) missing phenotypes or age<18<br>2) gender discrepancy or anomaly<br>3) unexplained duplicate sample<br>4) relateds across DR's EXTRA, FUSION, and METSIM | Measured | Measured | Yes | 19223598 |
| NSHD | MRC National Survey of Health & Development | Birth cohort | European | UK | 2476 | 95% | 1) sex-check<br>2) relatedness check<br>3) ethnic ancestry outlying<br>4) hetezygosity check | Measured | Measured | Yes | 15814867<br>16204333 |
| SCARFSHEEP | Stockholm Coronary Atherosclerosis Risk Factor-Stockholm Heart Epidemiology Program | MI case-control | European | Europe (Stockholm, Sweden) | 2899 | ≥95% | 1) ambiguous sex<br>2) cryptic relatedness<br>3) non-european descent | Measured | Measured | Yes | 16238676<br>10447785 |
| SPT | Spanish Town | Population-based | Jamaican | Caribbean | 476 | ≥95% | 1) missing phenotype data<br>2) heterozygosity<br>3) PCA outliers<br>4) IBD cryptic relateds | Measured | Measured | Yes | 9103091,<br>9098179,<br>20400458 |
| WHI | Women's Health Initiative Study | Population-based | African | USA-North America | YR3/YR4 (3508) ; Pilot (2203) | >95% | 1) first-degree relatives<br>2) ancestry outliers<br>3) gender mismatch<br>4) identity issues<br>5) excessive heterozygosity<br>6) missing height or weight | Measured | Measured | Yes | 14575938 |
| Whitehall | The Whitehall II study | Cohort of London-based civil servants | European | UK | 2413 | 95% | 1) missing phenotype data<br>2) gender check<br>3) duplicates check<br>4) relatedness check<br>5) ethnic check | Measured | Measured | Yes | 15576467<br>21441441 |
| **VALIDATION DATA** | | | | | | | | | | | |
| UKBB | UK Biobank | Population study | White British | UK | 120,286 | NA, See UKB document on QC http://biobank.ctsu.ox.ac.uk/showcase/refer.cgi?id=155580 | 1) Sex mismatch<br>2) HWE P<1x10-6<br>3) non British ancestry | Measured | Measured | Yes | DOI: 10.1371/journal.pmed.1001779 |

**Supplementary Table 2.** Information on genotyping methods, quality control of SNPs, imputation, and statistical analysis for GWAS and Metabochip study cohorts. Abbreviations: MAF- minor allele frequency, HWE- Hardy-Weinberg Equilibrium, QC- quality control, SNP- single nucleotide polymorphism, GWAS- genome-wide association study.

| GWAS DATA | | | | | | | | | | | |
|---|---|---|---|---|---|---|---|---|---|---|---|
| | | Genotyping | | | | | Imputation | | | | |
| | | | | SNP QC | | | | | | | |
| Study | Ancestry | Genotyping array | Genotype calling software | Call rate* | p for HWE | No. of SNPs after QC | No. of SNPs used for imputation | Imputation Sofware | Imputation reference panel (NCBI build) | Imputed X chromosome data available? (software, reference) | Statistical Analysis Software |
| AE | European | Affymetrix SNP 5.0 | BRLMM-P | ≥ 97% | $> 10^{-6}$ | 403,789 | 403,789 | BEAGLE v3.3.2 | HapMap r 22 CEU | No | PLINKv1.07 |
| AGES | European | Illumina Hu370CNV | BeadStudio | >97% | $> 10^{-6}$ | 325,094 | 308,340 | Mach + MiniMac | HapMap r 22 CEU | Yes, (IMPUTE2) | ProbABEL |
| ARIC | European | Affymetrix 6.0 | Birdseed | ≥ 90% | $> 10^{-6}$ | 685,812 | 685,812 | MACH v1.0.16 | HapMap CEU, release 22, build 36 | No | ProbABEL |
| ARIC | African American | Affymetrix 6.0 | Birdseed | ≥ 90% | $> 10^{-6}$ | 685,812 | 685,812 | MACH v1.0.16 | HapMap YRI, release 22, build 36 | No | ProbABEL |
| AUSTWIN | European | Illumina370, Illumina610 | Beadstudio-gencall v3.0 | 95% | $> 10^{-6}$ | 269,840 | 269,840 | MACH v1.0.15 | HapMap r22 (build 36) | No | ProbABEL,R |
| BHS | European | Illumina 610 Quad | BeadStudio | >95% | $> 10^{-6}$ | 529,526 | 529,526 | MaCH | HapMap r 22 CEU | No | ProbABEL, mach2qtl |
| BioMe (MSSM) | European American | Illumina HumanOmniExpressExome-8 v1.0 | zCall (GenomeStudio) | ≥ 90% | $> 10^{-6}$ | 906,917 | 768,517 | IMPUTE2 | I000G v3 (March 2012) | Yes | SAS, Quicktest |
| BioMe (MSSM) | African American | Illumina HumanOmniExpressExome-8 v1.0 | zCall (GenomeStudio) | ≥ 90% | $> 10^{-6}$ | 906,917 | 768,517 | IMPUTE2 | I000G v3 (March 2012) | Yes | SAS, Quicktest |
| BioMe (MSSM) | Hispanic / Latinos | Illumina HumanOmniExpressExome-8 v1.0 | zCall (GenomeStudio) | ≥ 90% | $> 10^{-6}$ | 906,917 | 768,517 | IMPUTE2 | I000G v3 (March 2012) | Yes | SAS, Quicktest |
| BLSA | European | Illumina 450K | BeadStudio | ≥ 99% | $> 10^{-6}$ | 514,027 | 514,027 | MACH | HapMap release22, build 36 | No | ProbABEL |
| British 1958 birth cohort (B58C) | European | Illumina 550k_v1, 550k_v3, 610k (3 non-overlapping subsets) | BeadStudio | ≥ 95% | $> 10^{-4}$ | 500,521 | 500,521 | MACH/Minimac | HapMap r 21 CEU | Yes, (MACH/Minimac) | ProbABEL |
| CHS | European | Illumina 370CNV BeadChip | Illumina BeadStudio | ≥ 97% | $> 10^{-5}$ | 306,655 | 306,655 | BIMBAM v0.99 | HapMap release22, build 36 | Yes, (build 36, release 24) | R |
| CLHNS | East Asian (Filipino) | Affymetrix Genome-Wide Human SNP Array 5.0 | Birdseed (version 2) | ≥ 90% | $> 10^{-6}$ | 352,264 | 352,264 | MACH v.1.0 | HapMap r22 CHB+JPT+CEU | No | ProbABEL, mach2qtl |
| COLAUS | European | Affymetrix 500k | APT | 90% | $> 10^{-7}$ | 390,631 | 390,631 | IMPUTE | HapMap r 21 CEU | No | Matlab |
| CROATIA-Korcula | European | Illumina HumanHap370 CNV | BeadStudio | >98% | $> 10^{-6}$ | 316,730 | 316,730 | MACH | HapMap r 22 CEU | No | ProbABEL |
| CROATIA-Vis | European | Illumina HumanHap300 | BeadStudio | >98% | $> 10^{-6}$ | 308,996 | 308,996 | MACH | HapMap r 22 CEU | No | ProbABEL |
| DESIR | European | Illumina Human CNV370-Duo Array and Illumina HAP300 array | BeadStudio | ≥ 95% | $> 10^{-4}$ | 291 609 | 291 609 | IMPUTE2 | HapMap r 22 CEU | No | SNPtest |
| EGCUT GWAS | European | Illumina OmniExpress | Illumina BeadStudio | 95% | $> 10^{-6}$ | 611,549 | 611,549 | IMPUTE2 | HapMap r 22 CEU | No | R, Quicktest |
| EPIC-Norfolk | European | Affymetrix 500K | BRLMM | ≥ 90% | $> 10^{-6}$ | 382,036 | 382,036 | IMPUTE | HapMAP 21 CEU | Yes | Quicktest |
| ERF | European | Illumina 318K, 370K, 610K, Affymetrix 250K | Genome Studio & Beadstudio, BRLMM | 95% | $> 10^{-6}$ | 649,956 | 635,956 | MACH 1.0.16 | HapMap release22, build 36 | Yes, (build 36, rel 22 (IMPUTE)) | GenABEL, ProbABEL |
| FamHS | European | Illumina HumMap 550k, Human610-Quadv1, Human 1M-Duov3 | BeadStudio | 98% | $> 10^{-6}$ | 2,543,887 | 499,558 | MaCH | HapMap r 22 CEU | Yes | SAS, R |
| Fenland | European | Affymetrix SNP5.0 | BRLMM | 95% | $> 10^{-6}$ | 360,602 | 360,602 | IMPUTE2 | 1000GP Phase1 v3 | Yes | SNPtest, Quicktest |
| FramHS | European | Affymetrix 500K<br>Affymetrix 50K supplemental | BRLMM | >97% | $> 10^{-6}$ | 378,163 | 378,163 | MACH v1.0.15 | HapMap r 22 CEU | Yes | R |
| FUSION | European | Illumina HumanHap300 | BeadStudio | >98% | $> 10^{-6}$ | 315,635 | 315,635 | MaCH | HapMap r 22 CEU | No | ProbABEL |
| Gendian | European | Genome-Wide Human SNP Array 6.0 | Affymetrix - birdseed, (algorithm: BRLMM) | MAF>.1 & callrate<.9 MAF>.09 & MAF ≤.1 & callrate <.91 | $> 10^{-6}$ | 747,402 | 747,402 | Mach 1.0.18.c MiniMac 2012-10-09 | GIANT ALL 1000G v3 ref panel GRCh build 37 | Yes, (MaCH/ minimac, GIANT ALL 1000G v3 ref panel GRCh build 37) | ProbAbel, R, Plink |
| Generation Scotland | European | Illumina HumanOmniPlusExome | BeadStudio | >98% | $> 10^{-6}$ | 706,980 | 706,980 | ShapeIt2 + IMPUTE2 | 1000G (ALL) | Yes, (IMPUTE2) | ProbABEL |
| GENOA | European American | Affymetrix 6.0 & Illumina 1M-Duo Bead Chip | Birdseed and Genome Studio | ≥ 95% | NA | Affymetrix: 596,941; Illumina: 804,154 | Affymetrix: 596,941; Illumina: 804,154 | MACH 1.0.16 | HapMap (release 22) CEU | No | MMAP |
| GOOD | European | Illumina 610 | Beadstudio | >98% | $> 10^{-6}$ | 553,191 | 521,160 | MaCH | HapMap CEU, release 22, build 36 | Yes | PLINK, R |
| GOYA cases | European | Illumina 610K Quad | GenomeStudio | ≥ 95% | $> 10^{-7}$ | 545,349 | 545,349 | Mach 1.0 | HapMap r22 (build 36) | No | Quicktest |
| GOYA controls | European | Illumina 610K Quad | GenomeStudio | ≥ 95% | $> 10^{-7}$ | 545,349 | 545,349 | Mach 1.0 | HapMap r22 (build 36) | No | Quicktest |
| HERITAGE Family Study | European | Illumina 370CNV | GenomeStudio | ≥ 98% | $> 10^{-6}$ | 324,607 | 324,607 | MaCH | HapMap r 22 CEU | No | ProbABEL |
| HRS | European American | Illumina Omni2.5 Beadchip | BeadStudio | 98% | $> 10^{-4}$ | 1,681,327 | 551,936 | MaCH | HapMap r 22 CEU | Yes, (MaCH) | PLINK, ProbABEL |
| HRS | African American | Illumina Omni2.5 Beadchip | BeadStudio | 98% | $> 10^{-4}$ | 1,681,327 | 909,595 | MaCH | HapMap r 22 CEU+YRI | No | PLINK, ProbABEL |
| HYPERGENES | European | Illumina 1M-Duo | Genome-Studio | ≥ 99% | $> 10^{-8}$ | 882,854 | 882,854 | Minimac | HapMap r 22 CEU | No | PLINK |
| InCHIANTI | European | Illumina 450K | BeadStudio | ≥ 99% | $10^{-4}$ | 498,838 | 498,838 | MACH | HapMap release22, build 36 | No | ProbABEL |
| KORA3 | European | Affymetrix 500K | BRLMM | none | none | No | 490,032 | MACH v1.0.9 | HapMap r 22 CEU | No | ProbABEL, R |
| KORA4 | European | Affymetrix 6.0 | Birdseed | none | none | 909,622 | 909,622 | IMPUTE v0.4.2 | HapMap r 22 CEU | No | ProbABEL, R |
| Lifelines | European | Illumina Cyto SNP12 v2 | GenomeStudio | >95% | $> 10^{-4}$ | 257,581 | 257,581 | BEAGLE v3.3.2 | HapMap r24 CEU (build 36) | No | PLINKv1.07 |
| LOLIPOP_EW610 | European | Illumina Human610 | BeadStudio | 95% | $> 10^{-6}$ | 544,620 | 544,620 | IMPUTE2 | HapMap r 22 CEU | No | Quicktest |
| LOLIPOP_IA317 | Indian Asian | Illumina HumanHap300 | BeadStudio | 95% | $> 10^{-6}$ | 307,303 | 307,303 | IMPUTE2 | HapMap r 22 population combined | No | Quicktest |

| | | | | | | | | | | | |
|---|---|---|---|---|---|---|---|---|---|---|---|
| LOLIPOP_IA610 | Indian Asian | Illumina Human610 | BeadStudio | 95% | > $10^{-6}$ | 544,390 | 544,390 | IMPUTE2 | HapMap r 22 population combined | No | Quicktest |
| LURIC | European | Affymetrix 6.0 | Birdseed v2 | ≥ 98% | > $10^{-4}$ | 686,195 | 686,195 | MaCH | HapMap r 22 CEU | No | ProbABEL |
| MESA | European | Affymetrix 6.0 | Birdseed v2 | >95% | NA | 854,755 | 854,755 | IMPUTE v2.1.0 | HapMap r24 CEU | No | ProbABEL |
| MrOS Sweden | European | Illumina 1M | Beadstudio | 97% | > $10^{-4}$ | 739,477 | 714,543 | Minimac | HapMap CEU, release 22, build 36 | No | PLINK, R |
| Netherlands Twin Register | European | Affymetrix 6.0 + affymetrix perlegen 5.0 + Illumina 370 + Illumina 660 + Illumina Omni Express 1 M | Beadstudio | >95% | > $10^{-6}$ | 311,567-932,824 | 311567-932824 | IMPUTE 2 | Hapmap 2 build 36 release 24 CEU reference set | No | Quicktest |
| NFBC66 | European | Illumina HumanCNV370DUO Analysis BeadChip | Beadstudio | ≥ 95%, MAF< 5%, call rate ≥ 99% | ≥ $10^{-7}$ | 324,896 | 324,896 | IMPUTE2 | HapMap r 22 CEU | No | SNPTEST2, Quicktest |
| NHS | European | Affymetrix 6.0 | BeadStudio | 95% | > $10^{-6}$ | 704,409 | 704,409 | MaCH | HapMap r 22 CEU | No | ProbABEL, R |
| ORCADES | European | Illumina HumanHap300 | BeadStudio | >98% | > $10^{-6}$ | 293,687 | 293,687 | MACH | HapMap r 22 CEU | No | ProbABEL |
| PIVUS | European | Human Omni Express and Metabochip | GenCall | 95% | >$10^{-6}$ | 738,879 | 738,879 | IMPUTE2 | HapMap r 22 CEU | NA | SNPTEST, Quicktest |
| Prevend | Euopean | Illumina Cyto SNP12 v2 | GenomeStudio | >95% | > $10^{-4}$ | 232,571 | 232,571 | BEAGLE v3.3.2 | phased CEU haplotypes, HapMap release 22 (build 36) | No | PLINK, ProbABEL |
| PROSPER | European | Illumina 660K Quad | Beadstudio | ≥ 98% | > $10^{-6}$ | 557,192 | 557,192 | MACH 1.0.15 | HapMap r 22 CEU | No | PROBABEL |
| QFS | European | Illumina 610-Quad chip | BeadStudio | 95% | > $10^{-4}$ | 543,713 | 525,406 | MACH | HapMap r 22 CEU | No | GWAF 2.0, R |
| Rotterdam Study I | European | Version 3 Illumina Infinium II, HumanHap 550 SNP chip array | BeadStudio | >98 % | > $10^{-6}$ | 512,349 | 512,349 | MACH | HapMap release22, build 36 | No | SPSS, ProbABEL, R |
| Rotterdam Study II | European | Version 3 Illumina Infinium II, HumanHap 550 SNP chip array | BeadStudio | >98 % | > $10^{-6}$ | 466,389 | 466,389 | MACH | HapMap release22, build 36 | No | SPSS, ProbABEL, R |
| Rotterdam Study III | European | Version 3 Illumina Infinium II, HumanHap 550 SNP chip array | BeadStudio | >98 % | > $10^{-6}$ | 514,073 | 514,073 | MACH | HapMap release22, build 36 | No | SPSS, ProbABEL, R |
| SardiNIA | European | Affymetrix 10K, 500K, 6.0 | BRLMM, Birdseed | 95% | ≥ $10^{-6}$ | 759,213 | 731,209 | MACH | HapMap r 22 CEU | No | ProbABEL, R |
| SHIP | European | Affymetrix 6.0 | Birdseed2 | >92% | NA | 869,224 | 869,224 | IMPUTE v0.5.0 | Hapmap r22 CEU | No | Quicktest |
| THISEAS | European | Illumina OmniExpress | Illuminus | ≥98% | > $10^{-6}$ | 733,202 | NA | NA | NA | No | Plink, Quicktest |
| TRAILS | European | Illumina Cyto SNP12 v2 | GenomeStudio | 95% | > $10^{-4}$ | 260,127 | 260,127 | IMPUTE2 | HapMap r 22 CEU | Yes | Quicktest |
| TwinsUK | European | Illumina 318K, 610K | Illluminus | MAF≥.05 & callrate <.97 MAF≥.01 & MAF <.05 & | > $10^{-6}$ | Illumina 318K: 303,940, 610K: 553,487 | Illumina 318K: 303,940, 610K: 553,487 | IMPUTE2 | HapMap r 22 CEU | No | Quicktest |
| WGHS | European | Illumina Human Hap Duo 300 + | BeadStudio | 98% | > $10^{-6}$ | 339,596 | 333,470 | MaCH | HapMap r 22 CEU | Yes | ProbABEL, R |
| YFS | European | Illumina 670k custom | Illuminus | 95% | > $10^{-6}$ | 546,674 | 534,047 | MACH 1.0 | HapMap release22, build 36 | No | ProbABEL |

**METABOCHIP DATA**

| Study | Ancestry | Genotyping | | | | | Imputation | | | | Statistical Analysis Software |
|---|---|---|---|---|---|---|---|---|---|---|---|
| | | Genotyping array | Genotype calling software | SNP QC | | No. of SNPs after QC | No. of SNPs used for imputation | Imputation Sofware | Imputation reference panel (NCBI build) | Imputed X chromosome data available? (software, reference) | |
| | | | | Call rate* | p for HWE | | | | | | |
| DESIR | European | Illumina Metabochip | BeadStudio | ≥ 95% | > $10^{-4}$ | 194,763 | NA | NA | NA | No | PLINK, SNPtest, Quicktest |
| DR's EXTRA | European | Illumina Metabochip | BeadStudio | >98% | > $10^{-6}$ | 188,072 | NA | NA | NA | No | Quicktest |
| EGCUT Metabochip | European | Illumina Metabochip | BeadStudio | 95% | > $10^{-6}$ | 138,179 | No | No | No | No | R, Quicktest |
| Ely | European | Illumina Metabochip | GeneCall | 95% | > $10^{-6}$ | 144,581 | NA | NA | NA | No | PLINK, Quicktest |
| EPIC-Norfolk | European | Illumina Metabochip | GeneCall | 95% | > $10^{-6}$ | 140,943 | NA | NA | NA | No | PLINK, Quicktest |
| Fenland | European | Illumina Metabochip | GeneCall | 95% | > $10^{-6}$ | 149,819 | NA | NA | NA | No | PLINK, Quicktest |
| FUSION2 | European | Illumina Metabochip | BeadStudio | >98% | > $10^{-6}$ | 188,072 | NA | NA | NA | No | Quicktest |
| GLACIER | European | Illumina Metabochip | Illuminus | ≥ 95% | > $10^{-5}$ | 149,782 | NA | NA | NA | No | SAS, Quicktest |
| GXE | Jamaican | Illumina Metabochip | Birdseed | ≥ 90% | > $10^{-6}$ | 195,531 | NA | NA | NA | NA | R, PLINK |
| Health2006 | European | Illumina Metabochip | GenomeStudio | none | none | 196,725 | NA | NA | NA | No | PLINK |
| HUNT | European | Illumina Metabochip | BeadStudio | >98% | > $10^{-6}$ | 188,573 | NA | NA | NA | No | Quicktest |
| IMPROVE | European | Metabochip | GeneCall | ≥ 95% | > $10^{-6}$ | 185,704 | NA | NA | NA | No | PLINK |
| Inter99 (I99) | European | Illumina Metabochip | GenomeStudio | none | none | 196,725 | NA | NA | NA | No | PLINK |
| KORA S3 | European | Illumina Metabochip | Genome Studio | none | none | 185,781 | NA | NA | NA | No | ProbABEL, R |
| KORA S4 | European | Illumina Metabochip | Genome Studio | none | none | 185,781 | NA | NA | NA | No | ProbABEL, R |
| MEC | African | Illumina Metabochip | GenomeStudio with the GenCall 2.0 algorithm | ≥ 95% | > $10^{-6}$ | 196,725 | NA | NA | NA | No | PLINK |
| METSIM | European | Illumina Metabochip | BeadStudio | >98% | > $10^{-6}$ | 188,072 | NA | NA | NA | No | Quicktest |
| NSHD | European | Illumina Metabochip | GeneCall | 95% | > $10^{-6}$ | 147,949 | NA | NA | NA | No | PLINK, Quicktest |
| SCARFSHEEP | European | Metabochip | GeneCall | ≥ 95% | > $10^{-6}$ | 185,704 | NA | NA | NA | No | PLINK |
| SPT | Jamaican | Illumina Metabochip | Birdseed | ≥ 90% | > $10^{-6}$ | 195,567 | NA | NA | NA | NA | R, Plink |
| WHI | African | Illumina Metabochip | GenomeStudio with the GenCall 2.0 algorithm | ≥ 95% | > $10^{-6}$ | 196,725 | NA | NA | NA | No | PLINK |
| WHITEHALL | European | Illumina Metabochip | GeneCall | 95% | > $10^{-6}$ | 151,534 | NA | NA | NA | No | Plink, Quicktest |
| **VALIDATION DATA** | | | | | | | | | | | |
| UKBB | White British | UK Biobank Axiom Array and UK BiLEVE array | Affymetrix Power Tools | NA | NA | NA | NA | NA | NA | NA | STATA |

**Supplementary Table 3.** Study-specific descriptives for total sample from contributing studies. Abbreviations: BMI- body mass index, WHR- waist to hip ratio.

| GWAS Data | | | | | | | | | | | | | | | | | | | | | |
|---|---|---|---|---|---|---|---|---|---|---|---|---|---|---|---|---|---|---|---|---|---|
| Study | Ancestry | Groups | N | Age | | BMI (kg/m$^2$) | | BMI Categories* | | | | | | Waist (cm) | | WHR (ratio) | | Current Smoking Status | | | |
| | | | | | | | | Normal weight | | Overweight | | Obese | | | | | | Smoker | | Non-Smoker | |
| | | | | mean | sd | mean | sd | n | % | n | % | n | % | mean | sd | mean | sd | n | % | n | % |
| AE | EU | Overall | 627 | 67.70 | 8.84 | 26.30 | 3.65 | 242 | 38.60 | 307 | 49.00 | 78 | 12.40 | NA | NA | NA | NA | 228 | 36.40 | 397 | 63.30 |
| | | women | 190 | 67.64 | 9.63 | 26.32 | 4.69 | 77 | 12.30 | 82 | 13.10 | 31 | 4.90 | NA | NA | NA | NA | 80 | 12.80 | 110 | 17.60 |
| | | men | 435 | 67.72 | 8.50 | 26.26 | 3.19 | 165 | 26.30 | 225 | 35.90 | 47 | 7.50 | NA | NA | NA | NA | 148 | 23.70 | 287 | 45.90 |
| AGES | EU | Overall | 3,214 | 76.41 | 5.45 | 27.11 | 4.43 | 1,008 | 31.69 | 1,465 | 31.69 | 708 | 22.26 | 100.80 | 12.18 | NA | NA | 409 | 12.73 | 2,805 | 87.27 |
| | | women | 1,863 | 76.33 | 5.55 | 27.19 | 4.85 | 602 | 32.79 | 788 | 32.79 | 446 | 24.29 | 99.54 | 13.14 | NA | NA | 247 | 13.26 | 1,616 | 86.74 |
| | | men | 1,351 | 76.52 | 5.32 | 26.99 | 3.78 | 406 | 30.19 | 677 | 30.19 | 262 | 19.48 | 102.54 | 10.48 | NA | NA | 162 | 11.99 | 1,189 | 88.01 |
| ARIC | AF | Overall | 2,618 | 53.31 | 5.79 | 29.67 | 6.08 | 547 | 20.89 | 1,007 | 38.46 | 1,064 | 40.64 | 99.30 | 15.01 | 0.92 | 0.08 | 781 | 29.80 | 1,837 | 70.20 |
| | | women | 1,649 | 53.18 | 5.67 | 30.66 | 6.51 | 290 | 17.59 | 581 | 35.23 | 778 | 47.18 | 100.17 | 16.19 | 0.91 | 0.08 | 416 | 25.23 | 1,233 | 74.77 |
| | | men | 969 | 53.52 | 5.99 | 27.99 | 4.82 | 257 | 26.52 | 426 | 43.96 | 286 | 29.51 | 97.82 | 12.64 | 0.94 | 0.05 | 365 | 37.67 | 604 | 62.33 |
| ARIC | EU | Overall | 7,930 | 54.27 | 5.69 | 27.02 | 4.88 | 2,942 | 37.10 | 3,179 | 40.09 | 1,809 | 22.81 | 96.19 | 13.39 | 0.93 | 0.08 | 1,996 | 25.17 | 5,934 | 74.83 |
| | | women | 4,188 | 53.93 | 5.66 | 26.62 | 5.51 | 2,048 | 46.30 | 1,343 | 30.36 | 1,032 | 23.33 | 93.02 | 14.86 | 0.89 | 0.08 | 1,091 | 26.05 | 3,097 | 73.95 |
| | | men | 3,742 | 53.66 | 5.71 | 27.48 | 4.01 | 1,068 | 26.84 | 2,020 | 50.77 | 891 | 22.39 | 99.73 | 10.46 | 0.97 | 0.05 | 905 | 24.18 | 2,837 | 75.82 |
| AUSTWIN | EU | Overall | 2,166 | 50.90 | 15.31 | 26.78 | 5.06 | 863 | 39.80 | 829 | 38.30 | 459 | 21.20 | NA | NA | NA | NA | 448 | 20.70 | 1,718 | 79.30 |
| BHS | EU | women | 645 | 54.42 | 16.83 | 25.57 | 4.42 | 338 | 52.40 | 196 | 30.39 | 107 | 16.59 | 80.96 | 11.55 | 0.80 | 0.07 | 55 | 8.50 | 590 | 91.50 |
| | | men | 413 | 53.49 | 16.92 | 26.57 | 3.66 | 150 | 36.30 | 189 | 45.70 | 71 | 17.20 | 93.39 | 10.47 | 0.93 | 0.06 | 54 | 13.10 | 359 | 86.90 |
| BIOME | AF | Overall | 3,492 | 51.86 | 14.88 | 30.44 | 7.99 | 862 | 24.68 | 1,053 | 30.15 | 1,528 | 43.76 | NA | NA | NA | NA | 905 | 25.92 | 2,587 | 74.08 |
| | | women | 2,249 | 52.21 | 15.31 | 31.53 | 8.56 | 485 | 21.57 | 605 | 26.90 | 1,118 | 49.71 | NA | NA | NA | NA | 478 | 21.25 | 1,771 | 78.75 |
| | | men | 1,243 | 51.22 | 14.06 | 28.48 | 6.37 | 377 | 30.33 | 448 | 36.04 | 410 | 32.98 | NA | NA | NA | NA | 427 | 34.35 | 816 | 65.65 |
| BIOME | EU | Overall | 2,017 | 67.76 | 9.29 | 26.91 | 5.44 | 775 | 38.42 | 742 | 36.79 | 479 | 23.75 | NA | NA | NA | NA | 168 | 8.33 | 1,849 | 91.67 |
| | | women | 968 | 67.25 | 9.09 | 25.98 | 5.72 | 463 | 47.83 | 281 | 29.03 | 206 | 21.28 | NA | NA | NA | NA | 55 | 5.68 | 913 | 94.32 |
| | | men | 1,049 | 68.23 | 9.46 | 27.77 | 5.03 | 312 | 29.74 | 461 | 43.95 | 273 | 26.02 | NA | NA | NA | NA | 113 | 10.77 | 936 | 89.23 |
| BIOME | HS | Overall | 4,708 | 54.31 | 16.09 | 29.40 | 6.64 | 1,206 | 25.62 | 1,625 | 34.52 | 1,842 | 39.12 | NA | NA | NA | NA | 921 | 19.56 | 3,787 | 80.44 |
| | | women | 2,944 | 54.21 | 16.31 | 29.73 | 6.95 | 758 | 25.75 | 922 | 31.32 | 1,240 | 42.12 | NA | NA | NA | NA | 475 | 16.13 | 2,469 | 83.87 |
| | | men | 1,764 | 54.49 | 15.73 | 28.86 | 6.04 | 448 | 25.40 | 703 | 39.85 | 602 | 34.13 | NA | NA | NA | NA | 446 | 25.28 | 1,318 | 74.72 |
| BLSA | EU | women | 381 | 66.50 | 17.15 | 25.93 | 4.83 | 196 | 51.44 | 113 | 29.66 | 72 | 18.90 | 84.45 | 11.65 | 0.82 | 0.07 | 9 | 2.36 | 372 | 97.64 |
| | | men | 461 | 71.30 | 15.72 | 27.55 | 4.50 | 142 | 30.80 | 205 | 44.47 | 114 | 24.73 | 98.56 | 12.01 | 0.95 | 0.07 | 20 | 4.34 | 441 | 95.66 |
| B58C | EU | women | 3,255 | 45.16 | 0.39 | 26.92 | 5.53 | 1,426 | 43.80 | 1,063 | 32.70 | 751 | 23.00 | 85.32 | 12.61 | 0.81 | 0.06 | 809 | 24.90 | 2,446 | 75.20 |
| | | men | 3,226 | 45.17 | 0.38 | 27.88 | 4.29 | 791 | 24.50 | 1,594 | 49.40 | 834 | 25.90 | 98.62 | 11.11 | 0.93 | 0.06 | 732 | 22.70 | 2,494 | 77.30 |
| CHS | | women | 1,885 | 71.89 | 5.15 | 26.48 | 4.77 | 808 | 42.86 | 713 | 37.82 | 364 | 19.31 | 90.87 | 13.43 | 0.89 | 0.09 | 229 | 12.15 | 1,656 | 87.85 |
| | EU | men | 1,230 | 72.95 | 5.59 | 26.41 | 3.49 | 445 | 36.18 | 604 | 49.11 | 181 | 14.72 | 97.65 | 9.71 | 0.96 | 0.06 | 119 | 9.67 | 1,111 | 90.33 |
| CLHNS | AS | women | 1,793 | 48.45 | 6.10 | 24.31 | 4.37 | 1,029 | 57.39 | 581 | 32.40 | 183 | 10.21 | 81.10 | 10.88 | 0.88 | 0.05 | 267 | 14.89 | 1,526 | 85.11 |
| COLAUS | EU | women | 2,875 | 53.89 | 10.72 | 25.16 | 4.90 | 1,636 | 56.90 | 820 | 28.52 | 419 | 14.57 | 83.52 | 12.47 | 0.83 | 0.07 | 699 | 24.31 | 2,176 | 75.69 |
| | | men | 2,560 | 52.94 | 10.77 | 26.63 | 4.02 | 950 | 37.11 | 1,170 | 45.70 | 440 | 17.19 | 95.93 | 11.24 | 0.93 | 0.06 | 734 | 28.67 | 1,826 | 71.33 |
| CROATIA-KORCULA | EU | Overall | 898 | 56.27 | 13,94 | 27.96 | 4.14 | 206 | 23.41 | 417 | 47.39 | 257 | 29.20 | 94.19 | 12.16 | 0.90 | 0.09 | 203 | 22.91 | 683 | 77.09 |
| | | women | 573 | 55.54 | 13,71 | 27.52 | 4.35 | 165 | 29.26 | 250 | 44.33 | 149 | 26.42 | 90.85 | 11.91 | 0.87 | 0.08 | 140 | 24.78 | 425 | 75.22 |
| | | men | 325 | 57.55 | 14,27 | 28.76 | 3.60 | 41 | 12.97 | 167 | 52.85 | 108 | 34.18 | 100.12 | 10.18 | 0.96 | 0.07 | 63 | 19.63 | 258 | 80.37 |
| CROTIA-VIS | EU | Overall | 924 | 56.36 | 15.54 | 27.32 | 4.27 | 271 | 29.88 | 396 | 43.66 | 240 | 26.46 | 95.84 | 11.79 | 0.94 | 0.08 | 255 | 27.87 | 660 | 72.13 |
| | | women | 536 | 56.72 | 16.00 | 27.19 | 4.65 | 180 | 34.42 | 194 | 37.09 | 149 | 28.49 | 94.14 | 12.74 | 0.91 | 0.08 | 136 | 25.71 | 393 | 74.29 |
| | | men | 388 | 55.88 | 14.90 | 27.49 | 3.69 | 91 | 23.70 | 202 | 52.60 | 91 | 23.70 | 98.16 | 9.91 | 0.97 | 0.06 | 119 | 30.83 | 267 | 69.17 |
| DESIR | EU | Overall | 715 | 49.93 | 8.07 | 21.81 | 1.87 | 715 | 100 | 0 | 0.00 | 0 | 0 | 74.26 | 8.22 | 0.80 | 0.07 | 68 | 9.51 | 647 | 90.49 |
| | | women | 537 | 49.02 | 8.56 | 21.36 | 1.85 | 537 | 100 | 0 | 0.00 | 0 | 0 | 70.85 | 5.80 | 0.76 | 0.05 | 31 | 5.77 | 506 | 94.23 |
| | | men | 178 | 52.65 | 5.59 | 23.15 | 1.16 | 178 | 100 | 0 | 0.00 | 0 | 0 | 84.56 | 5.38 | 0.89 | 0.04 | 37 | 20.79 | 141 | 79.21 |
| EGCUT | EU | women | 1,083 | 65.99 | 21.42 | 27.32 | 5.59 | 402 | 37.15 | 373 | 34.47 | 307 | 28.37 | 88.63 | 14.14 | 0.85 | 0.09 | 125 | 11.54 | 958 | 88.46 |
| | | men | 976 | 56.53 | 19.06 | 26.95 | 4.82 | 367 | 37.64 | 394 | 40.41 | 214 | 21.95 | 96.31 | 13.75 | 0.94 | 0.09 | 338 | 34.63 | 638 | 65.37 |

| | | | | | | | | | | | | | | | | | | | | | |
|---|---|---|---|---|---|---|---|---|---|---|---|---|---|---|---|---|---|---|---|---|---|
| EPIC-Norfolk | EU | women | 1,285 | 58.75 | 9.00 | 26.19 | 4.39 | 569 | 44.38 | 489 | 38.14 | 224 | 17.47 | 81.94 | 10.77 | 0.79 | 0.06 | 130 | 10.25 | 1,138 | 89.75 |
| | | men | 1,132 | 59.81 | 9.03 | 26.62 | 3.21 | 347 | 30.68 | 626 | 55.35 | 158 | 13.97 | 95.81 | 9.45 | 0.93 | 0.06 | 124 | 11.04 | 999 | 88.96 |
| ERF | EU | women | 1,316 | 47.50 | 14.42 | 26.40 | 4.96 | 563 | 42.80 | 477 | 36.20 | 263 | 20.00 | 81.47 | 12.12 | 0.80 | 0.08 | 591 | 44.90 | 722 | 55.10 |
| | | men | 1,009 | 48.43 | 14.26 | 27.10 | 3.96 | 297 | 29.40 | 508 | 50.40 | 200 | 19.80 | 93.61 | 11.43 | 0.94 | 0.79 | 329 | 32.60 | 680 | 67.40 |
| FAMHS | EU | Overall | 3,663 | 52.20 | 13.64 | 27.75 | 5.49 | 1,241 | 34.00 | 1,371 | 37.56 | 1,038 | 28.44 | 97.65 | 15.31 | 0.92, | 0.09 | 525 | 14.37 | 3,138 | 85.67 |
| FENLAND | EU | women | 742 | 45.43 | 7.21 | 26.70 | 5.50 | 338 | 45.60 | 237 | 31.90 | 167 | 22.50 | 87.03 | 12.93 | 0.84 | 0.06 | 75 | 10.10 | 667 | 89.90 |
| | | men | 565 | 44.74 | 7.36 | 27.57 | 3.93 | 147 | 26.00 | 297 | 52.60 | 121 | 21.40 | 98.72 | 11.78 | 0.94 | 0.08 | 65 | 11.50 | 500 | 88.50 |
| FRAMINGHAM HEART STUDY (BMI) | EU | Overall | 8,354 | 37.75 | 9.35 | 25.80 | 4.92 | 4,090 | 49.50 | 2,829 | 34.24 | 1,344 | 16.27 | 93.04 | 15.15 | 0.88 | 0.10 | 2,507 | 30.01 | 5,847 | 69.99 |
| | | women | 4,518 | 37.60 | 9.23 | 24.82 | 5.25 | 2,831 | 63.65 | 1,003 | 22.55 | 614 | 13.80 | 88.38 | 15.69 | 0.81 | 0.07 | 1,320 | 29.22 | 3,198 | 70.78 |
| | | men | 3,836 | 37.93 | 9.50 | 26.95 | 4.23 | 1,259 | 33.00 | 1,826 | 47.86 | 730 | 19.13 | 98.30 | 12.60 | 0.96 | 0.06 | 1,187 | 30.94 | 2,649 | 69.06 |
| FRAMINGHAM HEART STUDY (WC) | EU | Overall | 7,100 | 42.23 | 10.87 | 26.85 | 5.22 | 3,342 | 47.56 | 2,454 | 34.92 | 1,231 | 17.52 | 91.38 | 14.95 | 0.88 | 0.10 | 1,380 | 19.44 | 5,720 | 80.56 |
| | | women | 3,745 | 45.04 | 10.78 | 26.01 | 5.82 | 2,318 | 62.80 | 824 | 22.32 | 549 | 14.87 | 85.48 | 15.11 | 0.81 | 0.07 | 713 | 19.04 | 3,032 | 80.96 |
| | | men | 3,355 | 45.43 | 10.96 | 27.78 | 4.28 | 1,024 | 30.70 | 1,630 | 48.86 | 682 | 20.44 | 97.97 | 11.66 | 0.96 | 0.06 | 667 | 19.88 | 2,688 | 80.12 |
| FRAMINGHAM HEART STUDY (WHR) | EU | Overall | 3,255 | 51.20 | 9.93 | 26.82 | 4.87 | 1,770 | 55.12 | 1,081 | 33.67 | 360 | 11.21 | 89.43 | 14.48 | 0.88 | 0.10 | 777 | 23.87 | 2,478 | 76.13 |
| | | women | 1,707 | 51.03 | 9.79 | 26.08 | 5.54 | 1,221 | 72.72 | 320 | 19.06 | 138 | 8.22 | 82.04 | 13.63 | 0.81 | 0.07 | 409 | 23.96 | 1,298 | 76.04 |
| | | men | 1,548 | 51.38 | 10.09 | 27.62 | 3.85 | 549 | 35.84 | 761 | 49.67 | 222 | 14.49 | 97.58 | 10.44 | 0.96 | 0.06 | 368 | 23.77 | 1,180 | 76.23 |
| FUSION-controls | EU | women | 47 | 60.82 | 7.48 | 26.98 | 4.00 | 17 | 36.20 | 21 | 44.70 | 9 | 19.10 | 86.63 | 10.44 | 0.83 | 0.06 | 30 | 63.83 | 17 | 36.17 |
| | | men | 60 | 68.15 | 7.34 | 27.20 | 3.72 | 19 | 31.70 | 30 | 50.00 | 11 | 18.30 | 98.29 | 9.96 | 0.95 | 0.06 | 24 | 40.00 | 36 | 60.00 |
| FUSION-T2D cases | | women | 63 | 62.16 | 7.82 | 31.76 | 4.92 | 5 | 7.90 | 17 | 27.00 | 41 | 65.10 | 101.88 | 13.18 | 0.90 | 0.06 | 25 | 39.70 | 38 | 60.30 |
| | | men | 261 | 62.85 | 7.15 | 29.31 | 4.07 | 33 | 12.60 | 121 | 46.40 | 107 | 41.00 | 104.75 | 10.93 | 0.98 | 0.07 | 55 | 21.10 | 206 | 78.90 |
| GENDIAN | EU | Overall | 449 | 65.01 | 10.37 | 31.59 | 5.87 | 39 | 8.69 | 170 | 37.86 | 240 | 53.45 | 111.14 | 15.04 | 1.00 | 0.12 | 47 | 10.47 | 402 | 89.53 |
| | | women | 212 | 66.66 | 10.54 | 32.30 | 6.15 | 15 | 7.08 | 74 | 34.91 | 123 | 58.02 | 110.31 | 15.74 | 0.96 | 0.13 | 16 | 7.55 | 196 | 92.45 |
| | | men | 237 | 63.53 | 10.00 | 30.96 | 5.54 | 24 | 10.13 | 96 | 40.51 | 117 | 49.37 | 111.89 | 14.37 | 1.03 | 0.11 | 31 | 13.08 | 206 | 86.92 |
| GENERATION SCOTLAND | EU | Overall | 9,852 | 52.21 | 13.63 | 27.05 | 5.11 | 2,447 | 36.57 | 2,706 | 40.44 | 1,539 | 23.00 | 90.30 | 13.84 | 0.87 | 0.09 | 1,543 | 15.86 | 8,186 | 84.14 |
| | | women | 5,782 | 51.92 | 13.52 | 26.86 | 5.59 | 1,632 | 41.33 | 1,402 | 35.50 | 915 | 23.17 | 86.19 | 13.65 | 0.83 | 0.09 | 855 | 14.97 | 4,858 | 85.03 |
| | | men | 4,070 | 52.62 | 13.78 | 27.32 | 4.29 | 815 | 29.71 | 1,304 | 47.54 | 624 | 22.75 | 96.24 | 11.82 | 0.93 | 0.07 | 688 | 17.13 | 3,328 | 82.87 |
| GENOA | EU | Overall | 1,434 | 55.31 | 10.81 | 30.41 | 6.35 | 264 | 18.41 | 521 | 36.33 | 649 | 45.26 | 100.09 | 15.64 | 0.91 | 0.09 | 181 | 12.62 | 1,253 | 87.38 |
| | | women | 792 | 55.04 | 10.82 | 30.56 | 7.20 | 190 | 23.99 | 233 | 29.42 | 369 | 46.59 | 96.27 | 17.16 | 0.86 | 0.08 | 92 | 11.62 | 700 | 88.38 |
| | | men | 642 | 55.63 | 10.79 | 30.21 | 5.12 | 74 | 11.53 | 288 | 44.86 | 280 | 43.61 | 104.80 | 11.96 | 0.97 | 0.06 | 89 | 13.86 | 553 | 86.14 |
| GOOD | EU | men | 731 | 24.10 | 0.60 | 23.60 | 3.40 | 535 | 73.20 | 148 | 20.20 | 32 | 4.40 | 84.40 | 8.10 | 0.85 | 0.04 | 48 | 6.60 | 683 | 93.40 |
| GOYA cases | EU | men | 671 | 43.00 | 6.23 | 35.61 | 5.63 | 5 | 0.74 | 99 | 14.75 | 567 | 84.50 | 116.90 | 14.07 | 1.02 | 0.09 | 353 | 52.60 | 318 | 47.39 |
| GOYA controls | | men | 790 | 47.76 | 8.55 | 26.12 | 3.60 | 315 | 39.92 | 372 | 47.14 | 102 | 12.92 | 93.48 | 10.52 | 0.93 | 0.07 | 396 | 50.13 | 393 | 49.75 |
| HERITAGE | EU | Overall | 499 | 35.84 | 14.56 | 25.86 | 4.96 | 247 | 49.50 | 155 | 31.10 | 97 | 19.40 | 90.44 | 14.89 | 0.88 | 0.08 | 75 | 15.00 | 424 | 85.00 |
| | | women | 255 | 35.12 | 14.14 | 25.04 | 4.89 | 146 | 57.20 | 70 | 27.40 | 39 | 15.30 | 86.22 | 14.53 | 0.84 | 0.08 | 41 | 16.10 | 214 | 83.90 |
| | | men | 244 | 36.59 | 14.96 | 26.71 | 4.90 | 101 | 41.40 | 85 | 34.80 | 58 | 23.80 | 94.84 | 13.98 | 0.92 | 0.07 | 34 | 13.90 | 210 | 86.10 |
| HRS | AF | women | 871 | 66.23 | 10.43 | 31.61 | 6.97 | 143 | 16.90 | 227 | 26.10 | 497 | 57.10 | 101.99 | 15.31 | NA | NA | 149 | 17.10 | 722 | 82.90 |
| | | men | 494 | 67.03 | 9.69 | 28.78 | 5.38 | 129 | 26.10 | 174 | 35.20 | 191 | 38.70 | 102.48 | 13.27 | NA | NA | 111 | 22.50 | 383 | 77.50 |
| HRS | EU | women | 4,727 | 68.25 | 10.78 | 28.65 | 6.25 | 1,483 | 64.70 | 1,548 | 32.80 | 1,696 | 35.90 | 95.95 | 15.45 | NA | NA | 603 | 12.80 | 4,124 | 87.20 |
| | | men | 3,415 | 68.93 | 9.76 | 29.12 | 4.93 | 679 | 19.90 | 1,445 | 42.30 | 1,291 | 37.80 | 105.10 | 11.92 | NA | NA | 440 | 12.90 | 2,975 | 87.10 |
| HYPERGENES-Cases | EU | women | 522 | 47.20 | 9.20 | 27.00 | 5.10 | 204 | 39.10 | 203 | 39.00 | 114 | 21.90 | 89.59 | 13.25 | 0.83 | 0.06 | 159 | 30.50 | 363 | 69.50 |
| | | men | 993 | 47.55 | 9.60 | 27.43 | 3.60 | 223 | 22.50 | 584 | 58.90 | 185 | 18.60 | 97.37 | 11.42 | 0.93 | 0.07 | 282 | 28.40 | 711 | 71.60 |
| HYPERGENES-Controls | | women | 563 | 60.60 | 12.00 | 25.00 | 3.80 | 306 | 55.00 | 198 | 35.50 | 53 | 9.50 | 84.84 | 11.15 | 0.92 | 0.06 | 121 | 21.50 | 442 | 78.50 |
| | | men | 802 | 58.70 | 11.10 | 26.00 | 3.30 | 320 | 40.00 | 388 | 48.50 | 92 | 11.50 | 95.09 | 11.25 | 0.82 | 0.08 | 269 | 33.50 | 533 | 66.50 |
| InCHIANTI | EU | women | 614 | 68.13 | 15.11 | 27.35 | 4.68 | 210 | 34.20 | 241 | 39.25 | 163 | 26.55 | 88.82 | 11.81 | 0.87 | 0.07 | 83 | 13.52 | 531 | 86.48 |
| | | men | 498 | 66.72 | 15.14 | 27.06 | 3.39 | 148 | 29.70 | 247 | 49.60 | 103 | 20.68 | 94.54 | 9.34 | 0.95 | 0.06 | 126 | 25.30 | 372 | 74.70 |
| KORA S3 | EU | women | 829 | 52.13 | 10.05 | 26.98 | 4.64 | 307 | 37.00 | 334 | 40.30 | 188 | 22.70 | 83.75 | 11.03 | 0.80 | 0.06 | 698 | 84.20 | 131 | 15.80 |
| | | men | 812 | 52.95 | 10.10 | 27.70 | 3.45 | 160 | 19.70 | 484 | 59.60 | 168 | 20.70 | 97.07 | 9.72 | 0.93 | 0.06 | 650 | 80.00 | 162 | 20.00 |
| KORA S4 | EU | women | 928 | 53.62 | 8.79 | 27.49 | 5.07 | 335 | 36.10 | 338 | 36.40 | 254 | 27.40 | 86.64 | 12.16 | 0.82 | 0.06 | 767 | 82.70 | 161 | 17.30 |
| | | men | 883 | 54.22 | 8.92 | 27.99 | 3.91 | 167 | 18.90 | 493 | 55.80 | 223 | 25.30 | 99.01 | 10.22 | 0.94 | 0.06 | 677 | 76.70 | 206 | 23.30 |
| LIFELINES | EU | women | 4,605 | 47.10 | 11.06 | 25.99 | 4.70 | 2,200 | 48.10 | 1,612 | 35.30 | 760 | 16.60 | 87.85 | 12.26 | 0.87 | 0.07 | 1,044 | 23.00 | 3,496 | 77.00 |
| | | men | 3,452 | 47.58 | 11.32 | 26.66 | 3.66 | 1,172 | 34.00 | 1,728 | 50.20 | 543 | 15.80 | 96.35 | 10.71 | 0.96 | 0.07 | 930 | 27.20 | 2,486 | 72.80 |

| | | | | | | | | | | | | | | | | | | | | | |
|---|---|---|---|---|---|---|---|---|---|---|---|---|---|---|---|---|---|---|---|---|---|
| LOLIPOP_EW610 | EU | women | 249 | 55.66 | 9.70 | 26.65 | 5.10 | 110 | 44.20 | 86 | 34.50 | 53 | 21.30 | 88.32 | 13.60 | 0.86 | 0.09 | 29 | 11.60 | 220 | 88.40 |
| | | men | 678 | 56.07 | 9.84 | 27.80 | 4.44 | 186 | 27.40 | 325 | 47.90 | 167 | 24.60 | 98.42 | 11.61 | 0.94 | 0.06 | 125 | 18.40 | 553 | 81.60 |
| LOLIPOP_IA317 | AS | men | 2,121 | 48.26 | 10.47 | 26.83 | 4.31 | 735 | 34.70 | 977 | 46.10 | 409 | 19.30 | 97.07 | 11.68 | 0.96 | 0.07 | 344 | 16.20 | 1,777 | 83.80 |
| LOLIPOP IA610 CASES | AS | women | 412 | 61.65 | 9.00 | 29.89 | 5.69 | 78 | 19.00 | 157 | 38.20 | 176 | 42.80 | 100.50 | 12.53 | 0.95 | 0.08 | 3 | 0.70 | 409 | 99.30 |
| | | men | 2,011 | 58.71 | 9.68 | 27.14 | 4.13 | 628 | 31.30 | 973 | 48.40 | 407 | 20.30 | 99.02 | 11.20 | 0.98 | 0.07 | 192 | 9.50 | 1,819 | 90.50 |
| LOLIPOP IA610 CONTROLS | | women | 618 | 53.65 | 9.25 | 27.97 | 4.90 | 164 | 26.50 | 282 | 45.60 | 172 | 27.80 | 94.61 | 12.19 | 0.91 | 0.09 | 7 | 1.10 | 611 | 98.90 |
| | | men | 3,516 | 53.04 | 10.64 | 26.68 | 4.00 | 1,166 | 33.20 | 1,708 | 48.60 | 638 | 18.20 | 97.21 | 10.68 | 0.96 | 0.07 | 421 | 12.00 | 3,095 | 88.00 |
| LURIC-Cases | EU | women | 592 | 66.40 | 9.72 | 27.20 | 4.58 | 190 | 32.10 | 243 | 41.00 | 153 | 25.80 | 94.50 | 13.00 | 0.91 | 0.08 | 99 | 16.70 | 493 | 83.30 |
| | | men | 1,772 | 63.10 | 9.75 | 27.60 | 3.69 | 467 | 26.40 | 907 | 51.20 | 396 | 22.30 | 101.10 | 10.60 | 0.99 | 0.06 | 449 | 25.30 | 1,323 | 74.70 |
| LURIC-Controls | EU | women | 296 | 62.10 | 10.80 | 27.40 | 4.88 | 97 | 32.80 | 129 | 43.60 | 70 | 23.60 | 93.40 | 12.60 | 0.89 | 0.07 | 49 | 16.60 | 247 | 83.40 |
| | | men | 324 | 54.70 | 12.60 | 27.40 | 3.75 | 89 | 27.50 | 171 | 52.80 | 64 | 19.80 | 99.50 | 11.00 | 0.97 | 0.07 | 87 | 26.90 | 237 | 73.10 |
| MESA | EU | women | 1,251 | 62.61 | 10.33 | 27.45 | 5.78 | 492 | 39.33 | 418 | 33.41 | 334 | 26.70 | 94.93 | 16.19 | 0.88 | 0.09 | 148 | 11.83 | 1,103 | 88.17 |
| | | men | 1,148 | 62.83 | 10.11 | 27.98 | 4.10 | 279 | 24.30 | 547 | 47.65 | 321 | 27.96 | 101.15 | 10.97 | 0.96 | 0.06 | 127 | 11.06 | 1,021 | 88.94 |
| MrOS SWEDEN | EU | men | 962 | 75.26 | 3.20 | 26.20 | 3.50 | 372 | 38.60 | 472 | 49.10 | 118 | 12.30 | NA | NA | NA | NA | 77 | 8.00 | 885 | 92.00 |
| NETHERLANDS TWIN REGISTER | EU | women | 2,017 | 44.60 | 14.30 | 25.01 | 4.46 | 1,163 | 57.70 | 604 | 29.90 | 250 | 12.40 | 83.39 | 12.04 | 0.80 | 0.08 | 360 | 17.80 | 1,648 | 81.70 |
| | | men | 1,314 | 50.80 | 14.80 | 25.90 | 3.50 | 559 | 42.50 | 581 | 44.20 | 174 | 13.20 | 94.00 | 11.40 | 0.90 | 0.08 | 317 | 24.10 | 994 | 75.60 |
| NFBC66 | EU | Overall | 4984 | 31.00 | 0.00 | 24.66 | 4.27 | 2,989 | 59.97 | 1,528 | 30.66 | 467 | 9.37 | 83.72 | 12.13 | 0.86 | 0.09 | 2,095 | 42.04 | 2,889 | 57.97 |
| | | women | 2608 | 31.00 | 0.00 | 24.17 | 4.73 | 1,776 | 68.10 | 571 | 21.89 | 261 | 10.01 | 78.75 | 12.00 | 0.81 | 0.08 | 938 | 35.97 | 1,670 | 64.03 |
| | | men | 2376 | 31.00 | 0.00 | 25.20 | 3.62 | 1,213 | 51.05 | 957 | 40.28 | 206 | 8.67 | 88.89 | 9.95 | 0.91 | 0.06 | 1,157 | 48.70 | 1,219 | 51.31 |
| NHS-case | EU | women | 1,127 | 53.57 | 6.67 | 29.63 | 5.59 | 283 | 21.50 | 467 | 35.60 | 562 | 42.80 | 89.89 | 13.05 | 0.83 | 0.14 | 158 | 21.10 | 590 | 78.90 |
| NHS-control | | women | 1,124 | 53.24 | 6.69 | 25.09 | 4.70 | 915 | 58.00 | 465 | 29.50 | 197 | 12.50 | 78.59 | 11.12 | 0.77 | 0.07 | 130 | 16.20 | 673 | 83.80 |
| ORCADES | EU | Overall | 889 | 53.49 | 15.73 | 27.67 | 4.84 | 257 | 29.75 | 377 | 43.63 | 230 | 26.62 | 93.51 | 13.88 | 0.91 | 0.09 | 373 | 42.73 | 500 | 57.27 |
| | | women | 485 | 52.71 | 15.71 | 27.34 | 5.31 | 177 | 37.50 | 175 | 37.08 | 120 | 25.42 | 89.00 | 13.60 | 0.86 | 0.08 | 168 | 35.22 | 309 | 64.78 |
| | | men | 404 | 54.43 | 15.73 | 28.07 | 4.19 | 80 | 20.41 | 202 | 51.53 | 110 | 28.06 | 98.92 | 12.19 | 0.96 | 0.07 | 205 | 51.77 | 191 | 48.23 |
| PIVUS | EU | Overall | 948 | 70.20 | 0.17 | 27.05 | 4.35 | 310 | 32.70 | 428 | 45.15 | 210 | 22.15 | 91.22 | 11.60 | 0.90 | 0.07 | 104 | 10.97 | 844 | 89.03 |
| | | women | 475 | 70.26 | 0.15 | 27.10 | 4.90 | 170 | 35.79 | 182 | 38.32 | 123 | 25.89 | 87.77 | 11.68 | 0.86 | 0.06 | 57 | 12.00 | 418 | 88.00 |
| | | men | 473 | 70.13 | 0.17 | 27.00 | 3.73 | 140 | 29.59 | 246 | 52.00 | 87 | 18.39 | 94.68 | 10.46 | 0.94 | 0.06 | 47 | 9.93 | 426 | 90.07 |
| PREVEND | EU | women | 1,754 | 48.23 | 12.03 | 25.82 | 4.77 | 863 | 49.20 | 583 | 33.24 | 293 | 16.70 | 83.34 | 12.99 | 0.82 | 0.08 | 622 | 35.50 | 1,132 | 64.54 |
| | | men | 1,870 | 50.93 | 12.77 | 26.41 | 3.68 | 704 | 37.60 | 878 | 46.95 | 285 | 15.24 | 94.50 | 11.17 | 0.94 | 0.75 | 659 | 35.24 | 1,209 | 64.65 |
| PROSPER | EU | Overall | 5,244 | 75.34 | 3.35 | 26.82 | 4.18 | 1,748 | 33.30 | 2,378 | 45.30 | 1,073 | 20.50 | NA | NA | NA | NA | 1,392 | 26.50 | 3,852 | 73.50 |
| | | women | 2,720 | 75.66 | 3.40 | 27.07 | 4.66 | 899 | 33.10 | 1,122 | 41.30 | 668 | 24.60 | NA | NA | NA | NA | 578 | 21.30 | 2,142 | 78.80 |
| | | men | 2,524 | 75.00 | 3.27 | 26.55 | 3.58 | 849 | 33.60 | 1,256 | 49.80 | 405 | 16.00 | NA | NA | NA | NA | 814 | 32.30 | 1,710 | 67.70 |
| QFS | EU | Overall | 853 | 41.57 | 15.46 | 27.79 | 7.73 | 382 | 44.80 | 237 | 27.80 | 234 | 27.40 | 88.94 | 18.40 | 0.85 | 0.10 | 178 | 20.90 | 675 | 79.10 |
| | | women | 481 | 41.16 | 15.07 | 28.04 | 8.59 | 229 | 47.60 | 108 | 22.50 | 144 | 29.90 | 84.89 | 18.75 | 0.80 | 0.08 | 100 | 20.80 | 381 | 79.20 |
| | | men | 372 | 42.09 | 15.95 | 27.47 | 6.45 | 153 | 41.10 | 129 | 34.70 | 90 | 24.20 | 94.17 | 16.64 | 0.93 | 0.08 | 78 | 21.00 | 294 | 79.00 |
| ROTTERDAM STUDY I | EU | women | 3,547 | 70.33 | 9.60 | 26.73 | 4.06 | 1,230 | 36.50 | 1,494 | 44.30 | 649 | 19.20 | 87.90 | 11.50 | 0.87 | 0.09 | 623 | 18.20 | 2,804 | 81.80 |
| | | men | 2,427 | 68.13 | 8.16 | 25.68 | 2.99 | 990 | 41.70 | 1,208 | 50.90 | 174 | 7.30 | 94.33 | 9.52 | 0.96 | 0.07 | 716 | 30.00 | 1,669 | 70.00 |
| ROTTERDAM STUDY II | EU | women | 1,173 | 65.02 | 8.30 | 27.50 | 4.45 | 361 | 30.90 | 525 | 44.90 | 283 | 24.20 | 89.85 | 11.58 | 0.86 | 0.08 | 237 | 20.20 | 934 | 79.80 |
| | | men | 984 | 64.47 | 7.59 | 26.92 | 3.36 | 285 | 29.00 | 543 | 55.20 | 155 | 15.80 | 99.03 | 10.20 | 0.97 | 0.07 | 182 | 18.50 | 801 | 81.50 |
| ROTTERDAM STUDY III | EU | women | 1,166 | 56.15 | 6.07 | 27.47 | 5.09 | 399 | 34.30 | 477 | 41.00 | 286 | 24.60 | 89.58 | 12.88 | 0.83 | 0.07 | 269 | 23.10 | 894 | 76.90 |
| | | men | 912 | 55.89 | 5.47 | 28.03 | 4.09 | 207 | 22.80 | 474 | 52.30 | 226 | 24.90 | 99.94 | 11.70 | 0.93 | 0.08 | 198 | 21.80 | 712 | 78.20 |
| SARDINIA | EU | women | 552 | 54.97 | 11.67 | 27.28 | 5.08 | 201 | 36.41 | 205 | 37.14 | 146 | 26.44 | 87.18 | 13.16 | 0.86 | 0.09 | 54 | 9.78 | 498 | 90.22 |
| | | men | 478 | 59.13 | 12.53 | 27.89 | 4.01 | 97 | 20.29 | 247 | 51.67 | 134 | 28.03 | 96.42 | 9.92 | 0.97 | 0.06 | 85 | 17.78 | 393 | 82.22 |
| SHIP | EU | women | 2,057 | 48.56 | 16.02 | 26.92 | 5.31 | 846 | 41.10 | 660 | 32.10 | 533 | 25.90 | 83.15 | 12.99 | 0.80 | 0.06 | 560 | 27.20 | 1,497 | 72.80 |
| | | men | 1,994 | 50.78 | 16.41 | 27.68 | 4.05 | 512 | 25.70 | 981 | 49.20 | 501 | 25.10 | 95.78 | 11.67 | 0.93 | 0.06 | 672 | 33.70 | 1,322 | 66.30 |
| SPT | JAMAICA | women | 553 | 45.59 | 13.06 | 28.41 | 6.06 | 157 | 28.39 | 193 | 34.90 | 203 | 36.70 | 84.24 | 12.47 | 1.00 | 0.00 | 66 | 11.93 | 487 | 88.07 |
| THISEAS_CASES | EU | women | 62 | 65.78 | 10.35 | 28.29 | 4.30 | 15 | 24.19 | 33 | 53.23 | 14 | 22.58 | 97.01 | 12.69 | 0.90 | 0.07 | 21 | 33.87 | 41 | 66.13 |
| | | men | 288 | 61.03 | 10.05 | 27.79 | 3.87 | 67 | 23.26 | 150 | 52.08 | 71 | 24.65 | 99.96 | 11.44 | 0.98 | 0.07 | 119 | 41.32 | 169 | 58.68 |
| THISEAS_CONTROLS | | women | 224 | 55.66 | 14.40 | 27.97 | 5.10 | 68 | 30.36 | 89 | 39.73 | 67 | 29.91 | 90.38 | 13.70 | 0.87 | 0.10 | 44 | 19.64 | 180 | 80.36 |
| | | men | 204 | 52.25 | 12.87 | 29.08 | 4.15 | 30 | 14.71 | 95 | 46.57 | 79 | 38.73 | 102.04 | 10.42 | 0.98 | 0.08 | 62 | 30.39 | 142 | 69.61 |
| TRAILS | EU | women | 609 | 19.13 | 0.57 | 23.09 | 3.80 | 451 | 74.10 | 108 | 17.70 | 35 | 5.70 | 75.47 | 8.92 | 0.75 | 0.05 | 212 | 34.80 | 397 | 65.20 |

| | | | | | | | | | | | | | | | | | | | | | |
|---|---|---|---|---|---|---|---|---|---|---|---|---|---|---|---|---|---|---|---|---|---|
| TRAILS | EU | men | 532 | 19.19 | 0.54 | 22.61 | 3.77 | 416 | 78.20 | 65 | 12.20 | 30 | 5.60 | 81.73 | 9.70 | 0.82 | 0.05 | 205 | 38.50 | 326 | 61.50 |
| TWINSUK | EU | women | 2,888 | 47.34 | 12.70 | 25.18 | 4.68 | 1,639 | 56.75 | 858 | 29.77 | 391 | 13.54 | 79.15 | 10.54 | 0.78 | 0.06 | 1,263 | 43.73 | 1,625 | 56.27 |
| WGHS | EU | women | 22,870 | 54.69 | 7.11 | 25.91 | 4.96 | 11,695 | 51.10 | 7,056 | 30.90 | 3,996 | 17.50 | 88.87 | 14.33 | 0.83 | 0.08 | 2,648 | 11.60 | 20,222 | 88.40 |
| WHI Pilot | AF | women | 1,998 | 61.00 | 6.88 | 31.36 | 6.81 | 323 | 16.17 | 660 | 33.03 | 1,015 | 50.80 | 91.82 | 14.00 | 0.82 | 0.07 | 220 | 11.01 | 1,778 | 88.99 |
| WHIYR3/YR4 | AF | women | 3,180 | 61.20 | 7.30 | 31.50 | 6.75 | 484 | 15.22 | 993 | 31.23 | 1,703 | 53.55 | 92.59 | 14.37 | 0.83 | 0.09 | 372 | 11.70 | 2,808 | 88.30 |
| YFS | EU | women | 1,067 | 37.79 | 4.97 | 25.29 | 4.96 | 588 | 55.10 | 299 | 28.00 | 169 | 15.80 | 83.50 | 12.48 | 0.83 | 0.07 | 160 | 15.00 | 907 | 85.00 |
| | | men | 900 | 37.69 | 5.04 | 26.75 | 4.29 | 327 | 36.30 | 405 | 45.00 | 166 | 18.40 | 94.44 | 12.01 | 0.94 | 0.07 | 206 | 22.90 | 694 | 77.10 |

**METBOCHIP Data**

| Study | Ancestry | Groups | N | Age | | BMI (kg/m²) | | BMI Categories* | | | | | | Waist (cm) | | WHR (ratio) | | Current Smoking Status | | | |
|---|---|---|---|---|---|---|---|---|---|---|---|---|---|---|---|---|---|---|---|---|---|
| | | | | | | | | Normal weight | | Overweight | | Obese | | | | | | Smoker | | Non-Smoker | |
| | | | | mean | sd | mean | sd | n | % | n | % | n | % | mean | sd | mean | sd | n | % | n | % |
| DESIR | EU | Overall | 4,263 | 46.26 | 10.21 | 25.23 | 3.86 | 2,182 | 51.22 | 1,611 | 37.82 | 467 | 10.96 | 84.91 | 11.62 | 0.87 | 0.09 | 1,035 | 24.28 | 3,228 | 75.72 |
| | | women | 1,968 | 46.38 | 10.32 | 24.79 | 4.29 | 1,160 | 59.00 | 565 | 28.74 | 241 | 12.26 | 78.87 | 10.72 | 0.80 | 0.07 | 362 | 18.39 | 1,606 | 81.61 |
| | | men | 2,295 | 46.15 | 10.12 | 25.61 | 3.40 | 1,022 | 44.55 | 1,046 | 45.60 | 226 | 9.85 | 90.08 | 9.70 | 0.92 | 0.06 | 673 | 29.32 | 1,622 | 70.68 |
| DR's EXTRA | EU | women | 621 | 66.47 | 5.25 | 27.57 | 5.04 | 215 | 34.60 | 246 | 39.60 | 160 | 25.80 | 88.71 | 13.10 | 0.87 | 0.07 | 44 | 7.10 | 577 | 92.90 |
| | | men | 546 | 66.50 | 5.44 | 27.37 | 3.74 | 147 | 26.90 | 289 | 52.90 | 110 | 20.20 | 98.17 | 10.69 | 0.99 | 0.05 | 78 | 14.30 | 466 | 85.70 |
| EGCUT | EU | women | 1490 | 59.45 | 12.58 | 28.27 | 6.91 | 573 | 39.19 | 319 | 21.82 | 570 | 38.99 | 87.78 | 17.69 | 0.84 | 0.09 | 387 | 36.48 | 1103 | 76.44 |
| | | men | 1014 | 60.25 | 11.64 | 28.02 | 5.70 | 362 | 35.7 | 288 | 28.40 | 364 | 35.9 | 97.95 | 15.03 | 0.95 | 0.10 | 674 | 63.52 | 340 | 23.56 |
| ELY | EU | women | 366 | 57.27 | 7.57 | 26.20 | 4.44 | 169 | 46.20 | 133 | 36.30 | 64 | 17.50 | 81.89 | 10.96 | 0.80 | 0.08 | 59 | 16.10 | 307 | 83.90 |
| | | men | 262 | 58.07 | 7.82 | 26.82 | 3.09 | 79 | 30.20 | 142 | 54.20 | 41 | 15.60 | 95.60 | 9.36 | 0.96 | 0.07 | 47 | 17.90 | 215 | 82.10 |
| EPIC-non-subcohort | EU | women | 216 | 62.68 | 8.50 | 28.95 | 4.61 | 41 | 19.00 | 100 | 46.30 | 75 | 34.70 | 90.47 | 11.63 | 0.84 | 0.06 | 31 | 14.40 | 185 | 85.60 |
| | | men | 321 | 61.92 | 8.39 | 28.38 | 3.57 | 35 | 10.90 | 218 | 67.90 | 68 | 21.20 | 101.92 | 9.72 | 0.97 | 0.05 | 36 | 11.20 | 285 | 88.80 |
| EPIC-subcohort | | women | 474 | 58.92 | 9.59 | 25.64 | 3.78 | 227 | 47.90 | 195 | 41.10 | 52 | 11.00 | 81.00 | 10.10 | 0.79 | 0.06 | 67 | 14.10 | 407 | 85.90 |
| | | men | 339 | 59.45 | 9.07 | 25.99 | 2.96 | 131 | 38.60 | 182 | 53.70 | 26 | 7.70 | 93.93 | 9.12 | 0.92 | 0.06 | 39 | 11.50 | 300 | 88.50 |
| FENLAND | EU | women | 1,542 | 47.01 | 7.01 | 26.47 | 5.54 | 740 | 48.00 | 485 | 31.50 | 317 | 20.60 | 85.18 | 12.87 | 0.82 | 0.07 | 194 | 12.60 | 1,348 | 87.40 |
| | | men | 1,368 | 46.98 | 7.17 | 27.04 | 4.08 | 453 | 33.10 | 642 | 46.90 | 273 | 20.00 | 96.50 | 11.20 | 0.93 | 0.07 | 197 | 14.40 | 1,171 | 85.60 |
| FUSION2-controls | EU | women | 47 | 39.09 | 9.24 | 26.56 | 5.99 | 23 | 48.90 | 12 | 25.50 | 12 | 25.50 | 84.72 | 15.24 | 0.81 | 0.08 | 30 | 63.80 | 17 | 36.20 |
| | | men | 43 | 41.29 | 13.98 | 26.47 | 4.03 | 14 | 32.60 | 24 | 55.80 | 5 | 11.60 | 95.21 | 11.11 | 0.92 | 0.07 | 29 | 67.40 | 14 | 32.60 |
| FUSION2-T2D cases | | women | 43 | 57.12 | 10.45 | 30.96 | 6.82 | 8 | 18.60 | 13 | 30.20 | 22 | 51.20 | 99.62 | 16.00 | 0.90 | 0.06 | 20 | 46.50 | 23 | 53.50 |
| | | men | 109 | 58.63 | 8.04 | 31.29 | 5.60 | 14 | 12.80 | 35 | 32.10 | 60 | 55.10 | 107.91 | 14.50 | 1.00 | 0.07 | 43 | 39.40 | 66 | 60.60 |
| GLACIER | EU | women | 3,619 | 49.27 | 8.82 | 25.56 | 4.38 | 1,869 | 51.80 | 1,219 | 33.80 | 517 | 14.30 | 86.56 | 11.73 | NA | NA | 861 | 23.80 | 2,758 | 76.20 |
| | | men | 2,341 | 50.07 | 8.33 | 26.09 | 3.42 | 927 | 39.60 | 1,146 | 49.00 | 265 | 11.30 | 96.83 | 10.07 | NA | NA | 496 | 21.20 | 1,845 | 78.80 |
| GXE | JAMAICA | women | 469 | 40.02 | 8.13 | 32.33 | 7.47 | 101 | 21.54 | 0 | 0.00 | 368 | 78.47 | 88.78 | 15.31 | 0.80 | 0.07 | 20 | 0.04 | 449 | 95.73 |
| | | men | 140 | 38.67 | 8.90 | 27.69 | 6.94 | 66 | 47.14 | 0 | 0.00 | 74 | 52.86 | 87.72 | 17.91 | 0.85 | 0.08 | 31 | 22.14 | 109 | 77.85 |
| HEALTH2006 | EU | women | 1,580 | 48.83 | 12.97 | 25.32 | 5.02 | 860 | 54.40 | 454 | 28.70 | 236 | 14.90 | 83.23 | 12.58 | 0.82 | 0.07 | 378 | 23.90 | 1,202 | 76.10 |
| | | men | 1,304 | 50.43 | 12.76 | 26.60 | 4.16 | 496 | 38.00 | 581 | 44.60 | 225 | 17.30 | 95.29 | 12.06 | 0.94 | 0.07 | 281 | 21.50 | 1,023 | 78.50 |
| HUNT-controls | EU | women | 319 | 68.03 | 13.56 | 27.65 | 4.56 | 92 | 28.80 | 130 | 40.80 | 97 | 30.40 | 86.72 | 11.11 | 0.83 | 0.06 | 84 | 26.30 | 235 | 73.70 |
| | | men | 368 | 63.58 | 14.58 | 26.53 | 3.30 | 120 | 32.60 | 202 | 54.90 | 46 | 12.50 | 93.38 | 8.69 | 0.92 | 0.06 | 106 | 28.80 | 262 | 71.20 |
| HUNT-T2D cases | | women | 238 | 70.42 | 11.92 | 30.11 | 5.15 | 37 | 15.50 | 83 | 34.90 | 118 | 49.60 | 93.94 | 11.97 | 0.86 | 0.06 | 24 | 10.10 | 214 | 89.90 |
| | | men | 277 | 66.90 | 11.08 | 28.12 | 3.79 | 61 | 22.00 | 133 | 48.00 | 83 | 30.00 | 98.60 | 9.61 | 0.95 | 0.06 | 58 | 20.90 | 219 | 79.10 |
| IMPROVE | EU | women | 1,777 | 64.38 | 5.45 | 27.10 | 4.76 | 651 | 36.60 | 702 | 39.50 | 424 | 23.90 | 89.61 | 12.44 | 0.87 | 0.07 | 238 | 13.40 | 1,539 | 86.60 |
| | | men | 1,653 | 64.04 | 5.35 | 27.39 | 3.64 | 420 | 25.40 | 900 | 54.50 | 332 | 20.00 | 98.70 | 10.81 | 0.97 | 0.07 | 274 | 16.60 | 1,379 | 83.40 |
| INTER99 | EU | women | 3,036 | 45.94 | 7.90 | 25.76 | 5.07 | 1,582 | 52.10 | 919 | 30.30 | 505 | 16.60 | 80.30 | 12.33 | 0.80 | 0.06 | 1,075 | 35.40 | 1,961 | 64.60 |
| | | men | 2,907 | 46.66 | 7.72 | 26.82 | 4.02 | 996 | 34.30 | 1,403 | 48.20 | 503 | 17.30 | 93.26 | 11.04 | 0.92 | 0.06 | 1,070 | 36.80 | 1,837 | 63.20 |
| KORAS3 | EU | women | 659 | 40.89 | 12.66 | 25.18 | 4.72 | 387 | 58.70 | 173 | 26.30 | 98 | 14.90 | 79.12 | 11.24 | 0.78 | 0.06 | 473 | 71.80 | 186 | 28.20 |
| | | men | 599 | 40.58 | 13.14 | 26.54 | 3.43 | 207 | 34.60 | 305 | 50.90 | 87 | 14.50 | 92.80 | 9.33 | 0.90 | 0.06 | 392 | 65.40 | 207 | 34.60 |
| KORAS4 | EU | women | 624 | 41.35 | 14.82 | 25.94 | 5.09 | 294 | 47.10 | 223 | 35.70 | 107 | 17.10 | 82.16 | 12.00 | 0.79 | 0.06 | 476 | 76.30 | 148 | 23.70 |
| | | men | 582 | 42.49 | 15.74 | 26.64 | 3.59 | 197 | 33.80 | 295 | 50.70 | 90 | 15.50 | 94.34 | 10.00 | 0.90 | 0.06 | 389 | 66.80 | 193 | 33.20 |
| MEC-Diabetes cases | AF | Overall | 384 | 68.72 | 8.27 | 31.53 | 6.63 | 48 | 12.50 | 135 | 35.16 | 200 | 52.08 | 103.58 | 15.09 | 0.91 | 0.10 | 70 | 18.23 | 314 | 81.77 |
| | | women | 278 | 68.03 | 8.22 | 32.18 | 6.94 | 35 | 12.59 | 88 | 31.65 | 154 | 55.40 | 102.94 | 15.55 | 0.89 | 0.10 | 51 | 18.35 | 227 | 81.65 |

| | | | | | | | | | | | | | | | | | | | | | |
|---|---|---|---|---|---|---|---|---|---|---|---|---|---|---|---|---|---|---|---|---|---|
| | | men | 106 | 70.52 | 8.15 | 29.80 | 5.41 | 13 | 12.26 | 47 | 44.34 | 46 | 43.40 | 105.26 | 13.72 | 0.96 | 0.08 | 19 | 17.92 | 87 | 82.08 |
| MEC-controls | AF | Overall | 1,284 | 69.37 | 8.6 | 28.59 | 6.36 | 367 | 28.58 | 492 | 38.32 | 414 | 32.24 | 95.03 | 14.86 | 0.87 | 0.11 | 158 | 12.31 | 1,126 | 87.69 |
| | | women | 1,026 | 68.90 | 8.68 | 29.01 | 6.61 | 280 | 27.29 | 376 | 36.65 | 362 | 35.28 | 94.42 | 15.29 | 0.86 | 0.11 | 116 | 11.31 | 910 | 88.69 |
| | | men | 258 | 71.26 | 7.97 | 26.91 | 4.90 | 87 | 33.72 | 116 | 44.96 | 52 | 20.16 | 97.37 | 12.82 | 0.94 | 0.09 | 42 | 16.28 | 216 | 83.72 |
| METSIM-controls | EU | men | 899 | 53.94 | 4.99 | 26.40 | 3.39 | 313 | 34.80 | 468 | 52.10 | 118 | 13.10 | 95.83 | 9.70 | 0.95 | 0.06 | 145 | 16.10 | 754 | 83.90 |
| METSIM-T2D cases | | men | 1,143 | 60.53 | 6.63 | 30.17 | 5.15 | 150 | 13.10 | 480 | 42.00 | 513 | 44.90 | 107.14 | 13.33 | 1.02 | 0.07 | 181 | 15.80 | 962 | 84.20 |
| NSHD | EU | women | 1,213 | 53.00 | 0.00 | 27.35 | 5.23 | 454 | 37.40 | 459 | 37.80 | 300 | 24.70 | 85.59 | 12.53 | 0.81 | 0.07 | 295 | 24.30 | 918 | 75.70 |
| | | men | 1,216 | 53.00 | 0.00 | 27.36 | 3.93 | 340 | 28.00 | 612 | 50.30 | 264 | 21.70 | 97.66 | 10.44 | 0.94 | 0.06 | 298 | 24.50 | 918 | 75.50 |
| SCARSHEEP cases | EU | women | 402 | 60.03 | 7.35 | 27.02 | 4.97 | 148 | 36.90 | 149 | 37.20 | 104 | 25.90 | 90.30 | 13.52 | 0.87 | 0.08 | 142 | 35.30 | 260 | 64.70 |
| | | men | 1,120 | 56.76 | 7.13 | 26.92 | 3.63 | 341 | 30.70 | 569 | 51.30 | 199 | 17.90 | 99.00 | 9.92 | 0.97 | 0.06 | 442 | 39.50 | 678 | 60.50 |
| SCARSHEEP controls | EU | women | 554 | 60.72 | 7.07 | 25.57 | 4.06 | 275 | 50.20 | 199 | 36.30 | 74 | 13.50 | 86.32 | 11.86 | 0.85 | 0.09 | 176 | 31.80 | 378 | 68.20 |
| | | men | 1,334 | 57.55 | 7.05 | 25.86 | 3.48 | 567 | 42.60 | 612 | 46.00 | 151 | 11.40 | 95.98 | 9.77 | 0.96 | 0.07 | 371 | 27.80 | 963 | 72.20 |
| SPT | JAMAICA | women | 553 | 45.59 | 13.06 | 28.41 | 6.06 | 157 | 28.39 | 193 | 34.90 | 203 | 36.70 | 84.24 | 12.47 | 1.00 | 0.00 | 66 | 11.93 | 487 | 88.07 |
| | | men | 351 | 47.95 | 14.81 | 24.29 | 4.34 | 251 | 71.50 | 103 | 29.34 | 33 | 9.40 | 82.77 | 12.53 | 0.85 | 0.88 | 116 | 33.05 | 235 | 66.95 |
| WHITEHALL | EU | women | 708 | 61.01 | 6.12 | 27.12 | 5.63 | 277 | 39.10 | 255 | 36.00 | 176 | 24.90 | 83.69 | 13.22 | 0.81 | 0.07 | 69 | 9.70 | 639 | 90.30 |
| | | men | 2,345 | 60.64 | 5.91 | 26.62 | 3.67 | 793 | 33.80 | 1,179 | 50.30 | 373 | 15.90 | 94.35 | 10.49 | 0.94 | 0.07 | 152 | 6.50 | 2,193 | 93.50 |
| **VALIDATION Data** | | | | | | | | | | | | | | | | | | | | | |
| **UKBB** | **EU** | **combined** | 118,634 | 56.93 | 7.93 | 27.5362 | 4.8278 | 37,551 | 31.7 | 50,468 | 42.5 | 29,680 | 25 | 90.81 | 13.6 | 0.8750 | 0.0906 | 13,416 | 11.3 | 105,218 | 88.69 |

* BMI categories will not add up to the total sample size (N) in studies that excluded underweight (BMI<18.5) individuals.

**Supplementary Table 4**. Study-specific descriptives from contributing studies stratified by smoking status. Abbreviations: BMI- body mass index, WHR- waist to hip ratio.

| GWAS Data | | | | | | | | | | | | | | | | | | | | | | | | | | | | | | | | |
|---|---|---|---|---|---|---|---|---|---|---|---|---|---|---|---|---|---|---|---|---|---|---|---|---|---|---|---|---|---|---|---|---|
| | | | SMOKERS | | | | | | | | | | | | | | | NON-SMOKERS | | | | | | | | | | | | | | |
| Study | Ancestry | Groups | N | Age | | BMI | | BMI Categories* | | | | | | Waist (cm) | | WHR (ratio) | | N | Age | | BMI | | BMI Categories* | | | | | | Waist (cm) | | WHR (ratio) | |
| | | | | | | | | Normal weight | | Overweight | | Obese | | | | | | | | | | | Normal weight | | Overweight | | Obese | | | | | |
| | | | | mean | sd | mean | sd | n | % | n | % | n | % | mean | sd | mean | sd | | mean | sd | mean | sd | n | % | n | % | n | % | mean | sd | mean | sd |
| AE | EU | Overall | 228 | 64.58 | 8.45 | 25.58 | 3.60 | 107 | 46.90 | 101 | 44.30 | 20 | 8.80 | NA | NA | NA | NA | 397 | 69.45 | 8.57 | 26.69 | 3.72 | 133 | 33.50 | 206 | 51.90 | 58 | 14.60 | NA | NA | NA | NA |
| | | women | 80 | 64.75 | 9.15 | 25.17 | 3.99 | 39 | 48.80 | 33 | 41.30 | 8 | 10.00 | NA | NA | NA | NA | 110 | 69.74 | 9.47 | 26.21 | 4.99 | 38 | 34.50 | 49 | 44.50 | 23 | 20.90 | NA | NA | NA | NA |
| | | men | 148 | 64.48 | 8.08 | 25.80 | 3.37 | 68 | 46.00 | 68 | 45.90 | 12 | 8.10 | NA | NA | NA | NA | 287 | 69.34 | 8.21 | 26.51 | 3.08 | 95 | 33.10 | 157 | 54.70 | 35 | 12.20 | NA | NA | NA | NA |
| AGES | EU | Overall | 409 | 74.50 | 4.90 | 25.80 | 4.40 | 167 | 40.83 | 166 | 40.59 | 65 | 15.89 | 98.10 | 12.40 | NA | NA | 2,805 | 76.68 | 5.48 | 27.29 | 4.40 | 841 | 30.22 | 1,299 | 46.68 | 643 | 23.10 | 101.19 | 12.10 | NA | NA |
| | | women | 247 | 74.60 | 4.90 | 25.90 | 4.80 | 98 | 39.68 | 94 | 38.06 | 46 | 18.62 | 97.20 | 13.30 | NA | NA | 1,616 | 76.59 | 5.59 | 27.39 | 4.83 | 504 | 31.54 | 694 | 43.43 | 400 | 25.03 | 99.89 | 13.07 | NA | NA |
| | | men | 162 | 74.50 | 4.90 | 25.70 | 3.80 | 69 | 42.59 | 72 | 44.44 | 19 | 11.73 | 99.50 | 10.70 | NA | NA | 1,189 | 76.80 | 5.31 | 27.16 | 3.75 | 337 | 28.44 | 605 | 51.05 | 243 | 20.51 | 102.96 | 10.38 | NA | NA |
| ARIC | AF | Overall | 781 | 53.00 | 5.70 | 27.80 | 5.50 | 245 | 31.40 | 308 | 39.40 | 228 | 29.20 | 96.10 | 13.80 | 0.92 | 0.07 | 1,837 | 53.45 | 5.85 | 30.48 | 6.13 | 302 | 16.44 | 699 | 38.05 | 836 | 45.51 | 100.68 | 15.30 | 0.92 | 0.08 |
| | | women | 416 | 52.70 | 5.40 | 28.60 | 6.00 | 115 | 27.60 | 154 | 37.00 | 147 | 35.30 | 96.80 | 15.10 | 0.91 | 0.08 | 1,233 | 53.35 | 5.74 | 31.35 | 6.52 | 175 | 14.19 | 427 | 34.63 | 631 | 51.18 | 101.31 | 16.38 | 0.90 | 0.08 |
| | | men | 365 | 53.30 | 5.90 | 26.80 | 4.60 | 130 | 35.60 | 154 | 42.20 | 81 | 22.20 | 95.20 | 12.00 | 0.94 | 0.06 | 604 | 53.65 | 6.05 | 28.71 | 4.80 | 127 | 21.03 | 272 | 45.03 | 205 | 33.94 | 99.41 | 12.76 | 0.95 | 0.05 |
| ARIC | EU | Overall | 1,996 | 53.60 | 5.50 | 25.80 | 4.60 | 951 | 47.70 | 713 | 35.70 | 332 | 16.60 | 94.00 | 13.10 | 0.93 | 0.08 | 5,934 | 54.51 | 5.73 | 27.43 | 4.92 | 1,991 | 33.55 | 2,466 | 41.56 | 1,477 | 24.89 | 96.94 | 13.42 | 0.93 | 0.08 |
| | | women | 1,091 | 53.30 | 5.60 | 25.20 | 4.90 | 617 | 56.60 | 295 | 27.00 | 179 | 16.40 | 90.50 | 13.90 | 0.89 | 0.08 | 3,097 | 54.16 | 5.69 | 27.11 | 5.64 | 1,330 | 42.94 | 972 | 31.39 | 795 | 25.67 | 93.92 | 15.08 | 0.89 | 0.08 |
| | | men | 905 | 53.90 | 5.50 | 26.60 | 4.00 | 334 | 36.90 | 418 | 46.00 | 153 | 16.90 | 98.20 | 10.60 | 0.97 | 0.05 | 2,831 | 54.89 | 5.76 | 27.77 | 3.96 | 661 | 23.30 | 1,494 | 52.66 | 682 | 24.04 | 100.23 | 10.37 | 0.97 | 0.05 |
| AUSTWIN | EU | Overall | 448 | 42.60 | 11.40 | 25.80 | 4.60 | 205 | 45.80 | 166 | 37.10 | 72 | 16.10 | NA | NA | NA | NA | 1,718 | 53.06 | 15.47 | 27.05 | 5.16 | 658 | 38.50 | 663 | 38.80 | 387 | 22.70 | NA | NA | NA | NA |
| BHS | EU | women | 55 | 45.80 | 16.70 | 27.00 | 4.60 | 22 | 40.00 | 19 | 34.50 | 14 | 25.50 | 84.20 | 12.00 | 0.81 | 0.07 | 590 | 55.23 | 16.63 | 25.44 | 4.38 | 316 | 53.60 | 177 | 30.00 | 93 | 15.80 | 80.66 | 11.50 | 0.80 | 0.07 |
| | | men | 54 | 45.10 | 15.50 | 26.20 | 3.90 | 26 | 33.30 | 18 | 33.30 | 10 | 18.50 | 91.80 | 10.50 | 0.93 | 0.07 | 359 | 54.75 | 16.78 | 26.63 | 3.64 | 124 | 34.50 | 171 | 47.60 | 61 | 17.00 | 93.62 | 10.46 | 0.93 | 0.06 |
| BIOME | AF | Overall | 905 | 50.80 | 12.70 | 29.50 | 7.70 | 264 | 29.20 | 266 | 29.40 | 358 | 39.60 | NA | NA | NA | NA | 2,587 | 52.24 | 15.56 | 30.78 | 8.07 | 598 | 23.12 | 787 | 30.42 | 1,170 | 45.23 | NA | NA | NA | NA |
| | | women | 478 | 51.90 | 12.80 | 30.90 | 8.10 | 106 | 22.20 | 126 | 26.40 | 233 | 48.70 | NA | NA | NA | NA | 1,771 | 52.28 | 15.92 | 31.70 | 8.68 | 379 | 21.40 | 479 | 27.05 | 885 | 49.97 | NA | NA | NA | NA |
| | | men | 427 | 49.50 | 12.40 | 27.90 | 6.80 | 158 | 37.00 | 140 | 32.80 | 125 | 29.30 | NA | NA | NA | NA | 816 | 52.14 | 14.77 | 28.78 | 6.10 | 219 | 26.84 | 308 | 37.75 | 285 | 34.93 | NA | NA | NA | NA |
| BIOME | EU | Overall | 168 | 64.40 | 8.00 | 27.60 | 5.50 | 48 | 28.60 | 74 | 44.10 | 44 | 26.20 | NA | NA | NA | NA | 1,849 | 68.07 | 9.34 | 26.85 | 5.43 | 727 | 39.32 | 668 | 36.13 | 435 | 23.53 | NA | NA | NA | NA |
| | | women | 55 | 63.60 | 8.90 | 26.70 | 6.00 | 22 | 40.00 | 17 | 30.90 | 15 | 27.30 | NA | NA | NA | NA | 913 | 67.47 | 9.06 | 25.93 | 5.70 | 441 | 48.30 | 264 | 28.92 | 191 | 20.92 | NA | NA | NA | NA |
| | | men | 113 | 64.70 | 7.50 | 28.00 | 5.30 | 26 | 23.00 | 57 | 50.40 | 29 | 25.70 | NA | NA | NA | NA | 936 | 68.66 | 9.58 | 27.74 | 5.00 | 286 | 30.56 | 404 | 43.16 | 244 | 26.07 | NA | NA | NA | NA |
| BIOME | HS | Overall | 921 | 49.20 | 13.30 | 29.20 | 6.50 | 239 | 26.00 | 315 | 34.20 | 358 | 38.90 | NA | NA | NA | NA | 3,787 | 55.55 | 16.47 | 29.44 | 6.67 | 967 | 25.53 | 1,310 | 34.59 | 1,484 | 39.19 | NA | NA | NA | NA |
| | | women | 475 | 49.10 | 13.50 | 29.90 | 6.80 | 119 | 25.10 | 140 | 29.50 | 212 | 44.60 | NA | NA | NA | NA | 2,469 | 55.19 | 16.62 | 29.70 | 6.98 | 639 | 25.88 | 782 | 31.67 | 1,028 | 41.64 | NA | NA | NA | NA |
| | | men | 446 | 49.40 | 13.20 | 28.60 | 6.10 | 120 | 26.90 | 175 | 39.20 | 146 | 32.70 | NA | NA | NA | NA | 1,318 | 56.22 | 16.15 | 28.96 | 6.01 | 328 | 24.89 | 528 | 40.06 | 456 | 34.60 | NA | NA | NA | NA |
| BLSA | EU | women | NA | NA | NA | NA | NA | NA | NA | NA | NA | NA | NA | NA | NA | NA | NA | 372 | 66.76 | 17.03 | 25.92 | 4.84 | 193 | 51.88 | 109 | 29.30 | 70 | 18.82 | 84.49 | 11.67 | 0.82 | 0.07 |
| | | men | NA | NA | NA | NA | NA | NA | NA | NA | NA | NA | NA | NA | NA | NA | NA | 441 | 71.53 | 15.70 | 27.54 | 4.56 | 139 | 31.52 | 191 | 43.31 | 111 | 25.17 | 98.54 | 12.17 | 0.95 | 0.07 |
| B58C | EU | women | 809 | 45.20 | 0.40 | 26.70 | 5.50 | 366 | 45.20 | 249 | 30.80 | 186 | 23.00 | 85.70 | 13.00 | 0.83 | 0.06 | 2,446 | 45.16 | 0.38 | 27.00 | 5.52 | 1,060 | 43.30 | 814 | 33.30 | 565 | 23.10 | 85.19 | 12.47 | 0.81 | 0.06 |
| | | men | 732 | 45.20 | 0.40 | 27.30 | 4.30 | 230 | 31.40 | 312 | 42.60 | 185 | 25.30 | 97.90 | 11.20 | 0.94 | 0.06 | 2,494 | 45.17 | 0.38 | 28.05 | 4.24 | 561 | 22.50 | 1,282 | 51.40 | 649 | 26.00 | 98.85 | 11.08 | 0.93 | 0.06 |
| CHS | | women | 229 | 70.10 | 3.80 | 25.40 | 4.50 | 127 | 55.50 | 67 | 29.30 | 35 | 15.30 | 89.40 | 12.80 | 0.89 | 0.08 | 1,656 | 72.14 | 5.26 | 26.63 | 4.78 | 681 | 41.12 | 646 | 39.01 | 329 | 19.87 | 91.08 | 13.51 | 0.89 | 0.10 |
| | EU | men | 119 | 71.00 | 4.90 | 25.40 | 3.20 | 54 | 45.40 | 59 | 49.60 | 6 | 5.00 | 96.00 | 9.40 | 0.96 | 0.06 | 1,111 | 73.16 | 5.62 | 26.52 | 3.51 | 391 | 35.19 | 545 | 49.05 | 175 | 15.75 | 97.82 | 9.73 | 0.96 | 0.06 |
| CLHNS | AS | women | 267 | 49.60 | 7.10 | 23.10 | 4.80 | 181 | 67.80 | 59 | 22.10 | 27 | 10.10 | 78.30 | 11.60 | 0.88 | 0.05 | 1,526 | 48.25 | 5.90 | 24.53 | 4.25 | 848 | 55.57 | 522 | 34.21 | 156 | 10.22 | 81.59 | 10.67 | 0.88 | 0.05 |
| COLAUS | EU | women | 699 | 52.04 | 9.87 | 23.98 | 4.34 | 444 | 63.52 | 168 | 24.03 | 87 | 12.45 | 64.05 | 11.84 | 0.65 | 0.07 | 2,176 | 54.49 | 10.91 | 25.55 | 5.01 | 1,146 | 52.67 | 653 | 30.01 | 377 | 17.33 | 67.23 | 13.20 | 0.66 | 0.07 |
| | | men | 734 | 51.18 | 10.39 | 26.23 | 3.77 | 292 | 39.78 | 331 | 45.10 | 111 | 15.12 | 80.35 | 12.68 | 0.79 | 0.08 | 1,826 | 53.64 | 10.84 | 26.79 | 4.10 | 646 | 35.38 | 839 | 45.95 | 341 | 18.67 | 82.00 | 13.48 | 0.79 | 0.08 |
| CROATIA-KORCULA | EU | Overall | 203 | 48.30 | 12.00 | 26.90 | 4.40 | 72 | 35.80 | 89 | 44.30 | 40 | 19.90 | 90.80 | 12.70 | 0.89 | 0.09 | 683 | 58.45 | 13.60 | 28.29 | 4.02 | 133 | 19.85 | 323 | 48.21 | 214 | 31.94 | 95.27 | 11.81 | 0.91 | 0.08 |
| | | women | 140 | 47.70 | 11,14 | 26.40 | 4.40 | 57 | 41.30 | 58 | 42.00 | 23 | 16.70 | 87.80 | 12.00 | 0.86 | 0.08 | 425 | 57.89 | 13.48 | 27.87 | 4.27 | 107 | 25.48 | 190 | 45.24 | 123 | 29.29 | 91.95 | 11.70 | 0.88 | 0.08 |
| | | men | 63 | 49.60 | 13.70 | 27.90 | 4.10 | 15 | 23.80 | 31 | 49.20 | 17 | 26.00 | 97.40 | 11.60 | 0.94 | 0.08 | 258 | 59.36 | 13.76 | 28.99 | 3.46 | 26 | 10.40 | 133 | 53.20 | 91 | 36.40 | 100.84 | 9.73 | 0.96 | 0.07 |
| CROTIA-VIS | EU | Overall | 255 | 47.60 | 14.50 | 26.10 | 4.50 | 105 | 42.20 | 98 | 39.40 | 46 | 18.50 | 92.20 | 12.50 | 0.91 | 0.08 | 660 | 59.69 | 14.57 | 27.78 | 4.11 | 165 | 25.19 | 297 | 45.34 | 193 | 29.47 | 97.21 | 11.24 | 0.94 | 0.08 |
| | | women | 136 | 46.50 | 13.70 | 25.90 | 4.90 | 62 | 47.00 | 43 | 32.60 | 27 | 20.50 | 89.80 | 13.30 | 0.88 | 0.08 | 393 | 60.15 | 15.19 | 27.63 | 4.47 | 117 | 30.08 | 151 | 38.82 | 121 | 31.11 | 95.64 | 12.23 | 0.92 | 0.09 |
| | | men | 119 | 48.80 | 15.40 | 26.30 | 3.90 | 43 | 36.80 | 55 | 47.00 | 19 | 16.20 | 95.10 | 10.90 | 0.95 | 0.07 | 267 | 59.01 | 13.60 | 28.00 | 3.51 | 48 | 18.05 | 146 | 54.89 | 72 | 27.07 | 99.52 | 9.16 | 0.98 | 0.06 |
| DESIR | EU | Overall | 68 | 51.80 | 5.40 | 22.70 | 1.40 | 68 | 100 | 0 | 0 | 0 | 0 | 79.60 | 8.20 | 0.84 | 0.08 | 647 | 49.73 | 8.28 | 21.71 | 1.89 | 647 | 100 | 0 | 0 | 0 | 0 | 73.70 | 8.03 | 0.79 | 0.07 |
| | | women | 31 | 50.20 | 5.00 | 22.60 | 1.50 | 31 | 100 | 0 | 0 | 0 | 0 | 73.60 | 6.60 | 0.78 | 0.07 | 506 | 48.95 | 8.73 | 21.28 | 1.85 | 506 | 100 | 0 | 0 | 0 | 0 | 70.68 | 5.71 | 0.76 | 0.05 |
| | | men | 37 | 53.10 | 5.50 | 22.80 | 1.40 | 37 | 100 | 0 | 0 | 0 | 0 | 84.50 | 5.60 | 0.90 | 0.05 | 141 | 52.53 | 5.64 | 23.24 | 1.08 | 141 | 100 | 0 | 0 | 0 | 0 | 84.56 | 5.34 | 0.89 | 0.04 |
| EGCUT | EU | women | 125 | 43.99 | 16.50 | 25.26 | 4.89 | 67 | 53.60 | 38 | 30.40 | 20 | 16.00 | 83.77 | 12.87 | 0.82 | 0.08 | 958 | 68.87 | 20.29 | 27.59 | 5.63 | 335 | 35.01 | 333 | 34.80 | 287 | 29.99 | 89.36 | 14.18 | 0.85 | 0.09 |
| | | men | 338 | 49.31 | 17.13 | 26.10 | 5.12 | 166 | 49.11 | 113 | 33.43 | 58 | 17.16 | 93.61 | 13.99 | 0.93 | 0.09 | 638 | 60.36 | 18.93 | 27.41 | 4.59 | 201 | 31.55 | 280 | 43.96 | 156 | 24.49 | 97.76 | 13.41 | 0.94 | 0.09 |
| EPIC-Norfolk | EU | women | 138 | 54.90 | 8.40 | 25.80 | 4.10 | 59 | 45.40 | 52 | 40.00 | 19 | 14.60 | 81.60 | 10.50 | 0.79 | 0.06 | 935 | 57.29 | 8.71 | 25.71 | 3.97 | 442 | 47.42 | 364 | 39.06 | 126 | 13.52 | 80.63 | 9.82 | 0.78 | 0.06 |
| | | men | 124 | 57.70 | 9.10 | 25.80 | 3.70 | 52 | 42.30 | 59 | 48.00 | 12 | 9.80 | 94.40 | 10.70 | 0.93 | 0.06 | 789 | 58.68 | 8.96 | 26.46 | 3.19 | 262 | 33.21 | 422 | 53.49 | 105 | 13.31 | 94.90 | 9.32 | 0.92 | 0.06 |
| ERF | EU | women | 591 | 47.30 | 12.90 | 25.70 | 4.60 | 299 | 50.60 | 192 | 32.50 | 95 | 16.10 | 80.40 | 11.60 | 0.81 | 0.08 | 722 | 47.66 | 15.54 | 27.00 | 5.14 | 264 | 36.50 | 285 | 36.50 | 168 | 23.20 | 82.38 | 12.49 | 0.80 | 0.08 |
| | | men | 329 | 48.90 | 13.90 | 26.30 | 4.10 | 124 | 37.70 | 147 | 44.70 | 56 | 17.00 | 92.40 | 11.50 | 0.95 | 0.08 | 680 | 48.23 | 14.46 | 27.51 | 3.83 | 173 | 25.40 | 363 | 53.30 | 144 | 21.10 | 94.20 | 11.38 | 0.94 | 0.08 |
| FAMHS | EU | Overall | 525 | 49.00 | 12.50 | 26.30 | 5.20 | 235 | 45.00 | 197 | 37.70 | 90 | 17.20 | 94.60 | 15.00 | 0.92 | 0.09 | 3,138 | 52.73 | 13.75 | 28.00 | 5.51 | 1,006 | 32.16 | 1,174 | 37.53 | 948 | 30.31 | 98.16 | 15.31 | 0.92 | 0.09 |
| FENLAND | EU | women | 75 | 45.30 | 7.40 | 26.60 | 5.30 | 35 | 46.70 | 21 | 28.00 | 19 | 25.30 | 87.20 | 13.30 | 0.85 | 0.06 | 667 | 45.45 | 7.19 | 26.72 | 5.53 | 303 | 45.40 | 216 | 32.40 | 148 | 22.20 | 87.01 | 12.90 | 0.84 | 0.06 |
| | | men | 65 | 44.40 | 6.70 | 27.00 | 4.10 | 24 | 36.90 | 31 | 47.70 | 10 | 15.40 | 96.60 | 10.30 | 0.94 | 0.06 | 500 | 44.79 | 7.45 | 27.65 | 3.91 | 123 | 24.60 | 266 | 53.20 | 111 | 22.20 | 99.00 | 11.94 | 0.94 | 0.08 |
| FRAMINGHAM HEART STUDY (BMI) | EU | Overall | 2,507 | 36.20 | 8.80 | 25.10 | 4.60 | 1,380 | 55.70 | 772 | 31.10 | 328 | 13.20 | 93.80 | 15.20 | 0.89 | 0.10 | 5,847 | 38.42 | 9.51 | 26.08 | 5.04 | 2,710 | 46.86 | 2,057 | 35.57 | 1,016 | 17.57 | 92.90 | 15.14 | 0.87 | 0.10 |
| | | women | 1,320 | 36.00 | 8.80 | 24.10 | 4.70 | 911 | 70.20 | 255 | 19.70 | 132 | 10.20 | 89.50 | 15.80 | 0.81 | 0.07 | 3,198 | 38.27 | 9.34 | 25.12 | 5.42 | 1,920 | 60.95 | 748 | 23.75 | 482 | 15.30 | 88.18 | 15.67 | 0.80 | 0.07 |
| | | men | 1,187 | 36.40 | 8.80 | 26.30 | 4.10 | 469 | 39.70 | 517 | 43.70 | 196 | 16.60 | 98.20 | 13.30 | 0.97 | 0.06 | 2,649 | 38.60 | 9.70 | 27.23 | 4.27 | 790 | 30.00 | 1,309 | 49.72 | 534 | 20.28 | 98.32 | 12.47 | 0.95 | 0.06 |
| FRAMINGHAM HEART STUDY (WC) | EU | Overall | 1,380 | 45.20 | 10.30 | 26.60 | 5.00 | 685 | 50.30 | 464 | 34.10 | 213 | 15.60 | 91.30 | 14.90 | 0.89 | 0.10 | 5,720 | 45.23 | 11.01 | 26.91 | 5.27 | 2,657 | 46.90 | 1,990 | 35.13 | 1,018 | 19.97 | 91.41 | 14.98 | 0.88 | 0.10 |
| | | women | 713 | 45.50 | 10.20 | 25.80 | 5.40 | 454 | 64.90 | 159 | 22.70 | 87 | 12.40 | 85.10 | 14.40 | 0.82 | 0.07 | 3,032 | 44.94 | 10.92 | 26.07 | 5.91 | 1,864 | 62.32 | 665 | 22.23 | 462 | 15.45 | 85.58 | 15.27 | 0.80 | 0.07 |
| | | men | 667 | 44.90 | 10.40 | 27.50 | 4.40 | 231 | 34.90 | 305 | 46.10 | 126 | 19.00 | 97.90 | 12.20 | 0.97 | 0.06 | 2,688 | 45.57 | 11.10 | 27.85 | 4.26 | 793 | 29.66 | 1,325 | 49.55 | 556 | 20.79 | 97.99 | 11.52 | 0.95 | 0.06 |
| FRAMINGHAM HEART STUDY (WHR) | EU | Overall | 777 | 49.50 | 9.40 | 26.40 | 4.50 | 437 | 57.20 | 256 | 33.50 | 71 | 9.30 | 89.30 | 14.30 | 0.89 | 0.10 | 2,478 | 51.75 | 10.02 | 26.96 | 4.96 | 1,333 | 54.47 | 825 | 33.71 | 289 | 11.81 | 89.47 | 14.55 | 0.88 | 0.10 |
| | | women | 409 | 49.70 | 9.50 | 25.40 | 4.80 | 302 | 75.30 | 74 | 18.50 | 25 | 6.20 | 81.80 | 12.40 | 0.82 | 0.07 | 1,298 | 51.45 | 9.86 | 26.30 | 5.74 | 919 | 71.91 | 246 | 19.25 | 113 | 8.84 | 82.12 | 14.00 | 0.80 | 0.07 |
| | | men | 368 | 49.20 | 9.40 | 27.40 | 4.00 | 135 | 37.20 | 182 | 50.10 | 46 | 12.70 | 97.60 | 11.20 | 0.97 | 0.06 | 1,180 | 52.07 | 10.19 | 27.69 | 3.80 | 414 | 35.41 | 579 | 49.53 | 176 | 15.06 | 97.57 | 10.19 | 0.95 | 0.06 |

| | | | | | | | | | | | | | | | | | | | | | | | | | | | | | | |
|---|---|---|---|---|---|---|---|---|---|---|---|---|---|---|---|---|---|---|---|---|---|---|---|---|---|---|---|---|---|---|
| FUSION-controls | EU | women | 30 | 60.90 | 8.40 | 27.00 | 4.80 | 13 | 43.30 | 9 | 30.00 | 8 | 26.70 | 86.90 | 11.40 | 0.83 | 0.06 | 17 | 60.69 | 5.79 | 26.92 | 2.13 | 4 | 23.50 | 12 | 70.60 | 1 | 5.90 | 86.21 | 8.84 | 0.83 | 0.06 |
| | | men | 24 | 67.50 | 6.50 | 27.40 | 4.20 | 6 | 25.00 | 12 | 50.00 | 6 | 25.00 | 100.50 | 10.30 | 0.96 | 0.05 | 36 | 68.57 | 7.90 | 27.07 | 3.41 | 13 | 36.10 | 18 | 50.00 | 5 | 13.90 | 96.83 | 9.58 | 0.94 | 0.06 |
| FUSION-T2D cases | | women | 25 | 59.90 | 6.90 | 31.60 | 5.80 | 3 | 12.00 | 8 | 32.00 | 14 | 56.00 | 102.30 | 15.70 | 0.90 | 0.06 | 38 | 63.63 | 8.11 | 31.87 | 4.33 | 2 | 5.30 | 9 | 23.70 | 27 | 71.00 | 101.63 | 11.57 | 0.90 | 0.06 |
| | | men | 55 | 59.70 | 7.50 | 28.50 | 4.10 | 10 | 18.20 | 28 | 50.90 | 17 | 30.90 | 103.30 | 12.60 | 0.98 | 0.07 | 206 | 63.68 | 6.84 | 29.55 | 4.05 | 23 | 11.20 | 93 | 45.10 | 90 | 43.70 | 105.13 | 10.43 | 0.98 | 0.06 |
| GENDIAN | EU | Overall | 47 | 56.10 | 9.20 | 31.30 | 6.50 | 6 | 12.80 | 18 | 38.30 | 23 | 48.90 | 109.90 | 14.80 | 0.99 | 0.08 | 402 | 66.05 | 10.00 | 31.63 | 5.79 | 33 | 8.21 | 152 | 37.81 | 217 | 53.98 | 111.30 | 15.09 | 1.00 | 0.13 |
| | | women | 16 | 55.10 | 10.90 | 31.90 | 5.50 | 2 | 12.50 | 4 | 25.00 | 10 | 62.50 | 106.80 | 11.90 | 0.94 | 0.10 | 196 | 67.60 | 9.96 | 32.33 | 6.21 | 13 | 6.63 | 70 | 35.71 | 113 | 57.65 | 110.63 | 16.04 | 0.96 | 0.13 |
| | | men | 31 | 56.60 | 8.30 | 31.00 | 7.10 | 4 | 12.90 | 14 | 45.20 | 13 | 41.90 | 111.50 | 16.10 | 1.02 | 0.05 | 206 | 64.58 | 9.84 | 30.95 | 5.30 | 20 | 9.71 | 82 | 39.81 | 104 | 50.49 | 111.95 | 14.12 | 1.04 | 0.12 |
| GENERATION SCOTLAND | EU | Overall | 1,543 | 47.80 | 13.40 | 26.30 | 5.20 | 440 | 43.70 | 366 | 36.40 | 200 | 19.90 | 89.70 | 13.60 | 0.88 | 0.08 | 8,186 | 52.95 | 13.49 | 27.18 | 5.07 | 1,981 | 35.37 | 2,305 | 41.15 | 1,315 | 23.48 | 90.40 | 13.89 | 0.87 | 0.10 |
| | | women | 855 | 47.50 | 13.30 | 26.40 | 5.80 | 258 | 45.90 | 184 | 32.70 | 120 | 21.40 | 86.70 | 13.80 | 0.84 | 0.07 | 4,858 | 52.62 | 13.39 | 26.94 | 5.56 | 1,356 | 40.62 | 1,200 | 35.95 | 782 | 23.43 | 86.17 | 13.65 | 0.83 | 0.09 |
| | | men | 688 | 48.30 | 13.60 | 26.20 | 4.40 | 182 | 41.00 | 182 | 41.00 | 80 | 18.00 | 93.80 | 12.30 | 0.92 | 0.07 | 3,328 | 53.44 | 13.63 | 27.53 | 4.24 | 625 | 27.62 | 1,105 | 48.83 | 533 | 23.55 | 96.68 | 11.68 | 0.93 | 0.07 |
| GENOA | EU | Overall | 181 | 51.90 | 11.00 | 28.00 | 6.20 | 59 | 32.60 | 69 | 38.10 | 53 | 29.30 | 95.90 | 15.80 | 0.91 | 0.08 | 1,253 | 55.80 | 10.69 | 30.76 | 6.30 | 205 | 16.36 | 452 | 36.07 | 596 | 47.57 | 100.70 | 15.53 | 0.91 | 0.09 |
| | | women | 92 | 51.80 | 11.80 | 27.20 | 7.10 | 41 | 44.60 | 25 | 27.20 | 26 | 28.30 | 90.60 | 17.00 | 0.86 | 0.07 | 700 | 55.46 | 10.62 | 31.00 | 7.10 | 149 | 21.29 | 208 | 29.71 | 343 | 49.00 | 97.02 | 17.06 | 0.86 | 0.09 |
| | | men | 89 | 51.90 | 10.30 | 28.70 | 5.00 | 18 | 20.20 | 44 | 49.40 | 27 | 30.30 | 101.30 | 12.30 | 0.96 | 0.06 | 553 | 56.23 | 10.77 | 30.45 | 5.11 | 56 | 10.13 | 244 | 44.12 | 253 | 45.75 | 105.36 | 11.82 | 0.97 | 0.06 |
| GOOD | EU | men | 48 | 24.10 | 0.70 | 23.90 | 3.50 | 34 | 70.80 | 10 | 20.80 | 4 | 8.30 | 85.60 | 8.30 | 0.86 | 0.04 | 683 | 24.05 | 0.61 | 23.57 | 3.36 | 517 | 75.70 | 138 | 20.20 | 28 | 4.10 | 84.30 | 8.05 | 0.85 | 0.05 |
| GOYA cases | EU | men | 69 | 43.54 | 6.35 | 36.36 | 5.71 | 1 | 1.44 | 9 | 13.04 | 59 | 85.50 | 120.50 | 14.86 | 1.04 | 0.08 | 255 | 42.41 | 6.30 | 36.06 | 5.79 | 2 | 0.78 | 36 | 14.11 | 217 | 85.09 | 117.03 | 14.00 | 1.02 | 0.11 |
| GOYA controls | | men | 76 | 46.15 | 7.62 | 26.07 | 3.92 | 32 | 42.10 | 32 | 42.10 | 12 | 15.78 | 94.09 | 11.83 | 0.94 | 0.08 | 306 | 47.46 | 8.68 | 26.45 | 3.81 | 113 | 36.92 | 149 | 48.69 | 44 | 14.37 | 93.69 | 11.00 | 0.93 | 0.07 |
| HERITAGE | EU | Overall | 75 | 32.21 | 13.16 | 25.34 | 4.68 | 40 | 53.30 | 25 | 33.30 | 10 | 13.30 | 88.11 | 14.97 | 0.86 | 0.09 | 424 | 36.55 | 14.71 | 25.96 | 5.01 | 207 | 48.80 | 130 | 30.70 | 87 | 20.50 | 90.92 | 14.85 | 0.88 | 0.08 |
| | | women | 41 | 31.42 | 12.81 | 24.03 | 3.75 | 28 | 68.30 | 11 | 26.80 | 2 | 4.90 | 82.03 | 11.00 | 0.82 | 0.06 | 214 | 35.89 | 14.31 | 25.25 | 5.07 | 118 | 55.10 | 59 | 27.60 | 37 | 17.30 | 87.09 | 15.01 | 0.84 | 0.08 |
| | | men | 34 | 33.17 | 13.70 | 26.91 | 5.25 | 12 | 35.30 | 14 | 41.20 | 8 | 23.50 | 95.45 | 15.96 | 0.92 | 0.08 | 210 | 37.22 | 15.11 | 26.69 | 4.86 | 89 | 42.40 | 71 | 33.80 | 50 | 23.80 | 94.83 | 13.64 | 0.92 | 0.07 |
| HRS | AF | women | 149 | 59.68 | 8.93 | 28.97 | 6.83 | 44 | 29.53 | 42 | 28.20 | 63 | 42.30 | 97.16 | 15.14 | NA | NA | 722 | 67.58 | 10.21 | 32.15 | 6.88 | 103 | 14.30 | 98 | 13.60 | 434 | 60.10 | 102.99 | 15.16 | NA | NA |
| | | men | 111 | 62.86 | 10.30 | 25.63 | 4.52 | 55 | 49.60 | 35 | 31.50 | 21 | 18.90 | 95.55 | 12.47 | NA | NA | 383 | 68.23 | 9.17 | 29.69 | 5.27 | 74 | 19.30 | 139 | 36.30 | 170 | 44.40 | 104.49 | 12.82 | NA | NA |
| HRS | EU | women | 603 | 63.00 | 9.76 | 27.30 | 5.78 | 242 | 40.10 | 189 | 31.30 | 172 | 28.50 | 94.18 | 14.89 | NA | NA | 4,124 | 69.02 | 10.71 | 28.85 | 6.29 | 1,241 | 30.10 | 1,359 | 33.00 | 1,524 | 37.00 | 96.21 | 15.51 | NA | NA |
| | | men | 440 | 63.72 | 8.79 | 27.49 | 5.17 | 156 | 35.50 | 158 | 35.90 | 126 | 28.60 | 101.82 | 12.69 | NA | NA | 2,975 | 69.70 | 9.66 | 29.36 | 4.85 | 523 | 17.60 | 1,287 | 43.30 | 1,165 | 39.20 | 105.58 | 11.73 | NA | NA |
| HYPERGENES-Cases | EU | women | 159 | 45.45 | 8.15 | 26.33 | 5.06 | 72 | 45.28 | 54 | 33.96 | 31 | 19.49 | 87.68 | 13.72 | 0.83 | 0.07 | 363 | 47.95 | 9.55 | 27.24 | 5.07 | 132 | 36.36 | 148 | 40.77 | 83 | 22.86 | 90.44 | 12.99 | 0.83 | 0.06 |
| | | men | 282 | 48.15 | 8.89 | 27.51 | 3.99 | 65 | 23.04 | 158 | 56.02 | 57 | 20.21 | 97.75 | 12.36 | 0.94 | 0.09 | 711 | 47.31 | 9.92 | 27.40 | 3.39 | 158 | 22.22 | 425 | 59.77 | 128 | 18.00 | 97.24 | 11.11 | 0.93 | 0.06 |
| HYPERGENES-Controls | | women | 121 | 58.89 | 10.40 | 24.70 | 4.09 | 66 | 54.54 | 39 | 32.23 | 12 | 9.91 | 81.96 | 8.99 | 0.81 | 0.05 | 442 | 61.03 | 12.32 | 25.06 | 3.81 | 239 | 54.07 | 159 | 35.97 | 41 | 9.27 | 85.44 | 11.48 | 0.82 | 0.08 |
| | | men | 269 | 58.01 | 11.31 | 25.54 | 3.10 | 125 | 46.46 | 115 | 42.75 | 27 | 10.03 | 95.59 | 12.55 | 0.93 | 0.05 | 533 | 58.96 | 11.01 | 26.23 | 3.33 | 194 | 36.39 | 273 | 51.21 | 65 | 12.19 | 94.90 | 10.74 | 0.92 | 0.06 |
| InCHIANTI | EU | women | 83 | 58.25 | 16.30 | 26.32 | 5.06 | 38 | 45.78 | 29 | 34.94 | 16 | 19.28 | 86.18 | 13.22 | 0.86 | 0.08 | 531 | 69.67 | 14.32 | 27.51 | 4.60 | 172 | 32.39 | 212 | 39.92 | 147 | 27.68 | 89.23 | 11.54 | 0.87 | 0.07 |
| | | men | 126 | 60.80 | 17.18 | 26.12 | 3.59 | 48 | 38.10 | 61 | 48.41 | 17 | 13.49 | 92.52 | 8.89 | 0.94 | 0.05 | 372 | 68.73 | 13.85 | 37.37 | 3.26 | 100 | 26.88 | 186 | 50.00 | 86 | 23.12 | 95.22 | 9.40 | 0.96 | 0.06 |
| KORA S3 | EU | women | 131 | 48.50 | 9.98 | 26.41 | 4.48 | 57 | 43.50 | 48 | 36.60 | 26 | 19.80 | 83.12 | 11.39 | 0.81 | 0.06 | 698 | 52.82 | 9.92 | 27.08 | 4.67 | 250 | 35.80 | 286 | 41.00 | 162 | 23.20 | 83.87 | 10.96 | 0.80 | 0.06 |
| | | men | 162 | 49.68 | 10.01 | 27.36 | 3.28 | 38 | 23.50 | 95 | 58.60 | 29 | 17.90 | 96.56 | 9.49 | 0.94 | 0.06 | 650 | 53.76 | 9.96 | 27.78 | 3.49 | 122 | 18.80 | 389 | 59.80 | 139 | 21.40 | 97.20 | 9.78 | 0.93 | 0.06 |
| KORA S4 | EU | women | 161 | 49.87 | 7.11 | 26.75 | 5.39 | 70 | 43.50 | 61 | 37.90 | 30 | 18.60 | 85.25 | 12.48 | 0.81 | 0.06 | 767 | 54.41 | 8.91 | 27.65 | 4.99 | 265 | 34.60 | 277 | 36.10 | 224 | 29.20 | 86.93 | 12.08 | 0.82 | 0.06 |
| | | men | 206 | 51.28 | 8.17 | 27.11 | 3.47 | 54 | 26.20 | 113 | 54.90 | 39 | 18.90 | 97.46 | 9.30 | 0.94 | 0.06 | 677 | 55.11 | 8.96 | 28.26 | 3.99 | 113 | 16.70 | 380 | 56.10 | 184 | 27.20 | 99.48 | 10.45 | 0.94 | 0.05 |
| LIFELINES | EU | women | 1,044 | 44.15 | 9.52 | 25.53 | 4.70 | 531 | 51.50 | 353 | 34.30 | 146 | 14.20 | 87.39 | 12.54 | 0.87 | 0.07 | 3,496 | 48.13 | 11.33 | 26.15 | 4.70 | 1,635 | 47.00 | 1,238 | 35.60 | 606 | 17.40 | 88.08 | 12.16 | 0.87 | 0.07 |
| | | men | 930 | 44.69 | 10.45 | 26.44 | 3.71 | 347 | 37.50 | 432 | 46.60 | 147 | 15.90 | 95.77 | 10.73 | 0.96 | 0.06 | 2,486 | 48.80 | 11.44 | 26.75 | 3.61 | 811 | 32.70 | 1,283 | 51.70 | 388 | 15.60 | 96.57 | 10.62 | 0.96 | 0.07 |
| LOLIPOP_EW610 | EU | women | 29 | 54.75 | 9.93 | 24.55 | 4.48 | 20 | 69.00 | 7 | 24.10 | 2 | 6.90 | 82.78 | 12.59 | 0.84 | 0.09 | 220 | 55.78 | 9.69 | 26.93 | 5.12 | 90 | 40.90 | 79 | 35.90 | 51 | 23.20 | 89.06 | 13.59 | 0.86 | 0.09 |
| | | men | 125 | 54.22 | 9.17 | 27.63 | 4.36 | 35 | 28.00 | 58 | 46.40 | 32 | 25.60 | 99.05 | 11.09 | 0.95 | 0.06 | 553 | 56.48 | 9.95 | 27.84 | 4.47 | 151 | 27.30 | 267 | 48.30 | 135 | 24.40 | 98.28 | 11.72 | 0.94 | 0.06 |
| LOLIPOP_IA317 | AS | men | 344 | 46.19 | 10.21 | 25.78 | 4.41 | 152 | 44.20 | 138 | 40.10 | 54 | 15.70 | 94.56 | 11.61 | 0.95 | 0.07 | 1,777 | 48.66 | 10.48 | 27.03 | 4.26 | 583 | 32.80 | 839 | 47.20 | 355 | 20.00 | 97.56 | 11.64 | 0.96 | 0.07 |
| LOLIPOP IA610 CASES | AS | women | 3 | 56.41 | 1.05 | 31.31 | 6.45 | 1 | 33.30 | 0 | 0.00 | 2 | 66.70 | 105.00 | 16.52 | 0.99 | 0.07 | 409 | 61.69 | 9.03 | 29.88 | 5.69 | 77 | 18.90 | 157 | 38.50 | 174 | 42.60 | 100.46 | 12.51 | 0.95 | 0.08 |
| | | men | 192 | 53.59 | 0.04 | 26.36 | 3.96 | 74 | 38.50 | 85 | 44.30 | 33 | 17.20 | 97.36 | 11.73 | 0.97 | 0.07 | 1,819 | 59.25 | 9.59 | 27.22 | 4.14 | 554 | 30.50 | 888 | 48.90 | 374 | 20.60 | 99.19 | 11.13 | 0.98 | 0.07 |
| LOLIPOP IA610 CONTROLS | | women | 7 | 46.62 | 10.60 | 29.21 | 6.64 | 0 | 0.00 | 6 | 85.70 | 1 | 14.30 | 95.00 | 11.02 | 0.90 | 0.05 | 611 | 53.73 | 9.21 | 27.95 | 4.88 | 164 | 26.80 | 276 | 45.20 | 171 | 28.00 | 94.60 | 12.21 | 0.91 | 0.09 |
| | | men | 421 | 52.11 | 10.10 | 25.79 | 4.41 | 178 | 42.40 | 170 | 40.50 | 72 | 17.70 | 94.94 | 11.68 | 0.96 | 0.07 | 3,095 | 53.17 | 10.70 | 26.80 | 3.92 | 988 | 32.00 | 1,538 | 49.70 | 566 | 18.30 | 97.52 | 10.50 | 0.96 | 0.06 |
| LURIC-Cases | EU | women | 99 | 59.30 | 10.90 | 26.40 | 4.75 | 42 | 42.40 | 34 | 34.30 | 23 | 23.20 | 92.10 | 13.70 | 0.90 | 0.07 | 493 | 67.80 | 8.82 | 27.40 | 4.53 | 154 | 31.20 | 209 | 42.40 | 130 | 26.40 | 95.00 | 12.90 | 0.91 | 0.07 |
| | | men | 449 | 56.90 | 9.84 | 27.10 | 3.87 | 142 | 31.60 | 217 | 48.30 | 90 | 20.00 | 99.90 | 11.10 | 0.98 | 0.06 | 1,323 | 65.20 | 8.79 | 27.70 | 3.62 | 327 | 24.70 | 690 | 52.20 | 306 | 23.10 | 101.50 | 10.50 | 0.98 | 0.06 |
| LURIC-Controls | EU | women | 49 | 53.40 | 10.00 | 26.40 | 5.24 | 22 | 44.90 | 17 | 34.70 | 10 | 20.40 | 89.80 | 12.90 | 0.89 | 0.07 | 247 | 63.90 | 10.10 | 27.60 | 4.79 | 75 | 30.40 | 112 | 45.30 | 60 | 24.30 | 94.10 | 12.50 | 0.90 | 0.08 |
| | | men | 87 | 50.30 | 10.30 | 27.10 | 3.83 | 25 | 28.70 | 47 | 54.00 | 15 | 17.20 | 98.90 | 11.00 | 0.98 | 0.07 | 237 | 56.30 | 12.90 | 27.50 | 3.73 | 64 | 27.00 | 124 | 52.30 | 49 | 20.70 | 99.70 | 11.00 | 0.97 | 0.07 |
| MESA | EU | women | 148 | 58.18 | 8.85 | 26.39 | 5.53 | 66 | 44.59 | 50 | 33.78 | 31 | 20.95 | 91.97 | 16.36 | 0.88 | 0.09 | 1,103 | 63.20 | 10.37 | 27.60 | 5.81 | 426 | 38.62 | 368 | 33.36 | 303 | 27.47 | 95.32 | 16.13 | 0.88 | 0.09 |
| | | men | 127 | 58.39 | 9.17 | 27.85 | 4.35 | 31 | 24.41 | 61 | 48.03 | 35 | 27.56 | 101.81 | 11.35 | 0.97 | 0.06 | 1,021 | 63.38 | 10.09 | 27.99 | 4.07 | 248 | 24.29 | 486 | 47.60 | 286 | 28.01 | 101.07 | 10.93 | 0.96 | 0.06 |
| MrOS SWEDEN | EU | men | 77 | 74.40 | 3.03 | 24.56 | 3.91 | 46 | 59.70 | 29 | 38.20 | 2 | 2.60 | NA | NA | NA | NA | 885 | 75.34 | 3.21 | 26.33 | 3.42 | 326 | 36.80 | 443 | 50.10 | 116 | 13.10 | NA | NA | NA | NA |
| NETHERLANDS TWIN REGISTER | EU | women | 360 | 43.56 | 13.56 | 24.54 | 4.56 | 220 | 61.10 | 105 | 29.20 | 35 | 9.70 | 83.48 | 12.14 | 0.81 | 0.08 | 1,618 | 44.87 | 14.42 | 25.14 | 4.44 | 915 | 56.60 | 491 | 30.30 | 212 | 13.10 | 83.37 | 12.02 | 0.80 | 0.08 |
| | | men | 317 | 28.24 | 14.44 | 25.40 | 3.39 | 141 | 44.50 | 150 | 47.30 | 26 | 8.20 | 93.30 | 11.44 | 0.90 | 0.09 | 974 | 51.04 | 14.87 | 26.12 | 3.50 | 410 | 42.10 | 419 | 43.00 | 145 | 14.90 | 94.22 | 11.37 | 0.90 | 0.08 |
| NFBC66 | EU | Overall | 2095 | 31.00 | 0.00 | 24.86 | 4.46 | 1,203 | 57.42 | 677 | 32.32 | 215 | 10.26 | 84.84 | 12.51 | 0.87 | 0.08 | 2889 | 31.00 | 0 | 24.52 | 4.12 | 1,786 | 61.82 | 851 | 29.46 | 252 | 8.72 | 82.89 | 11.77 | 0.85 | 0.09 |
| | | women | 938 | 31.00 | 0.00 | 24.43 | 5.19 | 621 | 66.20 | 207 | 22.07 | 110 | 11.73 | 79.49 | 13.10 | 0.81 | 0.08 | 1670 | 31.00 | 0 | 24.02 | 4.44 | 1,155 | 69.16 | 364 | 21.80 | 151 | 9.04 | 78.32 | 11.23 | 0.81 | 0.08 |
| | | men | 1157 | 31.00 | 0.00 | 25.20 | 3.73 | 582 | 50.30 | 470 | 40.62 | 105 | 9.08 | 89.00 | 10.28 | 0.91 | 0.06 | 1219 | 31.00 | 0 | 25.21 | 3.51 | 631 | 51.76 | 487 | 39.95 | 101 | 8.29 | 88.78 | 9.64 | 0.91 | 0.06 |
| NHS-case | EU | women | 584 | 53.47 | 6.96 | 29.66 | 5.64 | 130 | 22.70 | 200 | 34.90 | 243 | 42.40 | 89.64 | 12.57 | 0.83 | 0.08 | 516 | 53.70 | 6.49 | 30.10 | 5.62 | 91 | 17.90 | 182 | 35.70 | 236 | 46.40 | 90.68 | 13.33 | 0.84 | 0.05 |
| NHS-control | | women | 808 | 53.33 | 6.79 | 25.04 | 4.56 | 450 | 57.20 | 242 | 30.80 | 95 | 12.10 | 78.38 | 10.76 | 0.77 | 0.07 | 600 | 53.50 | 6.53 | 25.35 | 4.95 | 331 | 56.20 | 176 | 29.80 | 82 | 13.90 | 79.30 | 11.73 | 0.77 | 0.05 |
| ORCADES | EU | Overall | 371 | 55.81 | 14.77 | 28.00 | 4.77 | 91 | 24.80 | 167 | 45.50 | 109 | 29.70 | 96.07 | 13.89 | 0.93 | 0.09 | 498 | 52.28 | 15.52 | 27.47 | 4.89 | 162 | 32.99 | 208 | 42.36 | 121 | 24.64 | 91.74 | 13.56 | 0.89 | 0.09 |
| | | women | 167 | 53.77 | 14.07 | 27.11 | 4.99 | 62 | 37.35 | 66 | 39.76 | 28 | 22.89 | 89.75 | 12.95 | 0.87 | 0.07 | 308 | 52.54 | 15.84 | 27.52 | 5.48 | 112 | 36.96 | 109 | 35.97 | 82 | 27.06 | 88.73 | 13.93 | 0.86 | 0.08 |
| | | men | 204 | 57.48 | 15.14 | 28.72 | 4.47 | 29 | 14.43 | 101 | 50.25 | 71 | 35.32 | 101.24 | 12.43 | 0.98 | 0.07 | 190 | 51.85 | 15.02 | 27.39 | 3.76 | 50 | 26.60 | 99 | 52.66 | 39 | 20.74 | 96.58 | 11.40 | 0.95 | 0.07 |
| PIVUS | EU | Overall | 104 | 70.20 | 0.15 | 25.46 | 4.34 | 54 | 51.92 | 34 | 32.69 | 16 | 15.39 | 88.16 | 11.25 | 0.90 | 0.90 | 844 | 70.20 | 0.17 | 27.25 | 4.30 | 256 | 30.33 | 394 | 46.68 | 194 | 22.98 | 91.62 | 11.60 | 0.90 | 0.07 |
| | | women | 57 | 70.28 | 0.13 | 25.56 | 5.08 | 29 | 50.88 | 14 | 24.56 | 14 | 24.56 | 85.35 | 11.49 | 0.86 | 0.06 | 418 | 70.26 | 0.15 | 27.31 | 4.85 | 141 | 33.73 | 168 | 40.19 | 109 | 26.08 | 88.11 | 11.68 | 0.86 | 0.06 |
| | | men | 47 | 70.10 | 0.12 | 25.34 | 3.27 | 25 | 53.19 | 20 | 42.55 | 2 | 0.04 | 91.57 | 10.05 | 0.94 | 0.08 | 426 | 70.13 | 0.17 | 27.19 | 3.72 | 115 | 26.99 | 226 | 53.05 | 85 | 19.95 | 95.05 | 10.45 | 0.94 | 0.06 |
| PREVEND | EU | women | 622 | 46.76 | 11.20 | 25.01 | 4.55 | 351 | 56.43 | 186 | 29.90 | 77 | 12.38 | 81.97 | 13.02 | 0.82 | 0.07 | 1,132 | 49.01 | 12.40 | 26.26 | 4.83 | 512 | 45.23 | 397 | 35.07 | 216 | 19.08 | 83.09 | 12.91 | 0.82 | 0.08 |
| | | men | 659 | 49.45 | 11.61 | 26.02 | 3.70 | 288 | 43.70 | 277 | 42.03 | 93 | 14.11 | 93.56 | 11.20 | 0.93 | 0.08 | 1,211 | 51.73 | 13.29 | 26.63 | 3.65 | 416 | 34.35 | 601 | 49.60 | 192 | 15.85 | 95.01 | 11.12 | 0.94 | 0.07 |
| PROSPER | EU | Overall | 1,392 | 74.53 | 3.15 | 25.22 | 3.95 | 661 | 47.50 | 548 | 39.40 | 150 | 10.80 | NA | NA | NA | NA | 3,852 | 75.63 | 3.38 | 27.39 | 4.11 | 1,087 | 28.20 | 1,830 | 47.50 | 923 | 24.00 | NA | NA | NA | NA |
| | | women | 578 | 74.87 | 3.16 | 24.76 | 4.25 | 297 | 51.40 | 197 | 34.10 | 62 | 10.70 | NA | NA | NA | NA | 2,142 | 75.87 | 3.43 | 27.69 | 4.57 | 602 | 28.10 | 925 | 43.20 | 606 | 28.30 | NA | NA | NA | NA |

| | | | | | | | | | | | | | | | | | | | | | | | | | | | | | | | | |
|---|---|---|---|---|---|---|---|---|---|---|---|---|---|---|---|---|---|---|---|---|---|---|---|---|---|---|---|---|---|---|---|---|
| | | men | 814 | 74.29 | 3.12 | 25.55 | 3.69 | 364 | 44.70 | 351 | 43.10 | 88 | 10.80 | NA | NA | NA | NA | 1,710 | 75.34 | 3.29 | 27.03 | 3.43 | 485 | 28.40 | 905 | 52.90 | 317 | 18.50 | NA | NA | NA | NA |
| QFS | EU | Overall | 178 | 38.07 | 13.39 | 27.03 | 7.20 | 86 | 48.30 | 54 | 30.30 | 38 | 21.30 | 87.70 | 16.50 | 0.86 | 0.10 | 675 | 42.49 | 15.84 | 27.99 | 7.85 | 296 | 43.90 | 183 | 27.10 | 196 | 29.00 | 89.27 | 18.90 | 0.85 | 0.10 |
| | | women | 100 | 35.46 | 11.91 | 27.16 | 8.14 | 51 | 51.00 | 26 | 26.00 | 23 | 23.00 | 83.10 | 15.80 | 0.80 | 0.06 | 381 | 42.66 | 15.47 | 28.26 | 8.69 | 178 | 46.70 | 82 | 21.50 | 121 | 31.80 | 85.34 | 19.43 | 0.80 | 0.08 |
| | | men | 78 | 41.42 | 14.47 | 26.86 | 5.81 | 35 | 44.90 | 28 | 35.90 | 15 | 19.20 | 93.50 | 15.60 | 0.93 | 0.08 | 294 | 42.27 | 16.34 | 27.63 | 6.61 | 118 | 40.10 | 101 | 34.40 | 75 | 25.50 | 94.35 | 16.91 | 0.93 | 0.08 |
| ROTTERDAM STUDY I | EU | women | 623 | 65.73 | 7.45 | 25.95 | 4.22 | 279 | 45.60 | 243 | 39.70 | 90 | 14.70 | 85.89 | 11.95 | 0.86 | 0.09 | 2804 | 70.98 | 9.59 | 26.93 | 3.99 | 912 | 34.20 | 1,217 | 45.60 | 541 | 20.30 | 88.37 | 11.38 | 0.87 | 0.09 |
| | | men | 716 | 67.34 | 8.33 | 24.97 | 3.14 | 355 | 50.60 | 305 | 43.40 | 42 | 6.00 | 93.01 | 9.85 | 0.96 | 0.07 | 1669 | 68.32 | 7.97 | 26.01 | 2.87 | 618 | 37.80 | 885 | 54.20 | 131 | 8.00 | 94.93 | 9.31 | 0.96 | 0.07 |
| ROTTERDAM STUDY II | EU | women | 237 | 63.02 | 7.13 | 26.79 | 4.35 | 91 | 38.40 | 97 | 40.90 | 49 | 20.70 | 89.53 | 11.53 | 0.88 | 0.08 | 934 | 65.53 | 8.51 | 27.68 | 4.45 | 270 | 29.00 | 428 | 46.00 | 232 | 24.90 | 89.91 | 11.58 | 0.86 | 0.07 |
| | | men | 182 | 62.79 | 5.95 | 26.52 | 3.47 | 66 | 36.50 | 91 | 50.30 | 24 | 13.30 | 98.37 | 10.44 | 0.97 | 0.07 | 801 | 64.86 | 7.87 | 27.01 | 3.32 | 219 | 27.30 | 452 | 56.40 | 130 | 16.20 | 99.16 | 10.14 | 0.97 | 0.07 |
| ROTTERDAM STUDY III | EU | women | 269 | 55.11 | 5.33 | 26.25 | 4.71 | 123 | 46.10 | 101 | 37.80 | 43 | 16.10 | 87.52 | 11.87 | 0.83 | 0.07 | 894 | 56.49 | 6.24 | 27.84 | 5.14 | 274 | 30.70 | 375 | 42.00 | 243 | 27.20 | 90.21 | 13.11 | 0.83 | 0.08 |
| | | men | 198 | 55.21 | 4.62 | 27.63 | 3.92 | 51 | 26.00 | 95 | 48.50 | 50 | 25.50 | 99.87 | 11.55 | 0.94 | 0.08 | 712 | 56.08 | 5.68 | 28.14 | 4.14 | 156 | 22.00 | 377 | 53.20 | 176 | 24.80 | 99.98 | 11.77 | 0.93 | 0.08 |
| SARDINIA | EU | women | 54 | 47.48 | 8.74 | 25.04 | 5.23 | 33 | 61.10 | 12 | 22.22 | 9 | 16.67 | 81.53 | 13.10 | 83.20 | 0.07 | 498 | 55.79 | 11.66 | 27.52 | 5.00 | 168 | 33.73 | 193 | 38.75 | 137 | 27.51 | 87.79 | 13.03 | 0.86 | 0.95 |
| | | men | 85 | 54.13 | 10.99 | 26.90 | 3.72 | 29 | 34.11 | 40 | 47.05 | 16 | 18.82 | 93.41 | 10.24 | 96.13 | 0.06 | 393 | 60.21 | 12.59 | 28.18 | 4.03 | 68 | 17.30 | 207 | 52.67 | 118 | 30.03 | 97.07 | 9.73 | 0.97 | 0.06 |
| SHIP | EU | women | 560 | 40.38 | 13.03 | 25.61 | 4.92 | 290 | 51.80 | 159 | 28.40 | 103 | 18.40 | 80.11 | 12.19 | 0.80 | 0.06 | 1,497 | 51.62 | 15.97 | 27.41 | 5.37 | 556 | 37.10 | 501 | 33.50 | 430 | 28.70 | 84.29 | 13.09 | 0.81 | 0.07 |
| | | men | 672 | 43.14 | 14.52 | 26.76 | 3.90 | 226 | 33.60 | 319 | 47.50 | 127 | 18.90 | 93.03 | 11.05 | 0.92 | 0.06 | 1,322 | 54.66 | 15.95 | 28.14 | 4.04 | 286 | 21.60 | 662 | 50.10 | 374 | 28.30 | 97.18 | 11.73 | 0.93 | 0.06 |
| THISEAS_CASES | EU | women | 21 | 63.19 | 11.21 | 27.66 | 3.84 | 7 | 33.33 | 11 | 52.38 | 3 | 14.29 | 94.85 | 11.18 | 0.91 | 0.08 | 41 | 67.05 | 9.79 | 28.62 | 4.53 | 8 | 18.60 | 22 | 51.16 | 13 | 30.23 | 98.12 | 13.43 | 0.90 | 0.07 |
| | | men | 119 | 57.14 | 8.95 | 27.84 | 4.03 | 32 | 26.89 | 57 | 47.90 | 30 | 25.21 | 100.28 | 10.78 | 0.98 | 0.06 | 169 | 63.89 | 9.87 | 27.76 | 3.77 | 35 | 20.71 | 93 | 55.03 | 41 | 24.26 | 99.73 | 11.94 | 0.99 | 0.07 |
| THISEAS_CONTROLS | | women | 44 | 50.86 | 12.76 | 27.10 | 5.05 | 14 | 31.82 | 20 | 45.45 | 10 | 22.73 | 88.08 | 13.20 | 0.85 | 0.09 | 180 | 56.83 | 14.57 | 28.19 | 5.10 | 54 | 30.00 | 69 | 38.33 | 57 | 31.67 | 90.99 | 13.81 | 0.87 | 0.11 |
| | | men | 62 | 49.52 | 13.12 | 29.06 | 4.63 | 9 | 14.52 | 31 | 50.00 | 22 | 35.48 | 100.37 | 10.76 | 0.97 | 0.09 | 142 | 53.43 | 12.63 | 29.09 | 3.94 | 22 | 15.49 | 63 | 44.37 | 57 | 40.14 | 102.69 | 10.26 | 0.98 | 0.07 |
| TRAILS | EU | women | 212 | 19.18 | 0.59 | 23.45 | 4.02 | 142 | 67.00 | 49 | 23.10 | 14 | 6.60 | 76.29 | 9.41 | 0.76 | 0.05 | 397 | 19.11 | 0.56 | 22.90 | 3.67 | 309 | 77.80 | 59 | 14.90 | 21 | 5.30 | 75.03 | 8.62 | 0.75 | 0.05 |
| | | men | 205 | 19.25 | 0.57 | 22.80 | 3.96 | 158 | 77.10 | 28 | 13.70 | 13 | 6.30 | 81.94 | 9.86 | 0.83 | 0.05 | 327 | 19.15 | 0.52 | 22.49 | 3.64 | 258 | 78.90 | 37 | 11.30 | 17 | 5.20 | 81.69 | 9.62 | 0.82 | 0.05 |
| TWINSUK | EU | women | 1,263 | 47.56 | 12.08 | 25.19 | 4.65 | 714 | 56.53 | 386 | 30.56 | 163 | 12.91 | 79.58 | 10.62 | 0.78 | 0.06 | 1625 | 47.17 | 13.17 | 25.17 | 4.71 | 925 | 56.92 | 472 | 29.05 | 228 | 14.03 | 78.77 | 10.47 | 0.77 | 0.06 |
| WGHS | EU | women | 2,666 | 54.03 | 6.56 | 25.33 | 4.85 | 1,474 | 55.30 | 742 | 27.80 | 403 | 15.10 | 89.49 | 14.73 | 0.85 | 0.08 | 20,240 | 54.78 | 7.17 | 25.99 | 4.97 | 10,221 | 50.50 | 6,314 | 31.20 | 3,593 | 17.80 | 88.80 | 14.28 | 0.83 | 0.08 |
| WHI Pilot | AF | women | 220 | 58.52 | 6.02 | 29.49 | 6.55 | 50 | 22.73 | 87 | 39.55 | 83 | 37.73 | 89.80 | 12.96 | 0.83 | 0.07 | 1,778 | 61.30 | 6.92 | 31.59 | 6.81 | 273 | 15.35 | 573 | 32.23 | 932 | 52.42 | 92.07 | 14.11 | 0.82 | 0.07 |
| WHIYR3/YR4 | AF | women | 372 | 59.34 | 6.84 | 30.07 | 6.64 | 83 | 22.31 | 120 | 32.26 | 169 | 45.43 | 91.56 | 14.48 | 0.83 | 0.10 | 2,808 | 61.44 | 7.33 | 31.69 | 6.74 | 401 | 14.28 | 873 | 31.09 | 1,534 | 54.63 | 92.73 | 14.35 | 0.82 | 0.09 |
| YFS | EU | women | 160 | 37.31 | 5.12 | 25.79 | 5.02 | 73 | 45.60 | 55 | 34.40 | 28 | 17.50 | 85.25 | 13.44 | 0.85 | 0.07 | 907 | 37.87 | 4.94 | 25.20 | 4.94 | 515 | 56.80 | 244 | 26.90 | 141 | 15.50 | 83.19 | 12.29 | 0.83 | 0.07 |
| | | men | 206 | 37.30 | 5.01 | 26.52 | 4.19 | 78 | 37.90 | 98 | 47.60 | 30 | 14.60 | 93.89 | 11.40 | 0.95 | 0.06 | 694 | 37.80 | 5.05 | 26.82 | 4.32 | 249 | 35.90 | 307 | 44.20 | 136 | 19.60 | 94.60 | 12.18 | 0.94 | 0.07 |

**METABOCHIP Data**

| Study | Ancestry | Groups | SMOKERS N | Age mean | Age sd | BMI mean | BMI sd | Normal weight n | Normal weight % | Overweight n | Overweight % | Obese n | Obese % | Waist (cm) mean | Waist (cm) sd | WHR (ratio) mean | WHR (ratio) sd | NON-SMOKERS N | Age mean | Age sd | BMI mean | BMI sd | Normal weight n | Normal weight % | Overweight n | Overweight % | Obese n | Obese % | Waist (cm) mean | Waist (cm) sd | WHR (ratio) mean | WHR (ratio) sd |
|---|---|---|---|---|---|---|---|---|---|---|---|---|---|---|---|---|---|---|---|---|---|---|---|---|---|---|---|---|---|---|---|---|
| DESIR | EU | Overall | 1,035 | 41.60 | 9.03 | 24.48 | 4.10 | 622 | 60.15 | 322 | 31.14 | 90 | 8.70 | 84.16 | 12.45 | 0.87 | 0.09 | 3,228 | 47.75 | 10.12 | 25.47 | 3.74 | 1,560 | 48.36 | 1,289 | 39.96 | 377 | 11.69 | 85.15 | 11.33 | 0.86 | 0.09 |
| | | women | 362 | 39.57 | 8.35 | 23.17 | 4.42 | 271 | 74.86 | 61 | 16.85 | 30 | 8.29 | 75.25 | 10.98 | 0.79 | 0.06 | 1,606 | 47.91 | 10.10 | 25.15 | 4.17 | 889 | 55.42 | 504 | 31.42 | 211 | 13.15 | 79.68 | 10.50 | 0.80 | 0.07 |
| | | men | 673 | 42.68 | 9.20 | 25.18 | 3.74 | 351 | 52.23 | 261 | 38.84 | 60 | 8.93 | 88.98 | 10.37 | 0.92 | 0.07 | 1,622 | 47.59 | 10.13 | 25.79 | 3.23 | 671 | 41.37 | 785 | 48.40 | 166 | 10.23 | 90.54 | 9.37 | 0.92 | 0.06 |
| DR's EXTRA | EU | women | 44 | 65.03 | 5.42 | 27.68 | 4.35 | 12 | 27.30 | 20 | 45.50 | 12 | 27.30 | 90.64 | 11.83 | 0.89 | 0.06 | 577 | 66.58 | 5.22 | 27.57 | 5.09 | 203 | 35.20 | 226 | 39.20 | 148 | 25.60 | 88.56 | 13.20 | 0.86 | 0.067 |
| | | men | 78 | 65.97 | 5.10 | 26.98 | 3.99 | 22 | 28.20 | 42 | 53.80 | 14 | 17.90 | 98.52 | 11.71 | 1.00 | 0.06 | 468 | 66.59 | 5.50 | 27.44 | 3.69 | 125 | 26.70 | 247 | 52.80 | 96 | 20.50 | 98.11 | 10.52 | 0.98 | 0.054 |
| EGCUT | EU | women | 1,014 | 60.25 | 11.64 | 28.02 | 5.70 | 362 | 35.70 | 288 | 28.40 | 364 | 35.90 | 97.95 | 15.03 | 0.95 | 0.10 | 1,490 | 59.45 | 12.58 | 28.27 | 6.91 | 573 | 39.19 | 319 | 21.82 | 570 | 38.99 | 87.78 | 17.69 | 0.84 | 0.09 |
| | | men | 1,462 | 59.45 | 12.58 | 28.27 | 6.91 | 573 | 39.19 | 319 | 21.82 | 570 | 38.99 | 87.78 | 17.69 | 0.84 | 0.09 | 1,020 | 60.25 | 11.64 | 28.02 | 5.70 | 362 | 35.70 | 288 | 28.40 | 364 | 35.90 | 97.95 | 15.03 | 0.95 | 0.10 |
| ELY | EU | women | 59 | 54.53 | 6.64 | 25.68 | 4.91 | 31 | 52.50 | 21 | 35.60 | 7 | 11.90 | 81.43 | 12.21 | 0.82 | 0.08 | 307 | 57.80 | 7.64 | 26.30 | 4.35 | 138 | 45.00 | 112 | 36.50 | 57 | 18.60 | 81.98 | 10.72 | 0.80 | 0.08 |
| | | men | 47 | 57.69 | 7.17 | 26.04 | 2.99 | 19 | 40.40 | 24 | 51.10 | 4 | 8.50 | 93.47 | 9.12 | 0.96 | 0.07 | 215 | 58.15 | 7.97 | 26.99 | 3.09 | 60 | 27.90 | 118 | 54.90 | 37 | 17.20 | 96.06 | 9.36 | 0.96 | 0.07 |
| EPIC-non-subcohort | EU | women | 31 | 62.95 | 8.25 | 27.10 | 4.16 | 11 | 35.50 | 13 | 41.90 | 7 | 22.60 | 87.57 | 10.75 | 0.85 | 0.06 | 185 | 62.63 | 8.56 | 29.26 | 4.63 | 30 | 16.20 | 87 | 47.00 | 68 | 36.80 | 90.95 | 11.72 | 0.84 | 0.06 |
| | | men | 36 | 59.92 | 9.33 | 27.17 | 3.09 | 7 | 19.40 | 24 | 66.70 | 5 | 13.90 | 99.64 | 9.87 | 0.96 | 0.05 | 285 | 62.17 | 8.25 | 28.53 | 3.60 | 28 | 9.80 | 194 | 68.10 | 63 | 22.10 | 102.21 | 9.68 | 0.97 | 0.06 |
| EPIC-subcohort | | women | 67 | 56.58 | 8.52 | 24.39 | 3.68 | 44 | 65.70 | 18 | 26.90 | 5 | 7.50 | 78.03 | 9.85 | 0.79 | 0.06 | 407 | 59.30 | 9.71 | 25.84 | 3.76 | 183 | 45.00 | 177 | 43.50 | 47 | 11.50 | 81.49 | 10.07 | 0.79 | 0.06 |
| | | men | 39 | 56.83 | 8.57 | 25.91 | 3.64 | 15 | 38.50 | 21 | 53.80 | 3 | 7.70 | 93.46 | 12.07 | 0.92 | 0.08 | 300 | 59.79 | 9.09 | 26.00 | 2.87 | 116 | 38.70 | 161 | 53.70 | 23 | 7.70 | 94.00 | 8.69 | 0.92 | 0.06 |
| FENLAND | EU | women | 194 | 46.40 | 6.46 | 26.78 | 5.91 | 95 | 49.00 | 56 | 28.90 | 43 | 22.20 | 86.66 | 13.75 | 0.84 | 0.07 | 1,348 | 47.10 | 7.08 | 26.42 | 5.49 | 645 | 47.80 | 429 | 31.80 | 274 | 20.30 | 84.97 | 12.73 | 0.82 | 0.07 |
| | | men | 197 | 46.10 | 7.04 | 26.75 | 4.23 | 73 | 37.10 | 90 | 45.70 | 34 | 17.30 | 95.65 | 11.51 | 0.93 | 0.06 | 1,171 | 47.13 | 7.18 | 27.09 | 4.06 | 380 | 32.50 | 552 | 47.10 | 239 | 20.40 | 96.65 | 11.14 | 0.93 | 0.07 |
| FUSION2-controls | EU | women | 30 | 38.76 | 8.50 | 25.70 | 4.72 | 16 | 53.30 | 8 | 26.70 | 6 | 20.00 | 81.30 | 11.50 | 0.80 | 0.06 | 17 | 39.68 | 10.68 | 28.08 | 7.67 | 7 | 41.20 | 4 | 23.50 | 6 | 35.30 | 90.76 | 19.16 | 0.85 | 0.095 |
| | | men | 29 | 37.21 | 10.66 | 26.34 | 4.09 | 9 | 31.00 | 16 | 55.20 | 4 | 13.80 | 94.92 | 11.78 | 0.92 | 0.08 | 14 | 49.75 | 16.51 | 26.74 | 4.05 | 5 | 35.70 | 8 | 57.10 | 1 | 7.10 | 95.82 | 9.95 | 0.93 | 0.062 |
| FUSION2-T2D cases | | women | 20 | 54.07 | 10.89 | 30.64 | 8.01 | 5 | 25.00 | 4 | 20.00 | 11 | 55.00 | 99.53 | 18.14 | 0.90 | 0.07 | 23 | 59.77 | 9.50 | 31.24 | 5.77 | 3 | 13.00 | 9 | 39.10 | 11 | 47.80 | 99.70 | 14.31 | 0.89 | 0.052 |
| | | men | 43 | 55.73 | 9.30 | 30.60 | 6.21 | 5 | 11.60 | 17 | 39.50 | 21 | 48.80 | 106.92 | 15.05 | 1.00 | 0.07 | 66 | 60.52 | 6.51 | 31.74 | 5.16 | 9 | 13.60 | 18 | 27.30 | 39 | 59.10 | 108.56 | 14.21 | 1.00 | 0.071 |
| GLACIER | EU | women | 861 | 49.05 | 8.53 | 25.13 | 4.29 | 487 | 56.90 | 261 | 30.50 | 108 | 12.60 | 87.18 | 11.76 | NA | NA | 2,758 | 49.34 | 8.91 | 25.70 | 4.40 | 1,382 | 50.30 | 958 | 34.80 | 409 | 14.90 | 97.31 | 11.78 | NA | NA |
| | | men | 496 | 50.51 | 7.72 | 25.78 | 3.50 | 225 | 45.40 | 212 | 42.80 | 58 | 11.70 | 97.31 | 11.78 | NA | NA | 1,845 | 49.95 | 8.48 | 26.16 | 3.40 | 702 | 38.10 | 934 | 50.70 | 207 | 11.20 | 96.77 | 9.82 | NA | NA |
| GXE | JAMAICA | women | 20 | 38.00 | 9.12 | 32.76 | 7.13 | 2 | 10.00 | 0 | 0 | 17 | 85.00 | 93.67 | 15.66 | 0.84 | 0.09 | 449 | 40.12 | 8.09 | 32.30 | 7.49 | 91 | 20.27 | 0 | 0 | 351 | 78.17 | 88.56 | 15.27 | 0.79 | 0.07 |
| | | men | 31 | 36.61 | 7.79 | 24.59 | 6.61 | 20 | 64.50 | 0 | 0 | 9 | 29.03 | 80.01 | 16.38 | 0.82 | 0.07 | 109 | 38.67 | 8.89 | 28.57 | 6.09 | 42 | 38.53 | 0 | 0 | 65 | 59.63 | 89.91 | 16.38 | 0.85 | 0.07 |
| HEALTH2006 | EU | women | 378 | 49.49 | 11.80 | 25.00 | 4.90 | 207 | 54.80 | 109 | 28.80 | 52 | 13.80 | 83.71 | 13.09 | 0.83 | 0.07 | 1,202 | 48.62 | 13.31 | 25.42 | 5.05 | 635 | 52.80 | 345 | 28.70 | 184 | 15.30 | 83.08 | 12.41 | 0.81 | 0.07 |
| | | men | 281 | 50.29 | 11.73 | 25.74 | 4.38 | 129 | 45.90 | 117 | 41.60 | 35 | 12.50 | 93.69 | 12.34 | 0.94 | 0.07 | 1,023 | 50.47 | 13.03 | 26.83 | 4.07 | 367 | 35.90 | 464 | 45.40 | 190 | 18.60 | 95.73 | 11.95 | 0.94 | 0.07 |
| HUNT-controls | EU | women | 84 | 62.80 | 12.45 | 26.44 | 4.53 | 35 | 41.70 | 27 | 32.10 | 22 | 26.20 | 85.14 | 11.35 | 0.83 | 0.06 | 235 | 69.89 | 13.47 | 28.08 | 4.51 | 57 | 24.30 | 103 | 43.80 | 75 | 31.90 | 87.29 | 10.99 | 0.82 | 0.055 |
| | | men | 106 | 58.82 | 15.32 | 25.46 | 3.37 | 49 | 46.20 | 50 | 47.20 | 7 | 6.60 | 90.90 | 8.81 | 0.91 | 0.06 | 262 | 65.51 | 13.84 | 26.96 | 3.17 | 71 | 27.10 | 152 | 58.00 | 39 | 14.90 | 94.38 | 8.45 | 0.92 | 0.059 |
| HUNT-T2D cases | | women | 24 | 61.75 | 13.39 | 28.74 | 4.85 | 4 | 16.70 | 12 | 50.00 | 8 | 33.30 | 90.21 | 12.21 | 0.86 | 0.07 | 214 | 71.39 | 11.38 | 30.26 | 5.18 | 33 | 15.40 | 71 | 33.20 | 110 | 51.40 | 94.37 | 11.90 | 0.86 | 0.063 |
| | | men | 58 | 62.24 | 11.30 | 27.50 | 4.07 | 18 | 31.00 | 23 | 39.70 | 17 | 29.30 | 97.00 | 10.03 | 0.94 | 0.07 | 219 | 68.13 | 10.72 | 28.28 | 3.71 | 43 | 19.60 | 110 | 50.20 | 66 | 30.10 | 99.03 | 9.48 | 0.95 | 0.06 |
| IMPROVE | EU | women | 238 | 62.74 | 5.01 | 26.44 | 4.90 | 106 | 44.50 | 81 | 34.00 | 51 | 21.40 | 88.63 | 13.17 | 0.87 | 0.07 | 1,539 | 64.64 | 5.48 | 27.21 | 4.73 | 540 | 35.10 | 621 | 40.40 | 373 | 24.20 | 89.77 | 12.31 | 0.87 | 0.74 |
| | | men | 274 | 62.43 | 4.85 | 27.29 | 3.88 | 73 | 26.60 | 147 | 53.60 | 54 | 19.70 | 99.49 | 11.73 | 0.98 | 0.08 | 1,378 | 64.36 | 5.39 | 27.41 | 3.59 | 347 | 25.20 | 753 | 54.60 | 278 | 20.20 | 98.54 | 0.62 | 0.97 | 0.67 |
| INTER99 | EU | women | 1,075 | 45.49 | 7.56 | 25.02 | 4.76 | 608 | 56.60 | 309 | 28.70 | 136 | 12.70 | 79.69 | 12.23 | 0.80 | 0.06 | 1,961 | 46.19 | 8.07 | 26.16 | 5.19 | 974 | 49.70 | 610 | 31.10 | 369 | 18.80 | 80.60 | 12.40 | 0.79 | 0.06 |
| | | men | 1,070 | 46.97 | 7.66 | 26.31 | 3.95 | 418 | 39.10 | 490 | 45.80 | 158 | 14.80 | 92.34 | 10.74 | 0.92 | 0.06 | 1,837 | 46.48 | 7.76 | 27.11 | 4.04 | 578 | 31.50 | 913 | 49.70 | 345 | 18.80 | 93.79 | 11.18 | 0.92 | 0.07 |
| KORAS3 | EU | women | 186 | 37.42 | 8.39 | 24.02 | 3.91 | 123 | 66.10 | 46 | 24.70 | 17 | 9.10 | 76.05 | 8.42 | 0.77 | 0.05 | 473 | 42.26 | 13.76 | 25.64 | 4.93 | 264 | 55.80 | 127 | 26.80 | 81 | 17.10 | 80.33 | 11.97 | 0.78 | 0.06 |

| | | | | | | | | | | | | | | | | | | | | | | | | | | | | | | | | |
|---|---|---|---|---|---|---|---|---|---|---|---|---|---|---|---|---|---|---|---|---|---|---|---|---|---|---|---|---|---|---|---|---|
| KORAS3 | EU | men | 207 | 36.28 | 9.10 | 26.27 | 3.54 | 77 | 37.20 | 105 | 50.70 | 25 | 12.10 | 91.42 | 9.21 | 0.90 | 0.06 | 392 | 42.85 | 14.33 | 26.68 | 3.36 | 130 | 33.20 | 200 | 51.00 | 62 | 15.80 | 93.53 | 9.32 | 0.90 | 0.06 |
| KORAS4 | EU | women | 148 | 35.32 | 8.77 | 24.33 | 4.78 | 88 | 59.50 | 43 | 29.10 | 17 | 11.50 | 78.78 | 11.53 | 0.78 | 0.06 | 476 | 43.23 | 15.79 | 26.45 | 5.08 | 206 | 43.30 | 180 | 37.80 | 90 | 18.90 | 83.21 | 11.96 | 0.79 | 0.06 |
| | | men | 193 | 36.96 | 10.90 | 26.21 | 3.31 | 72 | 37.30 | 101 | 52.30 | 20 | 10.40 | 93.07 | 9.49 | 0.90 | 0.06 | 389 | 45.23 | 17.01 | 26.86 | 3.70 | 125 | 32.10 | 194 | 49.90 | 70 | 18.00 | 94.98 | 10.19 | 0.91 | 0.06 |
| MEC-Diabetes cases | AF | Overall | 70 | 66.83 | 8.02 | 31.21 | 6.17 | 10 | 14.29 | 26 | 37.14 | 34 | 48.57 | 102.81 | 15.61 | 0.91 | 0.09 | 314 | 69.14 | 8.27 | 31.60 | 6.73 | 35 | 11.15 | 109 | 34.71 | 166 | 52.87 | 103.75 | 14.98 | 0.91 | 0.11 |
| | | women | 51 | 64.77 | 7.03 | 31.39 | 5.66 | 7 | 13.73 | 18 | 35.29 | 26 | 50.98 | 102.00 | 15.19 | 0.90 | 0.09 | 227 | 68.77 | 8.30 | 32.36 | 7.19 | 28 | 12.33 | 70 | 30.84 | 128 | 56.39 | 103.16 | 15.66 | 0.89 | 0.11 |
| | | men | 19 | 72.37 | 8.04 | 30.73 | 7.50 | 3 | 15.79 | 8 | 42.11 | 8 | 42.11 | 105.13 | 17.99 | 0.93 | 0.06 | 87 | 70.11 | 8.17 | 29.60 | 4.86 | 7 | 8.05 | 39 | 44.83 | 38 | 43.68 | 105.29 | 13.02 | 0.97 | 0.08 |
| MEC-controls | AF | Overall | 158 | 67.93 | 8.47 | 27.46 | 6.56 | 65 | 41.14 | 51 | 32.28 | 41 | 25.95 | 93.98 | 15.69 | 0.89 | 0.12 | 1,126 | 69.58 | 8.60 | 28.75 | 6.32 | 302 | 26.82 | 441 | 39.17 | 373 | 33.13 | 95.18 | 14.74 | 0.87 | 0.11 |
| | | women | 116 | 66.79 | 8.23 | 28.09 | 18.33 | 43 | 37.07 | 38 | 32.76 | 35 | 30.17 | 94.00 | 16.08 | 0.87 | 0.09 | 910 | 69.17 | 8.71 | 29.13 | 6.61 | 237 | 26.04 | 338 | 37.14 | 327 | 35.93 | 94.48 | 15.20 | 0.86 | 0.11 |
| | | men | 42 | 71.07 | 8.42 | 25.71 | 6.20 | 22 | 52.38 | 13 | 30.95 | 6 | 14.29 | 93.92 | 14.79 | 0.95 | 0.15 | 216 | 71.29 | 7.90 | 27.15 | 4.58 | 65 | 30.09 | 103 | 47.69 | 46 | 21.30 | 98.03 | 12.34 | 0.94 | 0.08 |
| METSIM-controls | EU | men | 145 | 53.84 | 4.84 | 26.30 | 3.48 | 55 | 37.90 | 67 | 46.20 | 23 | 15.90 | 96.25 | 9.69 | 0.97 | 0.06 | 754 | 53.96 | 5.02 | 26.43 | 3.37 | 258 | 34.20 | 401 | 53.20 | 95 | 12.60 | 95.75 | 9.71 | 0.95 | 0.059 |
| METSIM-T2D cases | | men | 181 | 57.56 | 6.12 | 30.05 | 4.89 | 28 | 15.50 | 68 | 37.60 | 85 | 47.00 | 108.34 | 11.92 | 1.04 | 0.06 | 962 | 61.09 | 6.58 | 30.19 | 5.20 | 122 | 12.70 | 412 | 42.80 | 428 | 44.50 | 106.92 | 13.58 | 1.02 | 0.07 |
| NSHD | EU | women | 295 | 53.00 | 0.00 | 26.54 | 4.73 | 124 | 42.00 | 109 | 36.90 | 62 | 21.00 | 85.11 | 12.12 | 0.82 | 0.07 | 918 | 53.00 | 0.00 | 27.60 | 5.35 | 330 | 35.90 | 350 | 38.10 | 238 | 25.90 | 85.74 | 12.66 | 0.80 | 0.07 |
| | | men | 298 | 53.00 | 0.00 | 26.56 | 3.97 | 107 | 35.90 | 138 | 46.30 | 53 | 17.80 | 96.39 | 10.28 | 0.94 | 0.06 | 918 | 53.00 | 0.00 | 27.62 | 3.88 | 233 | 25.40 | 474 | 51.60 | 211 | 23.00 | 98.07 | 10.46 | 0.94 | 0.06 |
| SCARSHEEP cases | EU | women | 176 | 58.94 | 7.24 | 27.27 | 5.05 | 57 | 32.60 | 72 | 41.10 | 46 | 26.30 | 91.26 | 13.66 | 0.87 | 0.08 | 212 | 60.81 | 7.37 | 26.97 | 4.98 | 81 | 38.20 | 71 | 33.50 | 57 | 26.90 | 89.77 | 13.52 | 0.87 | 0.09 |
| | | men | 442 | 55.76 | 6.76 | 26.76 | 3.51 | 135 | 30.80 | 230 | 52.50 | 73 | 16.70 | 99.47 | 9.42 | 0.97 | 0.06 | 653 | 57.43 | 7.34 | 27.02 | 3.72 | 200 | 30.60 | 324 | 49.60 | 122 | 18.70 | 98.61 | 10.20 | 0.97 | 0.06 |
| SCARSHEEP controls | EU | women | 142 | 59.56 | 7.16 | 24.91 | 3.96 | 77 | 54.60 | 49 | 34.80 | 15 | 10.60 | 85.52 | 12.21 | 0.86 | 0.09 | 404 | 61.13 | 7.02 | 25.75 | 4.03 | 193 | 47.80 | 149 | 36.90 | 56 | 13.90 | 86.52 | 11.67 | 0.85 | 0.09 |
| | | men | 371 | 57.09 | 7.16 | 25.33 | 3.56 | 181 | 48.90 | 153 | 41.40 | 36 | 9.70 | 94.94 | 10.15 | 0.96 | 0.08 | 947 | 57.78 | 7.00 | 26.06 | 3.41 | 376 | 39.70 | 454 | 47.90 | 112 | 11.80 | 96.39 | 9.58 | 0.96 | 0.07 |
| SPT | JAMAICA | women | 66 | 43.355 | 12.02 | 28.41 | 6.06 | 20 | 30.30 | 20 | 30.30 | 20 | 30.30 | 82.64 | 13.53 | 0.89 | 0.06 | 487 | 45.9 | 13.19 | 28.58 | 5.90 | 126 | 25.87 | 173 | 35.52 | 183 | 35.58 | 84.50 | 12.31 | 0.81 | 0.11 |
| | | men | 116 | 49.88 | 13.53 | 24.30 | 4.34 | 85 | 73.28 | 19 | 16.37 | 4 | 0.03 | 101.6 | 5.16 | 0.85 | 0.11 | 235 | 47 | 15.34 | 20.24 | 4.43 | 117 | 49.79 | 84 | 35.74 | 29 | 12.34 | 84.95 | 13.02 | 0.85 | 0.07 |
| WHITEHALL | EU | women | 69 | 60.19 | 6.36 | 27.06 | 5.88 | 22 | 31.90 | 35 | 50.70 | 12 | 17.40 | 85.22 | 14.00 | 0.84 | 0.08 | 639 | 61.10 | 6.09 | 27.13 | 5.61 | 255 | 39.90 | 220 | 34.40 | 164 | 25.70 | 83.52 | 13.14 | 0.81 | 0.07 |
| | | men | 152 | 58.77 | 5.42 | 26.14 | 3.53 | 55 | 36.20 | 76 | 50.00 | 21 | 13.80 | 93.86 | 9.89 | 0.95 | 0.06 | 2,193 | 60.77 | 5.92 | 26.66 | 3.67 | 738 | 33.70 | 1,103 | 50.30 | 352 | 16.10 | 94.39 | 10.53 | 0.94 | 0.07 |

**VALIDATION Data**

| | | | SMOKERS | | | | | | | | | | | | | | | NON-SMOKERS | | | | | | | | | | | | | | |
|---|---|---|---|---|---|---|---|---|---|---|---|---|---|---|---|---|---|---|---|---|---|---|---|---|---|---|---|---|---|---|---|---|
| Study | Ancestry | Groups | N | Age | | BMI | | BMI Categories* | | | | | | Waist (cm) | | WHR (ratio) | | N | Age | | BMI | | BMI Categories* | | | | | | Waist (cm) | | WHR (ratio) | |
| | | | | | | | | Normal weight | | Overweight | | Obese | | | | | | | | | | | Normal weight | | Overweight | | Obese | | | | | |
| | | | | mean | sd | mean | sd | n | % | n | % | n | % | mean | sd | mean | sd | | mean | sd | mean | sd | n | % | n | % | n | % | mean | sd | mean | sd |
| UKBB | EU | combined | 13,416 | 54.914 | 8.0029 | 26.9962 | 4.8998 | 4,800 | 35.78 | 5,384 | 40.13 | 3,007 | 22.42 | 90.866 | 13.590 | 0.890 | 0.088 | 105,218 | 57.188 | 7.889 | 27.605 | 4.814 | 32,751 | 31.100 | 45,084 | 42.850 | 26,673 | 25.350 | 90.8008 | 13.61 | 0.8732 | 0.09071 |

**Supplementary Table 5**. Results for approximate conditional analyses to identify secondary signals and to determine independence from previously-identified anthropometric-associated SNPs for novel loci identifie in Approach 1 (SNPadjSMK). *The top variant after conditioning differed between ARIC and FHS for rs6012558.

**Secondary Signals**

| | Tag SNP (TS) | | | | Condition on | Most Significant after conditioning | | | Before Conditioning | | | ARIC reference panel After Conditioning | | | FHS reference panel After Conditioning | | |
|---|---|---|---|---|---|---|---|---|---|---|---|---|---|---|---|---|---|
| Chr | Marker | β | SE | P | TS Marker | Marker | Distance | $r^2$ | β | SE | P | β | SE | P | β | SE | P |
| **BMI** | | | | | | | | | | | | | | | | | |
| 3 | rs6794880 | 0.028 | 0.005 | **4.3E-08** | rs6794880 | rs7627971 | 962779 | 0.006 | 0.017 | 0.004 | 7.95E-05 | 0.016 | 0.004 | 1.62E-04 | 0.015 | 0.004 | 3.44E-04 |
| 6 | rs4498364 | -0.023 | 0.004 | **7.2E-09** | rs4498364 | rs13213457 | 945919 | 0.001 | -0.020 | 0.005 | 3.03E-05 | -0.020 | 0.005 | 2.98E-05 | -0.020 | 0.005 | 5.15E-05 |
| **WHRadjBMI** | | | | | | | | | | | | | | | | | |
| 6 | rs1049281 | -0.025 | 0.004 | 2.16E-09 | rs1049281 | rs2261033 | 367024 | 0.129 | -0.0193 | 0.0034 | 1.92E-08 | -0.013 | 0.003 | 4.44E-05 | -0.014 | 0.003 | 1.33E-05 |
| **WCadjBMI** | | | | | | | | | | | | | | | | | |
| 3 | rs4378999 | 0.036 | 0.007 | **4.12E-08** | rs4378999 | rs4256170 | 16520 | 0.064 | 0.093 | 0.019 | 1.37E-06 | 0.080 | 0.019 | *2.63E-05* | 0.086 | 0.019 | *7.67E-06* |
| 9 | rs9409082 | 0.027 | 0.005 | **1.49E-08** | rs9409082 | rs902143 | 280862 | 0.005 | 0.016 | 0.004 | 6.49E-06 | 0.015 | 0.004 | 2.08E-05 | 0.016 | 0.004 | 8.22E-06 |
| 20 | rs6012558 | 0.020 | 0.004 | **1.91E-08** | rs6012558 | rs4810880/ | 100357 | 0.125 | 0.216 | 0.004 | 2.96E-07 | 0.143 | 0.004 | 3.33E-04 | - | - | - |
| | | | | | | rs237724* | 406146 | 0.094 | 0.024 | 0.006 | 1.17E-05 | - | - | - | 0.020 | 0.005 | 2.83E-04 |

**Independence of known loci**

| | Tag SNP (TS) Before Conditioning | | | | Condition On | | | | Known SNP | | | ARIC reference panel TS After Conditioning | | | FHS reference panel TS After Conditioning | | |
|---|---|---|---|---|---|---|---|---|---|---|---|---|---|---|---|---|---|
| Chr | Marker | β | SE | P | Known Marker | Trait | Distance | $r^2$ | β | SE | P | β | SE | P | β | SE | P |
| **BMI** | | | | | | | | | | | | | | | | | |
| 2 | rs10929925 | -0.020 | 0.003 | **1.1E-09** | rs10495537 | Visceral fat | 538962 | 3.32E-07 | 0.002 | 0.004 | 6.59E-01 | -0.020 | 0.003 | **1.42E-09** | -0.020 | 0.003 | **1.36E-09** |
| | | | | | rs7586540 | coronary artery calcification | 102806 | 0.02039 | 0.002 | 0.007 | 7.79E-01 | -0.020 | 0.003 | **1.20E-09** | -0.020 | 0.003 | **1.36E-09** |
| **WHRadjBMI** | | | | | | | | | | | | | | | | | |
| 6 | rs1049281 | -0.025 | 0.004 | **2.16E-09** | rs6457374 | height | 35694 | 0.713 | 0.023 | 4.40E-03 | 1.37E-07 | -0.007 | 0.003 | 5.08E-03 | -0.008 | 0.003 | 3.41E-03 |
| | | | | | rs2247056 | height/triglycerides | 28923 | 0.714 | -0.021 | 4.00E-03 | 1.41E-07 | -0.008 | 0.003 | 3.03E-03 | -0.009 | 0.003 | 1.95E-03 |
| | | | | | rs2844479 | height | 336389 | 0.090 | -0.016 | 3.90E-03 | 2.94E-05 | -0.020 | 0.004 | 7.72E-07 | -0.021 | 0.004 | 4.47E-07 |
| | | | | | rs2857693 | height | 351817 | 0.112 | 0.015 | 3.70E-03 | 3.76E-05 | -0.020 | 0.004 | 1.05E-06 | -0.020 | 0.004 | 6.39E-07 |
| | | | | | rs2256183 | height | 143962 | 0.223 | -0.011 | 4.00E-03 | 0.006 | -0.019 | 0.004 | 2.01E-07 | -0.020 | 0.004 | 1.92E-07 |
| | | | | | rs7741091 | HIPadjBMI | 116064 | 0.149 | -0.010 | 3.70E-03 | 0.007 | -0.021 | 0.004 | 1.10E-07 | -0.021 | 0.004 | 1.11E-07 |
| | | | | | rs13437082 | height | 117993 | 0.102 | 0.010 | 4.20E-03 | 0.022 | -0.022 | 0.004 | **4.50E-08** | -0.022 | 0.004 | **4.67E-08** |
| | | | | | rs1265097 | height | 130108 | 0.056 | 0.012 | 7.30E-03 | 0.098 | -0.023 | 0.004 | **1.27E-08** | -0.023 | 0.004 | **1.29E-08** |
| | | | | | rs12175489 | VATadjBMI | 141020 | 0.010 | 0.008 | 5.80E-03 | 0.167 | -0.024 | 0.004 | **6.72E-09** | -0.024 | 0.004 | **5.36E-09** |
| | | | | | rs879882 | height | 97115 | 0.093 | -0.002 | 3.90E-03 | 0.593 | -0.024 | 0.004 | **2.07E-09** | -0.024 | 0.004 | **1.83E-09** |
| **WCadjBMI** | | | | | | | | | | | | | | | | | |
| 2 | rs6743226 | -0.022 | 0.004 | **1.20E-10** | rs4344931 | Height | 418445 | 0.043 | -0.008 | 0.004 | 6.91E-02 | -0.022 | 0.004 | **1.40E-10** | -0.023 | 0.003 | **1.22E-10** |
| | | | | | rs6739772 | HIPadjBMI | 396261 | 0.043 | -0.007 | 0.004 | 1.05E-01 | -0.022 | 0.004 | **1.49E-10** | -0.023 | 0.003 | **1.25E-10** |
| | | | | | rs11687941 | Height | 45562 | 0.341 | 0.019 | 0.005 | 6.74E-05 | -0.006 | 0.004 | 1.21E-01 | - | - | - |
| | | | | | rs12694997 | Height | 26014 | 0.375 | -0.017 | 0.004 | 6.96E-05 | -0.015 | 0.003 | 5.58E-07 | -0.014 | 0.003 | 5.84E-07 |
| 3 | rs4378999 | -0.036 | 0.007 | **4.12E-08** | rs13088462 | Height | 136933 | 0.432 | -0.035 | 0.008 | 2.58E-05 | -0.023 | 0.006 | 7.06E-05 | -0.012 | 0.012 | 3.20E-01 |
| | | | | | rs4256170 | Height | 16520 | 0.010 | 0.093 | 0.019 | 1.37E-06 | -0.032 | 0.006 | 6.91E-07 | -0.034 | 0.006 | 2.04E-07 |
| 4 | rs7697556* | 0.021 | 0.004 | **5.21E-09** | rs9993613 | HipadjBMI, Height | 39299 | 1 | 0.021 | 0.004 | 3.04E-07 | - | - | - | 0.006 | 0.002 | 3.41E-03 |
| 6 | rs615672ɫ | -0.023 | 0.004 | **3.31E-08** | rs2076529 | WHRadjBMI | 210216 | 0.174 | -0.013 | 0.004 | 2.03E-04 | -0.017 | 0.004 | 9.56E-06 | -0.017 | 0.004 | 9.50E-06 |
| | | | | | rs7759742 | WHRadjBMI | 192435 | 0.124 | 0.013 | 0.004 | 2.26E-04 | -0.017 | 0.004 | 8.17E-06 | -0.016 | 0.004 | 1.12E-05 |
| | | | | | rs6457620 | Height | 89828 | 0.039 | 0.004 | 0.004 | 2.91E-01 | -0.023 | 0.004 | **3.86E-08** | -0.023 | 0.004 | **3.40E-08** |
| | | | | | rs13196329 | Visceral fat | 248800 | 0.002 | 0.022 | 0.017 | 1.89E-01 | -0.023 | 0.004 | **2.20E-08** | -0.024 | 0.004 | **1.91E-08** |
| 7 | rs10269774 | 0.023 | 0.004 | **2.87E-08** | rs2888877 | Height | 25572 | 0.514 | 0.020 | 0.005 | 6.18E-05 | 0.013 | 0.003 | 7.40E-05 | 0.013 | 0.003 | 7.04E-05 |
| | | | | | rs42039 | Height | 9550 | 0.663 | 0.025 | 0.005 | 1.01E-07 | 0.007 | 0.003 | 9.34E-03 | 0.008 | 0.003 | 8.07E-03 |
| | | | | | rs42235 | HipadjBMI, Height | 5896 | 0.857 | 0.016 | 0.004 | 1.38E-04 | 0.009 | 0.002 | 5.60E-06 | 0.009 | 0.002 | 4.48E-06 |
| | | | | | rs2040494 | Height | 2933 | 0.557 | 0.016 | 0.004 | 3.27E-05 | 0.012 | 0.003 | 1.47E-04 | 0.012 | 0.003 | 1.54E-04 |
| | | | | | rs2282978 | Height | 10438 | 1 | -0.018 | 0.004 | 2.44E-06 | - | - | - | - | - | - |
| | | | | | rs2188177 | Height | 405188 | 0.035 | 0.008 | 0.004 | 5.51E-02 | 0.023 | 0.004 | **2.50E-08** | 0.023 | 0.004 | **2.58E-08** |
| 9 | rs9409082* | -0.027 | 0.005 | **1.49E-08** | rs9408815 | Lead SNP for Pjoint | 10528 | 0.834 | 0.026 | 0.005 | **2.28E-08** | - | - | - | -0.005 | 0.003 | 5.65E-02 |
| | | | | | rs902143 | Height SNP | 280862 | 0.005 | 0.016 | 0.004 | 6.49E-06 | -0.026 | 0.005 | **4.61E-08** | -0.026 | 0.005 | **1.85E-08** |
| | | | | | rs10120372 | Visceral adipose tissue/subcutaneous adipose tissu | 460507 | 0.161 | 0.014 | 0.009 | 1.02E-01 | -0.026 | 0.005 | **2.54E-08** | -0.026 | 0.005 | **3.17E-08** |
| 20 | rs6012558 | 0.020 | 0.004 | **1.91E-08** | rs17450430 | Height SNP | 240978 | 0.555 | -0.021 | 0.005 | 4.30E-06 | 0.011 | 0.003 | 1.73E-04 | 0.011 | 0.003 | 1.59E-04 |
| | | | | | rs237743 | Height SNP | 371733 | 0.441 | 0.024 | 0.005 | 5.76E-07 | 0.011 | 0.003 | 2.27E-04 | 0.011 | 0.003 | 2.39E-04 |

ɫ - ld calculated in HapMap r22

**Supplementary Table 6**. Results for approximate conditional analyses to identify secondary signals and to determine independence from previously-identified anthropometric-associated SNPs for novel loci identified in Approach 2 (SNPjoint). *IndexSNP is also a previously identified height SNP.

Secondary Signals

| | | | | | | | | | | | | | ARIC reference panel | | | | | | | | | | | | | | FHS reference panel | | | | | | |
|---|---|---|---|---|---|---|---|---|---|---|---|---|---|---|---|---|---|---|---|---|---|---|---|---|---|---|---|---|---|---|---|---|---|
| Chr | Marker | Tag SNP (TS) | | | | | | | Condition on Lead SNP | Most Significant Marker After Conditioning | Distance | $r^2$ | Before Conditioning | | | | | | | After Conditioning | | | | | | | After Conditioning (FHS) | | | | | | |
| | | SMOKERS | | | NON-SMOKERS | | | $P_{joint}$ | | | | | SMOKERS | | | NON-SMOKERS | | | $P_{joint}$ | SMOKERS | | | NON-SMOKERS | | | $P_{joint}$ | SMOKERS | | | NON-SMOKERS | | | $P_{joint}$ |
| | | β | SE | P | β | SE | P | | | | | | β | SE | P | β | SE | P | | β | SE | P | β | SE | P | | β | SE | P | β | SE | P | |
| BMI | | | | | | | | | | | | | | | | | | | | | | | | | | | | | | | | | |
| 3 | rs13069244 | 0.061 | 0.014 | 1.8E-05 | 0.031 | 0.008 | 6.6E-05 | **3.5E-08** | rs13069244 | rs6443750 | 888510 | 0.00011 | -0.006 | 0.010 | 5.48E-01 | -0.018 | 0.005 | **8.11E-04** | 4.64E-02 | - | - | - | - | - | - | - | -0.006 | 0.010 | 5.69E-01 | -0.018 | 0.005 | 8.66E-04 | 3.32E-03 |
| WHRadjBMI | | | | | | | | | | | | | | | | | | | | | | | | | | | | | | | | | |
| **6** | rs1049281 | -0.022 | 0.009 | 1.27E-02 | -0.027 | 0.005 | 1.98E-08 | **5.3E-09** | rs1049281 | rs9262169 | 508863 | 0.004 | 0.014 | 0.0298 | 0.6384 | 0.057 | 0.0159 | 3.38E-04 | 1.45E-03 | 0.012186 | 0.02979 | 6.82E-01 | 0.05437 | 0.015896 | 6.25E-04 | 2.65E-03 | -0.008 | 0.004 | 4.30E-02 | -0.010 | 0.003 | 3.83E-04 | 2.35E-04 |
| WCadjBMI | | | | | | | | | | | | | | | | | | | | | | | | | | | | | | | | | |
| 9 | rs9408815 | 0.012 | 0.010 | 2.28E-01 | 0.030 | 0.005 | 4.22E-09 | **1.7E-08** | rs9408815 | rs902143 | 10528 | 0.834 | 0.0156 | 0.0074 | 0.03654 | 0.0178 | 0.0041 | 1.16E-05 | 8.75E-06 | 0.015137 | 0.00739 | 0.040537 | 0.016394 | 0.004094 | 6.21E-05 | *4.04E-05* | 0.015 | 0.0074 | 0.039463 | 0.017 | 0.004 | 4.73E-05 | *3.05E-05* |

Independence of known loci

| | | | | | | | | | | | | | ARIC reference panel | | | | | | | | | | | | | | FHS reference panel | | | | | | |
|---|---|---|---|---|---|---|---|---|---|---|---|---|---|---|---|---|---|---|---|---|---|---|---|---|---|---|---|---|---|---|---|---|---|
| Chr | Marker | Tag SNP (TS) | | | | | | | Condition on | | Distance | $r^2$ | Known SNP | | | | | | | Tag SNP After Conditioning | | | | | | | Tag SNP After Conditioning | | | | | | |
| | | SMOKERS | | | NON-SMOKERS | | | $P_{joint}$ | Marker | Trait | | | SMOKERS | | | NON-SMOKERS | | | $P_{joint}$ | SMOKERS | | | NON-SMOKERS | | | $P_{joint}$ | SMOKERS | | | NON-SMOKERS | | | $P_{joint}$ |
| | | β | SE | P | β | SE | P | | | | | | β | SE | P | β | SE | P | | β | SE | P | β | SE | P | | β | SE | P | β | SE | P | |
| BMI | | | | | | | | | | | | | | | | | | | | | | | | | | | | | | | | | |
| **15** | rs12902602 | 0.047 | 0.007 | **1.77E-11** | -0.002 | 0.004 | 0.547 | **1.12E-10** | **rs16969968** | **smoking** | 84476 | 0.40772 | -0.039 | 0.008 | 3.28E-07 | 0.001 | 0.004 | 7.67E-01 | 2.1E-06 | 0.026 | 0.006 | 3.80E-06 | -0.002 | 0.003 | 0.589 | 6.70E-05 | 0.027 | 0.0057 | 3.42E-06 | -0.002 | 0.003 | 0.590097 | 1.80E-05 |
| | | | | | | | | | **rs6495309** | **smoking** | 52156 | 0.08937 | 0.035 | 0.009 | 4.01E-05 | -0.008 | 0.005 | 8.05E-02 | 4.84E-05 | 0.040 | 0.007 | 3.54E-09 | -0.001 | 0.004 | 0.857 | 7.87E-08 | 0.04 | 0.0068 | 4.09E-09 | -6E-04 | 0.004 | 0.869515 | **3.06E-08** |
| | | | | | | | | | **rs899997** | **stroke** | 52177 | 0.20719 | -0.023 | 0.008 | 4.47E-03 | 0.002 | 0.004 | 6.11E-01 | 1.50E-02 | 0.038 | 0.006 | 9.91E-10 | -0.001 | 0.003 | 0.682 | **1.84E-09** | 0.04 | 0.0065 | 9.39E-10 | -0.002 | 0.004 | 0.661175 | **6.71E-09** |
| | | | | | | | | | **rs3825807** | **CHD** | 121710 | 0.56435 | 0.038 | 0.008 | 5.66E-07 | 0.001 | 0.004 | 7.97E-01 | 3.16E-06 | 0.023 | 0.005 | 5.89E-06 | -0.003 | 0.003 | 0.282 | 1.54E-05 | 0.024 | 0.0052 | 5.60E-06 | -0.003 | 0.003 | 0.289177 | 1.90E-05 |
| WCadjBMI | | | | | | | | | | | | | | | | | | | | | | | | | | | | | | | | | |
| 2 | rs6743226 | -0.018 | 0.007 | 1.29E-02 | -0.023 | 0.004 | **2.61E-09** | **5.81E-10** | rs4344931 (rs4311055, rsquare=1.00) | Height SNP | 418445 | 0.043 | -0.008 | 0.009 | 4.15E-01 | -0.009 | 0.005 | 8.44E-02 | 1.59E-01 | -0.018 | 0.007 | 1.24E-02 | -0.023 | 0.004 | **1.50E-09** | **5.11E-10** | -0.018 | 0.007 | 1.23E-02 | | - | | - |
| | | | | | | | | | rs6739772 (rs3936203,rsquare = 0.9885) | Hipadj | 396261 | 0.043 | -0.004 | 0.010 | 6.57E-01 | -0.009 | 0.005 | 1.01E-01 | 2.36E-01 | -0.018 | 0.007 | 1.25E-02 | -0.023 | 0.004 | **1.51E-09** | **5.18E-10** | -0.018 | 0.007 | 1.14E-02 | | - | | - |
| | | | | | | | | | rs11687941 | Height SNP | 45562 | 0.341 | 0.022 | 0.010 | 2.38E-02 | 0.018 | 0.005 | 8.69E-04 | 3.32E-04 | -0.010 | 0.006 | 1.11E-01 | -0.017 | 0.003 | 4.08E-07 | 7.57E-07 | -0.010 | 0.006 | 1.16E-01 | -0.017 | 0.003 | 4.25E-07 | 8.15E-07 |
| | | | | | | | | | rs12694997 | Height SNP | 26014 | 0.375 | -0.025 | 0.009 | 3.06E-03 | -0.014 | 0.005 | 2.78E-03 | 1.42E-04 | -0.006 | 0.006 | 2.98E-01 | -0.017 | 0.003 | 1.65E-07 | 6.52E-07 | -0.006 | 0.006 | 3.13E-01 | -0.017 | 0.003 | 1.60E-07 | 6.53E-07 |
| 4 | rs7697556* | 0.004 | 0.007 | 6.26E-01 | 0.025 | 0.004 | **7.30E-11** | **2.93E-09** | rs9993613 | HipadjBMI, Height | 39299 | 1 | 0.003 | 0.008 | 7.20E-01 | 0.025 | 0.004 | **7.30E-09** | 6.21E-08 | - | - | - | - | - | - | - | - | - | - | - | - | - | - |
| 9 | rs9408815 | 0.012 | 0.010 | 2.28E-01 | 0.030 | 0.005 | **4.22E-09** | **7.38E-09** | rs9409082 | lead SNP for PadjSMK | 10528 | 0.834 | -0.017 | 0.010 | 8.13E-02 | -0.029 | 0.005 | **2.56E-08** | **4.58E-08** | - | - | - | - | - | - | - | -0.002 | 0.006 | 7.01E-01 | 0.008 | 0.003 | 8.00E-03 | 0.027605 |
| | | | | | | | | | rs9409082 | Height SNP | 10528 | 0.834 | -0.017 | 0.010 | 8.13E-02 | -0.029 | 0.005 | **2.56E-08** | **4.58E-08** | - | - | - | - | - | - | - | - | - | - | - | - | - | - |
| | | | | | | | | | rs902143 | Height SNP | 291390 | 0.016 | 0.016 | 0.007 | 3.65E-02 | 0.018 | 0.004 | 1.16E-05 | 8.75E-06 | 0.011 | 0.010 | 2.73E-01 | 0.029 | 0.005 | **8.39E-09** | **3.42E-08** | 0.011 | 0.010 | 2.64E-01 | 0.029 | 0.005 | **6.44E-09** | **2.58E-08** |
| | | | | | | | | | rs10120372 | VAT/SAT | 449979 | 0.128 | -0.004 | 0.018 | 8.24E-01 | 0.016 | 0.010 | 1.05E-01 | 2.60E-01 | 0.012 | 0.010 | 2.19E-01 | 0.029 | 0.005 | **4.14E-09** | **1.47E-08** | 0.012 | 0.010 | 2.16E-01 | 0.029 | 0.005 | **4.86E-09** | **1.71E-08** |
| WHRadjBMI | | | | | | | | | | | | | | | | | | | | | | | | | | | | | | | | | |
| **6** | rs1049281 | -0.022 | 0.009 | 1.27E-02 | -0.027 | 0.005 | **1.98E-08** | **5.3E-09** | rs1265097 | Height | 130108 | 0.056 | 0.0045 | 0.0147 | 0.7595 | 0.0133 | 0.0086 | 1.21E-01 | 2.89E-01 | -0.021 | 0.008 | 1.29E-02 | -0.026 | 0.005 | **5.00E-08** | **1.59E-08** | -0.021 | 0.0084 | 0.012815 | -0.026 | 0.005 | 5.06E-08 | **1.61E-08** |
| | | | | | | | | | rs879882 | Height | 97115 | 0.093 | 0.0008 | 0.004 | 0.8443 | 0.0007 | 0.0029 | 8.13E-01 | 9.52E-01 | -0.022 | 0.009 | 1.07E-02 | -0.027 | 0.005 | **7.18E-09** | **2.06E-09** | -0.022 | 0.0085 | 0.010552 | -0.027 | 0.005 | 6.60E-09 | **1.88E-09** |
| | | | | | | | | | rs6457374 | Height | 35694 | 0.713 | 0.0048 | 0.0044 | 0.2693 | 0.0127 | 0.0033 | 1.34E-04 | 3.35E-04 | -0.018 | 0.008 | 0.024 | -0.017 | 0.004 | 2.11E-05 | 9.35E-06 | -0.018 | 0.008 | 0.024072 | -0.018 | 0.004 | 1.72E-05 | 7.61E-06 |
| | | | | | | | | | rs2247056 | Height/triglycerides | 28923 | 0.714 | -0.0039 | 0.0043 | 0.3657 | -0.0129 | 0.0032 | 6.24E-05 | 1.96E-04 | -0.019 | 0.008 | 0.020 | -0.017 | 0.004 | 3.11E-05 | 1.13E-05 | -0.019 | 0.008 | 0.019835 | -0.018 | 0.004 | 2.44E-05 | 8.99E-06 |
| | | | | | | | | | rs13437082 | Height | 117993 | 0.102 | 0.0027 | 0.0043 | 0.5293 | 0.0058 | 0.0033 | 7.34E-02 | 1.75E-01 | -0.021 | 0.009 | 0.015 | -0.025 | 0.005 | 6.55E-08 | **2.36E-08** | -0.021 | 0.0085 | 0.014967 | -0.025 | 0.005 | 6.72E-08 | **2.43E-08** |
| | | | | | | | | | rs2256183 | Height | 143962 | 0.223 | -0.0099 | 0.0084 | 0.2372 | -0.011 | 0.0047 | 1.83E-02 | 3.23E-02 | -0.017 | 0.008 | 0.027 | -0.022 | 0.004 | 2.73E-07 | 1.60E-07 | -0.017 | 0.0078 | 0.027132 | -0.022 | 0.004 | 2.79E-07 | 1.62E-07 |
| | | | | | | | | | rs2857693 | Height | 351817 | 0.112 | 0.0063 | 0.0039 | 0.1036 | 0.0119 | 0.0029 | 4.51E-05 | 5.98E-05 | -0.019 | 0.008 | 0.023 | -0.023 | 0.005 | 9.62E-07 | 4.61E-07 | -0.02 | 0.0085 | 0.021522 | -0.023 | 0.005 | 6.50E-07 | 3.00E-07 |
| | | | | | | | | | rs2844479 | Height | 336389 | 0.090 | -0.0047 | 0.0041 | 0.2518 | -0.0111 | 0.003 | 2.28E-04 | 5.52E-04 | -0.020 | 0.009 | 0.018 | -0.024 | 0.005 | 4.43E-07 | 1.79E-07 | -0.02 | 0.0085 | 0.017438 | -0.024 | 0.005 | 3.10E-07 | 1.22E-07 |
| | | | | | | | | | rs12175489 | VATadjBMI | 141020 | 0.010 | 0.001 | 0.0053 | 0.8543 | -0.0005 | 0.0042 | 9.10E-01 | 9.75E-01 | -0.021 | 0.009 | 0.013 | -0.027 | 0.005 | **1.47E-08** | **4.81E-09** | -0.021 | 0.0086 | 0.012573 | -0.027 | 0.005 | 1.57E-08 | **5.09E-09** |
| | | | | | | | | | rs7741091 | HIPadjBMI | 116064 | 0.149 | -0.0025 | 0.004 | 0.5326 | -0.0059 | 0.003 | 4.77E-02 | 1.19E-01 | -0.021 | 0.008 | 0.015 | -0.025 | 0.005 | 9.70E-08 | **3.51E-08** | -0.021 | 0.0085 | 0.015194 | -0.025 | 0.005 | 9.77E-08 | **3.54E-08** |

**Supplementary Table 7**. Results for approximate conditional analyses to identify secondary signals and to determine independence from previously-identified anthropometric-associated SNPs for novel loci identified in Approach 3 (SNPint).

**Secondary Signals**

| | | | | | | | | | | | | | | ARIC reference panel | | | | | | | | | | | | | | FHS reference panel | | | | | | |
|---|---|---|---|---|---|---|---|---|---|---|---|---|---|---|---|---|---|---|---|---|---|---|---|---|---|---|---|---|---|---|---|---|
| Chr | Marker | Tag SNP (TS) | | | | | | | Condition on | Most Significant Marker After Conditioning | Distanc | $r^2$ | Before Conditioning | | | | | | | After Conditioning | | | | | | | Most Significant | After Conditioning (FHS) | | | | | | |
| | | SMOKERS | | | NON-SMOKERS | | | $P_{int}$ | | | | | SMOKERS | | | NON-SMOKERS | | | $P_{int}$ | SMOKERS | | | NON-SMOKERS | | | $P_{int}$ | | SMOKERS | | | NON-SMOKERS | | | $P_{int}$ |
| | | β | SE | P | β | SE | P | | Lead SNP | | | | β | SE | P | β | SE | P | | β | SE | P | β | SE | P | | | β | SE | P | β | SE | P | |
| BMI | | | | | | | | | | | | | | | | | | | | | | | | | | | | | | | | | | |
| 4 | **rs336396** | 0.063 | 0.012 | **4.8E-08** | -0.0059 | 0.0062 | 0.343 | **2.1E-08** | **rs336396** | rs10857395 | 389724 | 0.001 | 0.035 | 0.013 | 5.7E-03 | 0.001 | 0.007 | 0.853 | 1.3E-02 | 0.036 | 0.012 | 0.004 | 0.0008 | 0.0070 | 0.9047 | 0.009 | rs10857395 | 0.036 | 0.012 | 3.87E-03 | - | - | - | - |

**Independence of known SNPs**

| | | | | | | | | | | | | | | ARIC reference panel | | | | | | | | | | | | | | | | | | | | |
|---|---|---|---|---|---|---|---|---|---|---|---|---|---|---|---|---|---|---|---|---|---|---|---|---|---|---|---|---|---|---|---|---|---|---|
| Chr | Marker | Tag SNP (TS) | | | | | | | Condition on | | Distanc | $r^2$ | Known SNP | | | | | | | Tag SNP After Conditioning | | | | | | | | Tag SNP After Conditioning | | | | | | |
| | | SMOKERS | | | NON-SMOKERS | | | $P_{int}$ | Marker | Trait | | | SMOKERS | | | NON-SMOKERS | | | $P_{int}$ | SMOKERS | | | NON-SMOKERS | | | $P_{int}$ | | SMOKERS | | | NON-SMOKERS | | | $P_{int}$ |
| | | β | SE | P | β | SE | P | | | | | | β | SE | P | β | SE | P | | β | SE | P | β | SE | P | | | β | SE | P | β | SE | P | |
| BMI | | | | | | | | | | | | | | | | | | | | | | | | | | | | | | | | | | |
| 15 | **rs12902602** | 0.047 | 0.007 | **1.77E-11** | -0.002 | 0.004 | 0.547 | **4.06E-11** | **rs16969968** | **smoking** | 84476 | 0.408 | -0.039 | 0.008 | 3.28E-07 | 0.001 | 0.004 | 7.67E-01 | 9.75E-07 | 0.026 | 0.006 | 3.80E-06 | -0.002 | 0.003 | 0.589 | 4.22E-06 | **smoking** | 0.027 | 0.006 | 3.42E-06 | -0.002 | 0.003 | 0.590 | 7.18E-07 |
| | | | | | | | | | **rs6495309** | **smoking** | 52156 | 0.089 | 0.035 | 0.009 | 4.01E-05 | -0.008 | 0.005 | 8.05E-02 | 2.43E-06 | 0.040 | 0.007 | **3.54E-09** | -0.001 | 0.004 | 0.857 | **1.96E-08** | **smoking** | 0.040 | 0.007 | **4.09E-09** | -0.001 | 0.004 | 0.870 | **9.51E-10** |
| | | | | | | | | | **rs899997** | **stroke** | 52177 | 0.207 | -0.023 | 0.008 | 4.47E-03 | 0.002 | 0.004 | 6.11E-01 | 3.33E-03 | 0.038 | 0.006 | **9.91E-10** | -0.001 | 0.003 | 0.682 | **3.34E-09** | **stroke** | 0.040 | 0.007 | **9.39E-10** | -0.002 | 0.004 | 0.661 | **1.67E-10** |
| | | | | | | | | | **rs3825807** | **CHD** | 121710 | 0.564 | 0.038 | 0.008 | 5.66E-07 | 0.001 | 0.004 | 7.97E-01 | 4.57E-06 | 0.023 | 0.005 | 5.89E-06 | -0.003 | 0.003 | 0.282 | 2.00E-06 | **CHD** | 0.024 | 0.005 | 5.60E-06 | -0.003 | 0.003 | 0.289 | 3.83E-07 |

**Supplementary Table 8**. Simulation-based type 1 error rates. Shown are the simulation-based type 1 error rates of Approach 1 (adjusted effect), Approach 2 (joint effect), Approach 3 and 4 (interaction effects). For two different MAFs, the approaches were applied to 10,000 simulated stratum-specific effect sizes that were generated under stratum-specific null hypotheses of "no stratum-specific effects" and under the assumption of 50,000 smokers and 180,000 nonsmokers. The type 1 error rates shown reflect the proportion of nominally significant simulation results for the respective approach.

| Approach | MAF | #Variants tested | #Variants with P<0.05 | Type 1 Error Rate [%] |
|---|---|---|---|---|
| **Approach 1** | 0.05 | 10,000 | 515 | 5.15 |
| ***(adjusted effect)*** | 0.3 | 10,000 | 486 | 4.86 |
| **Approach 2** | 0.05 | 10,000 | 492 | 4.92 |
| ***(joint effect)*** | 0.3 | 10,000 | 487 | 4.87 |
| **Approach 3** | 0.05 | 10,000 | 490 | 4.9 |
| ***(Interaction)*** | 0.3 | 10,000 | 514 | 5.14 |
| **Approach 4** | 0.05 | 515 | 27 | 5.24 |
| ***(Interaction)*** | 0.3 | 486 | 22 | 4.53 |

**Supplementary Table 9.** Variance explained in smoking strata for significant variants from Approach 1 (SNPadjSMK) at varying thresholds.

| Trait | Number of SNPs | Approach 1 $P_{SNPadjSMK}$ threshold | Variance smokers | Variance error Smokers | Variance nonsmokers | Variance error Nonsmokers | Difference in Variance (Smokers-Nonsmokers) | Error for difference in Variance | $P_{RsqDiff}$ |
|---|---|---|---|---|---|---|---|---|---|
| BMI | 44 | 1.00E-08 | 0.01840 | 0.00125 | 0.01279 | 0.00054 | 0.00562 | 0.00136 | **3.83E-05** |
| BMI | 50 | 3.16E-08 | 0.01907 | 0.00128 | 0.01368 | 0.00056 | 0.00539 | 0.00139 | **1.11E-04** |
| BMI | 65 | 1.00E-07 | 0.02034 | 0.00133 | 0.01583 | 0.00060 | 0.00451 | 0.00146 | **1.94E-03** |
| BMI | 84 | 3.16E-07 | 0.02374 | 0.00145 | 0.01809 | 0.00065 | 0.00565 | 0.00158 | **3.64E-04** |
| BMI | 105 | 1.00E-06 | 0.02616 | 0.00153 | 0.02068 | 0.00070 | 0.00549 | 0.00168 | **1.12E-03** |
| BMI | 132 | 3.16E-06 | 0.03011 | 0.00166 | 0.02343 | 0.00075 | 0.00668 | 0.00182 | **2.50E-04** |
| BMI | 190 | 1.00E-05 | 0.03797 | 0.00189 | 0.02885 | 0.00084 | 0.00912 | 0.00207 | **1.07E-05** |
| BMI | 271 | 3.16E-05 | 0.04679 | 0.00214 | 0.03568 | 0.00095 | 0.01111 | 0.00234 | **1.99E-06** |
| BMI | 436 | 1.00E-04 | 0.06237 | 0.00253 | 0.04806 | 0.00112 | 0.01431 | 0.00276 | **2.20E-07** |
| BMI | 708 | 3.16E-04 | 0.08633 | 0.00304 | 0.06589 | 0.00133 | 0.02044 | 0.00332 | **7.41E-10** |
| BMI | 1147 | 1.00E-03 | 0.11591 | 0.00361 | 0.09037 | 0.00158 | 0.02555 | 0.00394 | **8.49E-11** |
| BMI | 1841 | 3.16E-03 | 0.15124 | 0.00422 | 0.12235 | 0.00187 | 0.02889 | 0.00462 | **3.87E-10** |
| BMI | 2687 | 1.00E-02 | 0.18356 | 0.00475 | 0.15244 | 0.00211 | 0.03112 | 0.00519 | **2.08E-09** |
| BMI | 3407 | 3.16E-02 | 0.20449 | 0.00510 | 0.17062 | 0.00225 | 0.03387 | 0.00557 | **1.23E-09** |
| BMI | 3793 | 1.00E-01 | 0.21137 | 0.00524 | 0.17642 | 0.00230 | 0.03495 | 0.00571 | **9.62E-10** |
| WCadjBMI | 50 | 1.00E-08 | 0.01430 | 0.00120 | 0.01330 | 0.00060 | 0.00090 | 0.00140 | 4.98E-01 |
| WCadjBMI | 59 | 3.16E-08 | 0.01530 | 0.00130 | 0.01510 | 0.00060 | 0.00020 | 0.00140 | 9.05E-01 |
| WCadjBMI | 69 | 1.00E-07 | 0.01700 | 0.00130 | 0.01670 | 0.00070 | 0.00030 | 0.00150 | 8.38E-01 |
| WCadjBMI | 77 | 3.16E-07 | 0.01850 | 0.00140 | 0.01770 | 0.00070 | 0.00080 | 0.00160 | 6.14E-01 |
| WCadjBMI | 94 | 1.00E-06 | 0.02090 | 0.00150 | 0.02010 | 0.00070 | 0.00080 | 0.00170 | 6.51E-01 |
| WCadjBMI | 115 | 3.16E-06 | 0.02300 | 0.00160 | 0.02300 | 0.00080 | 0.00000 | 0.00180 | 9.87E-01 |
| WCadjBMI | 148 | 1.00E-05 | 0.02670 | 0.00170 | 0.02680 | 0.00090 | -0.00010 | 0.00190 | 9.59E-01 |
| WCadjBMI | 206 | 3.16E-05 | 0.03020 | 0.00190 | 0.03350 | 0.00100 | -0.00330 | 0.00210 | 1.17E-01 |
| WCadjBMI | 322 | 1.00E-04 | 0.03800 | 0.00220 | 0.04490 | 0.00120 | -0.00690 | 0.00250 | 4.91E-03 |
| WCadjBMI | 538 | 3.16E-04 | 0.05050 | 0.00260 | 0.06340 | 0.00140 | -0.01290 | 0.00290 | **1.19E-05** |
| WCadjBMI | 926 | 1.00E-03 | 0.06490 | 0.00300 | 0.09200 | 0.00170 | -0.02710 | 0.00350 | **8.51E-15** |
| WCadjBMI | 1603 | 3.16E-03 | 0.08620 | 0.00360 | 0.13170 | 0.00210 | -0.04550 | 0.00420 | **3.81E-27** |
| WCadjBMI | 2505 | 1.00E-02 | 0.10590 | 0.00420 | 0.17250 | 0.00250 | -0.06650 | 0.00490 | **2.04E-42** |
| WCadjBMI | 3303 | 3.16E-02 | 0.11290 | 0.00450 | 0.19910 | 0.00270 | -0.08620 | 0.00520 | **1.40E-60** |
| WCadjBMI | 3781 | 1.00E-01 | 0.11740 | 0.00470 | 0.20790 | 0.00270 | -0.09050 | 0.00540 | **4.11E-62** |
| WHRadjBMI | 50 | 1.00E-08 | 0.00154 | 0.00049 | 0.00282 | 0.00030 | -0.00128 | 0.00057 | 2.55E-02 |
| WHRadjBMI | 59 | 3.16E-08 | 0.00213 | 0.00056 | 0.00356 | 0.00034 | -0.00144 | 0.00065 | 2.80E-02 |
| WHRadjBMI | 69 | 1.00E-07 | 0.00243 | 0.00060 | 0.00414 | 0.00037 | -0.00172 | 0.00070 | 1.47E-02 |
| WHRadjBMI | 77 | 3.16E-07 | 0.00266 | 0.00063 | 0.00435 | 0.00038 | -0.00169 | 0.00073 | 2.13E-02 |
| WHRadjBMI | 94 | 1.00E-06 | 0.00269 | 0.00065 | 0.00500 | 0.00041 | -0.00231 | 0.00077 | **2.84E-03** |
| WHRadjBMI | 115 | 3.16E-06 | 0.00268 | 0.00069 | 0.00601 | 0.00046 | -0.00333 | 0.00083 | **5.69E-05** |
| WHRadjBMI | 148 | 1.00E-05 | 0.00294 | 0.00075 | 0.00697 | 0.00050 | -0.00404 | 0.00090 | **7.68E-06** |
| WHRadjBMI | 206 | 3.16E-05 | 0.00294 | 0.00083 | 0.00948 | 0.00060 | -0.00654 | 0.00103 | **1.82E-10** |
| WHRadjBMI | 321 | 1.00E-04 | 0.00342 | 0.00101 | 0.01453 | 0.00075 | -0.01110 | 0.00125 | **8.55E-19** |
| WHRadjBMI | 537 | 3.16E-04 | 0.00444 | 0.00128 | 0.02301 | 0.00096 | -0.01858 | 0.00160 | **3.18E-31** |
| WHRadjBMI | 923 | 1.00E-03 | 0.00467 | 0.00162 | 0.03627 | 0.00124 | -0.03160 | 0.00204 | **3.04E-54** |
| WHRadjBMI | 1598 | 3.16E-03 | 0.00529 | 0.00211 | 0.05407 | 0.00154 | -0.04878 | 0.00261 | **8.27E-78** |
| WHRadjBMI | 2496 | 1.00E-02 | 0.00649 | 0.00265 | 0.07207 | 0.00180 | -0.06558 | 0.00320 | **3.57E-93** |
| WHRadjBMI | 3293 | 3.16E-02 | 0.00683 | 0.00304 | 0.08359 | 0.00197 | -0.07676 | 0.00363 | **1.58E-99** |
| WHRadjBMI | 3773 | 1.00E-01 | 0.00654 | 0.00325 | 0.08819 | 0.00204 | -0.08165 | 0.00384 | **1.54E-100** |

**Supplementary Table 10**. Association of cis-acting expression quantitative trait loci (cis-eQTLs) for novel loci using publicly available databases.

| Trait | IndexSNP | Proxy SNP | $r^2$ CEU IndexSNP vs ProxySNP | P ProxySNP | Tissue | Transcript | Best E-SNP | Best E-SNP P | $r^2$ CEU IndexSNP vs BestESNP | PMID |
|---|---|---|---|---|---|---|---|---|---|---|
| BMI | rs12629427 | rs9860797 | 0.905428 | 2.23E-05 | Cerebellum (all samples) | EPHA3 | rs9860797 | 2.23E-05 | 0.865 | 23622250 |
| BMI | rs12629427 | rs9860797 | 0.905428 | 3.71E-05 | Prefrontal cortex (Alzheimer's) | EPHA3 | rs9860797 | 3.71E-05 | 0.865 | 23622250 |
| BMI | rs13069244 | rs13069244 | IndexSNP | 2.09E-05 | Peripheral blood mononuclear cells | TTC14 | rs1921678 | 6.46E-109 | unavailable | 26019233 |
| BMI | rs2481665 | rs2481665 | IndexSNP | 7.31E-06 | Prefrontal cortex (Huntington's) | INADL | rs2481665 | 7.31E-06 | SameSNP | 23622250 |
| WCadjBMI | rs10269774 | rs2282978 | 0.920627 | 2.62E-05 | Cerebellum (normal samples) | CDK6 | rs2282978 | 2.62E-05 | 1 | 23622250 |
| WCadjBMI | rs17396340 | rs17396340 | IndexSNP | 1.85E-15 | B-lymphoblastoid cell lines (collected at age 8) | ENSG00000054523 | rs17396340 | 1.85E-15 | SameSNP | 25010687 |
| WCadjBMI | rs17396340 | rs17396340 | IndexSNP | 8.27E-118 | Peripheral blood mononuclear cells | KIF1B | rs17396340 | 8.27E-118 | SameSNP | 26019233 |
| WCadjBMI | rs17396340 | rs17396340 | IndexSNP | 1.21E-07 | Peripheral blood mononuclear cells | KIF1B | rs17396340 | 8.27E-118 | SameSNP | 26019233 |
| WCadjBMI | rs17396340 | rs17396340 | IndexSNP | 3.85E-06 | Whole blood (PAX) in individuals without asthma | KIF1B | rs17396340 | 3.85E-06 | SameSNP | 25816334 |
| WCadjBMI | rs17396340 | rs17396340 | IndexSNP | 9.81E-198 | Whole blood (CHARGE) | KIF1B | rs12139158 | 9.81E-198 | 1 | 24013639 |
| WCadjBMI | rs17396340 | rs17396340 | IndexSNP | 2.64E-11 | Whole blood (CHARGE) | KIF1B | rs12139158 | 9.81E-198 | 1 | 24013639 |
| WCadjBMI | rs17396340 | rs17396340 | IndexSNP | 9.01E-29 | LCL (MuTHER) | KIF1B | rs6693965 | 7.66E-29 | 0.92 | 22941192 |
| WCadjBMI | rs17396340 | rs17397129 | 1 | 4.64E-113 | Peripheral blood mononuclear cells | KIF1B | rs17396340 | 8.27E-118 | SameSNP | 26019233 |
| WCadjBMI | rs17396340 | rs17397129 | 1 | 3.32E-07 | Peripheral blood mononuclear cells | KIF1B | rs17396340 | 8.27E-118 | SameSNP | 26019233 |
| WCadjBMI | rs17396340 | rs17397129 | 1 | 9.81E-198 | Whole blood (CHARGE) | KIF1B | rs12139158 | 9.81E-198 | 1 | 24013639 |
| WCadjBMI | rs17396340 | rs17397129 | 1 | 6.99E-10 | Whole blood (CHARGE) | KIF1B | rs12139158 | 9.81E-198 | 1 | 24013639 |
| WCadjBMI | rs17396340 | rs17397129 | 1 | 1.08E-26 | LCL (MuTHER) | KIF1B | rs6693965 | 7.66E-29 | 0.92 | 22941192 |
| WCadjBMI | rs4378999 | rs10212250 | 0.994018 | 1.62E-03 | Peripheral blood mononuclear cells | TEX264 | rs11130274 | 3.18E-04 | 1 | 26019233 |
| WCadjBMI | rs4378999 | rs10212250 | 0.994018 | 5.42E-14 | CD14+ monocytes (24h LPS stimulated) | MAPKAPK3 | rs7614881 | 9.39E-18 | 0.8 | 24604202 |
| WCadjBMI | rs4378999 | rs10212250 | 0.994018 | 1.66E-04 | CD14+ monocytes (2h LPS stimulated) | MAPKAPK3 | rs7614881 | 7.37E-06 | 0.8 | 24604202 |
| WCadjBMI | rs4378999 | rs10212250 | 0.994018 | 2.26E-04 | CD14+ monocytes (2h LPS stimulated) | TEX264 | rs1055429 | 8.02E-07 | 0.8 | 24604202 |
| WCadjBMI | rs4378999 | rs10212250 | 0.994018 | 1.02E-24 | Peripheral blood mononuclear cells | GRM2 | rs1055429 | 5.80E-35 | 0.8 | 26019233 |
| WCadjBMI | rs4378999 | rs11130274 | 0.988059 | 2.39E-06 | Liver(Greenawalt) | AK093691 | rs11130274 | 2.39E-06 | 1 | 21602305 |
| WCadjBMI | rs4378999 | rs11130274 | 0.988059 | 3.18E-04 | Peripheral blood mononuclear cells | TEX264 | rs11130274 | 3.18E-04 | 1 | 26019233 |
| WCadjBMI | rs4378999 | rs11130274 | 0.988059 | 9.28E-08 | Prefrontal cortex (all samples) | C3orf18 | rs11130274 | 9.28E-08 | 1 | 23622250 |
| WCadjBMI | rs4378999 | rs11130274 | 0.988059 | 1.13E-25 | Peripheral blood mononuclear cells | GRM2 | rs1055429 | 5.80E-35 | 0.8 | 26019233 |
| WCadjBMI | rs4378999 | rs11130274 | 0.988059 | 3.69E-05 | Subc adipose (MuTHER) | ARMET | rs6784455 | 3.27E-06 | 0.8 | 22941192 |
| WCadjBMI | rs4378999 | rs11130282 | 0.994018 | 1.62E-03 | Peripheral blood mononuclear cells | TEX264 | rs11130274 | 3.18E-04 | 1 | 26019233 |
| WCadjBMI | rs4378999 | rs11130282 | 0.994018 | 1.02E-24 | Peripheral blood mononuclear cells | GRM2 | rs1055429 | 5.80E-35 | 0.8 | 26019233 |
| WCadjBMI | rs4378999 | rs11919932 | 0.904684 | 2.48E-12 | Peripheral blood mononuclear cells | GRM2 | rs1055429 | 5.80E-35 | 0.8 | 26019233 |
| WCadjBMI | rs4378999 | rs13324500 | 0.904684 | 2.48E-12 | Peripheral blood mononuclear cells | GRM2 | rs1055429 | 5.80E-35 | 0.8 | 26019233 |
| WCadjBMI | rs4378999 | rs1383252 | 0.994018 | 1.63E-03 | Peripheral blood mononuclear cells | TEX264 | rs11130274 | 3.18E-04 | 1 | 26019233 |
| WCadjBMI | rs4378999 | rs1383252 | 0.994018 | 1.03E-24 | Peripheral blood mononuclear cells | GRM2 | rs1055429 | 5.80E-35 | 0.8 | 26019233 |
| WCadjBMI | rs4378999 | rs1480357 | 0.964819 | 1.81E-03 | Peripheral blood mononuclear cells | TEX264 | rs11130274 | 3.18E-04 | 1 | 26019233 |
| WCadjBMI | rs4378999 | rs1480357 | 0.964819 | 1.29E-24 | Peripheral blood mononuclear cells | GRM2 | rs1055429 | 5.80E-35 | 0.8 | 26019233 |
| WCadjBMI | rs4378999 | rs1480358 | 0.994018 | 1.81E-03 | Peripheral blood mononuclear cells | TEX264 | rs11130274 | 3.18E-04 | 1 | 26019233 |
| WCadjBMI | rs4378999 | rs1480358 | 0.994018 | 8.53E-11 | Monocytes | MAPKAPK3 | rs6779819 | 1.47E-16 | 0.8 | 20502693 |
| WCadjBMI | rs4378999 | rs1480358 | 0.994018 | 1.30E-24 | Peripheral blood mononuclear cells | GRM2 | rs1055429 | 5.80E-35 | 0.8 | 26019233 |
| WCadjBMI | rs4378999 | rs1480363 | 0.994018 | 1.63E-03 | Peripheral blood mononuclear cells | TEX264 | rs11130274 | 3.18E-04 | 1 | 26019233 |
| WCadjBMI | rs4378999 | rs1480363 | 0.994018 | 1.03E-24 | Peripheral blood mononuclear cells | GRM2 | rs1055429 | 5.80E-35 | 0.8 | 26019233 |
| WCadjBMI | rs4378999 | rs1552072 | 0.994018 | 1.81E-03 | Peripheral blood mononuclear cells | TEX264 | rs11130274 | 3.18E-04 | 1 | 26019233 |
| WCadjBMI | rs4378999 | rs1552072 | 0.994018 | 1.30E-24 | Peripheral blood mononuclear cells | GRM2 | rs1055429 | 5.80E-35 | 0.8 | 26019233 |
| WCadjBMI | rs4378999 | rs2061951 | 0.994018 | 1.63E-03 | Peripheral blood mononuclear cells | TEX264 | rs11130274 | 3.18E-04 | 1 | 26019233 |
| WCadjBMI | rs4378999 | rs2061951 | 0.994018 | 1.03E-24 | Peripheral blood mononuclear cells | GRM2 | rs1055429 | 5.80E-35 | 0.8 | 26019233 |
| WCadjBMI | rs4378999 | rs2355941 | 0.994018 | 1.63E-03 | Peripheral blood mononuclear cells | TEX264 | rs11130274 | 3.18E-04 | 1 | 26019233 |
| WCadjBMI | rs4378999 | rs2355941 | 0.994018 | 1.03E-24 | Peripheral blood mononuclear cells | GRM2 | rs1055429 | 5.80E-35 | 0.8 | 26019233 |
| WCadjBMI | rs4378999 | rs4244696 | 0.904684 | 2.46E-12 | Peripheral blood mononuclear cells | GRM2 | rs1055429 | 5.80E-35 | 0.8 | 26019233 |
| WCadjBMI | rs4378999 | rs4244697 | 1 | 9.21E-04 | Peripheral blood mononuclear cells | TEX264 | rs11130274 | 3.18E-04 | 1 | 26019233 |
| WCadjBMI | rs4378999 | rs4244697 | 1 | 1.76E-25 | Peripheral blood mononuclear cells | GRM2 | rs1055429 | 5.80E-35 | 0.8 | 26019233 |

| | | | | | | | | | | |
|---|---|---|---|---|---|---|---|---|---|---|
| WCadjBMI | rs4378999 | rs4244704 | 0.904684 | 2.47E-12 | Peripheral blood mononuclear cells | GRM2 | rs1055429 | 5.80E-35 | 0.8 | 26019233 |
| WCadjBMI | rs4378999 | rs4263323 | 0.904684 | 2.48E-12 | Peripheral blood mononuclear cells | GRM2 | rs1055429 | 5.80E-35 | 0.8 | 26019233 |
| WCadjBMI | rs4378999 | rs4286453 | 0.904684 | 1.89E-12 | Peripheral blood mononuclear cells | GRM2 | rs1055429 | 5.80E-35 | 0.8 | 26019233 |
| WCadjBMI | rs4378999 | rs4301044 | 0.988059 | 1.63E-03 | Peripheral blood mononuclear cells | TEX264 | rs11130274 | 3.18E-04 | 1 | 26019233 |
| WCadjBMI | rs4378999 | rs4301044 | 0.988059 | 1.02E-24 | Peripheral blood mononuclear cells | GRM2 | rs1055429 | 5.80E-35 | 0.8 | 26019233 |
| WCadjBMI | rs4378999 | rs4328835 | 0.988059 | 7.96E-26 | Peripheral blood mononuclear cells | GRM2 | rs1055429 | 5.80E-35 | 0.8 | 26019233 |
| WCadjBMI | rs4378999 | rs4328835 | 0.988059 | 4.00E-05 | Subc adipose (MuTHER) | ARMET | rs6784455 | 3.27E-06 | 0.8 | 22941192 |
| WCadjBMI | rs4378999 | rs4367101 | 0.994018 | 1.62E-03 | Peripheral blood mononuclear cells | TEX264 | rs11130274 | 3.18E-04 | 1 | 26019233 |
| WCadjBMI | rs4378999 | rs4367101 | 0.994018 | 1.01E-24 | Peripheral blood mononuclear cells | GRM2 | rs1055429 | 5.80E-35 | 0.8 | 26019233 |
| WCadjBMI | rs4378999 | rs4373098 | 0.904684 | 2.48E-12 | Peripheral blood mononuclear cells | GRM2 | rs1055429 | 5.80E-35 | 0.8 | 26019233 |
| WCadjBMI | rs4378999 | rs4377512 | 0.904684 | 2.54E-11 | Monocytes | MAPKAPK3 | rs6779819 | 1.47E-16 | 0.8 | 20502693 |
| WCadjBMI | rs4378999 | rs4377512 | 0.904684 | 2.48E-12 | Peripheral blood mononuclear cells | GRM2 | rs1055429 | 5.80E-35 | 0.8 | 26019233 |
| WCadjBMI | rs4378999 | rs4378999 | IndexSNP | 9.15E-04 | Peripheral blood mononuclear cells | TEX264 | rs11130274 | 3.18E-04 | 1 | 26019233 |
| WCadjBMI | rs4378999 | rs4378999 | IndexSNP | 1.72E-25 | Peripheral blood mononuclear cells | GRM2 | rs1055429 | 5.80E-35 | 0.8 | 26019233 |
| WCadjBMI | rs4378999 | rs4446244 | 0.904684 | 2.47E-12 | Peripheral blood mononuclear cells | GRM2 | rs1055429 | 5.80E-35 | 0.8 | 26019233 |
| WCadjBMI | rs4378999 | rs4453876 | 0.994018 | 1.62E-03 | Peripheral blood mononuclear cells | TEX264 | rs11130274 | 3.18E-04 | 1 | 26019233 |
| WCadjBMI | rs4378999 | rs4453876 | 0.994018 | 1.02E-24 | Peripheral blood mononuclear cells | GRM2 | rs1055429 | 5.80E-35 | 0.8 | 26019233 |
| WCadjBMI | rs4378999 | rs4478114 | 0.904684 | 4.76E-12 | Monocytes | MAPKAPK3 | rs6779819 | 1.47E-16 | 0.8 | 20502693 |
| WCadjBMI | rs4378999 | rs4478114 | 0.904684 | 2.46E-12 | Peripheral blood mononuclear cells | GRM2 | rs1055429 | 5.80E-35 | 0.8 | 26019233 |
| WCadjBMI | rs4378999 | rs4505743 | 0.904684 | 2.48E-12 | Peripheral blood mononuclear cells | GRM2 | rs1055429 | 5.80E-35 | 0.8 | 26019233 |
| WCadjBMI | rs4378999 | rs4511933 | 0.904684 | 2.48E-12 | Peripheral blood mononuclear cells | GRM2 | rs1055429 | 5.80E-35 | 0.8 | 26019233 |
| WCadjBMI | rs4378999 | rs4528983 | 1 | 1.62E-03 | Peripheral blood mononuclear cells | TEX264 | rs11130274 | 3.18E-04 | 1 | 26019233 |
| WCadjBMI | rs4378999 | rs4528983 | 1 | 1.01E-24 | Peripheral blood mononuclear cells | GRM2 | rs1055429 | 5.80E-35 | 0.8 | 26019233 |
| WCadjBMI | rs4378999 | rs4530566 | 0.904684 | 6.43E-11 | Monocytes | MAPKAPK3 | rs6779819 | 1.47E-16 | 0.8 | 20502693 |
| WCadjBMI | rs4378999 | rs4530566 | 0.904684 | 2.48E-12 | Peripheral blood mononuclear cells | GRM2 | rs1055429 | 5.80E-35 | 0.8 | 26019233 |
| WCadjBMI | rs4378999 | rs4532165 | 0.988059 | 3.20E-04 | Peripheral blood mononuclear cells | TEX264 | rs11130274 | 3.18E-04 | 1 | 26019233 |
| WCadjBMI | rs4378999 | rs4532165 | 0.988059 | 1.05E-25 | Peripheral blood mononuclear cells | GRM2 | rs1055429 | 5.80E-35 | 0.8 | 26019233 |
| WCadjBMI | rs4378999 | rs4532165 | 0.988059 | 3.89E-05 | Subc adipose (MuTHER) | ARMET | rs6784455 | 3.27E-06 | 0.8 | 22941192 |
| WCadjBMI | rs4378999 | rs4555554 | 0.904684 | 2.48E-12 | Peripheral blood mononuclear cells | GRM2 | rs1055429 | 5.80E-35 | 0.8 | 26019233 |
| WCadjBMI | rs4378999 | rs4568177 | 0.994018 | 1.62E-03 | Peripheral blood mononuclear cells | TEX264 | rs11130274 | 3.18E-04 | 1 | 26019233 |
| WCadjBMI | rs4378999 | rs4568177 | 0.994018 | 1.02E-24 | Peripheral blood mononuclear cells | GRM2 | rs1055429 | 5.80E-35 | 0.8 | 26019233 |
| WCadjBMI | rs4378999 | rs4582086 | 0.904684 | 2.48E-12 | Peripheral blood mononuclear cells | GRM2 | rs1055429 | 5.80E-35 | 0.8 | 26019233 |
| WCadjBMI | rs4378999 | rs4589968 | 0.904684 | 2.46E-12 | Peripheral blood mononuclear cells | GRM2 | rs1055429 | 5.80E-35 | 0.8 | 26019233 |
| WCadjBMI | rs4378999 | rs4615113 | 0.904684 | 2.48E-12 | Peripheral blood mononuclear cells | GRM2 | rs1055429 | 5.80E-35 | 0.8 | 26019233 |
| WCadjBMI | rs4378999 | rs4927978 | 0.904684 | 2.47E-12 | Peripheral blood mononuclear cells | GRM2 | rs1055429 | 5.80E-35 | 0.8 | 26019233 |
| WCadjBMI | rs4378999 | rs4927983 | 0.904684 | 2.46E-12 | Peripheral blood mononuclear cells | GRM2 | rs1055429 | 5.80E-35 | 0.8 | 26019233 |
| WCadjBMI | rs4378999 | rs4974095 | 0.982324 | 1.81E-03 | Peripheral blood mononuclear cells | TEX264 | rs11130274 | 3.18E-04 | 1 | 26019233 |
| WCadjBMI | rs4378999 | rs4974095 | 0.982324 | 1.30E-24 | Peripheral blood mononuclear cells | GRM2 | rs1055429 | 5.80E-35 | 0.8 | 26019233 |
| WCadjBMI | rs4378999 | rs4974109 | 0.976321 | 1.81E-03 | Peripheral blood mononuclear cells | TEX264 | rs11130274 | 3.18E-04 | 1 | 26019233 |
| WCadjBMI | rs4378999 | rs4974109 | 0.976321 | 1.30E-24 | Peripheral blood mononuclear cells | GRM2 | rs1055429 | 5.80E-35 | 0.8 | 26019233 |
| WCadjBMI | rs4378999 | rs6445455 | 0.988059 | 5.42E-04 | Peripheral blood mononuclear cells | TEX264 | rs11130274 | 3.18E-04 | 1 | 26019233 |
| WCadjBMI | rs4378999 | rs6445455 | 0.988059 | 4.79E-26 | Peripheral blood mononuclear cells | GRM2 | rs1055429 | 5.80E-35 | 0.8 | 26019233 |
| WCadjBMI | rs4378999 | rs6445455 | 0.988059 | 4.15E-05 | Subc adipose (MuTHER) | ARMET | rs6784455 | 3.27E-06 | 0.8 | 22941192 |
| WCadjBMI | rs4378999 | rs6445551 | 0.994018 | 1.63E-03 | Peripheral blood mononuclear cells | TEX264 | rs11130274 | 3.18E-04 | 1 | 26019233 |
| WCadjBMI | rs4378999 | rs6445551 | 0.994018 | 1.03E-24 | Peripheral blood mononuclear cells | GRM2 | rs1055429 | 5.80E-35 | 0.8 | 26019233 |
| WCadjBMI | rs4378999 | rs6445600 | 0.904684 | 2.91E-12 | Monocytes | MAPKAPK3 | rs6779819 | 1.47E-16 | 0.8 | 20502693 |
| WCadjBMI | rs4378999 | rs6445600 | 0.904684 | 2.48E-12 | Peripheral blood mononuclear cells | GRM2 | rs1055429 | 5.80E-35 | 0.8 | 26019233 |
| WCadjBMI | rs4378999 | rs6445717 | 0.904684 | 2.48E-12 | Peripheral blood mononuclear cells | GRM2 | rs1055429 | 5.80E-35 | 0.8 | 26019233 |
| WCadjBMI | rs4378999 | rs6445750 | 0.904684 | 4.68E-12 | Monocytes | MAPKAPK3 | rs6779819 | 1.47E-16 | 0.8 | 20502693 |
| WCadjBMI | rs4378999 | rs6445750 | 0.904684 | 2.48E-12 | Peripheral blood mononuclear cells | GRM2 | rs1055429 | 5.80E-35 | 0.8 | 26019233 |
| WCadjBMI | rs4378999 | rs6445779 | 0.904684 | 2.47E-12 | Peripheral blood mononuclear cells | GRM2 | rs1055429 | 5.80E-35 | 0.8 | 26019233 |
| WCadjBMI | rs4378999 | rs6446011 | 0.904684 | 2.46E-12 | Peripheral blood mononuclear cells | GRM2 | rs1055429 | 5.80E-35 | 0.8 | 26019233 |
| WCadjBMI | rs4378999 | rs6446118 | 0.904684 | 2.46E-12 | Peripheral blood mononuclear cells | GRM2 | rs1055429 | 5.80E-35 | 0.8 | 26019233 |

| | | | | | | | | | | |
|---|---|---|---|---|---|---|---|---|---|---|
| WCadjBMI | rs4378999 | rs6446167 | 0.904684 | 5.86E-12 | Monocytes | MAPKAPK3 | rs6779819 | 1.47E-16 | 0.8 | 20502693 |
| WCadjBMI | rs4378999 | rs6446167 | 0.904684 | 2.46E-12 | Peripheral blood mononuclear cells | GRM2 | rs1055429 | 5.80E-35 | 0.8 | 26019233 |
| WCadjBMI | rs4378999 | rs6768601 | 0.904684 | 3.84E-12 | Monocytes | MAPKAPK3 | rs6779819 | 1.47E-16 | 0.8 | 20502693 |
| WCadjBMI | rs4378999 | rs6768601 | 0.904684 | 2.48E-12 | Peripheral blood mononuclear cells | GRM2 | rs1055429 | 5.80E-35 | 0.8 | 26019233 |
| WCadjBMI | rs4378999 | rs6772664 | 1 | 1.62E-03 | Peripheral blood mononuclear cells | TEX264 | rs11130274 | 3.18E-04 | 1 | 26019233 |
| WCadjBMI | rs4378999 | rs6772664 | 1 | 1.01E-24 | Peripheral blood mononuclear cells | GRM2 | rs1055429 | 5.80E-35 | 0.8 | 26019233 |
| WCadjBMI | rs4378999 | rs6777719 | 0.988059 | 8.54E-06 | Lung | TEX264 | rs6777719 | 8.54E-06 | 1 | 23209423 |
| WCadjBMI | rs4378999 | rs6777719 | 0.988059 | 5.46E-04 | Peripheral blood mononuclear cells | TEX264 | rs11130274 | 3.18E-04 | 1 | 26019233 |
| WCadjBMI | rs4378999 | rs6777719 | 0.988059 | 4.84E-26 | Peripheral blood mononuclear cells | GRM2 | rs1055429 | 5.80E-35 | 0.8 | 26019233 |
| WCadjBMI | rs4378999 | rs6777719 | 0.988059 | 4.17E-05 | Subc adipose (MuTHER) | ARMET | rs6784455 | 3.27E-06 | 0.8 | 22941192 |
| WCadjBMI | rs4378999 | rs6779658 | 1 | 1.51E-03 | Peripheral blood mononuclear cells | TEX264 | rs11130274 | 3.18E-04 | 1 | 26019233 |
| WCadjBMI | rs4378999 | rs6779658 | 1 | 4.12E-25 | Peripheral blood mononuclear cells | GRM2 | rs1055429 | 5.80E-35 | 0.8 | 26019233 |
| WCadjBMI | rs4378999 | rs6793613 | 0.904684 | 2.46E-12 | Peripheral blood mononuclear cells | GRM2 | rs1055429 | 5.80E-35 | 0.8 | 26019233 |
| WCadjBMI | rs4378999 | rs6809080 | 0.994018 | 1.63E-03 | Peripheral blood mononuclear cells | TEX264 | rs11130274 | 3.18E-04 | 1 | 26019233 |
| WCadjBMI | rs4378999 | rs6809080 | 0.994018 | 1.02E-24 | Peripheral blood mononuclear cells | GRM2 | rs1055429 | 5.80E-35 | 0.8 | 26019233 |
| WCadjBMI | rs4378999 | rs7611873 | 0.904684 | 2.47E-12 | Peripheral blood mononuclear cells | GRM2 | rs1055429 | 5.80E-35 | 0.8 | 26019233 |
| WCadjBMI | rs4378999 | rs7614171 | 0.904684 | 2.48E-12 | Peripheral blood mononuclear cells | GRM2 | rs1055429 | 5.80E-35 | 0.8 | 26019233 |
| WCadjBMI | rs4378999 | rs7615291 | 0.904684 | 3.19E-12 | Monocytes | MAPKAPK3 | rs6779819 | 1.47E-16 | 0.8 | 20502693 |
| WCadjBMI | rs4378999 | rs7615291 | 0.904684 | 2.47E-12 | Peripheral blood mononuclear cells | GRM2 | rs1055429 | 5.80E-35 | 0.8 | 26019233 |
| WCadjBMI | rs4378999 | rs7619584 | 0.994018 | 1.62E-03 | Peripheral blood mononuclear cells | TEX264 | rs11130274 | 3.18E-04 | 1 | 26019233 |
| WCadjBMI | rs4378999 | rs7619584 | 0.994018 | 1.02E-24 | Peripheral blood mononuclear cells | GRM2 | rs1055429 | 5.80E-35 | 0.8 | 26019233 |
| WCadjBMI | rs4378999 | rs7628533 | 0.994018 | 5.42E-14 | CD14+ monocytes (24h LPS stimulated) | MAPKAPK3 | rs7614881 | 9.39E-18 | 0.8 | 24604202 |
| WCadjBMI | rs4378999 | rs7628533 | 0.994018 | 1.66E-04 | CD14+ monocytes (2h LPS stimulated) | MAPKAPK3 | rs7614881 | 7.37E-06 | 0.8 | 24604202 |
| WCadjBMI | rs4378999 | rs7628533 | 0.994018 | 2.26E-04 | CD14+ monocytes (2h LPS stimulated) | TEX264 | rs1055429 | 8.02E-07 | 0.8 | 24604202 |
| WCadjBMI | rs4378999 | rs7628533 | 0.994018 | 1.45E-12 | Peripheral blood mononuclear cells | GRM2 | rs1055429 | 5.80E-35 | 0.8 | 26019233 |
| WCadjBMI | rs4378999 | rs7630730 | 0.982324 | 9.64E-04 | Peripheral blood mononuclear cells | TEX264 | rs11130274 | 3.18E-04 | 1 | 26019233 |
| WCadjBMI | rs4378999 | rs7630730 | 0.982324 | 9.13E-22 | Peripheral blood mononuclear cells | GRM2 | rs1055429 | 5.80E-35 | 0.8 | 26019233 |
| WCadjBMI | rs4378999 | rs7630730 | 0.982324 | 3.88E-05 | Subc adipose (MuTHER) | ARMET | rs6784455 | 3.27E-06 | 0.8 | 22941192 |
| WCadjBMI | rs4378999 | rs7640263 | 0.904684 | 2.46E-12 | Peripheral blood mononuclear cells | GRM2 | rs1055429 | 5.80E-35 | 0.8 | 26019233 |
| WCadjBMI | rs4378999 | rs7644957 | 0.988059 | 1.63E-03 | Peripheral blood mononuclear cells | TEX264 | rs11130274 | 3.18E-04 | 1 | 26019233 |
| WCadjBMI | rs4378999 | rs7644957 | 0.988059 | 1.02E-24 | Peripheral blood mononuclear cells | GRM2 | rs1055429 | 5.80E-35 | 0.8 | 26019233 |
| WCadjBMI | rs4378999 | rs7652655 | 0.904684 | 2.46E-12 | Peripheral blood mononuclear cells | GRM2 | rs1055429 | 5.80E-35 | 0.8 | 26019233 |
| WCadjBMI | rs4378999 | rs7652818 | 0.904684 | 2.46E-12 | Peripheral blood mononuclear cells | GRM2 | rs1055429 | 5.80E-35 | 0.8 | 26019233 |
| WCadjBMI | rs4378999 | rs7653451 | 0.904684 | 7.37E-16 | CD14+ monocytes (24h LPS stimulated) | MAPKAPK3 | rs7614881 | 9.39E-18 | 0.8 | 24604202 |
| WCadjBMI | rs4378999 | rs7653451 | 0.904684 | 2.93E-05 | CD14+ monocytes (2h LPS stimulated) | MAPKAPK3 | rs7614881 | 7.37E-06 | 0.8 | 24604202 |
| WCadjBMI | rs4378999 | rs7653451 | 0.904684 | 2.20E-04 | CD14+ monocytes (2h LPS stimulated) | TEX264 | rs1055429 | 8.02E-07 | 0.8 | 24604202 |
| WCadjBMI | rs4378999 | rs7653451 | 0.904684 | 3.68E-09 | Peripheral blood mononuclear cells | GRM2 | rs1055429 | 5.80E-35 | 0.8 | 26019233 |
| WCadjBMI | rs4378999 | rs7653451 | 0.904684 | 2.79E-05 | Whole blood (Battle) | ACY1 | rs7653451 | 2.79E-05 | 0.8 | 24092820 |
| WCadjBMI | rs4378999 | rs922612 | 0.994018 | 1.63E-03 | Peripheral blood mononuclear cells | TEX264 | rs11130274 | 3.18E-04 | 1 | 26019233 |
| WCadjBMI | rs4378999 | rs922612 | 0.994018 | 1.03E-24 | Peripheral blood mononuclear cells | GRM2 | rs1055429 | 5.80E-35 | 0.8 | 26019233 |
| WCadjBMI | rs4378999 | rs9682672 | 0.904684 | 2.48E-12 | Peripheral blood mononuclear cells | GRM2 | rs1055429 | 5.80E-35 | 0.8 | 26019233 |
| WCadjBMI | rs4378999 | rs9713241 | 0.900438 | 2.48E-12 | Peripheral blood mononuclear cells | GRM2 | rs1055429 | 5.80E-35 | 0.8 | 26019233 |
| WCadjBMI | rs4378999 | rs9758600 | 0.904684 | 2.48E-12 | Peripheral blood mononuclear cells | GRM2 | rs1055429 | 5.80E-35 | 0.8 | 26019233 |
| WCadjBMI | rs4378999 | rs9828200 | 0.904684 | 3.90E-12 | Monocytes | MAPKAPK3 | rs6779819 | 1.47E-16 | 0.8 | 20502693 |
| WCadjBMI | rs4378999 | rs9828200 | 0.904684 | 2.47E-12 | Peripheral blood mononuclear cells | GRM2 | rs1055429 | 5.80E-35 | 0.8 | 26019233 |
| WCadjBMI | rs4378999 | rs9855442 | 0.994018 | 1.81E-03 | Peripheral blood mononuclear cells | TEX264 | rs11130274 | 3.18E-04 | 1 | 26019233 |
| WCadjBMI | rs4378999 | rs9855442 | 0.994018 | 1.30E-24 | Peripheral blood mononuclear cells | GRM2 | rs1055429 | 5.80E-35 | 0.8 | 26019233 |
| WCadjBMI | rs4378999 | rs9855454 | 0.988049 | 1.78E-12 | Peripheral blood mononuclear cells | GRM2 | rs1055429 | 5.80E-35 | 0.8 | 26019233 |
| WCadjBMI | rs4378999 | rs9858412 | 0.988059 | 1.63E-03 | Peripheral blood mononuclear cells | TEX264 | rs11130274 | 3.18E-04 | 1 | 26019233 |
| WCadjBMI | rs4378999 | rs9858412 | 0.988059 | 1.02E-24 | Peripheral blood mononuclear cells | GRM2 | rs1055429 | 5.80E-35 | 0.8 | 26019233 |
| WCadjBMI | rs4378999 | rs9869123 | 0.988059 | 1.63E-03 | Peripheral blood mononuclear cells | TEX264 | rs11130274 | 3.18E-04 | 1 | 26019233 |
| WCadjBMI | rs4378999 | rs9869123 | 0.988059 | 1.02E-24 | Peripheral blood mononuclear cells | GRM2 | rs1055429 | 5.80E-35 | 0.8 | 26019233 |
| WCadjBMI | rs4378999 | rs9871539 | 0.988059 | 9.65E-04 | Peripheral blood mononuclear cells | TEX264 | rs11130274 | 3.18E-04 | 1 | 26019233 |

| | | | | | | | | | | |
|---|---|---|---|---|---|---|---|---|---|---|
| WCadjBMI | rs4378999 | rs9871539 | 0.988059 | 8.85E-22 | Peripheral blood mononuclear cells | GRM2 | rs1055429 | 5.80E-35 | 0.8 | 26019233 |
| WCadjBMI | rs4378999 | rs9871539 | 0.988059 | 3.89E-05 | Subc adipose (MuTHER) | ARMET | rs6784455 | 3.27E-06 | 0.8 | 22941192 |
| WCadjBMI | rs4378999 | rs9878100 | 0.994018 | 1.81E-03 | Peripheral blood mononuclear cells | TEX264 | rs11130274 | 3.18E-04 | 1 | 26019233 |
| WCadjBMI | rs4378999 | rs9878100 | 0.994018 | 1.30E-24 | Peripheral blood mononuclear cells | GRM2 | rs1055429 | 5.80E-35 | 0.8 | 26019233 |
| WCadjBMI | rs4378999 | rs9883739 | 0.98216 | 1.63E-03 | Peripheral blood mononuclear cells | TEX264 | rs11130274 | 3.18E-04 | 1 | 26019233 |
| WCadjBMI | rs4378999 | rs9883739 | 0.98216 | 1.02E-24 | Peripheral blood mononuclear cells | GRM2 | rs1055429 | 5.80E-35 | 0.8 | 26019233 |
| WCadjBMI | rs6012558 | rs6012558 | IndexSNP | 7.46E-05 | CD14+ monocytes (IFNg stimulated) | DDX27 | rs6019512 | 2.56E-05 | 0.87 | 24604202 |
| WCadjBMI | rs6743226 | rs4675966 | 0.975484 | 6.19E-06 | Blood | FARP2 | rs4675966 | 6.19E-06 | 0.87 | 18344981 |
| WCadjBMI | rs6743226 | rs4675966 | 0.975484 | 4.02E-07 | Lymph | HDLBP | rs4675966 | 4.02E-07 | 0.87 | 17873875 |
| WCadjBMI | rs6743226 | rs4675966 | 0.975484 | 5.05E-05 | Omental adipose | GAL3ST2 | rs4675966 | 5.05E-05 | 0.87 | 21602305 |
| WCadjBMI | rs6743226 | rs6747036 | 0.952557 | 1.37E-06 | LCL (GenCord) | ENSG00000115677.12 | rs6747036 | 1.37E-06 | 0.84 | 23755361 |
| WCadjBMI | rs6743226 | rs6747036 | 0.952557 | 3.41E-07 | Primary PHA-stimulated T cells (GenCord) | ENSG00000115677.12 | rs6747036 | 3.41E-07 | 0.84 | 23755361 |
| WHRadjBMI | rs1049281 | rs1049281 | IndexSNP | 2.23E-308 | Peripheral blood mononuclear cells | HLA-C | rs1049281 | 2.23E-308 | SameSNP | 26019233 |
| WHRadjBMI | rs1049281 | rs2844622 | 0.907829 | 1.53E-284 | Peripheral blood mononuclear cells | HLA-C | rs1049281 | 2.23E-308 | SameSNP | 26019233 |
| WHRadjBMI | rs1049281 | rs7767581 | 0.935004 | 2.23E-308 | Peripheral blood mononuclear cells | HLA-C | rs1049281 | 2.23E-308 | SameSNP | 26019233 |
| WHRadjBMI | rs1049281 | rs9264594 | 0.912386 | 2.25E-233 | Peripheral blood mononuclear cells | HLA-C | rs1049281 | 2.23E-308 | SameSNP | 26019233 |
| WHRadjBMI | rs1049281 | rs9264594 | 0.912386 | 5.92E-05 | Peripheral blood mononuclear cells | HLA-C | rs1049281 | 2.23E-308 | SameSNP | 26019233 |
| WHRadjBMI | rs1049281 | rs9264601 | 0.96172 | 1.65E-233 | Peripheral blood mononuclear cells | HLA-C | rs1049281 | 2.23E-308 | SameSNP | 26019233 |
| WHRadjBMI | rs1049281 | rs9264601 | 0.96172 | 6.16E-05 | Peripheral blood mononuclear cells | HLA-C | rs1049281 | 2.23E-308 | SameSNP | 26019233 |
| WHRadjBMI | rs1049281 | rs9264602 | 0.986425 | 2.23E-308 | Peripheral blood mononuclear cells | HLA-C | rs1049281 | 2.23E-308 | SameSNP | 26019233 |
| WHRadjBMI | rs1049281 | rs9264602 | 0.986425 | <6.7E-45 | Whole blood (Battle) | HLA-L | rs9264602 | <6.7E-45 | 0.967 | 24092820 |
| WHRadjBMI | rs1049281 | rs9264603 | 0.971449 | 2.23E-308 | Peripheral blood mononuclear cells | HLA-C | rs1049281 | 2.23E-308 | SameSNP | 26019233 |
| WHRadjBMI | rs1049281 | rs9264604 | 0.918434 | 2.59E-279 | Peripheral blood mononuclear cells | HLA-C | rs1049281 | 2.23E-308 | SameSNP | 26019233 |
| WHRadjBMI | rs1049281 | rs9264606 | 0.974001 | 2.23E-308 | Peripheral blood mononuclear cells | HLA-C | rs1049281 | 2.23E-308 | SameSNP | 26019233 |
| WHRadjBMI | rs1049281 | rs9264608 | 0.986087 | 2.23E-308 | Peripheral blood mononuclear cells | HLA-C | rs1049281 | 2.23E-308 | SameSNP | 26019233 |
| WHRadjBMI | rs1049281 | rs9264636 | 0.971918 | 2.23E-308 | Peripheral blood mononuclear cells | HLA-C | rs1049281 | 2.23E-308 | SameSNP | 26019233 |
| WHRadjBMI | rs1049281 | rs9264638 | 0.915302 | 2.92E-260 | Peripheral blood mononuclear cells | HLA-C | rs1049281 | 2.23E-308 | SameSNP | 26019233 |
| WHRadjBMI | rs1049281 | rs9264638 | 0.915302 | 5.12E-12 | Peripheral blood mononuclear cells | HLA-C | rs1049281 | 2.23E-308 | SameSNP | 26019233 |
| WHRadjBMI | rs1049281 | rs9264647 | 0.935438 | 2.23E-308 | Peripheral blood mononuclear cells | HLA-C | rs1049281 | 2.23E-308 | SameSNP | 26019233 |
| WHRadjBMI | rs1856293 | rs1856293 | 1 | 1.65E-05 | Subc adipose (MuTHER) | TAAR6 | rs799227 | 1.32E-05 | 0.967 | 22941192 |
| WHRadjBMI | rs589428 | rs605203 | 0.994631 | 6.33E-20 | CD14+ monocytes (IFNg stimulated) | SKIV2L | rs486416 | 9.84E-21 | 1 | 24604202 |
| WHRadjBMI | rs589428 | rs589428 | IndexSNP | 6.33E-20 | CD14+ monocytes (IFNg stimulated) | SKIV2L | rs486416 | 9.84E-21 | 1 | 24604202 |

**Supplementary Table 11**. Association of cis-eQTLs for novel loci in whole blood adjusting for smoking status.

| Trait | Transcript | Tag SNP | CHR | POS | Gene | $\beta_{main}$ | $SE_{main}$ | Adj.Pvalue |
|---|---|---|---|---|---|---|---|---|
| BMI | 2339139 | rs2481665 | 1 | 62594677 | *INADL* | -0.02494 | 0.004266 | 8.980E-08 |
| BMI | 2339334 | rs2481665 | 1 | 62594677 | *L1TD1* | -0.01634 | 0.005455 | 1.808E-02 |
| WC | 3061319 | rs10269774 | 7 | 92253972 | *CDK6* | -0.01079 | 0.003726 | 2.467E-02 |
| WC | 3060994 | rs10269774 | 7 | 92253972 | *CYP51A1* | -0.0335 | 0.008983 | 1.758E-03 |
| WC | 3012633 | rs10269774 | 7 | 92253972 | *GATAD1* | -0.01556 | 0.003202 | 1.460E-05 |
| WC | 2319661 | rs17396340 | 1 | 10286176 | *SLC12A4* | 0.175292 | 0.006585 | 3.520E-154 |
| WC | 2675239 | rs4378999 | 3 | 51208646 | *C6orf10* | -0.01162 | 0.004206 | 3.572E-02 |
| WC | 2622970 | rs4378999 | 3 | 51208646 | *C6orf174* | 0.011549 | 0.003284 | 3.637E-03 |
| WC | 2622859 | rs4378999 | 3 | 51208646 | *C6orf58* | -0.0377 | 0.004833 | 1.530E-13 |
| WC | 2622912 | rs4378999 | 3 | 51208646 | *ECHDC1* | -0.06322 | 0.006041 | 4.000E-24 |
| WC | 2675504 | rs4378999 | 3 | 51208646 | *HLA-DOB* | 0.018156 | 0.005838 | 1.334E-02 |
| WC | 2623139 | rs4378999 | 3 | 51208646 | *PTPRK* | -0.03202 | 0.005981 | 1.360E-06 |
| WC | 2536298 | rs6743226 | 2 | 242236972 | *ACD* | -0.05566 | 0.003215 | 1.630E-65 |
| WC | 2536531 | rs6743226 | 2 | 242236972 | *ACD* | 0.015183 | 0.00276 | 6.070E-07 |
| WC | 2607055 | rs6743226 | 2 | 242236972 | *CENPT* | 0.009859 | 0.002622 | 1.586E-03 |
| WC | 2607110 | rs6743226 | 2 | 242236972 | *CENPT* | 0.016588 | 0.005435 | 1.560E-02 |
| WHR | 2902935 | rs1049281 | 6 | 31236567 | *AIF1* | 0.013159 | 0.003775 | 3.959E-03 |
| WHR | 2949311 | rs1049281 | 6 | 31236567 | *C2* | 0.012404 | 0.003394 | 2.244E-03 |
| WHR | 2948587 | rs1049281 | 6 | 31236567 | *CSNK2B* | 0.050802 | 0.004927 | 1.840E-23 |
| WHR | 2949885 | rs1049281 | 6 | 31236567 | *CYP21A2* | 0.014103 | 0.003826 | 2.018E-03 |
| WHR | 2902178 | rs1049281 | 6 | 31236567 | *DPCR1* | -0.01876 | 0.003565 | 2.050E-06 |
| WHR | 2902427 | rs1049281 | 6 | 31236567 | *HCG27* | 0.034784 | 0.005156 | 2.980E-10 |
| WHR | 2901463 | rs1049281 | 6 | 31236567 | *HLA-L* | 0.013763 | 0.004762 | 2.487E-02 |
| WHR | 2949132 | rs1049281 | 6 | 31236567 | *HSPA1A* | 0.02243 | 0.003579 | 6.750E-09 |
| WHR | 2949230 | rs1049281 | 6 | 31236567 | *HSPA1B* | 0.011195 | 0.003703 | 1.693E-02 |
| WHR | 2902804 | rs1049281 | 6 | 31236567 | *LST1* | 0.009603 | 0.003193 | 1.763E-02 |
| WHR | 2948630 | rs1049281 | 6 | 31236567 | *LY6G5B* | 0.033662 | 0.004684 | 1.510E-11 |
| WHR | 2948648 | rs1049281 | 6 | 31236567 | *LY6G5B* | 0.044411 | 0.007633 | 1.020E-07 |
| WHR | 2902574 | rs1049281 | 6 | 31236567 | *MICB* | 0.028136 | 0.007337 | 1.210E-03 |
| WHR | 2948887 | rs1049281 | 6 | 31236567 | *MSH5* | 1.877682 | 0.015225 | <1.264E-305 |
| WHR | 2948926 | rs1049281 | 6 | 31236567 | *MSH5* | 0.124382 | 0.005847 | 9.930E-99 |
| WHR | 2902326 | rs1049281 | 6 | 31236567 | *MUC21* | -0.05582 | 0.005415 | 1.840E-23 |
| WHR | 2901552 | rs1049281 | 6 | 31236567 | *RPP21* | 0.017709 | 0.005752 | 1.465E-02 |
| WHR | 2949488 | rs1049281 | 6 | 31236567 | *SKIV2L* | 0.127829 | 0.005665 | 6.330E-111 |
| WHR | 2949588 | rs1049281 | 6 | 31236567 | *STK19* | 0.019773 | 0.003975 | 8.560E-06 |
| WHR | 2902416 | rs1049281 | 6 | 31236567 | *TCF19* | 0.0124 | 0.004302 | 2.518E-02 |
| WHR | 2902725 | rs1049281 | 6 | 31236567 | *TNF* | 0.022217 | 0.005359 | 3.638E-04 |
| WHR | 3115008 | rs2001945 | 8 | 126477978 | *TRIB1* | 0.012548 | 0.00379 | 7.088E-03 |
| WHR | 2902884 | rs589428 | 6 | 31848220 | *AGER* | -0.01319 | 0.002381 | 5.060E-07 |
| WHR | 2902958 | rs589428 | 6 | 31848220 | *AGER* | 0.010621 | 0.002251 | 2.770E-05 |
| WHR | 2902935 | rs589428 | 6 | 31848220 | *AGER* | 0.015093 | 0.003697 | 4.709E-04 |
| WHR | 2902804 | rs589428 | 6 | 31848220 | *AGPAT1* | 0.011354 | 0.00313 | 2.456E-03 |
| WHR | 2950125 | rs589428 | 6 | 31848220 | *CIB2* | 0.024457 | 0.006273 | 9.480E-04 |
| WHR | 4048241 | rs589428 | 6 | 31848220 | *CTSH* | 0.69375 | 0.02946 | 1.010E-120 |
| WHR | 2902427 | rs589428 | 6 | 31848220 | *DOM3Z* | 0.02022 | 0.005091 | 7.249E-04 |
| WHR | 2902326 | rs589428 | 6 | 31848220 | *EHMT2* | -0.05188 | 0.005353 | 8.920E-21 |
| WHR | 2902207 | rs589428 | 6 | 31848220 | *EHMT2* | 0.025565 | 0.004894 | 2.460E-06 |
| WHR | 2902178 | rs589428 | 6 | 31848220 | *EHMT2* | -0.01341 | 0.003505 | 1.227E-03 |
| WHR | 2902574 | rs589428 | 6 | 31848220 | *FKBPL* | 0.03168 | 0.007208 | 1.244E-04 |

| | | | | | | | | |
|---|---|---|---|---|---|---|---|---|
| WHR | 2902609 | rs589428 | 6 | 31848220 | *FKBPL* | 0.015829 | 0.005918 | 4.587E-02 |
| WHR | 2903258 | rs589428 | 6 | 31848220 | *GPSM3* | -0.0567 | 0.007397 | 4.250E-13 |
| WHR | 2903219 | rs589428 | 6 | 31848220 | *GPSM3* | 0.147451 | 0.028362 | 2.760E-06 |
| WHR | 2949971 | rs589428 | 6 | 31848220 | *LINGO1* | -0.07492 | 0.007936 | 9.780E-20 |
| WHR | 2948887 | rs589428 | 6 | 31848220 | *MGC34034* | 0.85956 | 0.027996 | 5.680E-205 |
| WHR | 2949859 | rs589428 | 6 | 31848220 | *MORF4L1* | -0.025 | 0.007999 | 1.298E-02 |
| WHR | 2949488 | rs589428 | 6 | 31848220 | *NDUFAF4* | 0.267428 | 0.004336 | <1.264E-305 |
| WHR | 2903189 | rs589428 | 6 | 31848220 | *PBX2* | -0.03287 | 0.004706 | 6.020E-11 |
| WHR | 2903075 | rs589428 | 6 | 31848220 | *PBX2* | 0.011404 | 0.002362 | 1.680E-05 |
| WHR | 2902725 | rs589428 | 6 | 31848220 | *PRRT1* | 0.016694 | 0.005276 | 1.170E-02 |
| WHR | 4048265 | rs589428 | 6 | 31848220 | *RASGRF1* | 0.488385 | 0.024768 | 6.860E-85 |
| WHR | 2949885 | rs589428 | 6 | 31848220 | *RASGRF1* | 0.011886 | 0.003761 | 1.170E-02 |
| WHR | 2949132 | rs589428 | 6 | 31848220 | *STX7* | 0.018885 | 0.003533 | 1.370E-06 |
| WHR | 2949148 | rs589428 | 6 | 31848220 | *TAAR5* | -0.02488 | 0.005096 | 1.330E-05 |
| WHR | 2948926 | rs589428 | 6 | 31848220 | *TCF21* | 0.089165 | 0.005928 | 1.430E-49 |
| WHR | 2902463 | rs589428 | 6 | 31848220 | *TNXB* | 0.012875 | 0.003483 | 1.971E-03 |
| WHR | 2949230 | rs589428 | 6 | 31848220 | *VNN1* | 0.01419 | 0.003638 | 9.480E-04 |
| WHR | 2902348 | rs589428 | 6 | 31848220 | *ZBTB12* | -0.02464 | 0.003681 | 4.130E-10 |
| WHR | 2949311 | rs589428 | 6 | 31848220 | *DDAH2* | 0.015901 | 0.003326 | 2.090E-05 |

**Supplementary Table 12**. Association of cis-eQTLs for novel loci in whole blood for joint main + smoking ineraction.

| Trait | Transcript | Tag SNP | CHR | POS | Gene | $\beta_{main}$ | $SE_{main}$ | $\beta_{int}$ | $SE_{main}$ | $Cov_{main}$ | Adj. Pvalue |
|---|---|---|---|---|---|---|---|---|---|---|---|
| BMI | 3603436 | rs12902602 | 15 | 78967401 | *CHRNA5* | -0.03626 | 0.004127 | 0.007321 | 0.0133 | -1.67E-05 | 4.240E-17 |
| BMI | 3634811 | rs12902602 | 15 | 78967401 | *CTSH* | 0.03209 | 0.005488 | -0.00724 | 0.01741 | -2.87E-05 | 2.490E-07 |
| BMI | 3603408 | rs12902602 | 15 | 78967401 | *PSMA4* | -0.03678 | 0.005925 | -0.01281 | 0.01902 | -3.42E-05 | 2.610E-09 |
| WC | 2319661 | rs17396340 | 1 | 10286176 | *KIF1B* | -0.1778 | 0.006891 | 0.02395 | 0.02162 | -4.55E-05 | 4.810E-154 |
| WC | 2536531 | rs6743226 | 2 | 242236972 | *FARP2* | -0.01581 | 0.002886 | 0.005153 | 0.009423 | -8.01E-06 | 2.770E-06 |
| WC | 2607110 | rs6743226 | 2 | 242236972 | *HDLBP* | 0.01823 | 0.005656 | -0.00809 | 0.01854 | -3.10E-05 | 4.261E-02 |
| WC | 2607055 | rs6743226 | 2 | 242236972 | *PASK* | -0.01045 | 0.002739 | 0.005422 | 0.008806 | -6.98E-06 | 6.528E-03 |
| WC | 2536298 | rs6743226 | 2 | 242236972 | *SEPT2* | 0.05532 | 0.003322 | -0.00267 | 0.0105 | -9.89E-06 | 8.700E-64 |
| WHR | 2949311 | rs1049281 | 6 | 31236567 | *DDAH2* | 0.01253 | 0.003575 | -0.00232 | 0.01127 | -1.26E-05 | 1.362E-02 |
| WHR | 2949588 | rs1049281 | 6 | 31236567 | *DOM3Z* | 0.01789 | 0.004185 | 0.02139 | 0.01327 | -1.75E-05 | 1.280E-05 |
| WHR | 2948587 | rs1049281 | 6 | 31236567 | *FLOT1* | -0.04864 | 0.005155 | -0.01361 | 0.01588 | -2.51E-05 | 6.390E-22 |
| WHR | 2949885 | rs1049281 | 6 | 31236567 | *GPSM3* | 0.01243 | 0.004026 | 0.01338 | 0.01269 | -1.60E-05 | 8.922E-03 |
| WHR | 2902326 | rs1049281 | 6 | 31236567 | *HCP5* | -0.05335 | 0.00569 | -0.0261 | 0.01758 | -3.08E-05 | 7.540E-23 |
| WHR | 2948926 | rs1049281 | 6 | 31236567 | *HLA-B* | -0.1248 | 0.006133 | 0.005888 | 0.01854 | -3.43E-05 | 1.070E-96 |
| WHR | 2902725 | rs1049281 | 6 | 31236567 | *HSPA1B* | 0.02119 | 0.005638 | 0.008389 | 0.01761 | -3.09E-05 | 2.192E-03 |
| WHR | 2948630 | rs1049281 | 6 | 31236567 | *IER3* | 0.02917 | 0.004926 | 0.04421 | 0.01541 | -2.36E-05 | 2.690E-12 |
| WHR | 2902427 | rs1049281 | 6 | 31236567 | *LST1* | -0.03445 | 0.005426 | -0.00309 | 0.01694 | -2.86E-05 | 2.830E-09 |
| WHR | 2902574 | rs1049281 | 6 | 31236567 | *LY6G5B* | 0.03189 | 0.007651 | -0.02135 | 0.024 | -5.73E-05 | 1.819E-03 |
| WHR | 2949132 | rs1049281 | 6 | 31236567 | *NCR3* | -0.02313 | 0.003767 | 0.00752 | 0.01176 | -1.38E-05 | 5.170E-08 |
| WHR | 2948648 | rs1049281 | 6 | 31236567 | *NCRNA00243* | 0.04079 | 0.008008 | 0.04349 | 0.02457 | -6.02E-05 | 9.640E-08 |
| WHR | 2901552 | rs1049281 | 6 | 31236567 | *RPP21* | 0.01873 | 0.006047 | -0.00356 | 0.01917 | -3.65E-05 | 4.950E-02 |
| WHR | 2949488 | rs1049281 | 6 | 31236567 | *SLC44A4* | -0.1251 | 0.00593 | -0.02214 | 0.01779 | -3.16E-05 | 4.980E-109 |
| WHR | 2902935 | rs1049281 | 6 | 31236567 | *STK19* | 0.01406 | 0.003975 | -0.00735 | 0.01258 | -1.57E-05 | 1.541E-02 |
| WHR | 2902178 | rs1049281 | 6 | 31236567 | *TCF19* | 0.02045 | 0.003745 | -0.02038 | 0.01172 | -1.37E-05 | 5.420E-06 |
| WHR | 2948887 | rs1049281 | 6 | 31236567 | NA* | 1.88 | 0.01597 | -0.03048 | 0.04849 | -0.00023 | <1.754E-321 |
| WHR | 2974671 | rs1856293 | 6 | 133480940 | *C6orf192* | -0.02827 | 0.006785 | -0.01251 | 0.021 | -4.27E-05 | 3.215E-04 |

*The transcript id is "2948887 " according to the affymetrix exon array annotation. Gene symbol is null, entrze gene id is null.

**Supplementary Table 13**. GWAS Catalog lookups for novel loci.

| Current Study SNP | | | | | Previously Identified SNP | | | | | | | | | | | | | |
|---|---|---|---|---|---|---|---|---|---|---|---|---|---|---|---|---|---|---|
| TRAIT | CHR | rsID | POS (hg19) | Distance | rsID | POS | Risk Allele (RA) | RA freq | $r^2$ | P | OR/Beta | Disease/Trait | PUBMEDID | Discovery Sample Size | Replication Sample Size | Reported Gene | Mapped Gene | Annotation |
| BMI | 15 | rs12902602 | 78967401 | 73062 | rs1051730 | 78894339 | G | 0.65 | 0.52227 | 3.00E-73 | -1.02 | Smoking behavior | 20418890 | 74,035 | 68,988 | CHRNA3 | CHRNA3 | STOP-GAIN |
| BMI | 15 | rs12902602 | 78967401 | 73062 | rs1051730 | 78894339 | G | 0.66 | 0.52227 | 2.00E-66 | -0.08 | Smoking behavior | 20418889 | 41,150 | 120,516 | CHRNA5, CHRNA3 | CHRNA3 | STOP-GAIN |
| BMI | 15 | rs12902602 | 78967401 | 73062 | rs1051730 | 78894339 | T | 0.35 | 0.52227 | 2.00E-51 | 1.31 | Lung adenocarcinoma | 19836008 | 11,587 | 21,379 | CHRNA5, CHRNA3 | CHRNA3 | STOP-GAIN |
| BMI | 15 | rs12902602 | 78967401 | 73062 | rs1051730 | 78894339 | T | 0.35 | 0.52227 | 6.00E-20 | 0.1 | Nicotine dependence | 18385739 | 10,995 | 4,848 | CHRNA5, CHRNA3, CHRNB4 | CHRNA3 | STOP-GAIN |
| BMI | 15 | rs12902602 | 78967401 | 73062 | rs1051730 | 78894339 | A | 0.34 | 0.52227 | 2.00E-69 | 0.8 | Smoking behavior | 20418888 | 31,266 | 54,731 | NR | CHRNA3 | STOP-GAIN |
| BMI | 15 | rs12902602 | 78967401 | 73062 | rs1051730 | 78894339 | A | NR | 0.52227 | 1.00E-15 | 1.35 | Lung cancer | 18978790 | 6,717 | 8,472 | intergenic | CHRNA3 | STOP-GAIN |
| BMI | 15 | rs12902602 | 78967401 | 112833 | rs1994016 | 79080234 | C | 0.6 | 0.559287 | 5.00E-13 | 1.19 | Coronary artery disease | 21239051 | 2,723 | 17,053 | ADAMTS7 | ADAMTS7 | intron |
| BMI | 15 | rs12902602 | 78967401 | 121710 | rs3825807 | 79089111 | A | 0.57 | 0.52833 | 1.00E-12 | 1.08 | Coronary heart disease | 21378990 | 86,995 | 56,682 | ADAMTS7 | ADAMTS7 | missense |
| BMI | 15 | rs12902602 | 78967401 | 121710 | rs3825807 | 79089111 | A | 0.57 | 0.52833 | 7.00E-06 | 0.363 | Coronary artery calcification | 23561647 | 2,620 | NA | ADAMTS7 | ADAMTS7 | missense |
| WCadjBMI | 1 | rs17396340 | 10286176 | 0 | rs17396340 | 10286176 | A | NR | 1 | 3.00E-08 | 0.008 | Mean platelet volume | 22139419 | 18,600 | 18,838 | KIF1B | KIF1B, MIR1273D | intron;nearGene-5 |
| WCadjBMI | 3 | rs4378999 | 51208646 | 170628 | rs1031925 | 51379274 | NR | | 0.756449 | 8.00E-06 | 1.15 | Melanoma | 24980573 | 6,122 | 18,416 | DOCK3 | DOCK3 | intron |
| WCadjBMI | 4 | rs7697556 | 73515313 | 39299 | rs9993613 | 73476014 | T | 0.473 | 0.986617 | 5.00E-24 | 0.03 | Height | 25282103 | 253,288 | 80,067 | ADAMTS3 | ADAMTS3 - HNRNPA1P67 | intergenic |
| WCadjBMI | 4 | rs7697556 | 73515313 | 0 | rs7697556 | 73515313 | T | 0.47 | 1 | 2.00E-14 | 0.028 | Height | 20881960 | 133,653 | 50,074 | ADAMTS3 | ADAMTS3 - HNRNPA1P67 | intergenic |
| WCadjBMI | 7 | rs10269774 | 92253972 | 5896 | rs42235 | 92248076 | T | 0.31 | 0.679196 | 8.00E-47 | 0.057 | Height | 20881960 | 133,653 | 50,074 | CDK6 | CDK6 | intron |
| WCadjBMI | 7 | rs10269774 | 92253972 | 5896 | rs42235 | 92248076 | T | 0.3 | 0.679196 | 1.00E-28 | 1.28 | Height | 23563607 | 16,196 | 9,703 | CDK6 | CDK6 | intron |
| WCadjBMI | 7 | rs10269774 | 92253972 | 10438 | rs2282978 | 92264410 | NR | 0.08 | 0.920627 | 1.00E-08 | -0.06 | Height | 19343178 | 12,611 | 7,187 | CDK6 | CDK6 | intron |
| WCadjBMI | 7 | rs10269774 | 92253972 | 10438 | rs2282978 | 92264410 | C | 0.29 | 0.920627 | 1.00E-08 | 5.8 | Height | 18391951 | 30,968 | 8,541 | CDK6, ERVWE1, GATAD1, PEX1 | CDK6 | intron |
| WCadjBMI | 7 | rs10269774 | 92253972 | 10438 | rs2282978 | 92264410 | C | 0.33 | 0.920627 | 8.00E-23 | 0.09 | Height | 18391952 | 13,665 | 16,482 | CDK6 | CDK6 | intron |
| WCadjBMI | 8 | rs6470765 | 130736697 | 12969 | rs4733724 | 130723728 | A | 0.802 | 0.920945 | 1.00E-41 | 0.05 | Height | 25282103 | 253,288 | 80,067 | MLZE | MIR3686 - GSDMC | intergenic |
| WCadjBMI | 8 | rs6470765 | 130736697 | 11032 | rs6470764 | 130725665 | T | 0.2 | 0.911131 | 2.00E-28 | 0.05 | Height | 20881960 | 133,653 | 50,074 | GSDMC | MIR3686 - GSDMC | intergenic |
| WCadjBMI | 8 | rs6470765 | 130736697 | 11032 | rs6470764 | 130725665 | T | 0.203 | 0.911131 | 1.00E-06 | 0.054 | Infant length | 25281659 | 28,238 | NA | GSDMC | MIR3686 - GSDMC | intergenic |
| WCadjBMI | 8 | rs6470765 | 130736697 | 2236 | rs2062078 | 130734461 | T | 0.73 | 0.992777 | 1.00E-13 | 0.032 | Height | 25429064 | 36,227 | 57,699 | GSDMC | MIR3686 - GSDMC | intergenic |
| WHRadjBMI | 6 | rs1049281 | 31344546 | 4456 | rs3130542 | 31340090 | A | 0.13 | 0.720397 | 9.00E-14 | 1.33 | Hepatitis B | 24162738 | 1,888 | 9,903 | HLA-C | TRNAI25 | |
| WHRadjBMI | 6 | rs1049281 | 31344546 | 1751 | rs9264638 | 31346297 | A | 0.58 | 0.915302 | 2.00E-23 | -0.04 | Beta-2 microglubulin plasma levels | 23417110 | 6,728 | | HLA-C | HLA-C | intron |
| WHRadjBMI | 6 | rs1049281 | 31344546 | 15829 | rs2524054 | 31360375 | A | 0.32 | 0.79929 | 2.00E-28 | 0.37 | CD4:CD8 lymphocyte ratio | 20045101 | 3,627 | 988 | HLA-B | TRNAI25 | |
| WHRadjBMI | 6 | rs1049281 | 31344546 | 15829 | rs2524054 | 31360375 | A | 0.32 | 0.79929 | 6.00E-21 | -0.31 | CD4:CD8 lymphocyte ratio | 20045101 | 3,627 | 988 | HLA-B | TRNAI25 | |
| WHRadjBMI | 6 | rs1049281 | 31344546 | 28923 | rs2247056 | 31373469 | T | 0.25 | 0.853347 | 2.00E-15 | -2.99 | Triglycerides | 20686565 | 96,598 | NA | HLA | TRNAI25 | |
| WHRadjBMI | 6 | rs1049281 | 31344546 | 28923 | rs2247056 | 31373469 | T | 0.27 | 0.853347 | 4.00E-12 | 1.18 | Height | 23563607 | 16,196 | 9,703 | HLA-B, HLA-C | TRNAI25 | |
| WHRadjBMI | 6 | rs1049281 | 31344546 | 35694 | rs6457374 | 31380240 | T | 0.73 | 0.857577 | 8.00E-35 | -0.041 | Height | 25282103 | 253,288 | 80,067 | HLA-C | WASF5P - HLA-B | intergenic |
| WHRadjBMI | 8 | rs2001945 | 126477978 | 0 | rs2001945 | 126477978 | C | 0.42 | 1 | 1.00E-20 | 0.0405 | Triglycerides | 21909109 | 12,545 | 30,395 | intergenic | TRIB1 - LINC00861 | intergenic |
| WHRadjBMI | 8 | rs2001945 | 126477978 | 4099 | rs2954021 | 126482077 | G | 0.5 | 0.816403 | 1.00E-07 | -0.02 | LDL cholesterol | 20864672 | 17,723 | 47,439 | TRIB1 | TRIB1 - LINC00861 | intergenic |
| WHRadjBMI | 8 | rs2001945 | 126477978 | 4099 | rs2954021 | 126482077 | A | 0.5 | 0.816403 | 5.00E-09 | 1.6 | Liver enzyme levels (alanine transaminase) | 22001757 | 61,089 | NA | TRIB1 | TRIB1 - LINC00861 | intergenic |
| WHRadjBMI | 8 | rs2001945 | 126477978 | 4099 | rs2954021 | 126482077 | A | 0.5 | 0.816403 | 2.00E-13 | 1.4 | Liver enzyme levels (alkaline phosphatase) | 22001757 | 61,089 | NA | TRIB1 | TRIB1 - LINC00861 | intergenic |
| WHRadjBMI | 8 | rs2001945 | 126477978 | 8431 | rs17321515 | 126486409 | A | 0.47 | 0.610514 | 8.00E-07 | 0.07 | Lipid traits | 24386095 | 3,451 | 8,830 | TRIB1 | TRIB1 - LINC00861 | intergenic |
| WHRadjBMI | 8 | rs2001945 | 126477978 | 8431 | rs17321515 | 126486409 | A | 0.56 | 0.610514 | 7.00E-13 | 6.42 | Triglycerides | 18193043 | 8,684 | 9,741 | TRIB1 | TRIB1 - LINC00861 | intergenic |
| WHRadjBMI | 8 | rs2001945 | 126477978 | 8431 | rs17321515 | 126486409 | G | 0.49 | 0.610514 | 4.00E-17 | 0.08 | Triglycerides | 18193044 | 2,758 | 18,544 | TRIB1 | TRIB1 - LINC00861 | intergenic |
| WHRadjBMI | 13 | rs17065323 | 44627788 | 0 | rs17065323 | 44627788 | NR | NR | 1 | 4.00E-06 | -4.29 | Uric acid levels | 18759275 | 868 | | NR | LINC00284 - SMIM2-AS1 | intergenic |

**Supplementary Table 14**. Lookups of previously identified smoking loci in current combined ancestries meta-analyses results for BMI, WCadjBMI and WHRadjBMI.

| SNPS | CHR | POS(hg19) | DISEASE/TRAIT | REPORTED GENE(S) | PUBMEDID | Effect Allele | Other Allele | EAF | N | SMOKERS | | | NON-SMOKERS | | | MAIN and INTERACTION EFFECTS | | | | |
|---|---|---|---|---|---|---|---|---|---|---|---|---|---|---|---|---|---|---|---|---|
| | | | | | | | | | | β | SE | P | β | SE | P | $β_{adj}$ | $SE_{adj}$ | $P_{adj}$ | $P_{int}$ | $P_{joint}$ |
| **BMI** | | | | | | | | | | | | | | | | | | | | |
| rs1451240 | 8 | 42546711 | Nicotine dependence | *CHRNB3* | 22524403 | A | G | 0.263 | 149,164 | -0.011 | 0.010 | 2.84E-01 | 0.000 | 0.005 | 9.63E-01 | -0.003 | 0.005 | 6.04E-01 | 3.04E-01 | 5.63E-01 |
| rs6474412 | 8 | 42550498 | Smoking behavior | *CHRNB3, CHRNA6* | 20418888 | T | C | 0.740 | 173,262 | 0.007 | 0.010 | 4.48E-01 | -0.003 | 0.005 | 5.24E-01 | -0.001 | 0.005 | 8.90E-01 | 3.03E-01 | 6.10E-01 |
| rs3025343 | 9 | 136478355 | Smoking behavior (cessation) | *DBH* | 20418890 | A | G | 0.116 | 168,375 | -0.035 | 0.014 | 1.14E-02 | -0.013 | 0.008 | 9.69E-02 | -0.019 | 0.007 | **4.34E-03** | 1.28E-01 | 1.01E-02 |
| rs1329650 | 10 | 93348120 | Smoking behavior (CPD) | *LOC100188947* | 20418890 | T | G | 0.300 | 174,980 | 0.003 | 0.009 | 7.55E-01 | -0.008 | 0.005 | 9.13E-02 | -0.006 | 0.004 | 1.51E-01 | 2.63E-01 | 2.24E-01 |
| rs6265 | 11 | 27679916 | Smoking behavior (initiation) | *BDNF* | 20418890 | T | C | 0.193 | 217,329 | -0.061 | 0.009 | **2.49E-11** | -0.041 | 0.005 | **1.31E-16** | -0.043 | 0.004 | **8.13E-23** | 4.24E-02 | **3.50E-25** |
| rs2036527 | 15 | 78851615 | Smoking behavior (CPD) | *CHRNA5* | 22832964 | A | G | 0.331 | 240,437 | -0.039 | 0.007 | **1.11E-07** | 0.002 | 0.004 | 6.89E-01 | -0.007 | 0.003 | 2.98E-02 | **2.43E-07** | **7.96E-07** |
| rs1051730 | 15 | 78894339 | Smoking behavior (CPD, nicotine dependence) | *CHRNA3* | 20418890; 20418888; 20418889; 18385739 | A | G | 0.329 | 215,806 | -0.041 | 0.008 | **8.53E-08** | 0.002 | 0.004 | 6.64E-01 | -0.008 | 0.004 | 3.09E-02 | **1.61E-07** | **5.01E-07** |
| rs3733829 | 19 | 41310571 | Smoking behavior (CPD) | *EGLN2, CYP2A6* | 20418890 | A | G | 0.642 | 183,652 | -0.003 | 0.009 | 7.29E-01 | 0.000 | 0.005 | 9.59E-01 | 0.000 | 0.004 | 9.55E-01 | 7.27E-01 | 9.40E-01 |
| rs4105144 | 19 | 41358624 | Smoking behavior | *CYP2A6, RAB4D* | 20418888 | T | C | 0.312 | 135,367 | 0.003 | 0.011 | 7.66E-01 | -0.003 | 0.006 | 6.75E-01 | -0.002 | 0.005 | 7.77E-01 | 6.22E-01 | 8.76E-01 |
| rs8102683 | 19 | 41363765 | Smoking behavior | *CYP2A6* | 23049750 | T | C | 0.266 | 157,411 | 0.006 | 0.011 | 5.94E-01 | 0.000 | 0.006 | 9.81E-01 | 0.002 | 0.005 | 7.53E-01 | 6.12E-01 | 8.68E-01 |
| **WCadjBMI** | | | | | | | | | | | | | | | | | | | | |
| rs1451240 | 8 | 42546711 | Nicotine dependence | *CHRNB3* | 22524403 | A | G | 0.246 | 120,877 | -0.007 | 0.011 | 5.22E-01 | 0.008 | 0.006 | 1.80E-01 | 0.007 | 0.005 | 1.62E-01 | 2.03E-01 | 3.24E-01 |
| rs6474412 | 8 | 42550498 | Smoking behavior | *CHRNB3, CHRNA6* | 20418888 | T | C | 0.754 | 142,297 | 0.006 | 0.011 | 5.76E-01 | -0.005 | 0.005 | 3.73E-01 | -0.005 | 0.005 | 3.25E-01 | 3.45E-01 | 5.77E-01 |
| rs3025343 | 9 | 136478355 | Smoking behavior (cessation) | *DBH* | 20418890 | A | G | 0.116 | 138,287 | 0.000 | 0.015 | 9.97E-01 | 0.025 | 0.008 | **2.19E-03** | 0.015 | 0.007 | 3.34E-02 | 1.36E-01 | 9.19E-03 |
| rs1329650 | 10 | 93348120 | Smoking behavior (CPD) | *LOC100188947* | 20418890 | T | G | 0.310 | 143,896 | 0.007 | 0.010 | 4.66E-01 | 0.001 | 0.005 | 8.20E-01 | 0.001 | 0.005 | 8.96E-01 | 5.64E-01 | 7.47E-01 |
| rs6265 | 11 | 27679916 | Smoking behavior (initiation) | *BDNF* | 20418890 | T | C | 0.195 | 185,265 | 0.005 | 0.010 | 6.05E-01 | -0.003 | 0.005 | 6.10E-01 | -0.002 | 0.005 | 6.87E-01 | 4.62E-01 | 7.65E-01 |
| rs2036527 | 15 | 78851615 | Smoking behavior (CPD) | *CHRNA5* | 22832964 | A | G | 0.333 | 203,471 | 0.010 | 0.008 | 2.20E-01 | -0.003 | 0.004 | 4.66E-01 | -0.001 | 0.004 | 7.67E-01 | 1.32E-01 | 3.57E-01 |
| rs1051730 | 15 | 78894339 | Smoking behavior (CPD, nicotine dependence) | *CHRNA3* | 20418890; 20418888; 20418889; 18385739 | A | G | 0.331 | 183,565 | 0.007 | 0.008 | 4.06E-01 | -0.002 | 0.004 | 5.77E-01 | 0.000 | 0.004 | 9.70E-01 | 3.00E-01 | 6.13E-01 |
| rs3733829 | 19 | 41310571 | Smoking behavior (CPD) | *EGLN2, CYP2A6* | 20418890 | A | G | 0.638 | 154,278 | -0.010 | 0.009 | 2.79E-01 | -0.005 | 0.005 | 3.38E-01 | -0.006 | 0.004 | 1.83E-01 | 5.85E-01 | 3.50E-01 |
| rs4105144 | 19 | 41358624 | Smoking behavior | *CYP2A6, RAB4D* | 20418888 | T | C | 0.311 | 117,256 | 0.006 | 0.012 | 6.14E-01 | -0.007 | 0.007 | 2.68E-01 | -0.003 | 0.006 | 5.51E-01 | 3.06E-01 | 4.77E-01 |
| rs8102683 | 19 | 41363765 | Smoking behavior | *CYP2A6* | 23049750 | T | C | 0.268 | 130,065 | 0.008 | 0.012 | 5.03E-01 | -0.005 | 0.006 | 4.62E-01 | -0.002 | 0.006 | 7.77E-01 | 3.25E-01 | 6.07E-01 |
| **WHRadjBMI** | | | | | | | | | | | | | | | | | | | | |
| rs1451240 | 8 | 42546711 | Nicotine dependence | *CHRNB3* | 22524403 | A | G | 0.240 | 106,301 | -0.011 | 0.011 | 3.19E-01 | 0.007 | 0.006 | 2.93E-01 | 0.004 | 0.006 | 4.42E-01 | 1.52E-01 | 3.51E-01 |
| rs6474412 | 8 | 42550498 | Smoking behavior | *CHRNB3, CHRNA6* | 20418888 | T | C | 0.759 | 126,252 | 0.012 | 0.011 | 2.77E-01 | -0.002 | 0.006 | 7.75E-01 | -0.001 | 0.005 | 8.91E-01 | 2.63E-01 | 5.34E-01 |
| rs3025343 | 9 | 136478355 | Smoking behavior (cessation) | *DBH* | 20418890 | A | G | 0.117 | 121,686 | -0.008 | 0.015 | 5.90E-01 | 0.011 | 0.009 | 2.18E-01 | 0.004 | 0.007 | 6.29E-01 | 2.65E-01 | 4.07E-01 |
| rs1329650 | 10 | 93348120 | Smoking behavior (CPD) | *LOC100188947* | 20418890 | T | G | 0.312 | 129,224 | 0.004 | 0.010 | 6.55E-01 | -0.005 | 0.005 | 3.93E-01 | -0.003 | 0.005 | 5.83E-01 | 4.07E-01 | 6.29E-01 |
| rs6265 | 11 | 27679916 | Smoking behavior (initiation) | *BDNF* | 20418890 | T | C | 0.196 | 166,380 | 0.000 | 0.005 | 9.52E-01 | -0.001 | 0.004 | 8.30E-01 | -0.001 | 0.005 | 7.61E-01 | 9.37E-01 | 9.78E-01 |
| rs2036527 | 15 | 78851615 | Smoking behavior (CPD) | *CHRNA5* | 22832964 | A | G | 0.333 | 188,062 | 0.001 | 0.004 | 7.16E-01 | -0.001 | 0.003 | 8.55E-01 | 0.000 | 0.004 | 9.36E-01 | 6.91E-01 | 9.27E-01 |
| rs1051730 | 15 | 78894339 | Smoking behavior (CPD, nicotine dependence) | *CHRNA3* | 20418890; 20418888; 20418889; 18385739 | A | G | 0.332 | 166,117 | 0.001 | 0.004 | 7.89E-01 | 0.000 | 0.003 | 9.40E-01 | 0.001 | 0.004 | 8.54E-01 | 8.52E-01 | 9.61E-01 |
| rs3733829 | 19 | 41310571 | Smoking behavior (CPD) | *EGLN2, CYP2A6* | 20418890 | A | G | 0.637 | 137,052 | -0.004 | 0.009 | 6.27E-01 | -0.005 | 0.005 | 3.33E-01 | -0.005 | 0.004 | 2.50E-01 | 9.61E-01 | 5.61E-01 |
| rs4105144 | 19 | 41358624 | Smoking behavior | *CYP2A6, RAB4D* | 20418888 | T | C | 0.314 | 102,594 | 0.010 | 0.012 | 3.86E-01 | -0.010 | 0.007 | 1.69E-01 | -0.004 | 0.006 | 4.80E-01 | 1.39E-01 | 2.66E-01 |
| rs8102683 | 19 | 41363765 | Smoking behavior | *CYP2A6* | 23049750 | T | C | 0.270 | 113,338 | 0.008 | 0.012 | 4.81E-01 | -0.014 | 0.007 | 3.92E-02 | -0.008 | 0.006 | 1.82E-01 | 9.25E-02 | 9.00E-02 |

**Supplementary Table 15**. Validation results. We report the results for the current study (GIANT), validation study (UK Biobank [UKBB]), and the combined GIANT + UKBB meta-analysys results for the smoking stratum, SNPadjSMK, SNPint, and SNPjoint models. The discovery approach is included in the App column (1-SNPadjSMK, 2-SNPjoint, 3-SNPint, 4-SNPscreen) and strata (C=Combined Men and Women, W=Women-only, M=Men-only).

| | | | | | | | | Smokers | | | | | Non-Smokers | | | | | Main and Interaction Effects | | | | | | |
|---|---|---|---|---|---|---|---|---|---|---|---|---|---|---|---|---|---|---|---|---|---|---|---|---|
| Stage | App | MARKERNAME | Chr | pos_hg19 | Gene | Effect Allele | Other Allele | N | EAF | BETA | SE | P | N | EAF | BETA | SE | P | N | EAF | $BETA_{adjSmk}$ | $SE_{adjSmk}$ | $P_{adjSmk}$ | $P_{int}$ | $P_{joint}$ |
| **BMI** | | | | | | | | | | | | | | | | | | | | | | | | |
| GIANT | | | | | | | | 45,695 | 0.560 | 0.015 | 0.007 | 4.6E-02 | 163,758 | 0.560 | 0.021 | 0.004 | 8.9E-08 | 209,453 | 0.560 | 0.019 | 0.003 | **3.5E-08** | 4.0E-01 | 6.7E-08 |
| UKBB | 1:EC | rs2481665 | 1 | 62594677 | *INADL* | T | C | 14,688 | 0.562 | 0.012 | 0.013 | 3.6E-01 | 104,956 | 0.562 | 0.010 | 0.004 | 1.6E-02 | 119,644 | 0.562 | 0.011 | 0.004 | 1.0E-02 | 9.2E-01 | 3.6E-02 |
| GIANT + UKBB | | | | | | | | 60,383 | 0.561 | 0.014 | 0.006 | 2.0E-02 | 268,714 | 0.561 | 0.016 | 0.003 | **4.1E-08** | 329,097 | 0.561 | 0.016 | 0.002 | **3.3E-11** | 7.8E-01 | **2.0E-08** |
| GIANT | | | | | | | | 48,147 | 0.550 | 0.019 | 0.007 | 7.8E-03 | 176,920 | 0.546 | 0.020 | 0.004 | 8.4E-08 | 225,067 | 0.550 | 0.020 | 0.003 | **1.1E-09** | 8.2E-01 | **1.6E-08** |
| UKBB | 1,2 | rs10929925 | 2 | 6155557 | *SOX11* | C | A | 14,688 | 0.575 | 0.002 | 0.013 | 8.5E-01 | 104,956 | 0.575 | 0.020 | 0.004 | 5.8E-06 | 119,644 | 0.575 | 0.018 | 0.004 | 2.2E-05 | 2.0E-01 | 3.4E-05 |
| GIANT + UKBB | | | | | | | | 62,835 | 0.555 | 0.015 | 0.006 | 1.5E-02 | 281,876 | 0.557 | 0.020 | 0.003 | **2.1E-12** | 344,711 | 0.559 | 0.019 | 0.003 | **1.5E-13** | 4.5E-01 | **9.8E-13** |
| GIANT | | | | | | | | 39,000 | 0.850 | 0.025 | 0.011 | 2.3E-02 | 147,968 | 0.850 | 0.027 | 0.006 | 3.9E-06 | 186,968 | 0.850 | 0.028 | 0.005 | **4.3E-08** | 8.5E-01 | 1.8E-06 |
| UKBB | 1 | rs6794880 | 3 | 84451512 | *SRRM1P2* | A | G | 14,688 | 0.843 | 0.010 | 0.018 | 5.5E-01 | 104,956 | 0.843 | 0.018 | 0.006 | 2.0E-03 | 119,644 | 0.843 | 0.015 | 0.006 | 8.4E-03 | 1.2E-01 | 7.0E-03 |
| GIANT + UKBB | | | | | | | | 53,688 | 0.848 | 0.015 | 0.009 | 1.1E-01 | 252,924 | 0.847 | 0.023 | 0.004 | 5.0E-08 | 306,612 | 0.847 | 0.022 | 0.004 | **4.9E-09** | 4.5E-01 | 9.7E-08 |
| GIANT | | | | | | | | 23,249 | 0.260 | 0.024 | 0.011 | 3.1E-02 | 94,693 | 0.260 | 0.032 | 0.006 | 8.9E-08 | 117,942 | 0.260 | 0.031 | 0.005 | **7.3E-09** | 5.7E-01 | 6.5E-08 |
| UKBB | 1:EW | rs2173039 | 3 | 89142175 | *EPHA3* | C | G | 7,163 | 0.253 | 0.014 | 0.021 | 4.9E-01 | 55,866 | 0.253 | 0.011 | 0.007 | 1.1E-01 | 63,029 | 0.253 | 0.011 | 0.006 | 8.3E-02 | 8.7E-01 | 2.2E-01 |
| GIANT + UKBB | | | | | | | | 30,412 | 0.258 | 0.022 | 0.010 | 2.4E-02 | 150,559 | 0.257 | 0.023 | 0.005 | 4.3E-07 | 180,971 | 0.258 | 0.024 | 0.004 | **2.4E-09** | 9.3E-01 | 2.2E-07 |
| GIANT | | | | | | | | 26,237 | 0.257 | 0.025 | 0.011 | 2.1E-02 | 111,724 | 0.255 | 0.028 | 0.006 | 3.6E-07 | 137,961 | 0.260 | 0.027 | 0.005 | **4.8E-08** | 8.0E-01 | 2.0E-07 |
| UKBB | 1:AW | rs12629427 | 3 | 89145340 | *EPHA3* | C | T | 7,163 | 0.244 | 0.014 | 0.021 | 5.1E-01 | 55,866 | 0.254 | 0.011 | 0.007 | 1.0E-01 | 63,029 | 0.254 | 0.011 | 0.006 | 7.9E-02 | 9.0E-01 | 2.1E-01 |
| GIANT + UKBB | | | | | | | | 33,400 | 0.254 | 0.022 | 0.010 | 1.9E-02 | 167,590 | 0.254 | 0.021 | 0.004 | 7.2E-07 | 200,990 | 0.258 | 0.021 | 0.004 | 7.7E-08 | 9.1E-01 | 3.0E-07 |
| GIANT | | | | | | | | 47,957 | 0.080 | 0.061 | 0.014 | 1.8E-05 | 185,819 | 0.075 | 0.031 | 0.008 | 6.6E-05 | 233,776 | 0.080 | 0.035 | 0.007 | 1.2E-07 | 4.6E-02 | **3.5E-08** |
| UKBB | 2 | rs13069244 | 3 | 180441172 | *CCDC39* | A | G | 14,688 | 0.073 | 0.060 | 0.024 | 1.4E-02 | 104,956 | 0.073 | 0.021 | 0.008 | 1.1E-02 | 119,644 | 0.073 | 0.026 | 0.008 | 1.0E-03 | 1.3E-01 | 1.9E-03 |
| GIANT + UKBB | | | | | | | | 62,645 | 0.078 | 0.061 | 0.012 | 7.6E-07 | 290,775 | 0.074 | 0.026 | 0.006 | 3.2E-06 | 353,420 | 0.078 | 0.031 | 0.005 | **6.1E-10** | 1.1E-02 | **9.6E-11** |
| GIANT | | | | | | | | 35,428 | 0.184 | 0.063 | 0.012 | **4.8E-08** | 134,218 | 0.183 | -0.006 | 0.006 | 3.4E-01 | 169,646 | 0.180 | 0.007 | 0.006 | 2.3E-01 | **2.1E-08** | 1.9E-07 |
| UKBB | 3 | rs336396 | 4 | 143062811 | *INPP4B* | T | C | 14,688 | 0.185 | 0.003 | 0.016 | 8.7E-01 | 104,956 | 0.185 | -0.005 | 0.006 | 4.1E-01 | 119,644 | 0.185 | -0.004 | 0.005 | 4.8E-01 | 6.7E-01 | 7.0E-01 |
| GIANT + UKBB | | | | | | | | 50,116 | 0.184 | 0.043 | 0.009 | 4.8E-06 | 239,174 | 0.184 | -0.005 | 0.004 | 2.1E-01 | 289,290 | 0.182 | 0.001 | 0.004 | 7.4E-01 | 2.7E-06 | 1.3E-05 |
| GIANT | | | | | | | | 50,624 | 0.613 | 0.047 | 0.007 | **1.8E-11** | 189,511 | 0.619 | -0.002 | 0.004 | 5.5E-01 | 240,135 | 0.620 | 0.009 | 0.003 | 8.6E-03 | **4.1E-11** | **1.1E-10** |
| UKBB | 2,3 | rs12902602 | 15 | 78967401 | *CHRNB4* | A | G | 14,688 | 0.588 | 0.034 | 0.013 | 8.0E-03 | 104,956 | 0.588 | -0.008 | 0.004 | 7.6E-02 | 119,644 | 0.588 | -0.003 | 0.004 | 5.4E-01 | 2.1E-03 | 6.1E-03 |
| GIANT + UKBB | | | | | | | | 65,312 | 0.607 | 0.044 | 0.006 | **6.5E-13** | 294,467 | 0.608 | -0.005 | 0.003 | 1.1E-01 | 359,779 | 0.609 | 0.004 | 0.003 | 1.1E-01 | **6.0E-13** | **1.6E-12** |
| GIANT | | | | | | | | 13,819 | 0.589 | 0.074 | 0.014 | 9.8E-08 | 43,262 | 0.595 | 0.023 | 0.008 | 2.0E-03 | 57,081 | 0.590 | 0.036 | 0.007 | **4.9E-08** | 9.4E-04 | **5.6E-09** |
| UKBB | 4:AM | rs1809420 | 15 | 79056769 | *ADAMTS7* | T | C | 7,525 | 0.570 | 0.032 | 0.018 | 7.8E-02 | 49,090 | 0.574 | -0.003 | 0.006 | 6.9E-01 | 56,615 | 0.574 | 0.002 | 0.006 | 7.2E-01 | 7.3E-02 | 2.0E-01 |
| GIANT + UKBB | | | | | | | | 21,344 | 0.582 | 0.059 | 0.011 | 1.2E-07 | 92,352 | 0.584 | 0.008 | 0.005 | 8.5E-02 | 113,696 | 0.582 | 0.017 | 0.004 | 9.8E-05 | 3.3E-05 | 1.8E-07 |
| **WCadjBMI** | | | | | | | | | | | | | | | | | | | | | | | | |
| GIANT | | | | | | | | 42,263 | 0.140 | 0.016 | 0.011 | 1.4E-01 | 164,222 | 0.135 | 0.035 | 0.006 | **4.7E-10** | 206,485 | 0.135 | 0.028 | 0.005 | **3.0E-08** | 9.8E-02 | **9.1E-10** |
| UKBB | 1,2 | rs17396340 | 1 | 10286176 | *KIF1B* | A | G | 14,687 | 0.127 | -0.006 | 0.018 | 7.4E-01 | 104,926 | 0.127 | 0.028 | 0.007 | 1.4E-05 | 119,613 | 0.127 | 0.024 | 0.006 | 8.1E-05 | 6.8E-02 | 6.8E-05 |
| GIANT + UKBB | | | | | | | | 56,950 | 0.137 | 0.010 | 0.009 | 2.9E-01 | 269,148 | 0.132 | 0.032 | 0.004 | **2.7E-14** | 326,098 | 0.132 | 0.027 | 0.004 | **1.0E-11** | 2.9E-02 | **1.5E-13** |
| GIANT | | | | | | | | 41,843 | 0.533 | 0.018 | 0.007 | 1.3E-02 | 158,823 | 0.533 | 0.023 | 0.004 | **2.6E-09** | 200,666 | 0.533 | 0.022 | 0.004 | **1.2E-10** | 5.5E-01 | **5.8E-10** |
| UKBB | 1,2 | rs6743226 | 2 | 242236972 | *HDLBP* | C | T | 14,687 | 0.558 | 0.010 | 0.012 | 3.8E-01 | 104,926 | 0.558 | 0.013 | 0.004 | 3.2E-03 | 119,613 | 0.558 | 0.013 | 0.004 | 2.2E-03 | 8.4E-01 | 9.3E-03 |
| GIANT + UKBB | | | | | | | | 56,530 | 0.540 | 0.016 | 0.006 | 9.9E-03 | 263,749 | 0.543 | 0.019 | 0.003 | **9.3E-11** | 320,279 | 0.543 | 0.018 | 0.003 | **6.7E-12** | 7.0E-01 | **2.8E-11** |
| GIANT | | | | | | | | 31,449 | 0.130 | 0.035 | 0.014 | 1.3E-02 | 125,117 | 0.130 | 0.035 | 0.007 | 1.3E-06 | 156,566 | 0.128 | 0.036 | 0.007 | **4.1E-08** | 9.7E-01 | 4.1E-07 |
| UKBB | 1 | rs4378999 | 3 | 51208646 | *DOCK3* | T | A | 14,687 | 0.108 | 0.039 | 0.019 | 3.8E-02 | 104,926 | 0.108 | 0.022 | 0.007 | 1.7E-03 | 119,613 | 0.108 | 0.024 | 0.007 | 2.4E-04 | 4.0E-01 | 8.6E-04 |
| GIANT + UKBB | | | | | | | | 46,136 | 0.123 | 0.036 | 0.011 | 1.1E-03 | 230,043 | 0.120 | 0.029 | 0.005 | **8.6E-09** | 276,179 | 0.119 | 0.030 | 0.005 | **7.6E-11** | 5.3E-01 | **3.2E-10** |
| GIANT | | | | | | | | 42,135 | 0.489 | 0.004 | 0.007 | 6.3E-01 | 163,882 | 0.495 | 0.025 | 0.004 | **7.3E-11** | 206,017 | 0.494 | 0.021 | 0.004 | **5.2E-09** | 6.7E-03 | **7.6E-10** |
| UKBB | 1,2 | rs7697556 | 4 | 73515313 | *ADAMTS3* | T | C | 14,687 | 0.473 | 0.027 | 0.012 | 2.1E-02 | 104,926 | 0.473 | 0.028 | 0.004 | **8.4E-11** | 119,613 | 0.473 | 0.028 | 0.004 | **5.7E-12** | 9.4E-01 | **7.2E-11** |
| GIANT + UKBB | | | | | | | | 56,822 | 0.484 | 0.010 | 0.006 | 1.0E-01 | 268,808 | 0.487 | 0.027 | 0.003 | **8.9E-20** | 325,630 | 0.486 | 0.024 | 0.003 | **5.4E-19** | 1.9E-02 | **2.7E-19** |
| GIANT | | | | | | | | 19,078 | 0.732 | 0.044 | 0.012 | 3.1E-04 | 58,599 | 0.738 | 0.030 | 0.007 | 1.9E-05 | 77,677 | 0.732 | 0.034 | 0.006 | **1.8E-08** | 3.2E-01 | 1.7E-07 |
| UKBB | 1:EM | rs1545348 | 5 | 34718343 | *RAI14* | T | G | 7,524 | 0.758 | 0.023 | 0.019 | 2.3E-01 | 49,078 | 0.758 | 0.009 | 0.007 | 2.2E-01 | 56,602 | 0.758 | 0.011 | 0.007 | 1.2E-01 | 5.0E-01 | 2.3E-01 |
| GIANT + UKBB | | | | | | | | 26,602 | 0.739 | 0.038 | 0.010 | 2.1E-04 | 107,677 | 0.747 | 0.020 | 0.005 | 8.8E-05 | 134,279 | 0.743 | 0.024 | 0.005 | 1.2E-07 | 1.2E-01 | 4.8E-07 |

| | | | | | | | | | | | | | | | | | | | | | | | | |
|---|---|---|---|---|---|---|---|---|---|---|---|---|---|---|---|---|---|---|---|---|---|---|---|---|
| GIANT | | | | | | | | 32,215 | 0.346 | 0.024 | 0.009 | 6.6E-03 | 125,337 | 0.337 | 0.023 | 0.005 | 1.1E-06 | 157,552 | 0.339 | 0.023 | 0.004 | **2.9E-08** | 8.8E-01 | 1.6E-07 |
| UKBB | 1 | rs10269774 | 7 | 92253972 | *CDK6* | A | G | 14,687 | 0.325 | 0.003 | 0.012 | 7.9E-01 | 104,926 | 0.325 | 0.016 | 0.005 | 6.5E-04 | 119,613 | 0.325 | 0.014 | 0.004 | 1.0E-03 | 3.4E-01 | 3.2E-03 |
| GIANT + UKBB | | | | | | | | 46,902 | 0.339 | 0.017 | 0.007 | 1.8E-02 | 230,263 | 0.331 | 0.019 | 0.003 | **4.6E-09** | 277,165 | 0.333 | 0.019 | 0.003 | **2.9E-10** | 7.7E-01 | **2.1E-09** |
| GIANT | | | | | | | | 32,162 | 0.762 | 0.032 | 0.010 | 1.9E-03 | 125,288 | 0.760 | 0.023 | 0.005 | 1.7E-05 | 157,450 | 0.760 | 0.026 | 0.005 | **4.8E-08** | 4.3E-01 | 9.5E-07 |
| UKBB | 1 | rs6470765 | 8 | 130736697 | *GSDMC* | A | C | 14,687 | 0.798 | 0.011 | 0.015 | 4.7E-01 | 104,926 | 0.798 | 0.024 | 0.005 | 1.2E-05 | 119,613 | 0.798 | 0.022 | 0.005 | 1.3E-05 | 3.9E-01 | 4.7E-05 |
| GIANT + UKBB | | | | | | | | 46,849 | 0.773 | 0.025 | 0.008 | 3.2E-03 | 230,214 | 0.777 | 0.023 | 0.004 | **8.9E-10** | 277,063 | 0.777 | 0.024 | 0.003 | **2.5E-12** | 8.9E-01 | **9.0E-11** |
| GIANT | | | | | | | | 31,918 | 0.741 | 0.012 | 0.010 | 2.3E-01 | 124,509 | 0.748 | 0.030 | 0.005 | **4.2E-09** | 156,427 | 0.746 | 0.026 | 0.005 | **2.3E-08** | 8.5E-02 | **1.7E-08** |
| UKBB | 2 | rs9408815 | 9 | 108890521 | *TMEM38B* | C | G | 14,687 | 0.755 | 0.022 | 0.014 | 1.2E-01 | 104,926 | 0.755 | 0.019 | 0.005 | 2.9E-04 | 119,613 | 0.755 | 0.019 | 0.005 | 8.1E-05 | 8.4E-01 | 4.1E-04 |
| GIANT + UKBB | | | | | | | | 46,605 | 0.745 | 0.015 | 0.008 | 5.8E-02 | 229,435 | 0.751 | 0.024 | 0.004 | **1.9E-11** | 276,040 | 0.750 | 0.023 | 0.003 | **1.2E-11** | 3.0E-01 | **2.8E-11** |
| GIANT | | | | | | | | 32,248 | 0.754 | 0.017 | 0.010 | 8.1E-02 | 125,537 | 0.761 | 0.029 | 0.005 | **2.6E-08** | 157,785 | 0.760 | 0.027 | 0.005 | **1.5E-08** | 2.7E-01 | **4.6E-08** |
| UKBB | 1 | rs9409082 | 9 | 108901049 | *TMEM38B* | C | T | 14,687 | 0.758 | 0.024 | 0.014 | 8.2E-02 | 104,926 | 0.758 | 0.018 | 0.005 | 4.0E-04 | 119,613 | 0.758 | 0.019 | 0.005 | 8.9E-05 | 6.9E-01 | 4.1E-04 |
| GIANT + UKBB | | | | | | | | 46,935 | 0.755 | 0.020 | 0.008 | 1.5E-02 | 230,463 | 0.760 | 0.024 | 0.004 | **1.5E-10** | 277,398 | 0.759 | 0.023 | 0.003 | **9.5E-12** | 6.6E-01 | **6.5E-11** |
| GIANT | | | | | | | | 31,839 | 0.501 | 0.037 | 0.009 | 2.2E-05 | 122,053 | 0.496 | -0.015 | 0.005 | 9.6E-04 | 153,892 | 0.497 | -0.003 | 0.004 | 4.4E-01 | **2.7E-08** | 5.0E-07 |
| UKBB | 3 | rs4141488 | 16 | 9629067 | *GRIN2A* | T | C | 14,687 | 0.501 | 0.018 | 0.012 | 1.3E-01 | 104,926 | 0.501 | 0.001 | 0.004 | 7.6E-01 | 119,613 | 0.501 | 0.003 | 0.004 | 4.1E-01 | 1.9E-01 | 3.1E-01 |
| GIANT + UKBB | | | | | | | | 46,526 | 0.501 | 0.030 | 0.007 | 1.8E-05 | 226,979 | 0.498 | -0.007 | 0.003 | 3.7E-02 | 273,505 | 0.499 | 0.000 | 0.003 | 9.5E-01 | 1.8E-06 | 1.1E-05 |
| GIANT | | | | | | | | 43,171 | 0.410 | 0.026 | 0.007 | 5.4E-04 | 164,833 | 0.407 | 0.018 | 0.004 | 6.5E-06 | 208,004 | 0.407 | 0.020 | 0.004 | **1.9E-08** | 3.3E-01 | 1.3E-07 |
| UKBB | 1 | rs6012558 | 20 | 47531286 | *ARFGEF2* | A | G | 14,687 | 0.420 | 0.029 | 0.012 | 1.7E-02 | 104,926 | 0.420 | 0.009 | 0.004 | 4.4E-02 | 119,613 | 0.420 | 0.011 | 0.004 | 6.5E-03 | 1.3E-01 | 7.7E-03 |
| GIANT + UKBB | | | | | | | | 57,858 | 0.413 | 0.026 | 0.006 | 2.8E-05 | 269,759 | 0.412 | 0.014 | 0.003 | 3.3E-06 | 327,617 | 0.412 | 0.016 | 0.003 | **1.5E-09** | 7.0E-02 | **3.0E-09** |
| GIANT | | | | | | | | 11,858 | 0.969 | 0.169 | 0.039 | 1.4E-05 | 65,072 | 0.967 | -0.070 | 0.018 | 1.2E-04 | 76,930 | 0.969 | -0.034 | 0.016 | 3.5E-02 | **1.4E-08** | **4.8E-08** |
| UKBB | 2,3:EW | rs6076699 | 20 | 4566688 | *PRNP* | A | G | 7,163 | 0.978 | 0.012 | 0.056 | 8.3E-01 | 55,848 | 0.978 | -0.016 | 0.021 | 4.4E-01 | 63,011 | 0.978 | -0.013 | 0.020 | 5.2E-01 | 6.3E-01 | 7.2E-01 |
| GIANT + UKBB | | | | | | | | 19,021 | 0.973 | 0.117 | 0.032 | 2.3E-04 | 120,920 | 0.972 | -0.047 | 0.014 | 6.3E-04 | 139,941 | 0.973 | -0.025 | 0.012 | 4.2E-02 | 2.3E-06 | 3.4E-06 |
| **WHRadjBMI** | | | | | | | | | | | | | | | | | | | | | | | | |
| GIANT | | | | | | | | 40,484 | 0.647 | 0.003 | 0.004 | 3.9E-01 | 148,544 | 0.639 | 0.019 | 0.003 | **3.1E-11** | 186,625 | 0.621 | 0.029 | 0.004 | **3.1E-16** | 7.3E-04 | **2.1E-10** |
| UKBB | 4 | rs765751 | 1 | 219669226 | *LYPLA1* | C | T | 14,684 | 0.601 | 0.038 | 0.012 | 1.3E-03 | 104,916 | 0.601 | 0.034 | 0.004 | **3.0E-14** | 119,600 | 0.601 | 0.034 | 0.004 | **1.8E-16** | 7.2E-01 | **1.7E-15** |
| GIANT + UKBB | | | | | | | | 55,168 | 0.624 | 0.007 | 0.004 | 7.2E-02 | 253,460 | 0.620 | 0.024 | 0.002 | **3.2E-22** | 306,225 | 0.611 | 0.031 | 0.003 | **9.1E-31** | *1.4E-04* | **7.8E-22** |
| GIANT | | | | | | | | 20,609 | 0.319 | 0.012 | 0.006 | 5.5E-02 | 86,960 | 0.303 | 0.009 | 0.004 | 1.5E-02 | 106,640 | 0.305 | 0.027 | 0.005 | **4.9E-08** | 6.8E-01 | 7.8E-03 |
| UKBB | 1:AW | rs670752 | 3 | 107312980 | *BBX* | A | G | 7,161 | 0.286 | 0.058 | 0.018 | 1.7E-03 | 55,845 | 0.286 | 0.015 | 0.007 | 2.0E-02 | 63,006 | 0.286 | 0.020 | 0.006 | 1.3E-03 | 3.0E-02 | 4.8E-04 |
| GIANT + UKBB | | | | | | | | 27,770 | 0.303 | 0.016 | 0.006 | 4.9E-03 | 142,805 | 0.295 | 0.010 | 0.003 | 1.1E-03 | 169,646 | 0.296 | 0.024 | 0.004 | **3.1E-10** | 3.8E-01 | 9.5E-05 |
| GIANT | | | | | | | | 18,150 | 0.291 | 0.002 | 0.006 | 7.8E-01 | 53,274 | 0.266 | 0.031 | 0.006 | **3.7E-08** | 71,475 | 0.263 | 0.040 | 0.006 | **1.7E-10** | 1.6E-04 | 2.7E-07 |
| UKBB | 4:EM | rs30000 | 5 | 55803533 | *MAP3K1* | G | A | 7,523 | 0.256 | -0.008 | 0.019 | 6.7E-01 | 49,071 | 0.256 | 0.045 | 0.007 | **5.9E-10** | 56,594 | 0.256 | 0.038 | 0.007 | **2.2E-08** | 9.5E-03 | **4.2E-09** |
| GIANT + UKBB | | | | | | | | 25,673 | 0.273 | 0.001 | 0.005 | 8.9E-01 | 102,345 | 0.261 | 0.036 | 0.004 | **3.7E-16** | 128,069 | 0.260 | 0.039 | 0.005 | **2.7E-17** | *3.2E-07* | **3.8E-15** |
| GIANT | | | | | | | | 19,661 | 0.286 | 0.004 | 0.006 | 5.0E-01 | 61,191 | 0.271 | 0.034 | 0.005 | **4.1E-10** | 80,920 | 0.274 | 0.043 | 0.006 | **2.3E-13** | 6.8E-05 | **2.2E-09** |
| UKBB | 4:AM | rs459193 | 5 | 55806751 | *MAP3K1* | A | G | 7,523 | 0.253 | -0.008 | 0.019 | 6.6E-01 | 49,071 | 0.253 | 0.045 | 0.007 | **3.6E-10** | 56,594 | 0.253 | 0.038 | 0.007 | **1.5E-08** | 8.6E-03 | **2.6E-09** |
| GIANT + UKBB | | | | | | | | 27,184 | 0.270 | 0.003 | 0.005 | 6.0E-01 | 110,262 | 0.262 | 0.038 | 0.004 | **1.6E-18** | 137,514 | 0.264 | 0.041 | 0.004 | **3.5E-20** | *2.5E-07* | **1.6E-17** |
| GIANT | | | | | | | | 32,281 | 0.664 | 0.022 | 0.009 | 1.3E-02 | 117,004 | 0.664 | 0.027 | 0.005 | **2.0E-08** | 149,566 | 0.663 | 0.025 | 0.004 | **2.2E-09** | 5.6E-01 | **5.3E-09** |
| UKBB | 1,2 | rs1049281 | 6 | 31236567 | *HLA-C* | C | T | 14,684 | 0.641 | 0.033 | 0.012 | 6.6E-03 | 104,916 | 0.641 | 0.027 | 0.005 | **2.2E-09** | 119,600 | 0.641 | 0.028 | 0.004 | **5.3E-11** | 6.6E-01 | **4.3E-10** |
| GIANT + UKBB | | | | | | | | 46,965 | 0.652 | 0.025 | 0.007 | 3.0E-04 | 221,920 | 0.653 | 0.027 | 0.005 | **1.6E-08** | 269,166 | 0.652 | 0.026 | 0.003 | **1.2E-18** | 8.3E-01 | **1.8E-10** |
| GIANT | | | | | | | | 35,979 | 0.664 | 0.006 | 0.004 | 1.2E-01 | 126,939 | 0.670 | 0.011 | 0.003 | 4.1E-04 | 161,392 | 0.668 | 0.022 | 0.004 | **2.8E-08** | 3.5E-01 | 7.0E-04 |
| UKBB | 1:EC | rs589428 | 6 | 31848220 | *EHMT2* | G | T | 14,684 | 0.632 | 0.028 | 0.012 | 2.2E-02 | 104,916 | 0.632 | 0.028 | 0.004 | **8.4E-10** | 119,600 | 0.632 | 0.027 | 0.004 | **6.4E-11** | 9.9E-01 | **4.9E-10** |
| GIANT + UKBB | | | | | | | | 50,663 | 0.648 | 0.008 | 0.004 | 2.9E-02 | 231,855 | 0.651 | 0.016 | 0.003 | **2.1E-10** | 280,992 | 0.650 | 0.024 | 0.003 | **1.1E-17** | 8.4E-02 | **1.6E-10** |
| GIANT | | | | | | | | 39,761 | 0.479 | 0.007 | 0.004 | 7.9E-02 | 148,413 | 0.484 | 0.022 | 0.003 | **2.2E-15** | 186,080 | 0.477 | 0.037 | 0.003 | **3.7E-27** | 9.7E-04 | **3.8E-15** |
| UKBB | 4 | rs7766106 | 6 | 127455138 | *RSPO3* | T | C | 14,684 | 0.495 | 0.032 | 0.012 | 6.2E-03 | 104,916 | 0.495 | 0.044 | 0.004 | **1.5E-24** | 119,600 | 0.495 | 0.043 | 0.004 | **4.3E-26** | 3.3E-01 | **4.5E-25** |
| GIANT + UKBB | | | | | | | | 54,445 | 0.487 | 0.009 | 0.004 | 1.3E-02 | 253,329 | 0.489 | 0.028 | 0.002 | **5.0E-34** | 305,680 | 0.486 | 0.039 | 0.003 | **4.4E-51** | *1.0E-05* | **3.4E-34** |
| GIANT | | | | | | | | 27,688 | 0.524 | 0.006 | 0.009 | 5.3E-01 | 99,743 | 0.518 | -0.028 | 0.005 | **9.1E-09** | 127,508 | 0.519 | -0.019 | 0.004 | 6.5E-06 | 5.4E-04 | **4.7E-08** |
| UKBB | 2:EC | rs1856293 | 6 | 133480940 | *EYA4* | A | C | 14,684 | 0.514 | -0.011 | 0.012 | 3.5E-01 | 104,916 | 0.514 | -0.013 | 0.004 | 3.6E-03 | 119,600 | 0.514 | -0.012 | 0.004 | 2.3E-03 | 8.9E-01 | 9.3E-03 |
| GIANT + UKBB | | | | | | | | 42,372 | 0.519 | 0.000 | 0.007 | 9.6E-01 | 204,659 | 0.516 | -0.020 | 0.003 | **1.9E-09** | 247,108 | 0.517 | -0.016 | 0.003 | 9.6E-08 | 1.3E-02 | **1.5E-08** |
| GIANT | | | | | | | | 19,957 | 0.404 | 0.009 | 0.006 | 1.2E-01 | 83,489 | 0.442 | 0.013 | 0.003 | 1.0E-04 | 102,564 | 0.488 | 0.025 | 0.005 | **4.7E-08** | 5.9E-01 | 1.3E-04 |
| UKBB | 1:AW | rs2001945 | 8 | 126477978 | *TRIB1* | G | C | 7,161 | 0.479 | -0.003 | 0.016 | 8.3E-01 | 55,845 | 0.479 | 0.019 | 0.006 | 1.4E-03 | 63,006 | 0.479 | 0.017 | 0.006 | 3.2E-03 | 2.0E-01 | 5.9E-03 |
| GIANT + UKBB | | | | | | | | 27,118 | 0.441 | 0.008 | 0.006 | 1.6E-01 | 139,334 | 0.460 | 0.014 | 0.003 | 5.9E-07 | 165,570 | 0.484 | 0.022 | 0.004 | **1.1E-09** | 3.0E-01 | 1.4E-06 |
| GIANT | | | | | | | | 18,192 | 0.392 | 0.003 | 0.005 | 6.2E-01 | 52,944 | 0.342 | 0.026 | 0.005 | 8.2E-07 | 71,812 | 0.360 | 0.034 | 0.006 | **3.0E-09** | 1.1E-03 | 4.0E-06 |
| UKBB | 4:EM | rs754133 | 12 | 54418920 | *HOXC4-HOXC6* | A | G | 7,523 | 0.362 | 0.026 | 0.017 | 1.3E-01 | 49,071 | 0.362 | 0.024 | 0.007 | 2.7E-04 | 56,594 | 0.362 | 0.024 | 0.006 | 7.8E-05 | 9.0E-01 | 4.1E-04 |

| | | | | | | | | | | | | | | | | | | | | | | | | | | |
|---|---|---|---|---|---|---|---|---|---|---|---|---|---|---|---|---|---|---|---|---|---|---|---|---|---|---|
| GIANT + UKBB | | | | | [illegible] | | | 25,715 | 0.377 | 0.004 | 0.005 | 3.7E-01 | 102,015 | 0.352 | 0.025 | 0.004 | **7.6E-10** | 128,406 | 0.361 | 0.030 | 0.004 | **2.1E-12** | *9.7E-04* | **4.0E-09** |
| GIANT | | | | | | | | 18,144 | 0.402 | 0.003 | 0.005 | 6.0E-01 | 52,724 | 0.354 | 0.026 | 0.005 | 1.0E-06 | 71,571 | 0.364 | 0.034 | 0.006 | **9.1E-09** | 1.1E-03 | 5.7E-06 |
| UKBB | 4:AM | rs2071449 | 12 | 54428011 | *HOXC4-HOXC6* | A | C | 7,523 | 0.366 | 0.026 | 0.017 | 1.3E-01 | 49,071 | 0.366 | 0.025 | 0.007 | 1.3E-04 | 56,594 | 0.366 | 0.025 | 0.006 | 3.9E-05 | 9.7E-01 | 2.1E-04 |
| GIANT + UKBB | | | | | | | | 25,667 | 0.384 | 0.004 | 0.005 | 3.6E-01 | 101,795 | 0.360 | 0.026 | 0.004 | **5.4E-10** | 128,165 | 0.365 | 0.006 | 0.004 | **2.7E-12** | *8.0E-04* | **2.8E-09** |
| GIANT | | | | | | | | 6,602 | 0.007 | 0.154 | 0.116 | 1.9E-01 | 63,366 | 0.008 | -0.230 | 0.036 | **1.2E-10** | 86,739 | 0.007 | -0.181 | 0.032 | **9.2E-09** | 1.4E-03 | **3.9E-10** |
| UKBB | 1:EC | rs17065323 | 13 | 44627788 | *SMIM2* | T | C | 14,684 | 0.004 | 0.006 | 0.107 | 9.6E-01 | 104,916 | 0.004 | -0.091 | 0.040 | 2.3E-02 | 119,600 | 0.004 | -0.079 | 0.037 | 3.6E-02 | 4.0E-01 | 7.6E-02 |
| GIANT + UKBB | | | | | | | | 21,286 | 0.006 | 0.073 | 0.079 | 3.5E-01 | 168,282 | 0.006 | -0.168 | 0.027 | **2.5E-10** | 206,339 | 0.006 | -0.139 | 0.024 | **9.6E-09** | *3.6E-03* | **1.3E-09** |
| GIANT | | | | | | | | 19,459 | 0.389 | 0.006 | 0.005 | 2.6E-01 | 60,628 | 0.358 | 0.025 | 0.005 | 5.0E-07 | 80,912 | 0.357 | 0.032 | 0.005 | **4.7E-09** | 5.5E-03 | 1.8E-06 |
| UKBB | 4:AM | rs12608504 | 19 | 18389135 | *JUND* | A | G | 7,523 | 0.366 | 0.029 | 0.017 | 9.2E-02 | 49,071 | 0.366 | 0.019 | 0.007 | 3.1E-03 | 56,594 | 0.366 | 0.021 | 0.006 | 7.4E-04 | 6.0E-01 | 3.0E-03 |
| GIANT + UKBB | | | | | | | | 26,982 | 0.378 | 0.007 | 0.005 | 1.3E-01 | 109,699 | 0.362 | 0.023 | 0.004 | **6.9E-09** | 137,506 | 0.362 | 0.027 | 0.004 | **2.9E-11** | 1.3E-02 | **1.6E-08** |

**Supplementary Table 16.** Genetic Risk Score (GRS) calculation in the combined KORA-S3 and KORA-S4 study.

| SNPset | Model[a] | $Beta_{GRS}$ | $Pval_{GRS}$ | $Beta_{SMK}$ | $Pval_{SMK}$ | $Beta_{GRSxSMK}$ | $Pval_{GRSxSMK}$ | Multiple $R^2$ |
|---|---|---|---|---|---|---|---|---|
| 97 known | BMI~GRS | 0.08847 | 3.13E-13 | - | - | - | - | 0.01532 |
| | BMI~GRS+SMK | 0.08858 | 2.87E-13 | -0.36222 | 0.0455 | - | - | 0.01647 |
| | BMI~GRS+SMK+SMK*GRS | 0.07959 | 2.68E-09 | -4.85599 | 0.087 | 0.04993 | 0.113 | 0.01719 |
| 97 known + 6 novel | BMI~GRS | 0.09259 | 2.19E-15 | - | - | - | - | 0.01811 |
| | BMI~GRS+SMK | 0.09269 | 2.00E-15 | -0.36242 | 0.0451 | - | - | 0.01926 |
| | BMI~GRS+SMK+SMK*GRS | 0.08285 | 1.18E-10 | -5.57166 | 0.0532 | 0.05482 | 0.0701 | 0.02019 |
| 97 known + 6 novel + 3 int | BMI~GRS | 0.09224 | 6.39E-16 | - | - | - | - | 0.0188 |
| | BMI~GRS+SMK | 0.09225 | 6.19E-16 | -0.35696 | 0.0484 | - | - | 0.01992 |
| | BMI~GRS+SMK+SMK*GRS | 0.08158 | 8.69E-11 | -6.13026 | 0.0338 | 0.05914 | 0.0452 | 0.02106 |
| 77 known | WCADJ~GRS | 0.14954 | <2e-16 | - | - | - | - | 0.02585 |
| | WCADJ~GRS+SMK | 0.1475 | <2e-16 | 0.83181 | 8.30E-05 | - | - | 0.03023 |
| | WCADJ~GRS+SMK+SMK*GRS | 0.15436 | <2e-16 | 3.58699 | 0.243 | -0.03561 | 0.369 | 0.03046 |
| 77 known + 11 novel | WCADJ~GRS | 0.14768 | <2e-16 | - | - | - | - | 0.02908 |
| | WCADJ~GRS+SMK | 0.14571 | <2e-16 | 0.82427 | 9.38E-05 | - | - | 0.03338 |
| | WCADJ~GRS+SMK+SMK*GRS | 0.1518 | <2e-16 | 3.70027 | 0.27 | -0.03167 | 0.39 | 0.03358 |
| 77 known + 11 novel + 2 int | WCADJ~GRS | 0.14445 | <2e-16 | | - | - | - | 0.02832 |
| | WCADJ~GRS+SMK | 0.1425 | <2e-16 | 0.82549 | 9.22E-05 | - | - | 0.03264 |
| | WCADJ~GRS+SMK+SMK*GRS | 0.14908 | <2e-16 | 4.04436 | 0.24 | -0.03431 | 0.349 | 0.03289 |
| 66 known | WHRADJ~GRS | 0.0007688 | 1.11E-07 | - | - | - | - | 0.008165 |
| | WHRADJ~GRS+SMK | 0.0007543 | 1.61E-07 | 0.0137037 | 5.61E-11 | - | - | 0.02049 |
| | WHRADJ~GRS+SMK+SMK*GRS | 0.0008785 | 3.71E-08 | 0.0554007 | 0.0169 | -0.0006662 | 0.0709 | 0.02142 |
| 66 known + 6 novel | WHRADJ~GRS | 0.0008429 | 1.02E-09 | - | - | - | - | 0.01079 |
| | WHRADJ~GRS+SMK | 0.0008294 | 1.49E-09 | 0.0136828 | 5.66E-11 | - | - | 0.02308 |
| | WHRADJ~GRS+SMK+SMK*GRS | 0.0009172 | 1.81E-09 | 0.0449584 | 0.0585 | -0.0004602 | 0.1864 | 0.02357 |
| 66 known + 6 novel + 7 int | WHRADJ~GRS | 0.0006968 | 8.58E-09 | - | - | - | - | 0.009597 |
| | WHRADJ~GRS+SMK | 0.0006777 | 1.78E-08 | 0.0135856 | 8.01E-11 | - | - | 0.0217 |
| | WHRADJ~GRS+SMK+SMK*GRS | 0.0007379 | 3.05E-08 | 0.0375582 | 0.1 | -0.0003265 | 0.292 | 0.02202 |

[a] All outcome traits were adjusted for age, $age^2$ and sex. Waist circumferrence and waist-hip ration were additionally adjusted for BMI.

**SUPPLEMENTARY NOTE 1**. **Look-up of previously identified loci in our data set**

To fully explore the efficacy of accounting for smoking in GWAS of adiposity traits, we conducted a look-up in our data of recently published SNP associations with BMI, WHRadjBMI, and WCadjBMI identified in well-powered GWAS meta-analyses that did not account for SMK status[1,2]. Although our sample size was as little as one third of previously published GWAS[1,2], the majority of these loci (92% for BMI, 97% for WCadjBMI, and 92% for WHRadjBMI) reached Bonferroni corrected significant for at least one of the three Approaches in the current study.

All previously identified 97 BMI-associated SNPs were nominally significant ($P<0.05$) in Approach 1 (SNPadjSMK) for BMI including the sex-specific loci, 95 of the 97 for Approach 2 (SNPjoint), and seven for Approach 3 (SNPint). A total of 86 loci reached Bonferroni-corrected significance ($P<5.15x10^{-4}$) for Approach 1, 85 for Approach 2, and none for Approach 3. Finally, 41 loci from Approach 1 and 39 of the 97 from Approach 2 reached genome-wide significance (GWS, $P<5x10^{-8}$) (44 in total, 45%) (**Supplementary Table 11**). Of the 97 previously identified main effects loci for BMI, 3 of these were genome-wide significant GWS for women-only, 3 for men-only and the remaining in the sex-combined analysis in the previous publication. It is also worth noting that we report results for the All Ancestries meta-analysis, as this was our primary meta-analysis data-set; however, Locke et al. (2015) considered their European-descent only meta-analysis their primary data-set.

Of the 77 previously-identified WCadjBMI loci, 3 of these were GWS for women-only, 3 for men-only and the remaining in the sex-combined analysis as reported in Shungin et al[2]. Of these, 75 were nominally significant for Approachch 1 (SNPadjSMK) and Approach 2 (SNPjoint), and 5 for Approach 3 (SNPint). A total of 73 were Bonferroni-corrected significant ($P<6.49x10^{-4}$) for Approach 1 and 2; with 41 and 40 reaching GWS, respectively (43 non-overlapping, 56%) (**Supplementary Table 12**).

Eleven of the 68 previously published WHRadjBMI SNPs were associated in the women-only analyses in the previous investigation[2]. Of the 68 variants, 64 were nominally significant for Approach 1 (SNPadjSMK), 59 for Approach 2 (SNPjoint), and 10 for Approach 3 (SNPint). A total of 61 were Bonferroni-corrected significant ($P<6.49x10^{-4}$) for Approach 1 and 38 for Approach 2; with 36 and 8 reaching GWS, respectively (36 in total, 53%) (**Supplementary Table 13**).

In summary, we replicated all previously-identified BMI loci using one or more of our approaches ($P<0.05$ and concordant direction of effect), but did not replicate all previously-identified loci for WCadjBMI and WHRadjBMI in our current analyses. It is unclear if the lack of replication of previous findings is due to smaller sample size, patterns of linkage disequilibrium in our all ancestries sample, the adjustment of smoking status in the current discovery analysis, or even a combination of these factors.

**SUPPLEMENTARY NOTE 2. Summary of literature search on genes nearest to the 21 novel loci and all GxSMK interaction loci.**

We used SNIPPER (http://csg.sph.umich.edu/boehnke/snipper/) to identify potential biological functions of genes ±500kb of our novel association signals and those from Approach 3 (SNPint) for further investigation, and present a summary of those findings in this section (**Online Methods**).

**Body Mass Index (BMI)**

**rs2481665 (*INADL*)**: There are seven genes within the 500kb region of the lead SNP rs2481665 on chromosome 1. These genes are *INADL, L1TD1, KANK4, USP1, DOCK7, TM2D1,* and *ANGPTL3*. The lead SNP is in intron (#15) of the *INADL* (InaD-Like) gene. *INADL* encodes the protein Palsi1-Associated Tight Junction (PATJ), which helps regulate the formation of tight junctions, and is involved in the processes of cell polarization and directional migration of epithelial cells[3,4]. A GWAS study (n= 815) designed to identify variants associated with childhood obesity in the Hispanic population, found near genome-wide significant associations between the exonic, non-synonymous SNP rs1056513 in *INADL* (204 kb downstream from our lead SNP) and the following fat distribution traits: weight [kg] (EAF[effect allele frequency]: 0.031, p-value: $1.18 \times 10^{-07}$); BMI [kg/m$^2$] (EAF: 0.021, p-value: $8.34 \times 10^{-06}$); fat mass [kg] (EAF: 0.035, p-value: $1.59 \times 10^{-07}$); trunk fat mass [kg] (EAF: 0.035, p-value: $2.36 \times 10^{-07}$); fat free mass [kg] (EAF: 0.034, p-value: $2.80 \times 10^{-07}$) and hip circumference (EAF: 0.022, p-value: $2.47 \times 10^{-6}$).[5] The SNP rs1056513 accounted for 3% of the variance in body weight and body composition[5]. However, this SNP is not in LD with the lead SNP rs2481665 in this study ($R^2$<0.2).

Farther away is the *DOCK7* gene, 326 kb downstream from the lead SNP. This gene encodes a guanine nucleotide exchange factor (GEF) protein that is involved in axon formation and neuronal polarization. GWAS studies have reported the association of variants located near the *DOCK*7 gene with lipid levels. A GWAS study (n= up to 18,554) conducted with individuals of European ancestry identified the association of rs1213033 with triglycerides (eaf: -0.11, $2 \times 10^{-8}$)[6]. Another GWAS meta-analysis found a genome-wide significant association between rs1168013 and triglycerides in individuals of European ancestry (n=17,723; eaf: 0.035 (0.007), p-value: $6.4 \times 10^{-8}$)[7]. However, authors could not replicate this finding in other study samples consisting of 37,774 Europeans and 9,665 individuals of Indian Asian ethnicity. A GWAS replication study assessing the association between 15 SNPs and blood lipid and lipoprotein concentrations in individuals of Asian descent (n=4638), found a marginal association between the variant rs10889353, located in the intronic region of *DOCK7*, and triglycerides (eaf: -0.08, p-value: $6.5 \times 10^{-04}$)[8]. None of the variants from the different GWAS studies discussed above are in LD with SNP rs2481665 ($R^2$<0.2).

*TM2D1* is another gene in the 500kb area that is 404 kb upstream from rs2481665. This gene encodes a beta-amyloid peptide-binding protein (BBP), which is involved in neural death and in the decrease of cognitive skills that occurs in Alzheimer's disease**.** This protein may be targeted by the beta-amyloid peptide which has been linked to the formation of plaques resulting in neurotoxicity in Alzheimer's disease[9]. The APP, the precursor of beta-amyloid peptide, is expressed in adipose tissue and its expression is up-regulated in obesity[10,11].

*ANGPTL3* (Angiopoietin-Like 3) is 469 kb upstream from the lead SNP, and upstream of the *DOCK7* gene. *ANGPTL3* encodes a protein that plays a role in angiogenesis. This protein is expressed mostly in the liver. Mutations in this gene lead to the disease familial hypobetalipoproteinemia type 2 (*FHBL2*), which causes low levels of apolipoprotein B (apoB), total cholesterol, low-density lipoprotein (LDL) cholesterol and high density lipoprotein cholesterol [12]. Several genetic association studies suggest that ANGPTL3 has a role in regulating plasma lipoprotein metabolism[6,8,13,14]. A few single-nucleotide polymorphisms, near the *ANGPTL3* gene, have been associated with lower triglyceride: rs1213033, rs213192, rs12042319[6]. One of these, rs1213033, is also near the *DOCK7* gene[6].

There are several nearby genes with no documented role in adiposity or related cardiometabolic traits. Including, *L1TD1* (Line-1 type transposase domain containing 1) located 66 kb upstream from the lead SNP. *L1TD1* encodes the protein ES Cell-Associated Protein 11, a RNA-binding protein that plays a role in maintaining the pluripotency of stem cells, and in the proliferation of cancer cells[15,16]. Also, *KANK4* (KN

motif and ankyrin repeat domains 4) is a gene located 107 kb downstream from our SNP of interest. It encodes the protein Ankyrin Repeat Domain 38, a member of the Kank family of proteins, which are involved in the control of cytoskeleton microfilaments by regulating the polymerization of actin. The Kank gene is a tumor suppressor in renal cell carcinoma[17]. *USP1*, 307 kb upstream from rs2481665, encodes a protein that cleaves ubiquitin, a peptide that is added to proteins to signal them for degradation, or modification of their cellular location or enzymatic activity.

The intronic rs2481665 variant does not seem to have a functional role (Score 4 in RegulomeDB[18]). Two eQTLs were found for rs2481665 (Gene: L1TD1, p-value: $2.1 \times 10^{-7}$, EAF: -0.73, tissue: brain-cerebellum) and (Gene: INALD, p-value: $4.0 \times 10^{-6}$, EAF: 0.29, tissue: heart-atrial appendage).

**rs10929925 (*LOC400940*)**: *LOC400940* and *SOX11* are the two genes on Chr2 that are within 500 kb of the lead SNP rs10929925. SNP rs10929925 is downstream of *LOC400940*, the nearest gene, a non-coding RNA gene that remains uncharacterized. The variant is also 314 kb downstream from *SOX11*, a gene without introns that encodes a transcription factor that is part of the SOX (SRY-related HMG-box) family. This family of transcription factors is involved with processes that regulate embryonic development and cell fate[19]. One study has proposed that *SOX11* has a role in brain development after observing that mutations in the gene may lead to microcephaly, developmental delays and other features found in mild Coffin-Siris Syndrome, a genetic disorder that causes developmental delays[20]. A recent GWAS meta-analysis study of fat distribution, which included 224,459 individuals of European and non-European ancestry, identified a genome wide significant association ($p=4.5 \times 10^{-8}$) between rs10929925 and hip circumference unadjusted for BMI [2]. Based on a literature review, the study identified *SOX11* as the best candidate gene for rs10929925.[2]

There is no available information regarding the potential regulatory role of the lead SNP (RegulomeDB[18]). But there is evidence of an eQTL, although it does not reach 5% FDR (Gene: *SOX11*, P-value: $8.7 \times 10^{-6}$, Effect size: 0.39, Tissue: thyroid). In brain tissue, the SNP altered the TATA box motif of the Dlx3 gene a homeodomain gene (HaploReg[21]).

**rs6794880 (*SRRM1P2):*** The 500kb region around the lead SNP, rs6794880, does not show the presence of any protein coding genes. The nearest genomic feature to rs6794880 is SRRM1P2, a pseudogene, named the serine/arginine repetitive matrix 1 pseudogene 2. Upstream rs6794880 is *LINC00971*, a long intergenic non-protein coding RNA gene that remains uncharacterized.

There is no evidence that the lead SNP rs6794880 has a functional/regulatory role (Score 6 in RegulomeDB[18]) in the genome. Additionally, there are no reports of eQTLs for this variant.

**rs12629427 *(EPHA3)*:** There is only one gene found within 500kb of the peak signal, rs12629427. *EPHA3* (EPH receptor A3) is 11kb downstream from rs12629427, and is a member of the ephrin receptor subfamily of the protein-tyrosine kinase family. EPH and EPH-related receptors have been implicated in mediating developmental events, particularly in the nervous system. This gene encodes a protein that binds ephrin-A ligands. *EPHA3* has been implicated in the pathogenesis of lung cancer[22-26]. The SNP rs12629427 has a score of 6 in RegulomeDB[18] (minimal binding evidence). No significant eQTLs were found for rs12629427 and no GWAS hits were identified within the 1MB region of the lead SNP.

**rs2173039 (*EPHA3*)**: There is only one gene found within 500kb of rs2173039, which is 14.5kb upstream from EPHA3 (EPH receptor A3). See rs12629427 above.

**rs13069244 (*CCDC39*)**: A total of 4 genes are found within 500kb of the lead marker, rs13069244. *CCDC39* (coiled-coil domain containing 39) is located 43.88kb downstream from the lead marker and encodes a protein involved in the motility of cilia and flagella. Defects in this gene cause primary ciliary dyskinesia type 14. Lung disease was worse in those with IDA/CA/MTD ultrastructural defects, most of whom had biallelic mutations in *CCDC39*[27]. *FXR1* (fragile X mental retardation, autosomal homolog 1) is located 189kb downstream from rs13069244, and codes for an RNA binding protein that shuttles between the nucleus and cytoplasm, and is associated with polyribosomes, predominantly with the 60S ribosomal subunit. Deregulation of FXR protein 1 by the lipodystrophic lamin A p.R482W mutation elicits a myogenic gene expression program in preadipocytes[28]. *DNAJC19* (DnaJ (Hsp40) homolog, subfamily C, member 19), located 260kb upstream from our lead marker, encodes a protein involved in the ATP-dependent transport of transit peptide-containing proteins from the inner cell membrane to the mitochondrial matrix. Defects in this gene are a cause of 3-methylglutaconic aciduria type 5 (MGA5), also known as dilated cardiomyopathy with ataxia (DCMA)[29-31]. The loss of DNAJC19/PHB complexes affects cardiolipin acylation and leads to the accumulation of cardiolipin species with altered acyl chains[32]. There is no evidence that rs13069244 has a functional/regulatory role (RegulomeDB[18] Score 6: minimal binding evidence) in the genome. No GWAS hits were identified within the 1Mb region of rs13069244 and no report of eQTL for the variant.

**rs336396 (*INPP4B*)**: There are two genes found within 500kb of rs336396. The SNP lies within *INPP4B* (inositol polyphosphate-4-phosphatase, type II, 105kDa), which encodes inositol polyphosphate 4-phosphatase type II, one of the enzymes involved in phosphatidylinositol signaling pathways. INPP4B has been identified as a tumor suppressor by negatively regulating normal and malignant cell proliferation through regulation of the PI3K/Akt signaling pathway[33,34]. Different residues within the catalytic site of INPP4B are responsible for activity with lipid and protein substrates[35]. *IL15* (interleukin 15) is located 407kb upstream of rs336396. *IL15* encodes a cytokine that regulates T and natural killer (NK) cell activation and proliferation. This cytokine may act as an antagonist to IL2, which binds common hematopoietin receptor subunits, and may compete for the same receptor. This cytokine induces the activation of JAK kinases, as well as the phosphorylation and activation of transcription activators STAT3, STAT5, and STAT6. Murine models show that this cytokine may increase expression of apoptosis inhibitor BCL2L1/BCL-x(L), possibly through the transcription activation activity of STAT6, and thus prevent apoptosis. Cigarette smoke compromises IL-15 production – and as a result NK cell function – which could link to the higher incidence of cancers or viral infections observed among smokers[36]. A group of SNPs, upstream from *IL15*, were associated with both smoking status and quantity of cigarette consumption[37]. No data was provided for rs336396 by RegulomeDB[18]. No GWAS hits were identified within the 1Mb region of rs336396 and no report of an eQTL for the variant.

**rs12902602 (*CHRNA5-CHRNA3-CHRNB4*)**: A total of 10 genes are found within 500kb of rs12902602. The SNP is located 33.81kb upstream of *CHRNB4* (cholinergic receptor, nicotinic beta 4). The *CHRNA5-CHRNA3-CHRNB4* gene cluster has consistently been associated with smoking quantity and nicotine dependence[38-40], COPD, lung cancer and peripheral artery disease[39,41,42], and increased risk of death[43]. Variants of *CHRNA5-CHRNA3-CHRNB4* have also been associated with lower birth weight from smoking mothers[44], and with lower BMI in current adult smokers[45,46], but with lower BMI in never smokers[46]. The *CHRNA5-CHRNA3-CHRNB4* genes encode the nicotinic acetylcholine receptor (nAChR) subunits α3, α5 and β4 that are expressed in mammalian brain[47,48]. GWASs have also identified loci at *ADAMTS7* (ADAM metallopeptidase with thrombospondin type 1 motif 7), at 84.14 kb downstream from the leader SNP rs12902602, associated with coronary artery disease and its risk factors[49-52].

**Waist Circumference adjusted for BMI (WCADJBMI):**

**rs17396340 (*KIF1B*)**. A total of 10 genes are found within 500kb of the lead marker, rs17396340, which is intronic to *KIF1B*. We highlight four genes in the region here. *KIF1B* is involved in synaptic vesicle and mitochondrial transport, and may play a critical role in the development of hepatocellular carcinoma[53]. *6PGD* codes for an oxidative carboxylase responsible for reduction of 6-phosphogluconate. Cells lacking 6PGD appear to metabolize glucose as an inhibitor to induce senescence[54]. *RBP7* is involved in carotenoid metabolism. In avian model organisms, the *RBP7* promoter is important in regulating expression of several genes in adipose tissue at later developmental stages[55]. Nicotinamide mononucleotide adenylyltransferease (*NMNAT*) reversibly catalyzes the important step in the biosynthesis of NAD from ATP and NMN. NAD and NADP are used reversibly in anabolic and catabolic reactions. NAD is necessary for cell survival in oxidative stress and DNA damage. The top SNP, rs17396340, is associated with the expression levels of ARSA (p-value of 6.0e-05) at LCL tissue in *Homo sapiens*. Human adipocytes express functional DAR (Dopamine receptors) and ARSA, suggesting a regulatory role for peripheral dopamine in adipose functions[56]. It is speculated that the propensity of some DAR-activating antipsychotics to increase weight and alter metabolic homeostasis is due to their direct action on adipose tissue. Our lead SNP is also associated with mean platelet volume[57]. From HaploReg[21], the lead SNP, rs17396340, is annotated as KIF1B in GENCODE, and is functionally annotated as intronic. This lead SNP is associated with enhancer histone marks in 9 tissues; associated with regulatory motifs at GATA and Hoxa5; and with cis-eQTLs from various tissues (cells transformed fibroblasts, muscle skeletal, lymphoblastoid EUR exonlevel, lymphoblastoid EUR genelevel, and whole blood). The RegulomeDB[18] score for the lead SNP is 4.

**rs6743226 (*HDLBP*).** A total of 10 genes are found within 500kb of our lead marker, rs6743226. Three, of biological interest, are mentioned here. Our lead SNP, rs6743226, is intronic to *HDLBP,* which codes for a protein that binds high density lipoprotein (HDL) that functions to regulate excess cholesterol levels in cells.

*STK25* codes for a serine/threonine kinase with important functions in the Golgi apparatus. This gene has been associated with severe hypoxia[58] and pseudohypoparathyroidism, symptoms of which include short stature and obesity[59]. Significantly higher serine/threonine kinase 25 (STK25) levels were observed in the skeletal muscle of type 2 diabetic patients, compared with individuals with normal glucose tolerance[60]. The overexpression of STK25 in conditions of excess dietary fuels associates with a shift in the metabolic balance in peripheral tissues from lipid oxidation to storage, leading to a systemic insulin resistance[61].

Expression of PAS domain containing serine/threonine kinase (*PASK*) is regulated by glucose and the encoded protein plays a role in the regulation of insulin gene expression. Down regulation of this gene may play a role in type 2 diabetes[62-64]. Far2 and Stk25 are candidate genes for the HDL cholesterol locus in mice[65]. The top SNP, rs6743226, is associated with the expression of B-cell CLL/lymphoma 10 (BCL10). The protein encoded by the gene *BCL10* contains a caspase recruitment domain (CARD), and induce apoptosis and to activate NF-kappaB MALT1 and this protein are thought to synergize in the activation of NF-kappaB, and the deregulation of either of them may contribute to the same pathogenetic process that leads to the malignancy[66].

There is no GWAS signal nearby the lead SNP rs6743226. This lead SNP is associated with enhancer histone marks in 4 tissues; associated with regulatory motifs changed at Goxa and TCF12; and with eQTL from various tissues including adipose subcutaneous, lung, and muscle tissues. The RegulomeDB[18] score for the lead SNP is 6.

**rs4378999 *(DOCK3*):** A total of 4 genes are found near our lead marker, rs4378999, *DOCK3*, *MANF*, *VPRBP*, and *RBM15B*. Our lead variant is intronic to *DOCK3* (dedicator of cytokinesis 3), which is highly expressed in the central nervous system and like previously identified obesity related genes, is involved in neurite outgrowth downstream of BDNF-TrkB[67]. *MANF* (mesencephalic astrocyte-derived neurotrophic factor) is an endoplasmic reticulum protein that acts to protect ER in response to cellular/organismal stress [68], for example, expression is increased in skeletal muscle of the leg in rats in response to exercise [69]. Further, recent evidence shows that *MANF* may be an important factor in the protection of pancreatic beta cells and disruption of *MANF* expression can lead to diabetes [68]. There is very little known about *VPRBP*, and *RBM15B.*

Genome-wide association studies have reported the association within 1MB region of lead SNPs for height ($R^2$=0.35)[70,71] and melanoma ($R^2$=0.48)[72]. Our lead SNP is associated with regulatory motifs changed at Cdx2; and with eQTL from various tissues including adipose subcutaneous, and muscle skeletal. The lead SNP is associated eQTL in esophagus muscularis tissue based on GTEx[73] lookup. GWAS studies have report the association within 1Mb of lead SNP for height ($R^2$=0.38)[71], and fibrinogen ($R^2$=0.41)[74]. The RegulomeDB[18] does not have data for lead SNP rs4378999.

**rs7697556 (*ADAMTS3*):** One gene is found within 500kb of our lead marker, rs7697556. ADAM metallopeptidase with thrombospondin type 1 motif, 3 (*ADAMTS3*) is located 80 kb upstream of our variant, rs7697556. While there is no established role for ADAMTS3 in obesity-related traits, there are a number of variants within and near this gene associated with relate anthropometric and cardiometabolic traits, including height[70,71], lipid metabolism[75], and metabolites[76]. From There is no score assigned for our lead SNP in the RegulomeDB[18].

**rs10269774 (*CDK6*):** A total of 10 genes are found within 500 kb of the lead marker, rs10269774. The SNP is located within an intron in cyclin-dependent kinase 6 (*CDK6*). CDK family members are important regulators of cell cycle progression. GWAS have reported associations between *CDK6* variants with height[70,71,77-81]. The *CDK6*-rs2282978 associated with height is in complete LD with our lead marker (rs10269774: $R^2$=1, D'=1). Also, GWAS identified associations between *CDK6* variants with white blood cell counts[82] and rheumatoid arthritis[83,84]. *CDK6* rs42041 is associated with juvenile idiopathic arthritis (JIA)[85], and patients with JIA are significantly shorter and more often overweight or obese than controls[86]. Research suggests that the microRNA-103a-3p controls proliferation and osteogenic differentiation of human adipose tissue-derived stromal cells by binding to specific target sequences in the *CDK6* mRNA 3'-untranslated region[87]. Another study in the human placental transcriptome found that *CDK6* mRNA levels correlated with offspring birth weight and birth weight percentiles[88].

rs10269774 is located in enhancer regions (H3K4Me1 and H3K27ac) with histone modification enrichment in mammary epithelial tissue and lymphoblastoid cell lines. rs10269774 was suggested to have cis-acting associations with five gamma-glutamyltransferase (GGT) family gene expression in lymphoblastoid of Yoruba population (p=6E−05[89]). Elevated serum GGT is associated with waist circumference[90,91], BMI[91], visceral fat area[91], triglyceride levels[91], metabolic syndrome[90,92], coronary artery calcification[93] and biomarkers of atherosclerosis[94], arterial stiffness[95,96], incident CVD and death[92]. rs10269774 is located near to several transcription factor binding sites (*CTCF*, *EP300*, *JUN*, *POLR2A*, *FOS, NFIC*, and *RFX5*, among others).

**rs9409082 and rs9408815 (*TMEM38B*):** A total of 3 genes are found within 500 kb of the lead markers rs9409082 and rs9408815. At 364 kb downstream of rs9409082 is located *TMEM38B* (transmembrane protein 38B, 9q31.2) gene, which encodes an intracellular monovalent cation channel that functions in

maintenance of intracellular calcium release. Deletions in *TMEM38B* are associated with autosomal recessive osteogenesis imperfecta[97-99]. There is evidence of genome-wide association between rs9409082 with height[70]. Also, GWAS have reported several variants in this region associated with age at menarche[100-102], which is a risk factor to develop obesity, type 2 diabetes, cardiovascular disease, breast cancer and all-cause mortality[101]. However, the reported variants for age at menarche are in low-to-moderate LD (0.005 $<R^2<0.68$) with our lead marker from Approach 1, rs9409082. Variants on 9q31, in low LD with rs9409082, have shown suggestive association with visceral adipose to subcutaneous adipose ratio in men ($R^2$=0.161)[103] and with a protein quantitative trait locus modulating cellular response to chemotherapy ($R^2$=0.002)[104].

At 497.6 kb downstream of rs9409082 is the *FKTN* (fukutin, 9q31.2) gene that encodes a putative transmembrane protein of the cis-Golgi compartment. *FKTN* protein may be involved in the glycosylation of alpha-dystroglycan in skeletal muscle. Mutations in *FKTN* have shown association with congenital muscular dystrophy[105,106]. No significant eQTLs were found for SNP rs9409082 (GTEx[73], SNIPPER, RegulomeDB[18], and HaploReg[21]).

**rs6012558 (*ARFGEF2*):** A total of 11 genes are found within 500kb +/- of our lead SNP, rs6012558, which is 6,989 bp upstream of *ARFGEF2* (ADP-ribosylation factor guanine nucleotide-exchange factor 2). *ARFGEF2*'s primary function involves intracellular trafficking. Our lead variant is 86,866 bp upstream of *PREX1* (phosphatidylinositol-3,4,5-trisphosphate-dependent Rac exchange factor 1), a gene which encodes a protein involved in intraceullar signaling, lipid and protein binding, and regulation of GTPase activity [107-109]. *PREX1* is primarily expressed in the blood leukocytes and brain[107]. Recent mouse models indicate that PREX1 may be important for the regulation of thermogenic potential of brown adipose tissue and white preadipocytes, making this gene very important for energy expenditure[110]. Additionally, rs6012558 is a significant (<5% FDR) cis-acting expression quantitative trait locus (cis-eQTL) for *ARFGEF2* (subcutaneous adipose and sigmoid colon tissues), *CSE1L* (artery, thyroid, subcutaneous adipose, esophagus mucosa, and skeletal muscle tissues), and *STAU1* (transformed fibroblast cells) (GTEx[73]). Additional evidence that this variant lies in a potentially important regulatory region includes a RegulomeDB[18] score of 4 [18], it is nearby (<500kb +/- and $R^2>0.7$) other variants that rest in active enhancers for *ARFGEF2*, other cis-eQTLs for *ARFGEF2* (monocytes, whole blood, cerebellum, and temporal cortex), *DDX27* (monocytes), *C2orf199* (monocytes), *CSE1L* (whole blood), and *PREX1* (Cerebellum and Temporal Cortex) (HaploReg[21] and UCSC Browser[111]). Our lead SNP is within 500kb +/- of several previously identified GWAS SNPs for multiple traits, the nearest of which is rs6012564 associated with tendency toward anger (distance=10kb)[112]; however, all of these are in low LD with rs6012558 ($R^2<0.3$).

**rs4141488 (*GRIN2A*):** There are only two genes within 500 kb +/- of our lead SNP, rs4141488, which lies 218 kb downstream of *GRIN2A* (glutamate receptor, ionotropic, N-methyl D-aspartate 2A). The primary function of *GRIN2A* is to assist in controlling long-term memory and learning through regulation and efficiency of synaptic transmission. These receptors are essentially the gateway for calcium into post-synaptic cells[113]. Variants in this gene have been associated with various forms of epilepsy, sleep patterns, delayed psychomotor development, speech difficulties, seizures, mental retardation, and various mental disorders, including heroin addiction[114-120]. The only other gene within 500 kb of rs4141488 is C16orf72; little is known about the function of this gene. While GTEx[73] revealed no significant eQTLs nearby our lead variant, there is some evidence that this locus may lie within an important regulatory region. RegulomeDB[18] provided a score of 5 (minimal binding evidence) for rs4141488. Additionally, HaploReg[21] and UCSC browser show that our lead SNP and variants in high LD ($R^2>0.7$) are within active enhancer regions for several tissues, including liver, fetal leg muscle, smooth stomach and intestinal muscle, cortex, and several embryonic and pluripotent cell types; and within altered binding motifs for EWSR1-FLI1, Elf3,

STAT, CDP, HNF1, and SOX. Our lead SNP is within 500kb +/- of several previously identified GWAS SNPs for multiple traits, the nearest of which is rs17550532 associated with sudden cardiac arrest[121]. Other associations in this region include behavioral disinhibition[122], venous thromboembolism[123], and Transforming Growth Factor-β1 [5]; however, all of these are in low LD with rs4141488 ($R^2$<0.4).

**rs1545348 (*RAI14*):** Our lead SNP, rs1545348, lies within the intron of *RAI14* (Retinoic Acid Induced 14), although very little is known about the function of this gene in humans. There are four additional genes within 500 kb+/- of rs1545348, including *RAD1* (RAD1 checkpoint DNA exonuclease) 187 kb upstream. *RAD1* encodes a protein involved in stopping the cell cycle in response to DNA damage, as well as recruiting other proteins responsible for DNA repair[124,125], including in response to stress caused by cigarette smoke[126]. There is strong evidence of a regulatory role within the region surrounding our lead variant (RegulomeDB[18] score 4, minimal binding evidence). One significant (beta=-0.28, P=5.3E-6) eQTL between rs1545348 and *TTC23L* was found in sun exposed skin tissue (lower leg) (GTEx[73]). Additionally, HaploReg[21] and the UCSC browser reveal that the region surrounding our lead variant (+/- 500 kb, $R^2$>0.7) harbors marks of open and active chromatin and DNase hypersensitive regions across multiple tissues, including cancer, pluripotent, and normal tissue, brain and adipose tissue among others. Traits with nearby GWAS associations include several metabolite markers and left ventricular mass, although each of these associations are in low LD with rs1545348[127-131].

**rs6470765 (*GSDMC*):** There are three genes within 500 kb +/- of our lead SNP, rs6470765, which lies within an intron of *GSDMC* (gasdermin C). There is very little known about the function of *GSDMC*. Our lead SNP also lies 80 kb downstream of *FAM49B* (family with sequence similarity 49, member B). Similar to *CDK6*, a gene nearby another one of our novel variants, rs10269774, *FAM49B* is a target of *BACH1* transcription factor, which is involved in cellular response to oxidative stress and management of the cell cycle[132]. Also, *ASAP1* (ArfGAP With SH3 Domain, Ankyrin Repeat And PH Domain 1), a gene located 328 kb upstream of our association signal, may be involved in the differentiation of fibroblasts into adipocytes[133]. There is moderate evidence for the functional role of lead variant in regulation of gene expression (RegulomeDB[18] score of 6: minimal binding evidence). However, the GTEx[73] database indicates that rs6470765 is a significant eQTL for *GSDMC* in skeletal muscle, sun-exposed skin, and mucous in the esophagus. Furthermore, HaploReg[21] and the UCSC Browser highlight moderate evidence for regulatory elements in high LD >0.9, including DNAse hypersentive regions, and active enhancer and promotor regions in >20 tissue types (e.g. lung, adipose, skeletal muscle, epidermal and esophageal tissues, and many stem/pluripotent cell types). Our lead variant is within several altered binding sites for FOX1, FOX2 and SOX. Last, our lead SNP is in high LD with other potential cis-eQTLs for *GSDMC*. Nearby associations with other traits include height, hip circumference adjusted for BMI, and inflammatory bowel disorder[2,70,71,134].

**rs6076699 (*PRNP*)**: There are seven genes within 500 kb+/- of our lead SNP, rs6076699. The lead SNP is 100kb upstream of *PRNP* (prion protein) is likely a signaling transducer involved in multiple biological processes related to nervous system, immune system, and general cellular functions[135-138]. Mutations in the repeat region as well as elsewhere in this gene have been associated with Creutzfeldt-Jakob disease, fatal familial insomnia, Gerstmann-Straussler disease, Huntington disease-like 1, and kuru [139-145].

Alternate forms of the oligmers have been shown to form in response to oxidative stress caused by copper exposure[146]. Copper is present in cigarette smoke and elevated in serum of smokers, but is not outside of safe ranges according the U.S. Centers for Disease Control and Prevention, National Center for Chronic Disease Prevention and Health Promotion, and Office on Smoking and Health[147,148]. Our lead SNP is 136 kb upstream from a related gene, *PRND* (prion protein 2), which is biochemically and structurally similar to *PRNP*[149]. Like PRNP, mutations in this gene may also be involved in neurocognitive disorders, although

there are only weak associations[150,151]. A third prion protein (testes specific, *PRNT*) is found 145 kb away from our lead SNP; however no much is known about the function of this gene. Other nearby genes include *SLC23A2* (Solute Carrier Family 23 [Ascorbic Acid Transporter], Member 2), *ADRA1D* (Adrenoceptor Alpha 1D), *SMOX* (Spermine Oxidase), and *RASSF2* (Ras association [RalGDS/AF-6] domain family member 2). *SLC23A2* is essential for the uptake and transport of Vitamin C, which is an important nutrient for DNA and cellular repair in response to oxidative stress both directly and through supporting the repair of Vitamin E after exposure to oxidative agents[152-155]. Furthermore, this region is associated with success in smoking cessation and is implicated in addictive behaviors in general[156,157]. Nearby GWAS-identified associations include preeclampsia, and height[70,71,158]. There is little evidence that our association signal is involved in regulation of gene expression (RegulomeDB[18] score-5: minimal binding evidence) [18]. While our tag SNP is located within an active enhancer region (open chromatin marks, DNAse hypersentivity, and several transcription factor binding motifs), this activity appears tissue specific (sex-specific tissues and lungs)[21,111]. There are no other significant regulatory elements in high LD with rs6076699[21,73].

**Waist-to-Hip Ratio adjusted for BMI (WHRadjBMI)**

**rs670752 (*BBX*):** There are only three genes within 500 kb+/- of our lead SNP, rs670752, which lies within an intron of *BBX* (Bobby Sox Homolog [Drosophila]). While there is little known about the function of *BBX*, another nearby intronic variant, rs6437740, has been associated with smoking behavior in a previous GWAS[159]. Other nearby genes include *CCDC54* (coiled-coil domain containing 54) and *CD47* (CD47 molecule). Much is known about the function of *CD47* due to mouse models. *CD47* encodes a cell surface antigen involved in immune response to bacteria, cell adhesion, inflammatory response, and cell to cell signaling[160-162]. *CD47* expression is significantly decreased in obese individuals and negatively correlated with BMI, WC, and HIP in RBC [163].

Conversely, in mouse models, *CD47* deficient mice show decreased weight gain on high fat diets, increased energy expenditure, improved glucose profile, and decreased inflammation[164]. Our lead SNP, rs670752, has a score of 6 (very little binding evidence) in RegulomeDB[18] and no significant eQTLs were identified in GTEx[73]. However, our tag SNP was identified as a significant eQTL for *BBX* in brain tissue in HaploReg[21], Additionally, multiple SNPs in high LD with rs670752 provide several lines of evidence for nearby regulatory elements (e.g. active promoters, transcription factor binding motifs, strong and poised enhancers), mostly in pluripotent and embryonic cell lines, but also blood cell lines and brain tissue[21,111].

**rs589428 (*EHMT2*)**. A total of seventy-seven genes are found near our lead SNP, rs589428, which is intronic within *EHMT2* (Euchromatic Histone-Lysine N-Methyltransferase 2). *EHMT2* encodes a histone methyltransferase, a group of genes involved in repression of transcription through the regulation of chromatin state [165]. The lead SNP is 302kb downstream of *TNF*. In patients with end-stage renal disease (ESRD) on long-term hemodialysis (HD), the SNP in the promoter region of the *IL-6* and *TNF-alpha*, and *IL-10*, show a strong association with indices of comorbidity and function, and biological and nutritional markers[166]. TNF-alpha promotes bone loss and inhibits bone formation and has an important role as a mediator of skeletal damage in inflammatory arthritis[167-170]. *TNF* is the master regulator of other inflammatory cytokines and the major cytokine in the pathogenesis of chronic inflammatory disease[171]. TNF-alpha exerts an important influence on adipose tissue metabolism and function. It inhibits the expression of two major adipose tissue differentiation regulators: CCAAT and PPARγ-2 [172]. TNF-alpha promoter methylation levels could be involved in the susceptibility to stroke[173] and correlates with increased risk of coronary artery disease[174]. The risk of early childhood wheeze associated with early maternal smoking may be modified by TNF[175]. The lead SNP is also 287kb upstream of *NCR3*, which is associated with pulmonary function[176].

The top SNP is 17.5kb upstream of *NEU1* (Sialidase 1 (Lysosomal Sialidase)). The activity of *NEU1* is higher in epididymal fat and lower in the livers of two strains of obese and diabetic mice. Fluctuations in *NEU1* activity might be associated with the pathological status of these tissues in obesity[177]. The lead SNP is 50kb downstream of *HSPA1B*. Functional *HSPA1B* variants are associated with lung cancer risk and survival[178]. The top SNP is 65kb upstream of *CFB*. Increased concentrations of circulating binding factors fH and fB in subjects with altered glucose tolerance could reflect increased SVC-induced activation of the alternative pathway of the complement in omental adipose tissue linked to insulin resistance and metabolic disturbances[179]. The top SNP is 91kb upstream of *STK19*, which has been reported to be a pleiotropic gene for metabolic syndrome and inflammation and is associated with TG, BMI, WAIST, SBP and inflammatory markers including plasminogen activator inhibitor 1 (PAI-1) and white blood cell count (WBCC)[180]. Our top snp is 102kb upstream of *C4A*, which was identified as novel potential adipokine candidate regulator of obesity and adipose regions [181] between visceral and subcutaneous adipose tissue. The Top SNP is 102kb upstream of *C4B*. The carriers of C4B*Q0 (silent allele for the C4B gene) have a substantially increased risk to suffer from myocardial infarction or stroke. Compared to controls, C4B*Q0 carrier frequency was significantly higher at diagnosis in Icelandic smokers with angina pectoris (AP) or acute myocardial infarction (AMI) and Hungarian smokers with severe coronary artery disease, while no such difference was seen in nonsmokers. These findings indicate that C4B*Q0 genotype can be considered as a major covariate of smoking in precipitating the risk for AMI and associated mortality[182]. The top SNP is 150kb upstream of *DDAH2* in which SNP rs9267551 may confer increased risk for type 2 diabetes by affecting insulin sensitivity through increased asymmetric dimethylarginine (ADMA) levels [183,184].

Our top SNP is 222kb downstream of *APOM*. The PCSK9 pathway contributes to plasma apoM regulation in humans and the influence of PCSK9 on circulating apoM appears to be modified by adiposity [185]. In addition, APOM expression is related to FEV1/FVC (forced expiratory volume 1/ forced vital capacity) ratio and per cent emphysema [186]. The top SNP is 261kb downstream of *AGER/RAGE*. The lower level of soluble RAGE/AGER is associated with a number of components of metabolic syndrome (central obesity, hypertension, and hyperglycemia) [187]. Soluble RAGE is inversely associated with pancreatic cancer risk among Finnish male smokers [188]. The RAGE(2) haplotype is associated with diabetic nephropathy (DN) in type 2 diabetics and with earlier DN onset and, thus, can be regarded a marker for DN [189]. RAGE, via its interaction with ligands, serves as a cofactor exacerbating diabetic vascular disease [190]. Serum endogenous secretory RAGE (esRAGE) levels were inversely correlated with BMI and serum HDL-cholesterol[191]. In healthy subjects plasma levels of sRAGE were negatively correlated with BMI and waist/hip ratio supporting a possible protective role for these proteins before any evidence of diabetic or vascular complications[192].

The top SNP is 263 downstream of *AIF1*. The serum AIF-1 concentrations were positively correlated with levels of fasting plasma glucose, hemoglobin A1c, triglycerides, and uric acid, and with WC and BMI, and were inversely correlated with HDL cholesterol levels[193]. Also, the variants in AIF1 show evidence of association with adult obesity in the Greek population[194]. The top SNP is 306 downstream of *LTA*. SNPs in LTA are associated with chronic kidney disease in Type 2 diabetes[195]. The variability of LT-alpha genotypes may have potential implications for individual susceptibility to asthma in atopic or in ever-smoking Chinese adults in Hong Kong[196].

The genome-wide association studies have reported the associations within 1Mb of region for age at menopause ($R^2$=0.32)[197], telomere length ($R^2$=0.22)[198], idiopathic membranous nephropathy[199] ($R^2$=0.45), chronic hepatitis B infection[200] ($R^2$= 0.45) and phospholipid levels (plasma) ($R^2$=0.23)[201]. This lead SNP is associated with regulatory motifs changed at Bcl6b, NF-kappaB, Pou5f1; associated with enhancer histone

marks in stomach mucosa, HSMM cell derived skeletal muscle myotubes cell tissue; and in eQTL in various tissues including subcutaneous adipose, visceral omentum, lung and skeletal muscle tissues. The lead SNP is associated with eQTL in tibial artery and blood tissues from GTEx[73] analysis. The RegulomeDB[18] score for the lead SNP is 1f.

**rs1856293** ***(EYA4)***: A total of nine genes are found near our lead SNP, rs1856293. The lead SNP is 342kb downstream of *RPS12*. *RPS12* is a potential target gene of microRNA-377, which has been consistently upregulated in *in vitro* diabetic nephropathy (DN) models and in *in vivo* DN mouse models[202]. If *RPS12* is also upregulated in the diabetic milieu, it may contribute to the progression of DN. *RPS12* has been reported to be a strong candidate for diabetic nephropathy[203]. In addition, in the study of E3 rats, there were significant positive correlations between TG and the expression of *RPS12* gene[204]. The lead SNP is 83kb upstream of *EYA4*. Serum methylation levels of *EYA4* were significant discriminants between stage I colorectal cancer and healthy controls [205] and high methylation of the EYA4 gene is associated with ulcerative colitis with colorectal cancer[206]. The lead SNP is 446kb upstream of *VNN1*. Alternative splicing in *VNN1* is associated with colorectal cancer[207]. The combination of VNN1 and MMP9 may be used as a blood biomarker panel for the discrimination of pancreatic cancer-associated diabetes from type II diabetes[208]. There is no reported GWAS signal in high LD with the lead SNP. This lead SNP is associated with regulatory motifs changed at Esr2, LRH1, Myf_3, Sin3Ak-20_disc3 and T3R; and associated with enhancer histone marks in ESDR, SKIN and brain tissue. The RegulomeDB[18] score for the lead SNP is 6.

**rs2001945 (*TRIB1*):** There are five protein coding genes within 500 kb+/- of our lead SNP, rs2001945, which lies 27 kb downstream from *TRIB1*. *TRIB1* (tribbles pseudokinase 1) encodes a protein involved in ATP binding and the MAPK/ERK1/2 pathway[209]. Very little is known about the function of the other nearby genes, including *NSMCE2* (non-SMC element 2, *MMS21* homolog), *KIAA0196* (strumpellin), *SQLE* (qualene epoxidase), and *ZNF572* (Zinc Finger Protein 572). GTEx[73] indentified no significant eQTLs for our lead SNP; however, RegulomeDB[18] provided a score of 4 (minimal binding evidence [Transcription Factor binding + DNase peak]). Further, HaploReg[21]/UCSC Genome Browser reveal multiple lines of evidence across multiple tissues, including cis-eQTLs between rs2001945 for *TRIB1* and *NSMCE2* in brain tissue, strong DNAse hypersensitivity clusters both at the association peak and across SNPs in high LD with our lead SNP, transcription factor binding motifs, and open chromatin marks primarily in Human Umbilical Vein Endothelial Cells (HUVEC). There are several nearby previously-identified GWAS signals for related cardiometabolic and digestion-related traits, including lipids (e.g. triglycerides, LDL, HDL)[6,8,13,14,210-217], adiponectin[218], liver enzyme levels[219], gestational age[5], inflammatory bowel disease[134], Crohn's disease[220,221], and metabolite levels[222].

**rs17065323 (*SMIM2*):** A total of 6 genes are found within 500 kb of the lead marker, rs17065323. The SNP rs17065323, which is located 23.19 kb downstream of the long intergenic non-protein coding RNA 284 (*LINC00284*, 13q14.11), showed suggestive association with uric acid levels (p=8.7E-6, [223]). Variants of the *LACC1* (laccase (multicopper oxidoreductase) domain containing 1), at 159.72 downstream of rs17065323, were genome-wide associated with Crohn's disease [134,221], and a *LACC1* mutant showed evidence of association with systemic juvenile idiopathic arthritis [224]. In addition, GWASs have suggested associations between variants on 13q14 with response to tocilizumab in rheumatoid arthritis (p=2E-7[225]), antineutrophil cytoplasmic antibody-associated vasculitis (p=3E-6[226]), and myotrophic lateral sclerosis (p=4E-6, [227]), as well as *SERP2* genotype-carbohydrate interaction influencing fasting insulin and homeostasis model assessment of insulin resistance (p=7E-6 and p=5E-6 , respectively [228]). The nearest protein-coding gene to our tag SNP is *SMIM2* (Small Integral Membrane Protein 2), located 89.5 kb upstream; however, very little is known about the function of *SMIM2*.

**rs1049281 (*HLA-C*):** Eighty-six genes are found within 500kb of rs1049281, which lies within the *HLA-C* gene at 6p21.3. *HLA-C*encodes an HLA class I heavy chain paralogue found in nearly all cells and important in the function of the immune system. There is strong evidence that our SNP is in a region likely to affect binding activity and gene expression in adipose tissue (RegulomeDB[18] score 1f). Over 100 alleles of the *HLA-C* gene have been described, and *HLA-C* has been associated with risk of various autoimmune diseases which can influence adiposity, including Type I diabetes, celiac disease, and psoriatic arthritis [229,230]. Our lead SNP is 314569 bp downstream of *DPCR1,* a gene associated with diffuse panbronchiolitis, a chronic inflammatory lung disease [231]. A variant near this gene (rs9368649), has been suggestively associated with smoking status (ever somker) and pack years (P~1.3E-07) [232], but not at GWS. This SNP is not in high LD with our lead SNP ($R^2$=0.152, D'=0.902). Our lead SNP is 190789 bp upstream of *HCP5*, a lncRNA. A variant (rs12175489) near this gene was suggestively associated (p=2.13E-06) with visceral adipose tissue (VAT) in men [103], but this variant is also not in high LD with our lead SNP ($R^2$=0.022, D'=0.478). Our lead SNP is 336394bp upstream of *AIF1*, 310030bp downstream of *NCR3*, and 341847 upstream of *BAT2*. Three variants in this region [rs2260000 ($R^2$=0.122, D'=0.526), rs1077393 ($R^2$=0.114, D'=0.434), and rs2844479 ($R^2$=0.100, D'=0.523) have been previously associated with variation in weight [233]. Another variant near *NCR3* (rs2070600) has been previously associated with ever-smoking and lung function, but is not in high LD with our lead SNP ($R^2$=0.137, D'=0.642) [176,232]. Our lead SNP is 340905bp downstream of *VARS2*, and a variant near this gene (rs7751505) has been suggestively associated with height change ($P<4.05 \times 10^{-6}$), though it is not in LD with our top SNP ($R^2$=0.054, D'=0.569). Two other variants in the region have been previously associated with extremes of height (p<5E-08), one of which is in strong LD with our lead SNP (rs2247056, 28923bp from rs1049281: $R^2$=0.814, D'=1.000; rs7741091: $R^2$=0.093, D'=0.652) [77].

**SUPPLEMENTARY NOTE 3**. **Detailed summary of eQTL methods and results.**

**eQTL Methods**

We used two approaches to systematically explore the role of novel loci in regulating gene expression. First, to gain a general overview of the regulatory role of newly identified GWAS regions, we conducted an eQTL lookup using >50 eQTL studies [234], with specific citations for >100 datasets included in the current query: 1) Blood cell related eQTL studies included fresh lymphocytes [235], fresh leukocytes [236], leukocyte samples in individuals with Celiac disease [237], whole blood samples [73,238-256], lymphoblastoid cell lines (LCL) derived from asthmatic children [257,258], HapMap LCL from 3 populations [259], a separate study on HapMap CEU LCL [260], additional LCL population samples [261-267],neutrophils [268,269],CD19+ B cells [270], primary PHA-stimulated T cells [261,264], CD4+ T cells [271], peripheral blood monocytes [267,270,272-275], long non-coding RNAs in monocytes [276] and CD14+ monocytes before and after stimulation with LPS or interferon-gamma [277], CD11+ dendritic cells before and after *Mycobacterium tuberculosis* infection [278] and a separate study of dendritic cells before or after stimulation with LPS, influenza or interferon-beta [279]. Micro-RNA QTLs [280,281], DNase-I QTLs [282], histone acetylation QTLs [283], and ribosomal occupancy QTLs [284] were also queried for LCL. Splicing QTLs [285] and micro-RNA QTLs [286] were queried in whole blood. 2) Non-blood cell tissue eQTLs searched included omental and subcutaneous adipose tissues[73,238,256,263,287], visceral adipose tissue [256], stomach [287], endometrial carcinomas [288], ER+ and ER- breast cancer tumor cells [289], liver [256,287,290-293], osteoblasts [294], intestine [295] and normal and cancerous colon [296,297], skeletal muscle [256,298], breast tissue (normal and cancer)[299,300], lung [73,301-304], skin [73,263,267,305], primary fibroblasts [261,264,306], sputum [307], pancreatic islet cells [308], prostate [309], rectal mucosa [310], arterial wall [256] and heart tissue from left ventricles [73,311] and left and right atria [312]. Micro-RNA QTLs were also queried for gluteal and abdominal adipose [313] and liver [314]. Methylation QTLs were queried in pancreatic islet cells [315]. Further mRNA and micro-RNA QTLs were queried from ER+ invasive breast cancer samples, colon-, kidney renal clear-, lung- and prostate-adenocarcinoma samples [316]; 2 Brain eQTL studies included brain cortex [252,272,317-319], cerebellar cortex [320],

cerebellum [289,318,321-323], frontal cortex [320,321,323], gliomas [324], hippocampus [320,323], inferior olivary nucleus (from medulla) [320], intralobular white matter [320], occiptal cortex [320], parietal lobe [322], pons [321], pre-frontal cortex [289,323,325,326], putamen (at the level of anterior commussure) [320], substantia nigra [320], temporal cortex [318,320,321,323], thalamus [323] and visual cortex [289].

Additional eQTL data was integrated from online sources including ScanDB (http://www.scandb.org/newinterface/about.html), the Broad Institute GTEx[73] Portal, and the Pritchard Lab (eqtl.uchicago.edu). Cerebellum, parietal lobe and liver eQTL data were downloaded from ScanDB. Cis-eQTLs were limited to those with P<1.0E-6 and trans-eQTLs with P<5.0E-8. Results for GTEx[73] Analysis V4 for 13 tissues were downloaded from the GTEx[73] Portal and then additionally filtered as described below [www. GTExportal.org: thyroid, leg skin (sun exposed), tibial nerve, aortic artery, tibial artery, skeletal muscle, esophagus mucosa, esophagus muscularis, lung, heart (left ventricle), stomach, whole blood, and subcutaneous adipose tissue [73]]. Splicing QTL (sQTL) results generated with sQTLseeker with false discovery rate P≤0.05 were retained. For all gene-level eQTLs, if at least 1 SNP passed the tissue-specific empirical threshold in GTEx[73], the best SNP for that eQTL was always retained. All gene-level eQTL SNPs with P<1.67E-11 were also retained, reflecting a global threshold correction of P=0.05/(30,000 genes X 1,000,000 tests).

Second, since public databases with eQTL data do not have information available on current smoking status, we also conducted an eQTL association analysis using expression results derived from fasting peripheral whole blood collected. Total RNA was isolated from frozen PAXgene blood tubes (PreAnalytiX, Hombrechtikon, Switzerland) and amplified using the WT-Ovation Pico RNA Amplification System (NuGEN, San Carlos, CA) according to the manufacturers' standard operating procedures. The obtained cDNA was hybridized to the Human Exon 1.0 ST Array (Affymetrix, Inc., Santa Clara, CA). The raw data were quantile-normalized, log2 transformed, followed by summarization using Robust Multi-array Average [327]and further adjusted for technical covariates, including the first principal component of the expression data, batch effect, and the all-probeset-mean residual. Study specific covariates in the association model included blood cell counts and cohort membership.
We evaluated all transcripts +/- 1MB around each novel variant in the Framingham Heart Study while accounting for current smoking status, using the following four approaches similar to those used in our primary analyses of our traits:

**Model 1 (adjusted main effect of eQTL):** Expression ~***SNP*** + SMK + age + age-squared + sex + study specific covariates

**Model 2 (main effect of eQTL stratified by smoking status)**: Expression ~ ***SNP*** + age + age-squared + sex + study specific covariates

**Model 3 (Interaction effect of eQTL):** Expression ~ SNP + SMK + ***SNP*SMK*** + age + age-squared + sex + study specific covariates

**Model 4 (Joint effect of eQTL):** Expression ~ ***SNP*** + SMK + ***SNP*SMK*** + age + age-squared + sex + study specific covariates

Significance level was evaluated by FDR < 5% per eQTL analysis and across all loci identified for that model in the primary meta-analysis.

**eQTL Results by Trait**

Only significant cis-eQTLS in high LD with our novel lead SNPs ($r^2$>0.9, calculated in the CEU+YRI+CHB+JPT 1000 Genomes reference panel), or proxy SNPs, were retained for consideration.

For BMI, three of our seven novel SNPs across six loci that had at least one variant in high LD ($r^2$>0.9) with the tag SNP that is significantly (**Online Methods**) associated with expression of a gene transcript in the cerebellum and prefrontal cortex, or blood cell types, including *EPHA3*, *TTC14*, and *INADL*. Notably, our lead SNP, rs2481665, is a significant cis-eQTL for *INADL*, in prefrontal cortex tissue, and for *INADL* and *LITD1* in whole blood after adjusting for SMK (false discovery rate, FDR<5%). For the joint main + interaction effect eQTL analysis, we identified one significant eQTL for a BMI associated variant (rs12902602) for three gene transcripts (*PSMA4, CHRNA5*, and *CTSH*).

For WCadjBMI, five of our 12 novel SNPs were in high LD with a cis-eQTL for gene transcripts in the cerebellum, temporal cortex, prefrontal cortex, lymphoblastoid cells, liver, lung, lymph, omental adipose, subcutaneous adipose, Primary PHA-stimulated T cells, skin, and blood cell tissues in publicly available databases. In our cis-eQTL analyses adjusting for SMK, four of our nine novel lead SNPs were significant cis-eQTLs for 14 gene transcripts in 12 genes. Additionally, for the joint main + interaction effect eQTL analysis, we identified that two variants that were associated with the expression of *SEPT2, FARP2, PASK*, and *HDLBP* (rs6743226) and *KIF1B* (rs17396340).

For WHRadjBMI, three of out six novel SNPs were in high LD with a nearby cis-eQTL for gene transcripts in subcutaneous adipose tissue and blood cell types. We identified five novel WHRadjBMI variants near significant cis-eQTLs for 49 gene transcripts after adjusting for SMK, the most significant of which was between our tag SNP rs1049281 and *MSH5*. Additionally, for the joint main and interaction effect eQTL analysis, we identified two novel WHRadjBMI variants (rs1049281, rs1856293) were associated with 19 gene transcripts.

Across all of our three obesity-related traits, the majority of significant cis-eQTLs from public databases are found in blood cell lines (63% of unique SNP-transcript associations) (**Supplementary Table 16**). However, as in previous eQTL analyses of obesity-associated variants, we identify cis-eQTLs in brain and adipose tissue. Further analyses are needed to determine if these tissue-specific eQTLs remain significant after accounting for SMK, but our de-novo analysis in whole blood samples from the Framingham Heart Study using models to account for SMK indicate that gene expression may underlie our association signals in some instances and smoking exposure may play a role in influencing these associations (**Supplementary Tables 16-18**).

**SUPPLEMENTARY NOTE 4. Full list of acknowledgments, including study-specific acknowledgements.**

Writing and analysis of this study for AEJ were supported by the American Heart Association (13POST16500011) and NIH (2T32HL007055-36, 5K99HL130580-02), for TOK by the Danish Council for Independent Research (DFF – 1333-00124 and Sapere Aude program grant DFF – 1331-00730B), and analyses performed by JP were funded through NIH (T32GM074905).

**Study Specific Acknowledgements**

**AE:** Genotyping was funded by Cavadis B.V. Sander W. van der Laan is funded through grants from the Netherlands CardioVascular Research Initiative ("GENIUS", CVON2011-19), and the Interuniversity Cardiology Institute of the Netherlands (ICIN, 09.001). Sander W. van der Laan and Saskia Haitjema are funded through a grant from FP7 EU project CVgenes@target (HEALTH-F2-2013-601456). Marten A. Siemelink acknowledges funding by the European Union (BiomarCaRE, grant number: HEALTH-2011-278913) and Technology Foundation STW (Stichting voor de Technische Wetenschappen, Project 11679). Claudia Tersteeg, Krista den Ouden, Mirjam B. Smeets, and Loes B. Collé are graciously acknowledged for their work on the DNA extraction. Astrid E.M.W. Willems, Evelyn Velema, Kristy M. J. Vons, Sara Bregman, Timo R. ten Brinke, Sara van Laar, Sander M. van de Weg, Louise M. Catanzariti, Arjan H. Schoneveld, Petra H. Homoed-van der Kraak, Aryan Vink, and Joyce E.P. Vrijenhoek are graciously acknowledged for their past and continuing work on the Athero-Express Biobank Study. We would also like to thank all the (former) employees involved in the Athero-Express Biobank Study of the Departments of Surgery of the St. Antonius Hospital Nieuwegein and University Medical Center Utrecht for their continuing work. Jessica van Setten is graciously acknowledged for her help in the quality assurance and quality control of the genotype data. Lastly, we would like to thank all participants of the Athero-Express Biobank Study. MAS is funded by the European Union (BiomarCaRE, grant number: HEALTH-2011-278913), and the technology foundation "Stichting voor de Technische Wetenschappen" through the Danone partnership program (Project 11679). **SWvdL/SH**: SWvdL/SH: SWvdL is funded through grants from the Netherlands CardioVascular Research Initiative ("GENIUS", CVON2011-19) and the Interuniversity Cardiology Institute of the Netherlands (ICIN, 09.001). SWvdL and SH are both funded through the FP7 EU project CVgenes@target (HEALTH-F2-2013-601456).

**AGES:** The Reykjavik Study cohort originally comprised a random sample of 30,795 men and women born in 1907-1935 and living in Reykjavik in 1967. A total of 19,381 attended, resulting in 71% recruitment rate. Between 2002 and 2006, the AGES-Reykjavik study re-examined 5,764 survivors of the original cohort who had participated before in the Reykjavik Study. [Harris, T. B. et al. (2007). American Journal of Epidemiology, 165(9), 1076-1087. doi:10.1093/aje/kwk115]. This study has been funded by NIH contract N01-AG-1-2100, the NIA Intramural Research Program, Hjartavernd (the Icelandic Heart Association), and the Althingi (the Icelandic Parliament). The study is approved by the Icelandic National Bioethics Committee, VSN: 00-063. The researchers are indebted to the participants for their willingness to participate in the study.

**ARIC:** The Atherosclerosis Risk in Communities Study is carried out as a collaborative study supported by National Heart, Lung, and Blood Institute contracts N01-HC-55015, N01-HC-55016, N01-C-55018, N01-HC-55019, N01-HC-55020, N01-HC-55021, N01-HC-55022, R01HL087641, R01HL59367 and R01HL086694; National Human Genome Research Institute contract U01HG004402; and National Institutes of Health contract HHSN268200625226C. Infrastructure was partly supported by Grant Number UL1RR025005, a component of the National Institutes of Health and NIH Roadmap for Medical Research. The project described was supported by Grant Number UL1 RR 025005 from the National Center for Research Resources (NCRR), a component of the National Institutes of Health (NIH) and NIH Roadmap for Medical Research, and its contents are solely the responsibility of the authors and do not necessarily represent the official view of NCRR or NIH. The authors thank the staff and participants of the ARIC Study for their important contributions.

**AUSTWIN:** We acknowledge the contributions of many staff in the Genetic Epidemiology Unit, Queensland Institute of Medical Research, in interviewing study participants, sample processing and DNA extraction, and data management. Funding for aspects of this work was provided by the Australian National Health and Medical Research Council (241944, 339462, 389927, 389875, 389891, 389892,

389938, 442915, 442981, 496739, 552485, 552498), the EU 5th Framework Programme GenomeEUTwin Project (QLG2-CT-2002-01254), and the U.S. National Institutes of Health (AA07535, AA10248, AA11998, AA13320, AA13321, AA13326, AA14041, AA17688, DA12854, MH66206 ). A portion of the genotyping on which this study was based was carried out at the Center for Inherited Disease Research, Baltimore (CIDR) (Illumina 370K scans on 4300 individuals), through an access award to our late colleague Dr. Richard Todd. Parts of the statistical analyses were carried out on the Genetic Cluster Computer, which is financially supported by the Netherlands Scientific Organization (NWO 480-05-003). R.P.S.M. was, and G.W.M. is, supported by National Health and Medical Research Council (NHMRC) Fellowship Schemes.

**BHS:** The Busselton Health Study acknowledges the generous support for the 1994/5 follow-up study from Healthway, Western Australia and the numerous Busselton community volunteers who assisted with data collection and the study participants from the Shire of Busselton. The Busselton Health Study is supported by Department of Health and the Office of Science of the Government of Western Australia.

**BioMe (MSSM):** The Mount Sinai BioMe Biobank is supported by The Andrea and Charles Bronfman Philanthropies.

**BLSA:** The BLSA was supported by the Intramural Research Program of the NIH, National Institute on Aging.

**B58C:** We acknowledge use of phenotype and genotype data from the British 1958 Birth Cohort DNA collection, funded by the Medical Research Council grant G0000934 and the Wellcome Trust grant 068545/Z/02. Genotyping for the B58C-WTCCC subset was funded by the Wellcome Trust grant 076113/B/04/Z. The B58C-T1DGC genotyping utilized resources provided by the Type 1 Diabetes Genetics Consortium, a collaborative clinical study sponsored by the National Institute of Diabetes and Digestive and Kidney Diseases (NIDDK), National Institute of Allergy and Infectious Diseases (NIAID), National Human Genome Research Institute (NHGRI), National Institute of Child Health and Human Development (NICHD), and Juvenile Diabetes Research Foundation International (JDRF) and supported by U01 DK062418. B58C-T1DGC GWAS data were deposited by the Diabetes and Inflammation Laboratory, Cambridge Institute for Medical Research (CIMR), University of Cambridge, which is funded by Juvenile Diabetes Research Foundation International, the Wellcome Trust and the National Institute for Health Research Cambridge Biomedical Research Centre; the CIMR is in receipt of a Wellcome Trust Strategic Award (079895). The B58C-GABRIEL genotyping was supported by a contract from the European Commission Framework Programme 6 (018996) and grants from the French Ministry of Research.

**CHARGE consortium**: We acknowledge the Cohorts for Heart and Aging Research in Genomic Epidemiology (CHARGE) Consortium for encouraging CHARGE studies to participate in this effort and for the contributions of CHARGE members to the analyses conducted for this research.

**CHS:** This CHS research was supported by NHLBI contracts HHSN268201200036C, HHSN268200800007C, N01HC55222, N01HC85079, N01HC85080, N01HC85081, N01HC85082, N01HC85083, N01HC85086; and NHLBI grants U01HL080295, R01HL087652, R01HL105756, R01HL103612, R01HL130114, and R01HL120393 with additional contribution from the National Institute of Neurological Disorders and Stroke (NINDS). Additional support was provided through R01AG023629 from the National Institute on Aging (NIA). A full list of principal CHS investigators and institutions can be found at CHS-NHLBI.org. The provision of genotyping data was supported in part by the National Center for Advancing Translational Sciences, CTSI grant UL1TR000124, and the National Institute of Diabetes and Digestive and Kidney Disease Diabetes Research Center (DRC) grant DK063491 to the Southern California Diabetes

Endocrinology Research Center. Subjects for the present study were selected from CHS participants who had donated DNA samples for storage and provided informed consent for participation in DNA studies of cardiovascular-disease-related traits. The content of this work is solely the responsibility of the authors and does not necessarily represent the official views of the National Institutes of Health.

**CLHNS:** The Cebu Longitudinal Health and Nutrition Survey (CLHNS) was supported by National Institutes of Health grants DK078150, TW05596, HL085144 and TW008288 and pilot funds from RR20649, ES10126, and DK56350. We thank the Office of Population Studies Foundation research and data collection teams and the study participants who generously provided their time for this study.

**COLAUS:** The CoLaus study was and is supported by research grants from GlaxoSmithKline, the Faculty of Biology and Medicine of Lausanne, and the Swiss National Science Foundation (grants 33CSCO-122661, 33CS30-139468 and 33CS30-148401). The authors thank Vincent Mooser and Dawn Waterworth, Co-PIs of the CoLaus study. Special thanks to Yolande Barreau, Mathieu Firmann, Vladimir Mayor, Anne-Lise Bastian, Binasa Ramic, Martine Moranville, Martine Baumer, Marcy Sagette, Jeanne Ecoffey and Sylvie Mermoud for data collection. SBe is supported by the Swiss National Science Foundation (grant 3100AO-116323/1) and the Swiss Institute of Bioinformatics. ZK received financial support from the Leenaards Foundation, the Swiss Institute of Bioinformatics and the Swiss National Science Foundation (31003A-143914, 51RTP0_151019).

**CROATIA-Korcula:** We would like to acknowledge the contributions of the recruitment team in Korcula, the administrative teams in Croatia and Edinburgh and the people of Korcula. The SNP genotyping for the CROATIA-Korcula cohort was performed in Helmholtz Zentrum München, Neuherberg, Germany. The study was financed by the Medical Research Council UK, the Ministry of Science, Education and Sport in the Republic of Croatia (grant number 108-1080315-0302) and the Croatian Science Foundation (grant 8875).

**CROATIA-Vis:** We would like to acknowledge the staff of several institutions in Croatia that supported the field work, including but not limited to The University of Split and Zagreb Medical Schools, Institute for Anthropological Research in Zagreb and Croatian Institute for Public Health. The SNP genotyping for the CROATIA-Vis cohort was performed in the core genotyping laboratory of the Wellcome Trust Clinical Research Facility at the Western General Hospital, Edinburgh, Scotland. This study was supported through grants from the Medical Research Council UK, the Ministry of Science, Education and Sport of the Republic of Croatia (number 108-1080315-0302) and the European Union framework program 6 EUROSPAN project (contract no. LSHG-CT-2006-018947).

**DESIR:** This study was supported in part by grants from SFD ("Société Francophone du 358 Diabète"), CPER ("Contrat de Projets État-Région"), and ANR ("Agence Nationale de la 359 Recherche"). The D.E.S.I.R. study has been supported by INSERM contracts with CNAMTS, Lilly, Novartis Pharma and Sanofi-Aventis; by INSERM (Réseaux en Santé Publique, Interactions entre les déterminants de la santé), Cohortes Santé TGIR, the Association Diabète Risque Vasculaire, the Fédération Française de Cardiologie, La Fondation de France, ALFEDIAM, ONIVINS, Ardix Medical, Bayer Diagnostics, Becton Dickinson, Cardionics, Merck Santé, Novo Nordisk, Pierre Fabre, Roche, Topcon.

**DR's EXTRA:** The DR.s EXTRA Study was supported by grants to R. Rauramaa by the Ministry of Education and Culture of Finland (627;2004-2011), Academy of Finland (102318; 123885), Kuopio University Hospital , Finnish Diabetes Association, Finnish Heart Association, Päivikki and Sakari Sohlberg Foundation and by

grants from European Commission FP6 Integrated Project (EXGENESIS); LSHM-CT-2004-005272, City of Kuopio and Social Insurance Institution of Finland (4/26/2010).

**EGCUT:** EGCUT received support from EU FP7 grant Biobanking and Biomolecular Resources Research Infrastructure (BBMRI)-LPC 313010, targeted financing from Estonian Government IUT20-60, IUT24-6, Estonian Research Roadmap through the Estonian Ministry of Education and Research (3.2.0304.11-0312), Center of Excellence in Genomics (EXCEGEN), Development Fund from the University of Tartu (SP1GVARENG). This work was also supported by the US National Institute of Health [R01DK075787].

**Ely:** We are grateful to all the volunteers and to the staff of St. Mary's Street Surgery, Ely and the study team. The Ely Study was funded by the MRC (MC_U106179471) and Diabetes UK. Genotyping in the Ely and Fenland studies was supported in part by an MRC-GlaxoSmithKline pilot programme grant (G0701863).

**EPIC:** The EPIC Norfolk diabetes case cohort study is nested within the EPIC Norfolk Study, which is supported by programme grants from the Medical Research Council, and Cancer Research UK and with additional support from the European Union, Stroke Association, British Heart Foundation, Research into Ageing, Department of Health, The Wellcome Trust and the Food Standards Agency. Genotyping was in part supported by the MRC-GSK pilot programme grant. We acknowledge the contribution of the staff and participants of the EPIC-Norfolk Study.

**EPIC-Norfolk:** The EPIC Norfolk Study is funded by program grants from the Medical Research Council UK and Cancer Research UK, and by additional support from the European Union, Stroke Association, British Heart Foundation, Department of Health, Food Standards Agency, and the Wellcome Trust.

**ERF:** The ERF study as a part of EUROSPAN (European Special Populations Research Network) was supported by European Commission FP6 STRP grant number 018947 (LSHG-CT-2006-01947) and also received funding from the European Community's Seventh Framework Programme (FP7/2007-2013)/grant agreement HEALTH-F4-2007-201413 by the European Commission under the programme "Quality of Life and Management of the Living Resources" of 5th Framework Programme (no. QLG2-CT-2002-01254). High-throughput analysis of the ERF data was supported by joint grant from Netherlands Organization for Scientific Research and the Russian Foundation for Basic Research (NWO-RFBR 047.017.043). Exome sequencing analysis in ERF was supported by the ZonMw grant (project 91111025). We are grateful to all study participants and their relatives, general practitioners and neurologists for their contributions and to P. Veraart for her help in genealogy, J. Vergeer for the supervision of the laboratory work and P. Snijders for his help in data collection. Najaf Amin is supported by the Netherlands Brain Foundation (project number F2013(1)-28)

**FamHS:** The Family Heart Study was supported by grant R01-DK-089256 from NIDDK and grant R01HL117078 from NHLBI.

**Fenland:** The Fenland Study is funded by the Wellcome Trust and the Medical Research Council (MC_U106179471). We are grateful to all the volunteers for their time and help, and to the General Practitioners and practice staff for assistance with recruitment. We thank the Fenland Study Investigators, Fenland Study Co-ordination team and the Epidemiology Field, Data and Laboratory teams.

**FramHS**: This research was conducted in part using data and resources from the Framingham Heart Study of the National Heart Lung and Blood Institute of the National Institutes of Health and Boston University

School of Medicine. The analyses reflect intellectual input and resource development from the Framingham Heart Study investigators participating in the SNP Health Association Resource (SHARe) project. This work was partially supported by the National Heart, Lung and Blood Institute's Framingham Heart Study (Contract No. N01-HC-25195 and Contract No. HHSN268201500001I) and its contract with Affymetrix, Inc for genotyping services (Contract No. N02-HL-6-4278). A portion of this research utilized the Linux Cluster for Genetic Analysis (LinGA-II) funded by the Robert Dawson Evans Endowment of the Department of Medicine at Boston University School of Medicine and Boston Medical Center. This research was partially supported by grant R01-DK089256 from the National Institute of Diabetes and Digestive and Kidney Diseases (MPIs: I.B. Borecki, L.A. Cupples, K. North).

**FUSION:** Support for FUSION was provided by NIH grants R01-DK062370 (to M.B.), R01-DK072193 (to K.L.M.), and intramural project number 1Z01-HG000024 (to F.S.C.). Genome-wide genotyping was conducted by the Johns Hopkins University Genetic Resources Core Facility SNP Center at the Center for Inherited Disease Research (CIDR), with support from CIDR NIH contract no. N01-HG-65403.

**Gendian:** The support of the physicians, the patients, and the staff of the Diabetes Zentrum Mergentheim (Head: Prof. Dr. Thomas Haak), the diabetes outpatient clinic Dr Nusser - Dr Kreisel, the dialysis centers KfH Amberg, KfH Bayreuth, KfH Deggendorf, KfH Donauwörth, KfH Freising, KfH Freyung, KfH Fürth, KfH Hof, KfH Ingolstadt, KfH Kelheim, KfH München Elsenheimerstraße, KfH München-Schwabing, KfH Neumarkt, KfH Neusäß, KfH Oberschleißheim, KfH Passau, KfH Plauen, KfH Regensburg Günzstraße, KfH Regensburg Caritas-Krankenhaus, KfH Straubing, KfH Sulzbach-Rosenberg, KfH Weiden, Dialysezentrum Augsburg Dr. Kirschner, Dialysezentrum Bad Alexandersbad, KfH Bamberg, Dialysezentrum Emmering, Dialysezentrum Klinikum Landshut, Dialysezentrum Landshut, Dialysezentrum Pfarrkirchen, Dialysezentrum Schwandorf, Dr. Angela Götz, the medical doctoral student Johanna Christ and the Study Nurse Ingrid Lugauer. The expert technical assistance of Claudia Strohmeier is gratefully acknowledged. Phenotyping was funded by the Dr. Robert Pfleger-Stiftung (Dr Carsten A. Böger), the MSD Stipend Diabetes (Dr Carsten A. Böger) and the University Hospital of Regensburg (intramural grant ReForM A to Dr. A. Götz, ReForM C to Dr. Carsten Böger). Genome-wide genotyping was funded by the KfH Stiftung Präventivmedizin e.V. (Dr. Carsten A. Böger, Dr. Jens Brüning), the Else Kröner-Fresenius-Stiftung (2012_A147 to Dr Carsten A. Böger and Dr Iris M. Heid) and the University Hospital Regensburg (Dr Carsten A. Böger). Data analysis was funded by the Else Kröner-Fresenius Stiftung (Dr. Iris M. Heid and Dr. Carsten A. Böger: 2012_A147; Dr. Carsten A. Böger and Dr. Bernhard K. Krämer: P48/08//A11/08).

**Generation Scotland (GS):** We would like to acknowledge the contributions of the families who took part in the Generation Scotland: Scottish Family Health Study, the general practitioners and Scottish School of Primary Care for their help in recruiting them, and the whole Generation Scotland team, which includes academic researchers, IT staff, laboratory technicians, statisticians and research managers. Generation Scotland received core support from the Chief Scientist Office of the Scottish Government Health Directorates [CZD/16/6] and the Scottish Funding Council [HR03006]. Genotyping of the GS:SFHS samples was carried out by the Genetics Core Laboratory at the Wellcome Trust Clinical Research Facility, Edinburgh, Scotland and was funded by the Medical Research Council UK and the Wellcome Trust (Wellcome Trust Strategic Award "STratifying Resilience and Depression Longitudinally" (STRADL) Reference 104036/Z/14/Z). MIM is a Wellcome Trust Senior Investigator, and an NIHR Senior Investigator.

**GENOA:** The Genetic Epidemiology Network of Arteriopathy (GENOA) study is supported by the National Institutes of Health, grant numbers HL054457, HL087660, and HL119443 from National Heart, Lung, Blood Institute. We thank Eric Boerwinkle, PhD from the Human Genetics Center and Institute of Molecular Medicine and Division of Epidemiology, University of Texas Health Science Center, Houston, Texas, USA

and Julie Cunningham, PhD from the Department of Health Sciences Research, Mayo Clinic College of Medicine, Rochester, MN, USA for their help with genotyping.

**GIANT consortium**: This work was performed under the auspices of the Genetic Investigation of ANthropometric Traits (GIANT) consortium.

**GLACIER:** The GLACIER study was funded by project grants to Paul W. Franks from Novo Nordisk, the Swedish Heart-Lung Foundation, the Swedish Diabetes Association, Påhlssons Foundation, the Swedish Research Council, Umeå University Career Development Award, and The Heart Foundation of Northern Sweden. Frida Renström was supported by a post-doctoral stipend from the Swedish Heart-Lung Foundation. Shafqat Ahmad was supported by post-doctoral fellowship from Swedish Heart-Lung Foundation and Nutricia Research Foundation. The investigators thank the staff at the Wellcome Trust Sanger Institute for technical assistance with genotyping (WT098051 to the list of Wellcome Trust funded grants). The investigators are indebted to the study participants who dedicated their time and samples to these studies and the staff at the Umeå Medical Biobank and VIP for biomedical data collection and preparation.

**GOOD:** Financial support was received from the Swedish Research Council, the Swedish Foundation for Strategic Research, the ALF/LUA research grant in Gothenburg, the Lundberg Foundation, the Torsten and Ragnar Söderberg's Foundation, the Västra Götaland Foundation, the Göteborg Medical Society, the Novo Nordisk foundation, and the European Commission grant HEALTH-F2-2008-201865-GEFOS. We would like to acknowledge Maria Nethander at the genomics core facility at University of Gothenburg for statistical analyses.

**GOYA:** This study was conducted as part of the activities of the Gene-diet Interactions in Obesity project (GENDINOB, www.gendinob.dk) and the MRC centre for Causal Analyses in Translational Epidemiology (MRC CAiTE). We thank all the participants of the study. TSA was also funded by the GENDINOB project and acknowledges the same.

**GxE:** Our chief acknowledgement is to the participants in these studies for their willingness to contribute. We also thank Nurses Orgen Brown and Diedre Thomas for assistance with recruitment as well as past and present Laboratory technologists and drivers at TMRU for their invaluable technical assistance. This work was supported by NIH Grants R01HL53353 and R01DK075787.

**Health2006:** The Health2006 study was financially supported by grants from the Velux Foundation; the Danish Medical Research Council, Danish Agency for Science, Technology and Innovation; the Aase and Ejner Danielsens foundation; ALK-Abello´ A/S (Hørsholm, Denmark), Timber Merchant Vilhelm Bangs Foundation, MEKOS Laboratories (Denmark) and Research Centre for Prevention and Health, the Capital Region of Denmark. The Health2006 was approved by the Ethical Committee of Copenhagen (KA-20060011) and the Danish Data Protection Agency.

**HERITAGE:** The HERITAGE Family Study is supported by the National Heart, Lung, and Blood Institute Grants HL-45670 and HL118305.

**HRS:** The HRS is supported by the National Institute on Aging (NIA U01AG009740). The genotyping was funded separately by the National Institute on Aging (RC2 AG036495, RC4 AG039029). Our genotyping was conducted by the NIH Center for Inherited Disease Research (CIDR) at Johns Hopkins University.

Genotyping quality control and final preparation of the data were performed by the Genetics Coordinating Center at the University of Washington.

**HUNT2:** The Nord-Trøndelag Health Study (The HUNT Study) is a collaboration between HUNT Research Centre (Faculty of Medicine, Norwegian University of Science and Technology NTNU), Nord-Trøndelag County Council, Central Norway Health Authority, and the Norwegian Institute of Public Health.

**HYPERGENES:** The study was supported by the European Union (FP7-HEALTH-F4-2007-201550-HYPERGENES, HEALTH-2011.2.4.2-2-EU-MASCARA, HEALTH-F7-305507 HOMAGE and the European Research Council Advanced Researcher Grant-2011-294713-EPLORE); InterOmics project (PB05 MIUR-CNR Italian Flagship Project); The Fonds voor Wetenschappelijk Onderzoek Vlaanderen, Ministry of the Flemish Community, Brussels, Belgium (G.0881.13 and G.088013).

**IMPROVE:** IMPROVE was supported by the European Commission (Contract number: QLG1-CT-2002-00896), the Swedish Heart-Lung Foundation, the Swedish Research Council (projects 8691 and 0593), the Knut and Alice Wallenberg Foundation, the Foundation for Strategic Research, the Stockholm County Council (project 592229), the Strategic Cardiovascular and Diabetes Programmes of Karolinska Institutet and Stockholm County Council, the European Union Framework Programme 7 (FP7/2007-2013) for the Innovative Medicine Initiative under grant agreement n° IMI/115006 (the SUMMIT consortium), the Academy of Finland (Grant #110413), the British Heart Foundation (RG2008/08, RG2008/014) and the Italian Ministry of Health (Ricerca Corrente).

**InCHIANTI:** The InCHIANTI study baseline (1998-2000) was supported as a "targeted project" (ICS110.1/RF97.71) by the Italian Ministry of Health and in part by the U.S. National Institute on Aging (Contracts: 263 MD 9164 and 263 MD 821336).

**Inter99:** The Inter99 study was funded by: Danish Research Councils; The Health Foundation; The Danish Centre for Evaluation and Health Technology Assessment; Copenhagen County; Danish Heart Foundation; Ministry of Health and Prevention; Danish Pharmaceutical Association; Augustinus Foundation; Novo Nordisk; Velux Foundation; Becket Foundation and Ib Henriksens Foundation.

**KORA:** The KORA study was initiated and financed by the Helmholtz Zentrum München – German Research Center for Environmental Health, which is funded by the German Federal Ministry of Education and Research (BMBF) and by the State of Bavaria. Furthermore, KORA research was supported within the Munich Center of Health Sciences (MC-Health), Ludwig-Maximilians-Universität, as part of LMUinnovativ. IMH was supported by German Federal Ministry of Education and Research (BMBF, 01ER1206 and 01ER1507). [PMID: 16032513] [PMID: 16032514]

**Lifelines:** The Lifelines Cohort Study, and generation and management of GWAS genotype data for the Lifelines Cohort Study is supported by the Netherlands Organization of Scientific Research NWO (grant 175.010.2007.006), the Economic Structure Enhancing Fund (FES) of the Dutch government, the Ministry of Economic Affairs, the Ministry of Education, Culture and Science, the Ministry for Health, Welfare and Sports, the Northern Netherlands Collaboration of Provinces (SNN), the Province of Groningen, University Medical Center Groningen, the University of Groningen, Dutch Kidney Foundation and Dutch Diabetes Research Foundation.

**LOLIPOP:** The LOLIPOP study is supported by the National Institute for Health Research (NIHR) Comprehensive Biomedical Research Centre Imperial College Healthcare NHS Trust, the British Heart

Foundation (SP/04/002), the Medical Research Council (G0601966,G0700931), the Wellcome Trust (084723/Z/08/Z) the NIHR (RP-PG-0407-10371), European Union FP7 (EpiMigrant, 279143) and Action on Hearing Loss (G51). The work was carried out in part at the NIHR/Wellcome Trust Imperial Clinical Research Facility. We thank the participants and research staff who made the study possible.

**LURIC:** We thank the LURIC study team who were either temporarily or permanently involved in patient recruitment as well as sample and data handling, in addition to the laboratory staff at the Ludwigshafen General Hospital and the Universities of Freiburg and Ulm, Germany. This work was supported by the 7th Framework Program (integrated project AtheroRemo, grant agreement number 201668 and RiskyCAD, grant agreement number 305739) of the European Union and by the INTERREG IV Oberrhein Program (Project A28, Genetic mechanisms of cardiovascular diseases) with support from the European Regional Development Fund (ERDF) and the Wissenschaftsoffensive TMO.

**MEC:** The Multiethnic Cohort study (MEC) characterization of epidemiological architecture is funded through the NHGRI PAGE program (U01HG004802 and its NHGRI ARRA supplement). The MEC study is funded through the National Cancer Institute (R37CA54281, R01 CA63, P01CA33619, U01CA136792, and U01CA98758).

**MESA:** MESA and the MESA SHARe project are conducted and supported by the National Heart, Lung, and Blood Institute (NHLBI) in collaboration with MESA investigators. Support for MESA is provided by contracts N01-HC-95159, N01-HC-95160, N01-HC-95161, N01-HC-95162, N01-HC-95163, N01-HC-95164, N01-HC-95165, N01-HC-95166, N01-HC-95167, N01-HC-95168, N01-HC-95169, UL1-TR-001079, and UL1-TR-000040. Funding for MESA SHARe genotyping was provided by NHLBI Contract N02-HL-6-4278. The provision of genotyping data was supported in part by the National Center for Advancing Translational Sciences, CTSI grant UL1TR000124, and the National Institute of Diabetes and Digestive and Kidney Disease Diabetes Research Center (DRC) grant DK063491 to the Southern California Diabetes Endocrinology Research Center. The authors thank the MESA participants, as well as the Coordinating Centers, investigators, and study staff for their valuable contributions. A full list of participating MESA investigators and institutions can be found at http://www.mesa-nhlbi.org.

**METSIM:** The METSIM study was funded by the Academy of Finland (grants no. 77299 and 124243).

**MRC NSHD:** This work was funded by the Medical Research Council (MC_UU_12019/1), the British Heart Foundation (RG/10/12/28456) and the Wellcome Trust (088869/B/09/Z). We are very grateful to the members of this birth cohort for their continuing interest and participation in the study. We would like to acknowledge the Swallow group, UCL, who performed the DNA extractions (Rousseau, et al 2006). DOI: 10.1111/j.1469-1809.2006.00250.x.

**MrOS Sweden:** This work was supported by the Swedish Research Council, the Swedish Foundation for Strategic Research, The ALF/LUA research grant in Gothenburg, the Lundberg Foundation, the Torsten and Ragnar Söderberg's Foundation, Magnus Bergvall Foundation, Åke Wiberg Foundation, Tore Nilson Foundation and The Swedish Society for Medical Research.

**NHS:** The study was supported by grants from the National Heart, Lung, and Blood Institute (HL071981, HL034594, HL126024), the National Institute of Diabetes and Digestive and Kidney Diseases (DK091718, DK100383, DK078616), the Boston Obesity Nutrition Research Center (DK46200), and United States – Israel Binational Science Foundation Grant2011036.

**NTR:** This study was funded by the Netherlands Organization for Scientific Research (NWO) and The Netherlands Organisation for Health Research and Development (ZonMW) grants 904-61-090, 985-10-002, 904-61-193,480-04-004, 400-05-717, Addiction-31160008, Middelgroot-911-09-032, Spinozapremie 56-464-14192, Center for Medical Systems Biology (CSMB, NWO Genomics), NBIC/BioAssist/RK(2008.024), Biobanking and Biomolecular Resources Research Infrastructure (BBMRI – NL, 184.021.007). VU University's Institute for Health and Care Research (EMGO+ ) and Neuroscience Campus Amsterdam (NCA); the European Science Foundation (ESF, EU/QLRT-2001-01254), the European Community's Seventh Framework Program (FP7/2007-2013), ENGAGE (HEALTH-F4-2007-201413); the European Research Council (ERC Advanced, 230374, Starting grant 284 167), Rutgers University Cell and DNA Repository (NIMH U24 MH068457-06), the Avera Institute, Sioux Falls, South Dakota (USA) and the National Institutes of Health (NIH, R01D0042157-01A, MH081802, Grand Opportunity grant 1RC2 MH089951). Part of the genotyping and analyses were funded by the Genetic Association Information Network (GAIN) of the Foundation for the National Institutes of Health.

**NFBC1966/Oxford Univ:** The Northern Finland Birth Cohort (NFBC) Research program, received financial support from Academy of Finland (1114194, 24300796), NHLBI grant 5R01HL087679 through the STAMPEED program (1RL1MH083268-01), ENGAGE project and grant agreement HEALTH-F4-2007-201413, the Medical Research Council (grant G0500539, centre grant G0600705, PrevMetSyn), and the Wellcome Trust (project grant GR069224), UK. The program is currently being funded by the H2020-633595 DynaHEALTH action and Academy of Finland EGEA-project. University of Oxford, UK, was funded by the British Heart Foundation (grant code SP/13/2/30111), the European Commission (ENGAGE: HEALTH-F4-2007-201413), Medical Research Council (G0601261), and the Wellcome Trust (090532, 098381). M-R.J. received funding from the European Union's Horizon 2020 research and innovation programme [under grant agreement No 633595].

**ORCADES:** ORCADES was supported by the Chief Scientist Office of the Scottish Government, the Royal Society, the MRC Human Genetics Unit, Arthritis Research UK and the European Union framework program 6 EUROSPAN project (contract no. LSHG-CT-2006-018947). DNA extractions were performed at the Wellcome Trust Clinical Research Facility in Edinburgh. We would like to acknowledge the invaluable contributions of Lorraine Anderson and the research nurses in Orkney, the administrative team in Edinburgh and the people of Orkney.

**PIVUS:** PIVUS was supported by Knut and Alice Wallenberg Foundation (Wallenberg Academy Fellow), European Research Council (ERC Starting Grant), Swedish Diabetes Foundation (2013-024), Swedish Research Council (2012-1397, 2012-1727, and 2012-2215), Marianne and Marcus Wallenberg Foundation, County Council of Dalarna, Dalarna University, and Swedish Heart-Lung Foundation (20120197). The computations were performed on resources provided by SNIC through Uppsala Multidisciplinary Center for Advanced Computational Science (UPPMAX) under Project b2011036. Genotyping was funded by the Wellcome Trust under awards WT064890 and WT086596. Analysis of genetic data was funded by the Wellcome Trust under awards WT098017 and WT090532. We thank the SNP&SEQ Technology Platform in Uppsala (www.genotyping.se) for excellent genotyping. Andrew P Morris is a Wellcome Trust Senior Fellow in Basic Biomedical Science under award WT098017.

**PREVEND:** The PREVEND genetics is supported by the Dutch Kidney Foundation (Grant E033), the EU project grant GENECURE (FP-6 LSHM CT 2006 037697), the National Institutes of Health (grant 2R01LM010098), The Netherlands organization for health research and development (NWO-Groot grant 175.010.2007.006, NWO VENI grant 916.761.70, ZonMw grant 90.700.441), and the Dutch Inter University

Cardiology Institute Netherlands (ICIN). N. Verweij is supported by the Netherlands Heart Foundation (grant NHS2010B280).

**PROSPER:** The PROSPER study was supported by an investigator initiated grant obtained from Bristol-Myers Squibb. Prof. Dr. J. W. Jukema is an Established Clinical Investigator of the Netherlands Heart Foundation (grant 2001 D 032). Support for genotyping was provided by the seventh framework program of the European commission (grant 223004) and by the Netherlands Genomics Initiative (Netherlands Consortium for Healthy Aging grant 050-060-810).

**QFS:** The Quebec Family Study (QFS) was funded by multiple grants from the Medical Research Council of Canada and the Canadian Institutes for Health Research. This work was supported by a team grant from the Canadian Institutes for Health Research (FRCN-CCT-83028).

**RS1:** We thank the Genetic Laboratory of the Department of Internal Medicine of the Erasmus MC and specifically Pascal Arp, Mila Jhamai, Marijn Verkerk, and Carolina Medina-Gomez for their help in creating the GWAS database and the creation and analysis of imputed data. The dedication, commitment, and contribution of inhabitants, general practitioners, and pharmacists of the Ommoord district to the Rotterdam Study are gratefully acknowledged. We also thank the patients participating in the Erasmus Stroke Study.

**RS2:** The Rotterdam study is supported by the Erasmus MC and Erasmus University Rotterdam; the Netherlands Organisation for Scientific Research; the Netherlands Organisation for Health Research and Development (Zorg onderzoek Nederland Medische Wetenschappen); the Research Institute for Diseases in the Elderly; the Netherlands Genomics Initiative; the Ministry of Education, Culture and Science; the Ministry of Health, Welfare and Sports; the European Commission (Directorate-General XII); and the Municipality of Rotterdam.

**RS3:** None of the funders had any role in design and conduct of the study; collection, management, analysis, and interpretation of the data; and preparation, review, or approval of this article.

**SardiNIA:** We thank the many individuals who generously participated in this study. This work was supported by Contract NO1-AG-1-2109 from the National Institute of Aging, and in part by a grant from the Italian Ministry of Economy and Finance to the CNR for the Project "FaReBio di Qualità" to F Cucca. The efforts of GR Abecasis were supported in part by contract 263-MA-410953 from the NIA to the University of Michigan and by research grant HG002651 and HL084729 from the NIH.

**SCARFSHEEP:** The work was supported by the European Commission (LSHM-CT- 2007- 037273), the Swedish Heart-Lung Foundation, the Swedish Research Council (8691, 09533), the Knut and Alice Wallenberg Foundation, the Foundation for Strategic Research, the Torsten and Ragnar Söderberg Foundation, the Strategic Cardiovascular Programme of Karolinska Institutet and the Stockholm County Council and the Stockholm County Council (560183); the Magnus Bergvall Foundation, Stiftelsen för Gamla Tjänarinnor, and the Tore Nilsson and Fredrik och Ingrid Thurings Foundations.

**SHIP:** SHIP is part of the Community Medicine Research net of the University of Greifswald, Germany, which is funded by the Federal Ministry of Education and Research (grants no. 01ZZ9603, 01ZZ0103, and 01ZZ0403), the Ministry of Cultural Affairs as well as the Social Ministry of the Federal State of Mecklenburg-West Pomerania, and the network 'Greifswald Approach to Individualized Medicine (GANI_MED)' funded by the Federal Ministry of Education and Research (grant 03IS2061A). Genome-wide

data have been supported by the Federal Ministry of Education and Research (grant no. 03ZIK012) and a joint grant from Siemens Healthcare, Erlangen, Germany and the Federal State of Mecklenburg- West Pomerania. The University of Greifswald is a member of the Caché Campus program of the InterSystems GmbH.

**SORBS:** This work was supported by grants from the German Research Council (SFB- 1052 "Obesity mechanisms", SPP 1629 TO 718/2-1), from the German Diabetes Association and from the DHFD (Diabetes Hilfs- und Forschungsfonds Deutschland). Inga Prokopenko was funded in part through the European Community's Seventh Framework Programme (FP7/2007-2013), ENGAGE project, grant agreement HEALTH-F4-2007-201413.

**SPT:** Our chief acknowledgement is to the participants in these studies for their willingness to contribute. We also thank Nurses Orgen Brown and Diedre Thomas for assistance with recruitment as well as past and present Laboratory technologists and drivers at TMRU for their invaluable technical assistance. This work was supported by NIH Grants R01HL53353 and R01DK075787.

**THISEAS:** Recruitment for The Hellenic study of Interactions between SNPs and Eating in Atherosclerosis Susceptibility (THISEAS) study was partially funded by a research grant (PENED 2003) from the Greek General Secretary of Research and Technology. We thank all the dieticians and clinicians for their contribution to the project and the Genotyping Facility at the Wellcome Trust Sanger Institute for SNP typing. Analysis was partly supported by BHF grant (Deloukas) RG/14/5/30893 and the Barts Cardiovascular Biomedical Research Unit which is supported and funded by the National Institute for Health Research.

**TRAILS:** TRAILS (TRacking Adolescents' Individual Lives Survey) is a collaborative project involving various departments of the University Medical Center and University of Groningen, the Erasmus University Medical Center Rotterdam, the University of Utrecht, the Radboud Medical Center Nijmegen, and the Parnassia Bavo group, all in the Netherlands. TRAILS has been financially supported by grants from the Netherlands Organization for Scientific Research NWO (Medical Research Council program grant GB-MW 940-38-011; ZonMW Brainpower grant 100-001-004; ZonMw Risk Behavior and Dependence grant 60-60600-97-118; ZonMw Culture and Health grant 261-98-710; Social Sciences Council medium-sized investment grants GB-MaGW 480-01-006 and GB-MaGW 480-07-001; Social Sciences Council project grants GB-MaGW 452-04-314 and GB-MaGW 452-06-004; NWO large-sized investment grant 175.010.2003.005; NWO Longitudinal Survey and Panel Funding 481-08-013); the Dutch Ministry of Justice (WODC), the European Science Foundation (EuroSTRESS project FP-006), Biobanking and Biomolecular Resources Research Infrastructure BBMRI-NL (CP 32), the participating universities, and Accare Center for Child and Adolescent Psychiatry. We are grateful to all adolescents, their parents and teachers who participated in this research and to everyone who worked on this project and made it possible. Statistical analyses were carried out on the Genetic Cluster Computer (http://www.geneticcluster.org), which is financially supported by the Netherlands Scientific Organization (NWO 480-05-003) along with a supplement from the Dutch Brain Foundation.

**TwinsUK:** The study was funded by the Wellcome Trust; European Community's Seventh Framework Programme (FP7/2007-2013). The study also receives support from the National Institute for Health Research (NIHR) BioResource Clinical Research Facility and Biomedical Research Centre based at Guy's and St Thomas' NHS Foundation Trust and King's College London. SNP Genotyping was performed by The Wellcome Trust Sanger Institute and National Eye Institute via NIH/CIDR.

**UKBB:** Dr. Tyrrell is supported by a Diabetes Research and Wellness Foundation Fellowship. Prof. Frayling is supported by the European Research Council grant: 323195:SZ-245 50371-GLUCOSEGENES-FP7-IDEAS-ERC.

**WGHS:** The WGHS is supported by HL043851 and HL080467 from the National Heart, Lung, and Blood Institute, and CA047988 and UM1CA182913 from the National Cancer Institute (NCI), the Donald W. Reynolds Foundation and the Fondation Leducq, with collaborative scientific support and funding for genotyping provided by Amgen.

**WHI:** Funding support for the "Epidemiology of putative genetic variants: The Women's Health Initiative" study is provided through the NHGRI PAGE program (U01HG007376, HG004790, and its NHGRI ARRA supplement). The WHI acknowledgment statement that you have is out of date. The statement should be The WHI program is funded by the National Heart, Lung, and Blood Institute, National Institutes of Health, U.S. Department of Health and Human Services through contracts HHSN268201100046C, HHSN268201100001C, HHSN268201100002C, HHSN268201100003C, HHSN268201100004C, and HHSN271201100004C.

**Whitehall:** Dr. Kumari's and Professor Kivimaki's time on this manuscript was partially supported by the National Heart Lung and Blood Institute (NHLBI: HL36310). The Whitehall-II study has been supported by grants from the Medical Research Council (MRC); British Heart Foundation; Health and Safety Executive; Department of Health; National Institute on Aging (AG13196), US, NIH; Agency for Health Care Policy Research (HS06516); and the John D and Catherine T MacArthur Foundation Research Networks on Successful Midlife Development and Socioeconomic Status and Health.

**YFS:** The Young Finns Study has been financially supported by the Academy of Finland: grants 286284, 134309 (Eye), 126925, 121584, 124282, 129378 (Salve), 117787 (Gendi), and 41071 (Skidi); the Social Insurance Institution of Finland; Competitive State Research Financing of the Expert Responsibility area of Kuopio, Tampere and Turku University Hospitals (grant X51001); Juho Vainio Foundation; Paavo Nurmi Foundation; Finnish Foundation for Cardiovascular Research ; Finnish Cultural Foundation; Tampere Tuberculosis Foundation; Emil Aaltonen Foundation; Yrjö Jahnsson Foundation; Signe and Ane Gyllenberg Foundation; and Diabetes Research Foundation of Finnish Diabetes Association. The expert technical assistance in the statistical analyses by Irina Lisinen is gratefully acknowledged.

## SUPPLEMENTARY REFERENCES

1. Locke, A.E. *et al.* Genetic studies of body mass index yield new insights for obesity biology. *Nature* **518**, 197-206 (2015).
2. Shungin, D. *et al.* New genetic loci link adipose and insulin biology to body fat distribution. *Nature* **518**, 187-96 (2015).
3. Pena, J.C., Duhalt, R., Navarrette, R. & Garcia Zozaya, J.L. [Periodic hemodialysis in the treatment of chronic renal insufficiency]. *Gac Med Mex* **98**, 150-67 (1968).
4. Shin, K., Wang, Q. & Margolis, B. PATJ regulates directional migration of mammalian epithelial cells. *EMBO Rep* **8**, 158-64 (2007).
5. Comuzzie, A.G. *et al.* Novel genetic loci identified for the pathophysiology of childhood obesity in the Hispanic population. *PLoS One* **7**, e51954 (2012).
6. Kathiresan, S. *et al.* Six new loci associated with blood low-density lipoprotein cholesterol, high-density lipoprotein cholesterol or triglycerides in humans. *Nat Genet* **40**, 189-97 (2008).

7. Zhang, Z. *et al.* Association of genetic loci with blood lipids in the Chinese population. *PLoS One* **6**, e27305 (2011).
8. Waterworth, D.M. *et al.* Genetic variants influencing circulating lipid levels and risk of coronary artery disease. *Arterioscler Thromb Vasc Biol* **30**, 2264-76 (2010).
9. Murphy, M.P. & LeVine, H., 3rd. Alzheimer's disease and the amyloid-beta peptide. *J Alzheimers Dis* **19**, 311-23 (2010).
10. Lee, Y.H. *et al.* Amyloid precursor protein expression is upregulated in adipocytes in obesity. *Obesity (Silver Spring)* **16**, 1493-500 (2008).
11. Puig, K.L., Floden, A.M., Adhikari, R., Golovko, M.Y. & Combs, C.K. Amyloid precursor protein and proinflammatory changes are regulated in brain and adipose tissue in a murine model of high fat diet-induced obesity. *PLoS One* **7**, e30378 (2012).
12. Martin-Campos, J.M. *et al.* Identification of a novel mutation in the ANGPTL3 gene in two families diagnosed of familial hypobetalipoproteinemia without APOB mutation. *Clin Chim Acta* **413**, 552-5 (2012).
13. Kathiresan, S. *et al.* Common variants at 30 loci contribute to polygenic dyslipidemia. *Nat Genet* **41**, 56-65 (2009).
14. Willer, C.J. *et al.* Newly identified loci that influence lipid concentrations and risk of coronary artery disease. *Nat Genet* **40**, 161-9 (2008).
15. Wong, R.C. *et al.* L1TD1 is a marker for undifferentiated human embryonic stem cells. *PLoS One* **6**, e19355 (2011).
16. Santos, M.C. *et al.* Embryonic Stem Cell-Related Protein L1TD1 Is Required for Cell Viability, Neurosphere Formation, and Chemoresistance in Medulloblastoma. *Stem Cells Dev* **24**, 2700-8 (2015).
17. Zhu, Y., Kakinuma, N., Wang, Y. & Kiyama, R. Kank proteins: a new family of ankyrin-repeat domain-containing proteins. *Biochim Biophys Acta* **1780**, 128-33 (2008).
18. Boyle, A.P. *et al.* Annotation of functional variation in personal genomes using RegulomeDB. *Genome Res* **22**, 1790-7 (2012).
19. de la Rocha, A.M., Sampron, N., Alonso, M.M. & Matheu, A. Role of SOX family of transcription factors in central nervous system tumors. *Am J Cancer Res* **4**, 312-24 (2014).
20. Hempel, A. *et al.* Deletions and de novo mutations of SOX11 are associated with a neurodevelopmental disorder with features of Coffin-Siris syndrome. *J Med Genet* **53**, 152-62 (2016).
21. Ward, L.D. & Kellis, M. HaploReg: a resource for exploring chromatin states, conservation, and regulatory motif alterations within sets of genetically linked variants. *Nucleic Acids Res* **40**, D930-4 (2012).
22. Zhuang, G. *et al.* Effects of cancer-associated EPHA3 mutations on lung cancer. *J Natl Cancer Inst* **104**, 1182-97 (2012).
23. Wood, L.D. *et al.* Somatic mutations of GUCY2F, EPHA3, and NTRK3 in human cancers. *Hum Mutat* **27**, 1060-1 (2006).
24. Lisabeth, E.M., Fernandez, C. & Pasquale, E.B. Cancer somatic mutations disrupt functions of the EphA3 receptor tyrosine kinase through multiple mechanisms. *Biochemistry* **51**, 1464-75 (2012).
25. Lee, D.J. *et al.* Multiple tumor-suppressor genes on chromosome 3p contribute to head and neck squamous cell carcinoma tumorigenesis. *Cancer Biol Ther* **10**, 689-93 (2010).
26. Lahtela, J. *et al.* A high-content cellular senescence screen identifies candidate tumor suppressors, including EPHA3. *Cell Cycle* **12**, 625-34 (2013).
27. Davis, S.D. *et al.* Clinical features of childhood primary ciliary dyskinesia by genotype and ultrastructural phenotype. *Am J Respir Crit Care Med* **191**, 316-24 (2015).

28. Oldenburg, A.R., Delbarre, E., Thiede, B., Vigouroux, C. & Collas, P. Deregulation of Fragile X-related protein 1 by the lipodystrophic lamin A p.R482W mutation elicits a myogenic gene expression program in preadipocytes. *Hum Mol Genet* **23**, 1151-62 (2014).
29. Sparkes, R., Patton, D. & Bernier, F. Cardiac features of a novel autosomal recessive dilated cardiomyopathic syndrome due to defective importation of mitochondrial protein. *Cardiol Young* **17**, 215-7 (2007).
30. Ojala, T. *et al.* New mutation of mitochondrial DNAJC19 causing dilated and noncompaction cardiomyopathy, anemia, ataxia, and male genital anomalies. *Pediatr Res* **72**, 432-7 (2012).
31. Davey, K.M. *et al.* Mutation of DNAJC19, a human homologue of yeast inner mitochondrial membrane co-chaperones, causes DCMA syndrome, a novel autosomal recessive Barth syndrome-like condition. *J Med Genet* **43**, 385-93 (2006).
32. Richter-Dennerlein, R. *et al.* DNAJC19, a mitochondrial cochaperone associated with cardiomyopathy, forms a complex with prohibitins to regulate cardiolipin remodeling. *Cell Metab* **20**, 158-71 (2014).
33. Perez-Lorenzo, R. *et al.* A tumor suppressor function for the lipid phosphatase INPP4B in melanocytic neoplasms. *J Invest Dermatol* **134**, 1359-68 (2014).
34. Fedele, C.G. *et al.* Inositol polyphosphate 4-phosphatase II regulates PI3K/Akt signaling and is lost in human basal-like breast cancers. *Proc Natl Acad Sci U S A* **107**, 22231-6 (2010).
35. Lopez, S.M. *et al.* Determinants of the tumor suppressor INPP4B protein and lipid phosphatase activities. *Biochem Biophys Res Commun* **440**, 277-82 (2013).
36. Mian, M.F., Pek, E.A., Mossman, K.L., Stampfli, M.R. & Ashkar, A.A. Exposure to cigarette smoke suppresses IL-15 generation and its regulatory NK cell functions in poly I:C-augmented human PBMCs. *Mol Immunol* **46**, 3108-16 (2009).
37. Liu, Y.Z. *et al.* Genome-wide association analyses suggested a novel mechanism for smoking behavior regulated by IL15. *Mol Psychiatry* **14**, 668-80 (2009).
38. Thorgeirsson, T.E. *et al.* Sequence variants at CHRNB3-CHRNA6 and CYP2A6 affect smoking behavior. *Nat Genet* **42**, 448-53 (2010).
39. Saccone, N.L. *et al.* Multiple independent loci at chromosome 15q25.1 affect smoking quantity: a meta-analysis and comparison with lung cancer and COPD. *PLoS Genet* **6**(2010).
40. Hancock, D.B. *et al.* Genome-wide meta-analysis reveals common splice site acceptor variant in CHRNA4 associated with nicotine dependence. *Transl Psychiatry* **5**, e651 (2015).
41. Thorgeirsson, T.E. *et al.* A variant associated with nicotine dependence, lung cancer and peripheral arterial disease. *Nature* **452**, 638-42 (2008).
42. Chen, L.S. *et al.* CHRNA5 risk variant predicts delayed smoking cessation and earlier lung cancer diagnosis--a meta-analysis. *J Natl Cancer Inst* **107**(2015).
43. Hallden, S. *et al.* Gene variance in the nicotinic receptor cluster (CHRNA5-CHRNA3-CHRNB4) predicts death from cardiopulmonary disease and cancer in smokers. *J Intern Med* (2015).
44. Tyrrell, J. *et al.* Genetic variation in the 15q25 nicotinic acetylcholine receptor gene cluster (CHRNA5-CHRNA3-CHRNB4) interacts with maternal self-reported smoking status during pregnancy to influence birth weight. *Hum Mol Genet* **21**, 5344-58 (2012).
45. Winslow, U.C., Rode, L. & Nordestgaard, B.G. High tobacco consumption lowers body weight: a Mendelian randomization study of the Copenhagen General Population Study. *Int J Epidemiol* **44**, 540-50 (2015).
46. Taylor, A.E. *et al.* Stratification by smoking status reveals an association of CHRNA5-A3-B4 genotype with body mass index in never smokers. *PLoS Genet* **10**, e1004799 (2014).
47. Morel, C. *et al.* Nicotine consumption is regulated by a human polymorphism in dopamine neurons. *Mol Psychiatry* **19**, 930-6 (2014).

48. Antolin-Fontes, B., Ables, J.L., Gorlich, A. & Ibanez-Tallon, I. The habenulo-interpeduncular pathway in nicotine aversion and withdrawal. *Neuropharmacology* **96**, 213-22 (2015).
49. van Setten, J. *et al.* Genome-wide association study of coronary and aortic calcification implicates risk loci for coronary artery disease and myocardial infarction. *Atherosclerosis* **228**, 400-5 (2013).
50. Schunkert, H. *et al.* Large-scale association analysis identifies 13 new susceptibility loci for coronary artery disease. *Nat Genet* **43**, 333-8 (2011).
51. Reilly, M.P. *et al.* Identification of ADAMTS7 as a novel locus for coronary atherosclerosis and association of ABO with myocardial infarction in the presence of coronary atherosclerosis: two genome-wide association studies. *Lancet* **377**, 383-92 (2011).
52. Dichgans, M. *et al.* Shared genetic susceptibility to ischemic stroke and coronary artery disease: a genome-wide analysis of common variants. *Stroke* **45**, 24-36 (2014).
53. Wang, Z.C. *et al.* Genetic polymorphism of the kinesin-like protein KIF1B gene and the risk of hepatocellular carcinoma. *PLoS One* **8**, e62571 (2013).
54. Sukhatme, V.P. & Chan, B. Glycolytic cancer cells lacking 6-phosphogluconate dehydrogenase metabolize glucose to induce senescence. *FEBS Lett* **586**, 2389-95 (2012).
55. Ahn, J. *et al.* Identification of the avian RBP7 gene as a new adipose-specific gene and RBP7 promoter-driven GFP expression in adipose tissue of transgenic quail. *PLoS One* **10**, e0124768 (2015).
56. Borcherding, D.C. *et al.* Dopamine receptors in human adipocytes: expression and functions. *PLoS One* **6**, e25537 (2011).
57. Gieger, C. *et al.* New gene functions in megakaryopoiesis and platelet formation. *Nature* **480**, 201-8 (2011).
58. Nogueira, E. *et al.* SOK1 translocates from the Golgi to the nucleus upon chemical anoxia and induces apoptotic cell death. *J Biol Chem* **283**, 16248-58 (2008).
59. Davids, M.S. *et al.* STK25 is a candidate gene for pseudopseudohypoparathyroidism. *Genomics* **77**, 2-4 (2001).
60. Nerstedt, A. *et al.* Serine/threonine protein kinase 25 (STK25): a novel negative regulator of lipid and glucose metabolism in rodent and human skeletal muscle. *Diabetologia* **55**, 1797-807 (2012).
61. Cansby, E. *et al.* Increased expression of STK25 leads to impaired glucose utilization and insulin sensitivity in mice challenged with a high-fat diet. *FASEB J* **27**, 3660-71 (2013).
62. Semplici, F. *et al.* Human mutation within Per-Arnt-Sim (PAS) domain-containing protein kinase (PASK) causes basal insulin hypersecretion. *J Biol Chem* **286**, 44005-14 (2011).
63. Grose, J.H. & Rutter, J. The role of PAS kinase in PASsing the glucose signal. *Sensors (Basel)* **10**, 5668-82 (2010).
64. da Silva Xavier, G., Rutter, J. & Rutter, G.A. Involvement of Per-Arnt-Sim (PAS) kinase in the stimulation of preproinsulin and pancreatic duodenum homeobox 1 gene expression by glucose. *Proc Natl Acad Sci U S A* **101**, 8319-24 (2004).
65. Su, Z., Cox, A., Shen, Y., Stylianou, I.M. & Paigen, B. Farp2 and Stk25 are candidate genes for the HDL cholesterol locus on mouse chromosome 1. *Arterioscler Thromb Vasc Biol* **29**, 107-13 (2009).
66. Li, B.Z. *et al.* [Abnormal expression of bcl-10 protein in extranodal marginal zone B cell lymphoma of mucosa-associated lymphoid tissue lymphoma type]. *Zhonghua Bing Li Xue Za Zhi* **36**, 819-24 (2007).
67. Namekata, K. *et al.* Dock3 regulates BDNF-TrkB signaling for neurite outgrowth by forming a ternary complex with Elmo and RhoG. *Genes Cells* **17**, 688-97 (2012).
68. Liu, H., Tang, X. & Gong, L. Mesencephalic astrocyte-derived neurotrophic factor and cerebral dopamine neurotrophic factor: New endoplasmic reticulum stress response proteins. *Eur J Pharmacol* **750**, 118-22 (2015).

69. Padilla, J. *et al.* Transcriptome-wide RNA sequencing analysis of rat skeletal muscle feed arteries. II. Impact of exercise training in obesity. *J Appl Physiol (1985)* **116**, 1033-47 (2014).
70. Wood, A.R. *et al.* Defining the role of common variation in the genomic and biological architecture of adult human height. *Nat Genet* **46**, 1173-86 (2014).
71. Lango Allen, H. *et al.* Hundreds of variants clustered in genomic loci and biological pathways affect human height. *Nature* **467**, 832-8 (2010).
72. Song, F. *et al.* Identification of a melanoma susceptibility locus and somatic mutation in TET2. *Carcinogenesis* **35**, 2097-101 (2014).
73. Consortium, G.T. The Genotype-Tissue Expression (GTEx) project. *Nat Genet* **45**, 580-5 (2013).
74. Sabater-Lleal, M. *et al.* Multiethnic meta-analysis of genome-wide association studies in >100 000 subjects identifies 23 fibrinogen-associated Loci but no strong evidence of a causal association between circulating fibrinogen and cardiovascular disease. *Circulation* **128**, 1310-24 (2013).
75. Kettunen, J. *et al.* Genome-wide association study identifies multiple loci influencing human serum metabolite levels. *Nat Genet* **44**, 269-76 (2012).
76. Inouye, M. *et al.* Novel Loci for metabolic networks and multi-tissue expression studies reveal genes for atherosclerosis. *PLoS Genet* **8**, e1002907 (2012).
77. Berndt, S.I. *et al.* Genome-wide meta-analysis identifies 11 new loci for anthropometric traits and provides insights into genetic architecture. *Nat Genet* **45**, 501-12 (2013).
78. Weedon, M.N. *et al.* Genome-wide association analysis identifies 20 loci that influence adult height. *Nat Genet* **40**, 575-83 (2008).
79. Soranzo, N. *et al.* Meta-analysis of genome-wide scans for human adult stature identifies novel Loci and associations with measures of skeletal frame size. *PLoS Genet* **5**, e1000445 (2009).
80. Lettre, G. *et al.* Identification of ten loci associated with height highlights new biological pathways in human growth. *Nat Genet* **40**, 584-91 (2008).
81. Gudbjartsson, D.F. *et al.* Many sequence variants affecting diversity of adult human height. *Nat Genet* **40**, 609-15 (2008).
82. Reiner, A.P. *et al.* Genome-wide association study of white blood cell count in 16,388 African Americans: the continental origins and genetic epidemiology network (COGENT). *PLoS Genet* **7**, e1002108 (2011).
83. Okada, Y. *et al.* Genetics of rheumatoid arthritis contributes to biology and drug discovery. *Nature* **506**, 376-81 (2014).
84. Raychaudhuri, S. *et al.* Common variants at CD40 and other loci confer risk of rheumatoid arthritis. *Nat Genet* **40**, 1216-23 (2008).
85. Albers, H.M. *et al.* Genetic variation in VTCN1 (B7-H4) is associated with course of disease in juvenile idiopathic arthritis. *Ann Rheum Dis* **73**, 1198-201 (2014).
86. Markula-Patjas, K.P. *et al.* High adiposity and serum leptin accompanied by altered bone turnover markers in severe juvenile idiopathic arthritis. *J Rheumatol* **41**, 2474-81 (2014).
87. Kim da, S., Lee, S.Y., Lee, J.H., Bae, Y.C. & Jung, J.S. MicroRNA-103a-3p controls proliferation and osteogenic differentiation of human adipose tissue-derived stromal cells. *Exp Mol Med* **47**, e172 (2015).
88. Sedlmeier, E.M. *et al.* Human placental transcriptome shows sexually dimorphic gene expression and responsiveness to maternal dietary n-3 long-chain polyunsaturated fatty acid intervention during pregnancy. *BMC Genomics* **15**, 941 (2014).
89. Gamazon, E.R. *et al.* SCAN: SNP and copy number annotation. *Bioinformatics* **26**, 259-62 (2010).
90. Li, M., Campbell, S. & McDermott, R. gamma-Glutamyltransferase, obesity, physical activity, and the metabolic syndrome in indigenous Australian adults. *Obesity (Silver Spring)* **17**, 809-13 (2009).

91. Iwasaki, T. *et al.* Hepatic fat content-independent association of the serum level of gamma-glutamyltransferase with visceral adiposity, but not subcutaneous adiposity. *Diabetes Res Clin Pract* **79**, e13-4 (2008).
92. Lee, D.S. *et al.* Gamma glutamyl transferase and metabolic syndrome, cardiovascular disease, and mortality risk: the Framingham Heart Study. *Arterioscler Thromb Vasc Biol* **27**, 127-33 (2007).
93. Atar, A.I. *et al.* Association between gamma-glutamyltransferase and coronary artery calcification. *Int J Cardiol* **167**, 1264-7 (2013).
94. Bradley, R.D. *et al.* Associations between gamma-glutamyltransferase (GGT) and biomarkers of atherosclerosis: the Multi-ethnic Study of Atherosclerosis (MESA). *Atherosclerosis* **233**, 387-93 (2014).
95. Zhu, C. *et al.* Association of serum gamma-glutamyltransferase with arterial stiffness in established coronary artery disease. *Angiology* **64**, 15-20 (2013).
96. Park, J.S. *et al.* Association between gamma-glutamyltransferase, adiponectin and arterial stiffness. *J Atheroscler Thromb* **19**, 90-7 (2012).
97. Volodarsky, M. *et al.* A deletion mutation in TMEM38B associated with autosomal recessive osteogenesis imperfecta. *Hum Mutat* **34**, 582-6 (2013).
98. Shaheen, R. *et al.* Study of autosomal recessive osteogenesis imperfecta in Arabia reveals a novel locus defined by TMEM38B mutation. *J Med Genet* **49**, 630-5 (2012).
99. Rubinato, E. *et al.* A novel deletion mutation involving TMEM38B in a patient with autosomal recessive osteogenesis imperfecta. *Gene* **545**, 290-2 (2014).
100. Perry, J.R. *et al.* Meta-analysis of genome-wide association data identifies two loci influencing age at menarche. *Nat Genet* **41**, 648-50 (2009).
101. Perry, J.R. *et al.* Parent-of-origin-specific allelic associations among 106 genomic loci for age at menarche. *Nature* **514**, 92-7 (2014).
102. Elks, C.E. *et al.* Thirty new loci for age at menarche identified by a meta-analysis of genome-wide association studies. *Nat Genet* **42**, 1077-85 (2010).
103. Fox, C.S. *et al.* Genome-wide association for abdominal subcutaneous and visceral adipose reveals a novel locus for visceral fat in women. *PLoS Genet* **8**, e1002695 (2012).
104. Melzer, D. *et al.* A genome-wide association study identifies protein quantitative trait loci (pQTLs). *PLoS Genet* **4**, e1000072 (2008).
105. Ismail, S., Schaffer, A.E., Rosti, R.O., Gleeson, J.G. & Zaki, M.S. Novel mutation in the fukutin gene in an Egyptian family with Fukuyama congenital muscular dystrophy and microcephaly. *Gene* **539**, 279-82 (2014).
106. Costa, C. *et al.* A Portuguese case of Fukuyama congenital muscular dystrophy caused by a multi-exonic duplication in the fukutin gene. *Neuromuscul Disord* **23**, 557-61 (2013).
107. Welch, H.C. *et al.* P-Rex1, a PtdIns(3,4,5)P3- and Gbetagamma-regulated guanine-nucleotide exchange factor for Rac. *Cell* **108**, 809-21 (2002).
108. Kimura, S., Sato, K., Banno, Y., Nagase, T. & Ueda, H. The importance of interaction with membrane lipids through the pleckstrin homology domain of the guanine nucleotide exchange factor for rho family small guanosine triphosphatase, FLJ00018. *Biol Pharm Bull* **36**, 1204-7 (2013).
109. Damoulakis, G. *et al.* P-Rex1 directly activates RhoG to regulate GPCR-driven Rac signalling and actin polarity in neutrophils. *J Cell Sci* **127**, 2589-600 (2014).
110. Xue, R. *et al.* Clonal analyses and gene profiling identify genetic biomarkers of the thermogenic potential of human brown and white preadipocytes. *Nat Med* **21**, 760-8 (2015).
111. Kuhn, R.M., Haussler, D. & Kent, W.J. The UCSC genome browser and associated tools. *Brief Bioinform* **14**, 144-61 (2013).
112. Mick, E. *et al.* Genome-wide association study of proneness to anger. *PLoS One* **9**, e87257 (2014).

113. Micu, I. *et al.* NMDA receptors mediate calcium accumulation in myelin during chemical ischaemia. *Nature* **439**, 988-92 (2006).
114. Zhong, H.J. *et al.* Functional polymorphisms of the glutamate receptor N-methyl D-aspartate 2A gene are associated with heroin addiction. *Genet Mol Res* **13**, 8714-21 (2014).
115. Turner, S.J. *et al.* GRIN2A: an aptly named gene for speech dysfunction. *Neurology* **84**, 586-93 (2015).
116. Liu, R. *et al.* Correlation of functional GRIN2A gene promoter polymorphisms with schizophrenia and serum D-serine levels. *Gene* **568**, 25-30 (2015).
117. Leuba, G. *et al.* Pathological reorganization of NMDA receptors subunits and postsynaptic protein PSD-95 distribution in Alzheimer's disease. *Curr Alzheimer Res* **11**, 86-96 (2014).
118. Lemke, J.R. *et al.* Mutations in GRIN2A cause idiopathic focal epilepsy with rolandic spikes. *Nat Genet* **45**, 1067-72 (2013).
119. DeVries, S.P. & Patel, A.D. Two patients with a GRIN2A mutation and childhood-onset epilepsy. *Pediatr Neurol* **49**, 482-5 (2013).
120. Carvill, G.L. *et al.* GRIN2A mutations cause epilepsy-aphasia spectrum disorders. *Nat Genet* **45**, 1073-6 (2013).
121. Aouizerat, B.E. *et al.* GWAS for discovery and replication of genetic loci associated with sudden cardiac arrest in patients with coronary artery disease. *BMC Cardiovasc Disord* **11**, 29 (2011).
122. McGue, M. *et al.* A genome-wide association study of behavioral disinhibition. *Behav Genet* **43**, 363-73 (2013).
123. Greliche, N. *et al.* A genome-wide search for common SNP x SNP interactions on the risk of venous thrombosis. *BMC Med Genet* **14**, 36 (2013).
124. Volkmer, E. & Karnitz, L.M. Human homologs of Schizosaccharomyces pombe rad1, hus1, and rad9 form a DNA damage-responsive protein complex. *J Biol Chem* **274**, 567-70 (1999).
125. Marathi, U.K. *et al.* RAD1, a human structural homolog of the Schizosaccharomyces pombe RAD1 cell cycle checkpoint gene. *Genomics* **54**, 344-7 (1998).
126. Chaudhuri, S.P. *et al.* Activation of S phase checkpoint by cigarette smoke extract in Schizosaccharomyces pombe. *Yeast* **22**, 1223-38 (2005).
127. Suhre, K. *et al.* A genome-wide association study of metabolic traits in human urine. *Nat Genet* **43**, 565-9 (2011).
128. Seppala, I. *et al.* Genome-wide association study on dimethylarginines reveals novel AGXT2 variants associated with heart rate variability but not with overall mortality. *Eur Heart J* **35**, 524-31 (2014).
129. Rueedi, R. *et al.* Genome-wide association study of metabolic traits reveals novel gene-metabolite-disease links. *PLoS Genet* **10**, e1004132 (2014).
130. Nicholson, G. *et al.* A genome-wide metabolic QTL analysis in Europeans implicates two loci shaped by recent positive selection. *PLoS Genet* **7**, e1002270 (2011).
131. Arnett, D.K. *et al.* Genome-wide association study identifies single-nucleotide polymorphism in KCNB1 associated with left ventricular mass in humans: the HyperGEN Study. *BMC Med Genet* **10**, 43 (2009).
132. Warnatz, H.J. *et al.* The BTB and CNC homology 1 (BACH1) target genes are involved in the oxidative stress response and in control of the cell cycle. *J Biol Chem* **286**, 23521-32 (2011).
133. Kim, J. *et al.* Functional genomic screen for modulators of ciliogenesis and cilium length. *Nature* **464**, 1048-51 (2010).
134. Jostins, L. *et al.* Host-microbe interactions have shaped the genetic architecture of inflammatory bowel disease. *Nature* **491**, 119-24 (2012).
135. Linden, R. *et al.* Physiology of the prion protein. *Physiol Rev* **88**, 673-728 (2008).

136. Vanik, D.L. & Surewicz, W.K. Disease-associated F198S mutation increases the propensity of the recombinant prion protein for conformational conversion to scrapie-like form. *J Biol Chem* **277**, 49065-70 (2002).
137. Steele, A.D., Emsley, J.G., Ozdinler, P.H., Lindquist, S. & Macklis, J.D. Prion protein (PrPc) positively regulates neural precursor proliferation during developmental and adult mammalian neurogenesis. *Proc Natl Acad Sci U S A* **103**, 3416-21 (2006).
138. Mouillet-Richard, S. *et al.* Signal transduction through prion protein. *Science* **289**, 1925-8 (2000).
139. Telling, G.C. *et al.* Evidence for the conformation of the pathologic isoform of the prion protein enciphering and propagating prion diversity. *Science* **274**, 2079-82 (1996).
140. Poulter, M. *et al.* Inherited prion disease with 144 base pair gene insertion. 1. Genealogical and molecular studies. *Brain* **115 ( Pt 3)**, 675-85 (1992).
141. Moore, R.C. *et al.* Huntington disease phenocopy is a familial prion disease. *Am J Hum Genet* **69**, 1385-8 (2001).
142. Mead, S. *et al.* A novel protective prion protein variant that colocalizes with kuru exposure. *N Engl J Med* **361**, 2056-65 (2009).
143. Haik, S. *et al.* Striking PrPsc heterogeneity in inherited prion diseases with the D178N mutation. *Ann Neurol* **56**, 909-10; author reply 910-1 (2004).
144. Collinge, J. *et al.* Diagnosis of Gerstmann-Straussler syndrome in familial dementia with prion protein gene analysis. *Lancet* **2**, 15-7 (1989).
145. Basset-Leobon, C. *et al.* Familial Creutzfeldt-Jakob disease with an R208H-129V haplotype and Kuru plaques. *Arch Neurol* **63**, 449-52 (2006).
146. Redecke, L. *et al.* Structural characterization of beta-sheeted oligomers formed on the pathway of oxidative prion protein aggregation in vitro. *J Struct Biol* **157**, 308-20 (2007).
147. United States. Public Health Service. Office of the Surgeon General. *How tobacco smoke causes disease : the biology and behavioral basis for smoking-attributable disease : a report of the Surgeon General*, xv, 704 p (U.S. Dept. of Health and Human Services, Public Health Service, Rockville, MD; Washington, DC, 2010).
148. Bernhard, D., Rossmann, A. & Wick, G. Metals in cigarette smoke. *IUBMB Life* **57**, 805-9 (2005).
149. Moore, R.C. *et al.* Ataxia in prion protein (PrP)-deficient mice is associated with upregulation of the novel PrP-like protein doppel. *J Mol Biol* **292**, 797-817 (1999).
150. Croes, E.A. *et al.* Polymorphisms in the prion protein gene and in the doppel gene increase susceptibility for Creutzfeldt-Jakob disease. *Eur J Hum Genet* **12**, 389-94 (2004).
151. Schroder, B. *et al.* Polymorphisms within the prion-like protein gene (Prnd) and their implications in human prion diseases, Alzheimer's disease and other neurological disorders. *Hum Genet* **109**, 319-25 (2001).
152. Savini, I., Rossi, A., Pierro, C., Avigliano, L. & Catani, M.V. SVCT1 and SVCT2: key proteins for vitamin C uptake. *Amino Acids* **34**, 347-55 (2008).
153. Catania, A.S., Barros, C.R. & Ferreira, S.R. [Vitamins and minerals with antioxidant properties and cardiometabolic risk: controversies and perspectives]. *Arq Bras Endocrinol Metabol* **53**, 550-9 (2009).
154. Babaev, V.R. *et al.* Combined vitamin C and vitamin E deficiency worsens early atherosclerosis in apolipoprotein E-deficient mice. *Arterioscler Thromb Vasc Biol* **30**, 1751-7 (2010).
155. Hediger, M.A. New view at C. *Nat Med* **8**, 445-6 (2002).
156. Uhl, G.R., Drgon, T., Li, C.Y., Johnson, C. & Liu, Q.R. Smoking and smoking cessation in disadvantaged women: assessing genetic contributions. *Drug Alcohol Depend* **104 Suppl 1**, S58-63 (2009).

157. Rose, J.E., Behm, F.M., Drgon, T., Johnson, C. & Uhl, G.R. Personalized smoking cessation: interactions between nicotine dose, dependence and quit-success genotype score. *Mol Med* **16**, 247-53 (2010).
158. Zhao, L., Bracken, M.B. & DeWan, A.T. Genome-wide association study of pre-eclampsia detects novel maternal single nucleotide polymorphisms and copy-number variants in subsets of the Hyperglycemia and Adverse Pregnancy Outcome (HAPO) study cohort. *Ann Hum Genet* **77**, 277-87 (2013).
159. Caporaso, N. *et al.* Genome-wide and candidate gene association study of cigarette smoking behaviors. *PLoS One* **4**, e4653 (2009).
160. Oldenborg, P.A. *et al.* Role of CD47 as a marker of self on red blood cells. *Science* **288**, 2051-4 (2000).
161. Lindberg, F.P. *et al.* Decreased resistance to bacterial infection and granulocyte defects in IAP-deficient mice. *Science* **274**, 795-8 (1996).
162. Finley, M.J., Clark, K.A., Alferiev, I.S., Levy, R.J. & Stachelek, S.J. Intracellular signaling mechanisms associated with CD47 modified surfaces. *Biomaterials* **34**, 8640-9 (2013).
163. Wiewiora, M., Piecuch, J., Sedek, L., Mazur, B. & Sosada, K. The effects of obesity on CD47 expression in erythrocytes. *Cytometry B Clin Cytom* (2015).
164. Maimaitiyiming, H., Norman, H., Zhou, Q. & Wang, S. CD47 deficiency protects mice from diet-induced obesity and improves whole body glucose tolerance and insulin sensitivity. *Sci Rep* **5**, 8846 (2015).
165. Mozzetta, C., Pontis, J. & Ait-Si-Ali, S. Functional Crosstalk Between Lysine Methyltransferases on Histone Substrates: The Case of G9A/GLP and Polycomb Repressive Complex 2. *Antioxid Redox Signal* **22**, 1365-81 (2015).
166. Balakrishnan, V.S. *et al.* Cytokine gene polymorphisms in hemodialysis patients: association with comorbidity, functionality, and serum albumin. *Kidney Int* **65**, 1449-60 (2004).
167. Simmonds, R.E. & Foxwell, B.M. Signalling, inflammation and arthritis: NF-kappaB and its relevance to arthritis and inflammation. *Rheumatology (Oxford)* **47**, 584-90 (2008).
168. Nanes, M.S. Tumor necrosis factor-alpha: molecular and cellular mechanisms in skeletal pathology. *Gene* **321**, 1-15 (2003).
169. Boyce, B.F., Schwarz, E.M. & Xing, L. Osteoclast precursors: cytokine-stimulated immunomodulators of inflammatory bone disease. *Curr Opin Rheumatol* **18**, 427-32 (2006).
170. Lu, X. *et al.* Identification of the homeobox protein Prx1 (MHox, Prrx-1) as a regulator of osterix expression and mediator of tumor necrosis factor alpha action in osteoblast differentiation. *J Bone Miner Res* **26**, 209-19 (2011).
171. Clark, I.A. How TNF was recognized as a key mechanism of disease. *Cytokine Growth Factor Rev* **18**, 335-43 (2007).
172. Popko, K. *et al.* Proinflammatory cytokines Il-6 and TNF-alpha and the development of inflammation in obese subjects. *Eur J Med Res* **15 Suppl 2**, 120-2 (2010).
173. Gomez-Uriz, A.M. *et al.* Epigenetic patterns of two gene promoters (TNF-alpha and PON) in stroke considering obesity condition and dietary intake. *J Physiol Biochem* **70**, 603-14 (2014).
174. Elahi, M.M., Gilmour, A., Matata, B.M. & Mastana, S.S. A variant of position -308 of the Tumour necrosis factor alpha gene promoter and the risk of coronary heart disease. *Heart Lung Circ* **17**, 14-8 (2008).
175. Panasevich, S. *et al.* Interaction between early maternal smoking and variants in TNF and GSTP1 in childhood wheezing. *Clin Exp Allergy* **40**, 458-67 (2010).
176. Soler Artigas, M. *et al.* Genome-wide association and large-scale follow up identifies 16 new loci influencing lung function. *Nat Genet* **43**, 1082-90 (2011).

177. Natori, Y., Ohkura, N., Nasui, M., Atsumi, G. & Kihara-Negishi, F. Acidic sialidase activity is aberrant in obese and diabetic mice. *Biol Pharm Bull* **36**, 1027-31 (2013).
178. Guo, H. *et al.* Variations in HSPA1B at 6p21.3 are associated with lung cancer risk and prognosis in Chinese populations. *Cancer Res* **71**, 7576-86 (2011).
179. Moreno-Navarrete, J.M. *et al.* Complement factor H is expressed in adipose tissue in association with insulin resistance. *Diabetes* **59**, 200-9 (2010).
180. Kraja, A.T. *et al.* Pleiotropic genes for metabolic syndrome and inflammation. *Mol Genet Metab* **112**, 317-38 (2014).
181. Dahlman, I. *et al.* Functional annotation of the human fat cell secretome. *Arch Physiol Biochem* **118**, 84-91 (2012).
182. Arason, G.J. *et al.* Smoking and a complement gene polymorphism interact in promoting cardiovascular disease morbidity and mortality. *Clin Exp Immunol* **149**, 132-8 (2007).
183. Sesti, G. *et al.* A functional variant of the dimethylarginine dimethylaminohydrolase-2 gene is associated with chronic kidney disease. *Atherosclerosis* **231**, 141-4 (2013).
184. Andreozzi, F. *et al.* A functional variant of the dimethylarginine dimethylaminohydrolase-2 gene is associated with insulin sensitivity. *PLoS One* **7**, e36224 (2012).
185. Kappelle, P.J., Lambert, G., Dahlback, B., Nielsen, L.B. & Dullaart, R.P. Relationship of plasma apolipoprotein M with proprotein convertase subtilisin-kexin type 9 levels in non-diabetic subjects. *Atherosclerosis* **214**, 492-4 (2011).
186. Burkart, K.M. *et al.* APOM and high-density lipoprotein cholesterol are associated with lung function and per cent emphysema. *Eur Respir J* **43**, 1003-17 (2014).
187. Hudson, B.I. *et al.* Serum levels of soluble receptor for advanced glycation end-products and metabolic syndrome: the Northern Manhattan Study. *Metabolism* **63**, 1125-30 (2014).
188. Jiao, L. *et al.* Evidence that serum levels of the soluble receptor for advanced glycation end products are inversely associated with pancreatic cancer risk: a prospective study. *Cancer Res* **71**, 3582-9 (2011).
189. Kankova, K., Stejskalova, A., Hertlova, M. & Znojil, V. Haplotype analysis of the RAGE gene: identification of a haplotype marker for diabetic nephropathy in type 2 diabetes mellitus. *Nephrol Dial Transplant* **20**, 1093-102 (2005).
190. Nawroth, P., Bierhaus, A., Marrero, M., Yamamoto, H. & Stern, D.M. Atherosclerosis and restenosis: is there a role for RAGE? *Curr Diab Rep* **5**, 11-6 (2005).
191. Gohda, T. *et al.* Increased serum endogenous secretory receptor for advanced glycation end-product (esRAGE) levels in type 2 diabetic patients with decreased renal function. *Diabetes Res Clin Pract* **81**, 196-201 (2008).
192. Norata, G.D. *et al.* Circulating soluble receptor for advanced glycation end products is inversely associated with body mass index and waist/hip ratio in the general population. *Nutr Metab Cardiovasc Dis* **19**, 129-34 (2009).
193. Fukui, M. *et al.* The serum concentration of allograft inflammatory factor-1 is correlated with metabolic parameters in healthy subjects. *Metabolism* **61**, 1021-5 (2012).
194. Rouskas, K. *et al.* Common variants in FTO, MC4R, TMEM18, PRL, AIF1, and PCSK1 show evidence of association with adult obesity in the Greek population. *Obesity (Silver Spring)* **20**, 389-95 (2012).
195. Wang, Y. *et al.* Predictive role of multilocus genetic polymorphisms in cardiovascular disease and inflammation-related genes on chronic kidney disease in Type 2 diabetes--an 8-year prospective cohort analysis of 1163 patients. *Nephrol Dial Transplant* **27**, 190-6 (2012).
196. Mak, J.C. *et al.* Polymorphisms in the IL-4, IL-4 receptor alpha chain, TNF-alpha, and lymphotoxin-alpha genes and risk of asthma in Hong Kong Chinese adults. *Int Arch Allergy Immunol* **144**, 114-22 (2007).

197. Stolk, L. *et al.* Loci at chromosomes 13, 19 and 20 influence age at natural menopause. *Nat Genet* **41**, 645-7 (2009).
198. Levy, D. *et al.* Genome-wide association identifies OBFC1 as a locus involved in human leukocyte telomere biology. *Proc Natl Acad Sci U S A* **107**, 9293-8 (2010).
199. Stanescu, H.C. *et al.* Risk HLA-DQA1 and PLA(2)R1 alleles in idiopathic membranous nephropathy. *N Engl J Med* **364**, 616-26 (2011).
200. Kim, Y.J. *et al.* A genome-wide association study identified new variants associated with the risk of chronic hepatitis B. *Hum Mol Genet* **22**, 4233-8 (2013).
201. Walt, A.J., Bouwman, D.L., Weaver, D.W. & Sachs, R.J. The impact of technology on the management of pancreatic pseudocyst. Fifth annual Samuel Jason Mixter Lecture. *Arch Surg* **125**, 759-63 (1990).
202. Wang, Q. *et al.* MicroRNA-377 is up-regulated and can lead to increased fibronectin production in diabetic nephropathy. *FASEB J* **22**, 4126-35 (2008).
203. McDonough, C.W. *et al.* A genome-wide association study for diabetic nephropathy genes in African Americans. *Kidney Int* **79**, 563-72 (2011).
204. Lan, X. *et al.* Identification of differentially expressed genes related to metabolic syndrome induced with high-fat diet in E3 rats. *Exp Biol Med (Maywood)* **240**, 235-41 (2015).
205. Liu, Y. *et al.* Serum methylation levels of TAC1. SEPT9 and EYA4 as diagnostic markers for early colorectal cancers: a pilot study. *Biomarkers* **18**, 399-405 (2013).
206. Kisiel, J.B., Garrity-Park, M.M., Taylor, W.R., Smyrk, T.C. & Ahlquist, D.A. Methylated eyes absent 4 (EYA4) gene promotor in non-neoplastic mucosa of ulcerative colitis patients with colorectal cancer: evidence for a field effect. *Inflamm Bowel Dis* **19**, 2079-83 (2013).
207. Lovf, M. *et al.* A novel transcript, VNN1-AB, as a biomarker for colorectal cancer. *Int J Cancer* **135**, 2077-84 (2014).
208. Huang, H. *et al.* Novel blood biomarkers of pancreatic cancer-associated diabetes mellitus identified by peripheral blood-based gene expression profiles. *Am J Gastroenterol* **105**, 1661-9 (2010).
209. Soubeyrand, S., Naing, T., Martinuk, A. & McPherson, R. ERK1/2 regulates hepatocyte Trib1 in response to mitochondrial dysfunction. *Biochim Biophys Acta* **1833**, 3405-14 (2013).
210. Zhou, L. *et al.* A genome wide association study identifies common variants associated with lipid levels in the Chinese population. *PLoS One* **8**, e82420 (2013).
211. Kim, Y.J. *et al.* Large-scale genome-wide association studies in East Asians identify new genetic loci influencing metabolic traits. *Nat Genet* **43**, 990-5 (2011).
212. Kraja, A.T. *et al.* A bivariate genome-wide approach to metabolic syndrome: STAMPEED consortium. *Diabetes* **60**, 1329-39 (2011).
213. Ko, A. *et al.* Amerindian-specific regions under positive selection harbour new lipid variants in Latinos. *Nat Commun* **5**, 3983 (2014).
214. Kamatani, Y. *et al.* Genome-wide association study of hematological and biochemical traits in a Japanese population. *Nat Genet* **42**, 210-5 (2010).
215. Global Lipids Genetics, C. *et al.* Discovery and refinement of loci associated with lipid levels. *Nat Genet* **45**, 1274-83 (2013).
216. Aulchenko, Y.S. *et al.* Loci influencing lipid levels and coronary heart disease risk in 16 European population cohorts. *Nat Genet* **41**, 47-55 (2009).
217. Teslovich, T.M. *et al.* Biological, clinical and population relevance of 95 loci for blood lipids. *Nature* **466**, 707-13 (2010).
218. Dastani, Z. *et al.* Novel loci for adiponectin levels and their influence on type 2 diabetes and metabolic traits: a multi-ethnic meta-analysis of 45,891 individuals. *PLoS Genet* **8**, e1002607 (2012).

219. Chambers, J.C. *et al.* Genome-wide association study identifies loci influencing concentrations of liver enzymes in plasma. *Nat Genet* **43**, 1131-8 (2011).
220. Barrett, J.C. *et al.* Genome-wide association defines more than 30 distinct susceptibility loci for Crohn's disease. *Nat Genet* **40**, 955-62 (2008).
221. Franke, A. *et al.* Genome-wide meta-analysis increases to 71 the number of confirmed Crohn's disease susceptibility loci. *Nat Genet* **42**, 1118-25 (2010).
222. Yu, B. *et al.* Genome-wide association study of a heart failure related metabolomic profile among African Americans in the Atherosclerosis Risk in Communities (ARIC) study. *Genet Epidemiol* **37**, 840-5 (2013).
223. McArdle, P.F. *et al.* Association of a common nonsynonymous variant in GLUT9 with serum uric acid levels in old order amish. *Arthritis Rheum* **58**, 2874-81 (2008).
224. Wakil, S.M. *et al.* Association of a mutation in LACC1 with a monogenic form of systemic juvenile idiopathic arthritis. *Arthritis Rheumatol* **67**, 288-95 (2015).
225. Wang, J. *et al.* Genome-wide association analysis implicates the involvement of eight loci with response to tocilizumab for the treatment of rheumatoid arthritis. *Pharmacogenomics J* **13**, 235-41 (2013).
226. Lyons, P.A. *et al.* Genetically distinct subsets within ANCA-associated vasculitis. *N Engl J Med* **367**, 214-23 (2012).
227. Ahmeti, K.B. *et al.* Age of onset of amyotrophic lateral sclerosis is modulated by a locus on 1p34.1. *Neurobiol Aging* **34**, 357 e7-19 (2013).
228. Zheng, J.S. *et al.* Genome-wide contribution of genotype by environment interaction to variation of diabetes-related traits. *PLoS One* **8**, e77442 (2013).
229. Sokolik, R. *et al.* Significance of association of HLA-C and HLA-E with psoriatic arthritis. *Hum Immunol* **75**, 1188-91 (2014).
230. Smigoc Schweiger, D. *et al.* Genetic risk for co-occurrence of type 1 diabetes and celiac disease is modified by HLA-C and killer immunoglobulin-like receptors. *Tissue Antigens* **84**, 471-8 (2014).
231. Matsuzaka, Y. *et al.* Identification of novel candidate genes in the diffuse panbronchiolitis critical region of the class I human MHC. *Immunogenetics* **54**, 301-9 (2002).
232. Hancock, D.B. *et al.* Genome-wide joint meta-analysis of SNP and SNP-by-smoking interaction identifies novel loci for pulmonary function. *PLoS Genet* **8**, e1003098 (2012).
233. Thorleifsson, G. *et al.* Genome-wide association yields new sequence variants at seven loci that associate with measures of obesity. *Nat Genet* **41**, 18-24 (2009).
234. Zhang, X. *et al.* Synthesis of 53 tissue and cell line expression QTL datasets reveals master eQTLs. *BMC Genomics* **15**, 532 (2014).
235. Goring, H.H. *et al.* Discovery of expression QTLs using large-scale transcriptional profiling in human lymphocytes. *Nat Genet* **39**, 1208-16 (2007).
236. Idaghdour, Y. *et al.* Geographical genomics of human leukocyte gene expression variation in southern Morocco. *Nat Genet* **42**, 62-7 (2010).
237. Heap, G.A. *et al.* Complex nature of SNP genotype effects on gene expression in primary human leucocytes. *BMC Med Genomics* **2**, 1 (2009).
238. Emilsson, V. *et al.* Genetics of gene expression and its effect on disease. *Nature* **452**, 423-8 (2008).
239. Fehrmann, R.S. *et al.* Trans-eQTLs reveal that independent genetic variants associated with a complex phenotype converge on intermediate genes, with a major role for the HLA. *PLoS Genet* **7**, e1002197 (2011).
240. Mehta, D. *et al.* Impact of common regulatory single-nucleotide variants on gene expression profiles in whole blood. *Eur J Hum Genet* **21**, 48-54 (2013).

241. Zhernakova, D.V. *et al.* DeepSAGE reveals genetic variants associated with alternative polyadenylation and expression of coding and non-coding transcripts. *PLoS Genet* **9**, e1003594 (2013).
242. Sasayama, D. *et al.* Identification of single nucleotide polymorphisms regulating peripheral blood mRNA expression with genome-wide significance: an eQTL study in the Japanese population. *PLoS One* **8**, e54967 (2013).
243. Landmark-Hoyvik, H. *et al.* Genome-wide association study in breast cancer survivors reveals SNPs associated with gene expression of genes belonging to MHC class I and II. *Genomics* **102**, 278-87 (2013).
244. Westra, H.J. *et al.* Systematic identification of trans eQTLs as putative drivers of known disease associations. *Nat Genet* **45**, 1238-43 (2013).
245. van Eijk, K.R. *et al.* Genetic analysis of DNA methylation and gene expression levels in whole blood of healthy human subjects. *BMC Genomics* **13**, 636 (2012).
246. Battle, A. *et al.* Characterizing the genetic basis of transcriptome diversity through RNA-sequencing of 922 individuals. *Genome Res* **24**, 14-24 (2014).
247. Benton, M.C. *et al.* Mapping eQTLs in the Norfolk Island genetic isolate identifies candidate genes for CVD risk traits. *Am J Hum Genet* **93**, 1087-99 (2013).
248. Narahara, M. *et al.* Large-scale East-Asian eQTL mapping reveals novel candidate genes for LD mapping and the genomic landscape of transcriptional effects of sequence variants. *PLoS One* **9**, e100924 (2014).
249. Quinlan, J. *et al.* Genomic architecture of sickle cell disease in West African children. *Front Genet* **5**, 26 (2014).
250. Wright, F.A. *et al.* Heritability and genomics of gene expression in peripheral blood. *Nat Genet* **46**, 430-7 (2014).
251. Schramm, K. *et al.* Mapping the genetic architecture of gene regulation in whole blood. *PLoS One* **9**, e93844 (2014).
252. Lock, E.F. *et al.* Joint eQTL assessment of whole blood and dura mater tissue from individuals with Chiari type I malformation. *BMC Genomics* **16**, 11 (2015).
253. Powell, J.E. *et al.* The Brisbane Systems Genetics Study: genetical genomics meets complex trait genetics. *PLoS One* **7**, e35430 (2012).
254. Pierce, B.L. *et al.* Mediation analysis demonstrates that trans-eQTLs are often explained by cis-mediation: a genome-wide analysis among 1,800 South Asians. *PLoS Genet* **10**, e1004818 (2014).
255. Chen, W. *et al.* Expression quantitative trait loci (eQTL) mapping in Puerto Rican children. *PLoS One* **10**, e0122464 (2015).
256. Foroughi Asl, H. *et al.* Expression quantitative trait Loci acting across multiple tissues are enriched in inherited risk for coronary artery disease. *Circ Cardiovasc Genet* **8**, 305-15 (2015).
257. Dixon, A.L. *et al.* A genome-wide association study of global gene expression. *Nat Genet* **39**, 1202-7 (2007).
258. Liang, L. *et al.* A cross-platform analysis of 14,177 expression quantitative trait loci derived from lymphoblastoid cell lines. *Genome Res* **23**, 716-26 (2013).
259. Stranger, B.E. *et al.* Population genomics of human gene expression. *Nat Genet* **39**, 1217-24 (2007).
260. Kwan, T. *et al.* Genome-wide analysis of transcript isoform variation in humans. *Nat Genet* **40**, 225-31 (2008).
261. Dimas, A.S. *et al.* Common regulatory variation impacts gene expression in a cell type-dependent manner. *Science* **325**, 1246-50 (2009).

262. Cusanovich, D.A. *et al.* The combination of a genome-wide association study of lymphocyte count and analysis of gene expression data reveals novel asthma candidate genes. *Hum Mol Genet* **21**, 2111-23 (2012).
263. Grundberg, E. *et al.* Mapping cis- and trans-regulatory effects across multiple tissues in twins. *Nat Genet* **44**, 1084-9 (2012).
264. Gutierrez-Arcelus, M. *et al.* Passive and active DNA methylation and the interplay with genetic variation in gene regulation. *Elife* **2**, e00523 (2013).
265. Mangravite, L.M. *et al.* A statin-dependent QTL for GATM expression is associated with statin-induced myopathy. *Nature* **502**, 377-80 (2013).
266. Bryois, J. *et al.* Cis and trans effects of human genomic variants on gene expression. *PLoS Genet* **10**, e1004461 (2014).
267. Huang, J. *et al.* eQTL mapping identifies insertion- and deletion-specific eQTLs in multiple tissues. *Nat Commun* **6**, 6821 (2015).
268. Naranbhai, V. *et al.* Genomic modulators of gene expression in human neutrophils. *Nat Commun* **6**, 7545 (2015).
269. Andiappan, A.K. *et al.* Genome-wide analysis of the genetic regulation of gene expression in human neutrophils. *Nat Commun* **6**, 7971 (2015).
270. Fairfax, B.P. *et al.* Genetics of gene expression in primary immune cells identifies cell type-specific master regulators and roles of HLA alleles. *Nat Genet* **44**, 502-10 (2012).
271. Murphy, A. *et al.* Mapping of numerous disease-associated expression polymorphisms in primary peripheral blood CD4+ lymphocytes. *Hum Mol Genet* **19**, 4745-57 (2010).
272. Heinzen, E.L. *et al.* Tissue-specific genetic control of splicing: implications for the study of complex traits. *PLoS Biol* **6**, e1 (2008).
273. Zeller, T. *et al.* Genetics and beyond--the transcriptome of human monocytes and disease susceptibility. *PLoS One* **5**, e10693 (2010).
274. Almlof, J.C. *et al.* Powerful identification of cis-regulatory SNPs in human primary monocytes using allele-specific gene expression. *PLoS One* **7**, e52260 (2012).
275. Kirsten, H. *et al.* Dissecting the genetics of the human transcriptome identifies novel trait-related trans-eQTLs and corroborates the regulatory relevance of non-protein coding locidagger. *Hum Mol Genet* **24**, 4746-63 (2015).
276. Almlof, J.C. *et al.* Single nucleotide polymorphisms with cis-regulatory effects on long non-coding transcripts in human primary monocytes. *PLoS One* **9**, e102612 (2014).
277. Fairfax, B.P. *et al.* Innate immune activity conditions the effect of regulatory variants upon monocyte gene expression. *Science* **343**, 1246949 (2014).
278. Barreiro, L.B. *et al.* Deciphering the genetic architecture of variation in the immune response to Mycobacterium tuberculosis infection. *Proc Natl Acad Sci U S A* **109**, 1204-9 (2012).
279. Lee, M.N. *et al.* Common genetic variants modulate pathogen-sensing responses in human dendritic cells. *Science* **343**, 1246980 (2014).
280. Huang, R.S. *et al.* Population differences in microRNA expression and biological implications. *RNA Biol* **8**, 692-701 (2011).
281. Fischer, D. *et al.* MiRNA Profiles in Lymphoblastoid Cell Lines of Finnish Prostate Cancer Families. *PLoS One* **10**, e0127427 (2015).
282. Degner, J.F. *et al.* DNase I sensitivity QTLs are a major determinant of human expression variation. *Nature* **482**, 390-4 (2012).
283. del Rosario, R.C. *et al.* Sensitive detection of chromatin-altering polymorphisms reveals autoimmune disease mechanisms. *Nat Methods* **12**, 458-64 (2015).
284. Battle, A. *et al.* Genomic variation. Impact of regulatory variation from RNA to protein. *Science* **347**, 664-7 (2015).

285. Zhang, X. *et al.* Identification of common genetic variants controlling transcript isoform variation in human whole blood. *Nat Genet* **47**, 345-52 (2015).
286. Huan, T. *et al.* Genome-wide identification of microRNA expression quantitative trait loci. *Nat Commun* **6**, 6601 (2015).
287. Greenawalt, D.M. *et al.* A survey of the genetics of stomach, liver, and adipose gene expression from a morbidly obese cohort. *Genome Res* **21**, 1008-16 (2011).
288. Kompass, K.S. & Witte, J.S. Co-regulatory expression quantitative trait loci mapping: method and application to endometrial cancer. *BMC Med Genomics* **4**, 6 (2011).
289. Zhang, B. *et al.* Integrated systems approach identifies genetic nodes and networks in late-onset Alzheimer's disease. *Cell* **153**, 707-20 (2013).
290. Schadt, E.E. *et al.* Mapping the genetic architecture of gene expression in human liver. *PLoS Biol* **6**, e107 (2008).
291. Innocenti, F. *et al.* Identification, replication, and functional fine-mapping of expression quantitative trait loci in primary human liver tissue. *PLoS Genet* **7**, e1002078 (2011).
292. Schroder, A. *et al.* Genomics of ADME gene expression: mapping expression quantitative trait loci relevant for absorption, distribution, metabolism and excretion of drugs in human liver. *Pharmacogenomics J* **13**, 12-20 (2013).
293. Wang, X. *et al.* Mapping of hepatic expression quantitative trait loci (eQTLs) in a Han Chinese population. *J Med Genet* **51**, 319-26 (2014).
294. Grundberg, E. *et al.* Population genomics in a disease targeted primary cell model. *Genome Res* **19**, 1942-52 (2009).
295. Kabakchiev, B. & Silverberg, M.S. Expression quantitative trait loci analysis identifies associations between genotype and gene expression in human intestine. *Gastroenterology* **144**, 1488-96, 1496 e1-3 (2013).
296. Ongen, H. *et al.* Putative cis-regulatory drivers in colorectal cancer. *Nature* **512**, 87-90 (2014).
297. Hulur, I. *et al.* Enrichment of inflammatory bowel disease and colorectal cancer risk variants in colon expression quantitative trait loci. *BMC Genomics* **16**, 138 (2015).
298. Keildson, S. *et al.* Expression of phosphofructokinase in skeletal muscle is influenced by genetic variation and associated with insulin sensitivity. *Diabetes* **63**, 1154-65 (2014).
299. Quigley, D.A. *et al.* The 5p12 breast cancer susceptibility locus affects MRPS30 expression in estrogen-receptor positive tumors. *Mol Oncol* **8**, 273-84 (2014).
300. Curtis, C. *et al.* The genomic and transcriptomic architecture of 2,000 breast tumours reveals novel subgroups. *Nature* **486**, 346-52 (2012).
301. Hao, K. *et al.* Lung eQTLs to help reveal the molecular underpinnings of asthma. *PLoS Genet* **8**, e1003029 (2012).
302. Gao, C. *et al.* HEFT: eQTL analysis of many thousands of expressed genes while simultaneously controlling for hidden factors. *Bioinformatics* **30**, 369-76 (2014).
303. Lamontagne, M. *et al.* Refining susceptibility loci of chronic obstructive pulmonary disease with lung eqtls. *PLoS One* **8**, e70220 (2013).
304. Luo, W. *et al.* Airway Epithelial Expression Quantitative Trait Loci Reveal Genes Underlying Asthma and Other Airway Diseases. *Am J Respir Cell Mol Biol* (2015).
305. Ding, J. *et al.* Gene expression in skin and lymphoblastoid cells: Refined statistical method reveals extensive overlap in cis-eQTL signals. *Am J Hum Genet* **87**, 779-89 (2010).
306. Wagner, J.R. *et al.* The relationship between DNA methylation, genetic and expression inter-individual variation in untransformed human fibroblasts. *Genome Biol* **15**, R37 (2014).
307. Qiu, W. *et al.* Genetics of sputum gene expression in chronic obstructive pulmonary disease. *PLoS One* **6**, e24395 (2011).

308. Fadista, J. *et al.* Global genomic and transcriptomic analysis of human pancreatic islets reveals novel genes influencing glucose metabolism. *Proc Natl Acad Sci U S A* **111**, 13924-9 (2014).
309. Larson, N.B. *et al.* Comprehensively evaluating cis-regulatory variation in the human prostate transcriptome by using gene-level allele-specific expression. *Am J Hum Genet* **96**, 869-82 (2015).
310. Singh, T. *et al.* Characterization of expression quantitative trait loci in the human colon. *Inflamm Bowel Dis* **21**, 251-6 (2015).
311. Koopmann, T.T. *et al.* Genome-wide identification of expression quantitative trait loci (eQTLs) in human heart. *PLoS One* **9**, e97380 (2014).
312. Lin, H. *et al.* Gene expression and genetic variation in human atria. *Heart Rhythm* **11**, 266-71 (2014).
313. Rantalainen, M. *et al.* MicroRNA expression in abdominal and gluteal adipose tissue is associated with mRNA expression levels and partly genetically driven. *PLoS One* **6**, e27338 (2011).
314. Gamazon, E.R. *et al.* A genome-wide integrative study of microRNAs in human liver. *BMC Genomics* **14**, 395 (2013).
315. Olsson, A.H. *et al.* Genome-wide associations between genetic and epigenetic variation influence mRNA expression and insulin secretion in human pancreatic islets. *PLoS Genet* **10**, e1004735 (2014).
316. Li, Q. *et al.* Expression QTL-based analyses reveal candidate causal genes and loci across five tumor types. *Hum Mol Genet* **23**, 5294-302 (2014).
317. Webster, J.A. *et al.* Genetic control of human brain transcript expression in Alzheimer disease. *Am J Hum Genet* **84**, 445-58 (2009).
318. Zou, F. *et al.* Brain expression genome-wide association study (eGWAS) identifies human disease-associated variants. *PLoS Genet* **8**, e1002707 (2012).
319. Kim, Y. *et al.* A meta-analysis of gene expression quantitative trait loci in brain. *Transl Psychiatry* **4**, e459 (2014).
320. Ramasamy, A. *et al.* Genetic variability in the regulation of gene expression in ten regions of the human brain. *Nat Neurosci* **17**, 1418-28 (2014).
321. Gibbs, J.R. *et al.* Abundant quantitative trait loci exist for DNA methylation and gene expression in human brain. *PLoS Genet* **6**, e1000952 (2010).
322. Gamazon, E.R. *et al.* Enrichment of cis-regulatory gene expression SNPs and methylation quantitative trait loci among bipolar disorder susceptibility variants. *Mol Psychiatry* **18**, 340-6 (2013).
323. Kim, S., Cho, H., Lee, D. & Webster, M.J. Association between SNPs and gene expression in multiple regions of the human brain. *Transl Psychiatry* **2**, e113 (2012).
324. Shpak, M. *et al.* An eQTL analysis of the human glioblastoma multiforme genome. *Genomics* **103**, 252-63 (2014).
325. Colantuoni, C. *et al.* Temporal dynamics and genetic control of transcription in the human prefrontal cortex. *Nature* **478**, 519-23 (2011).
326. Liu, C. *et al.* Whole-genome association mapping of gene expression in the human prefrontal cortex. *Mol Psychiatry* **15**, 779-84 (2010).
327. Irizarry, R.A. *et al.* Exploration, normalization, and summaries of high density oligonucleotide array probe level data. *Biostatistics* **4**, 249-64 (2003).
